# Substitutive Approach Toward Heteroaromatic Amino Alcohols Accessed Through Dioxolanyl Radical Linchpin

**DOI:** 10.1002/adsc.70405

**Published:** 2026-03-31

**Authors:** Justin J. Chang, Munnu Kumar, Ryan C. Kashatus, Dylan J. Tomaselli, Daniel K. Kim

**Affiliations:** Department of Chemistry, Temple University, 1901 North 13th Street, Philadelphia, Pennsylvania, USA

**Keywords:** acetal radical, amino alcohol, aryl ethanolamine, dioxolanyl radical, vicinal functionalization

## Abstract

Aryl ethanolamines are an important functional group found in a variety of pharmaceuticals and natural products. For the past century, the main strategies for synthesizing these compounds are through Henry reactions followed by reductions or olefin oxidations using aldehyde or styrene starting materials. Herein, we disclose a highly adaptable synthesis of heteroaromatic amino alcohols enabled by a dioxolanyl radical linchpin strategy to take advantage of highly abundant aryl bromides and complex amine precursors. This methodology has been demonstrated across a variety of substrates, including a diverse set of thirteen distinct heteroarene classes. After mild deprotection, these diols can be simply activated and functionalized for chemoselective amino alcohol formation. The chosen retrosynthetic disconnection allows the incorporation of commercially available amines directly in synthesis.

## 1 | Introduction

**FIGURE 1 |:**
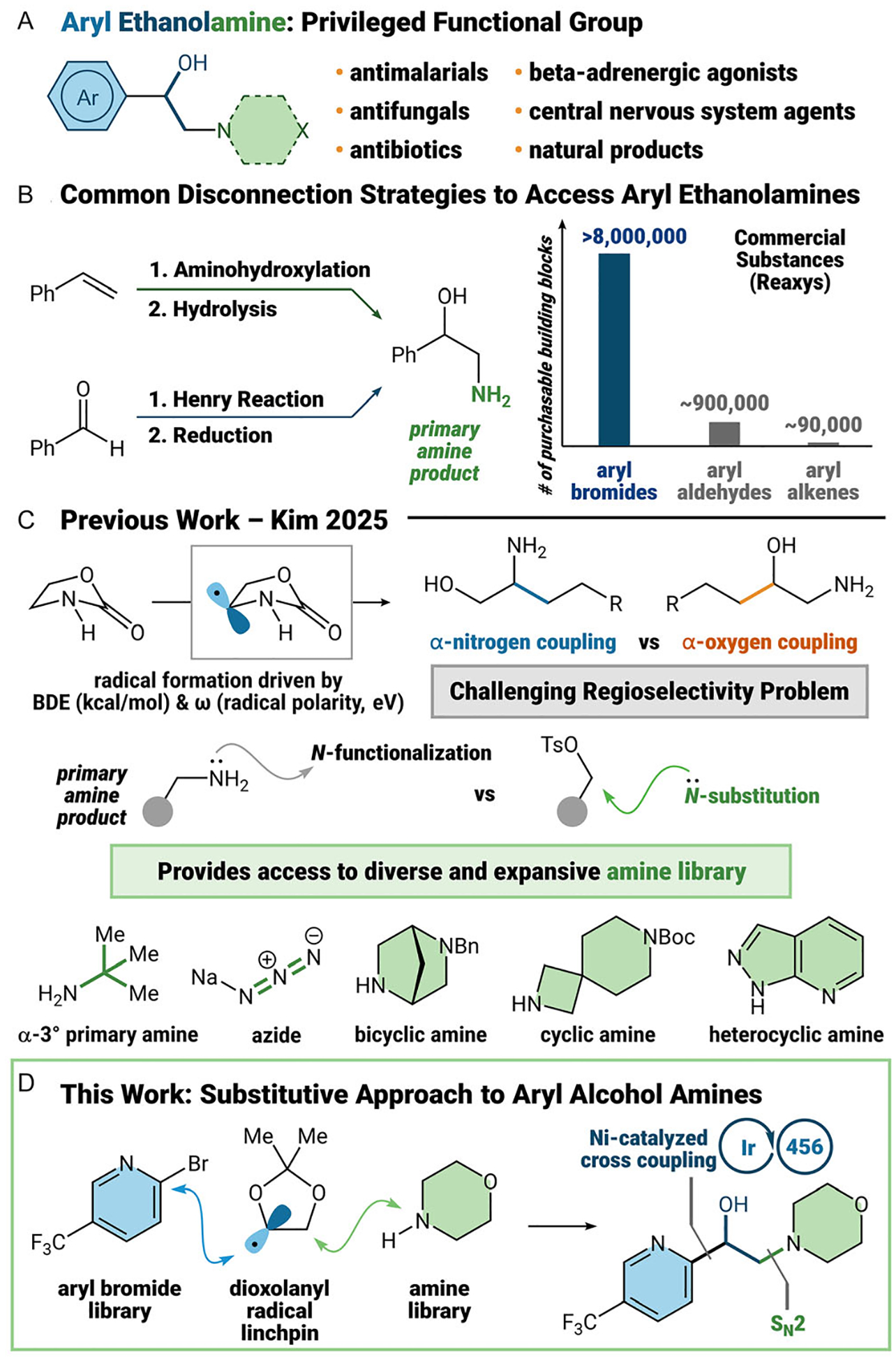
Application and Synthesis of Aryl Ethanolamines.

Aryl ethanolamines are a privileged synthetic functional group, which are present across a variety of drug classes, including anti-malarials [1, 2], antifungals [3, 4], antibiotics [5, 6], central nervous system agents [7, 8], and beta-adrenergic agonists [9, 10]. This ubiquitous pharmaceutical motif has been inspired by a variety of notable natural products, including epinephrine, adrenaline, and pseudoephedrine (Figure 1A) [11].

Over the past century, two retrosynthetic disconnections have emerged as the dominant way synthetic chemists construct this highly sought-after motif. Oxidation reactions of alkenes remain the most powerful and the most common way, either by epoxidation/substitution or Sharpless asymmetric aminohydroxylation [12–15]. A classical C─C bond forming approach to amino alcohols uses a Henry reaction with nitromethane, followed by reduction to the amine (Figure 1B) [16–19]. Recently, our lab has been focused on the use of acetal radicals to advance the molecular synthesis of vicinal functional groups. As such, we postulated that important aryl ethanolamines could be accessed through these radicals.

In our prior work, our group accessed 1,2-diols through a dioxolanyl radical, generated from hydrogen atom transfer (HAT) [20]. We disclosed the synthesis of 1,2-amino alcohols using oxazolidinone, on which HAT catalysis regioselectively favors the α-nitrogen carbon due to lower bond dissociation energy of the C─H bond (BDE, 84.7 vs. 87.4 kcal/mol α-oxygen C─H bond) and radical polarity for the α-amino radical (ω, 0.88 vs.0.93 eV α-oxy radical) [20]. Therefore, this presents a challenging regioselectivity problem with radical formation in our efforts toward aryl ethanolamines (Figure 1C).

On top of a regioselectivity challenge, we also wanted to gain access to the broadest scope of aryl ethanolamines that would be of practical use to expert users. To address both of these challenges, we devised a modular synthesis of aryl ethanolamines that uses a dioxolanyl radical as a key linchpin.

Given the high adoption of transition metal catalysis, the use of aryl halides has become an attractive starting point for synthesis. Aryl bromides, which are ten times more abundant than their aldehyde and olefin counterparts, can be leveraged to introduce a diverse catalog of complex heteroaryl ethanolamines [21]. As such, we thought to employ nickel-catalyzed cross-coupling as a powerful method to form C*sp*^2^–C*sp*^3^ bonds with dioxolanyl radicals. Furthermore, in contrast to our prior work, the direct installation of a primary amine is amenable to *N*-functionalization reactions like alkylation/acylation, reductive amination, or heterocycle condensation [22]. In order to increase the diversity of chemical space, we propose a substitutive approach to incorporate commercially available cyclic, heteroaromatic, and sterically encumbered amines (Figure 1C). This combinatorial approach would allow us to integrate the expansive libraries of commercially available aryl bromides and amines to access the broadest range of chemical space toward aryl ethanolamines (Figure 1D).

Finally, as part of our desire to access the broadest scope of aryl ethanolamines we envisioned a racemic route toward the synthesis of our chiral aryl ethanolamine products [24]. Due to the prevalence of heteroaromatics in drug discovery and synthesis, we wanted to develop a nickel-catalyzed arylation of dioxolanyl radicals. Based on literature, we recognized two common strategies for forming the acetal radical, either by direct HAT or by oxidative decarboxylation [25]. As such, we explored both methods across different classes of heteroaromatic rings to evaluate the generality of these methods.

We optimized both HAT and oxidative decarboxylation conditions with 2-bromo-5-trifluoromethylpyridine, using potassium salt **1** for the oxidative decarboxylation (Table 1). Initially, it seemed that HAT gave higher coupling yields (**2**, 81% versus 90% yield). However, photocatalyst-mediated decarboxylation proved the most robust across a range of electronically diverse substrates. Yields were higher using decarboxylation with electron-rich furan substrate **3** (58% versus 38% yield) and electron-rich benzofuran substrate **4** (47% versus 26% yield). Additionally, in general, yields were higher using decarboxylation with electron-deficient pyridine substrate **5** (60% versus 25% yield). Thus, we opted to move forward using our oxidative decarboxylation conditions. Control reactions show that light and photocatalyst both are essential for reactivity (see Supporting Information for more details).

**FIGURE 2 |:**
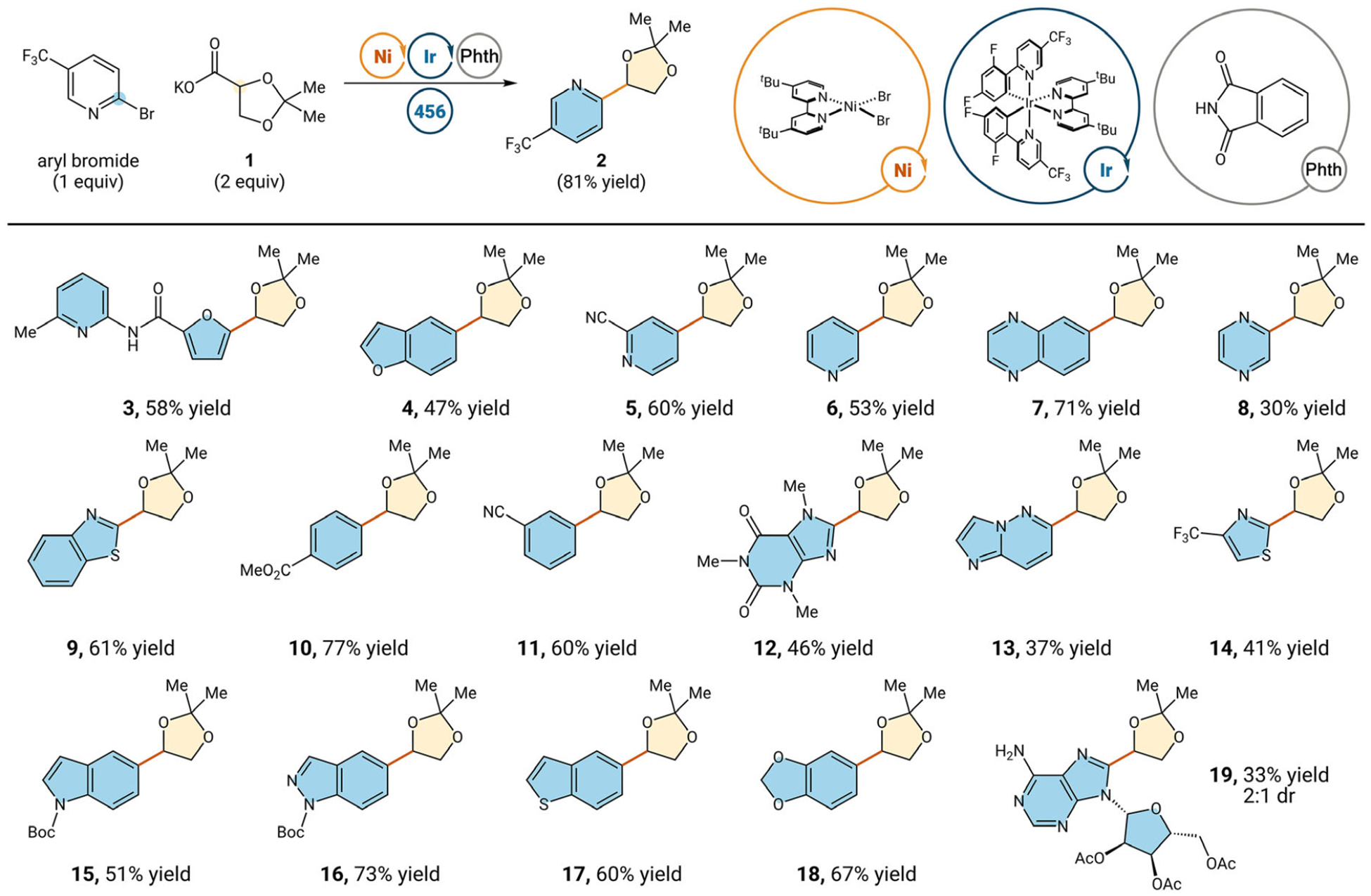
Evaluation of metallaphotoredox acetal reaction. See Supporting Information for reaction details.

The inclusion of phthalimide as a stoichiometric additive helped improve yield by limiting des-bromination and aryl dimerization pathways (**2a** and **2b**, respectively), leading to an overall increase in yield (81% vs. 67% yield, See Supporting Information for more details) [23]. With optimized conditions in hand, we evaluated a wide variety of heteroaryl and aryl bromides for our cross-coupling protocol (Figure 2).

Electron-deficient heterocycles like pyridine, pyrazine, quinoxa-line, and benzothiazole (**6–9**) are all well-tolerated for this protocol, giving modest to high yields (30%–71% yield). Electron-deficient aryls (10 and 11) also couple in good yields (**77**% and 60%, respectively). More challenging caffeine-derived substrate (**12**) couples in 46% yield. Additionally, challenging heterocycles like imidazo[1,2-*b*]pyridazine and thiazole (**13** and **14**) couple in moderate yields (37% and 41% yield, respectively) [26, 27]. Excitingly, more electron-rich aryls also reacted smoothly in this protocol. Nickel cross-coupling reactions can often suffer lower yields using electron-rich aryl halides due to slower oxidative addition [28]. However, coupling proceeds smoothly using electron-rich indole and indazole (**15** and **16**) in good yield (51% and 73%, respectively). Additionally, benzothiophene substrate (**17**) proved to couple effectively in 60% yield. Benzo[*d*][1,3]dioxole substrate (**18**) is well suited for this oxidative decarboxylation protocol compared to HAT, as it bears a labile C—H bond that may lead to undesired side products under HAT conditions [29]. Using this methodology, 67% yield is observed. Lastly, this protocol is amenable to coupling with acylated adenosine-derived substrate (**19**) in a modest 33% yield, tolerating the unprotected amine. We wanted to demonstrate that these heteroaryl acetonides could be deprotected in a facile manner with minimal purification required. Thus, we can take the acetonide material from the nickel cross-coupling and subject it to mildly acidic conditions, revealing the 1,2-diol motif without any purification (**20**, 81% yield over two steps).

**FIGURE 3 |:**
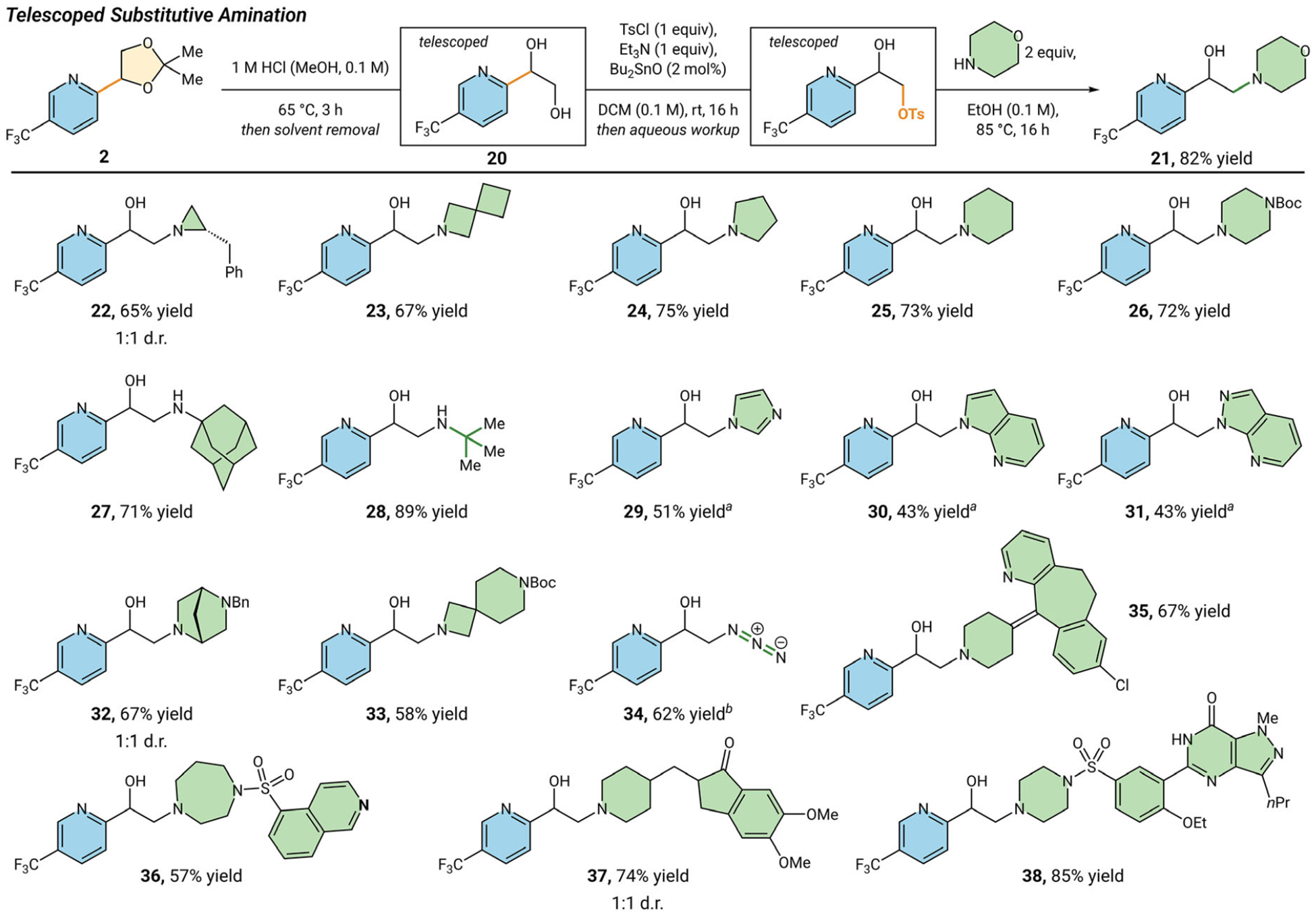
Evaluation of two-pot activation/substitution reaction sequence. ^a^Reaction was conducted with amine (1 equiv), Cs_2_CO_3_ (3 equiv) in DMSO (0.1 M). ^b^Reaction was conducted with NaN_3_ (3 equiv) in DMF (1.2 M). See Supporting Information for reaction details.

With a robust synthesis of aryl and heteroaryl acetonides in hand, we explored strategies to selectively activate and substitute the homobenzylic alcohol to access the aryl ethanolamine selectively (Figure 3). Leaning on strategies developed for selective *O*-functionalization of sugars and other polyols in the synthesis of glycosides, we employed a catalytic tin-mediated tosylation procedure to chemoselectively activate the primary alcohol [30, 31]. This substitution approach is complementary to functionalization of the free amine, allowing access to cyclic, aromatic, and sterically bulky amines. We first examined morpholine (**21**) as a medicinally relevant benchmark for the chemistry, which coupled in 82% yield. Pleased with this result, we explored a variety of differentially sized cyclic amines.

Aziridines, azetidines, pyrrolidines, and piperidines (**22–25**) are well tolerated with yields ranging from 65% to 75% yield. Piperazine (**26**) was also amenable (72% yield), which could lend itself as a valuable linker. It was important to us to explore α−3° primary amines as well, because this class can be difficult to synthesize using substitution strategies due to the increased steric bulk. To test if our approach was compatible with bulky amines, we tried adamantylamine (**27**) which coupled well in 72% yield. Additionally, substitution with *tert*-butyl amine (**28**) proceeds in 89% yield. Aromatic amines (**29–31**) work in 51% and 43% yield, respectively. Bicyclic and spirocyclic amines **32** and **33** afford the resulting aryl ethanolamine motif in good yields. Other nitrogen nucleophiles, like azide (**34**) can also be leveraged, coupling in 62% yield. Lastly, we wanted to examine how large, molecularly complex amines would fare. Desloratadine (**35**) coupled modestly in 67% yield. Excitingly, seven-membered diazacycle (**36**) works in 57% yield. Other late-stage drug molecules like desbenzylated donepezil (**37**) and desmethylated sildenafil (**38**) work well in 74% and 85% yield, respectively.

**FIGURE 4 |:**
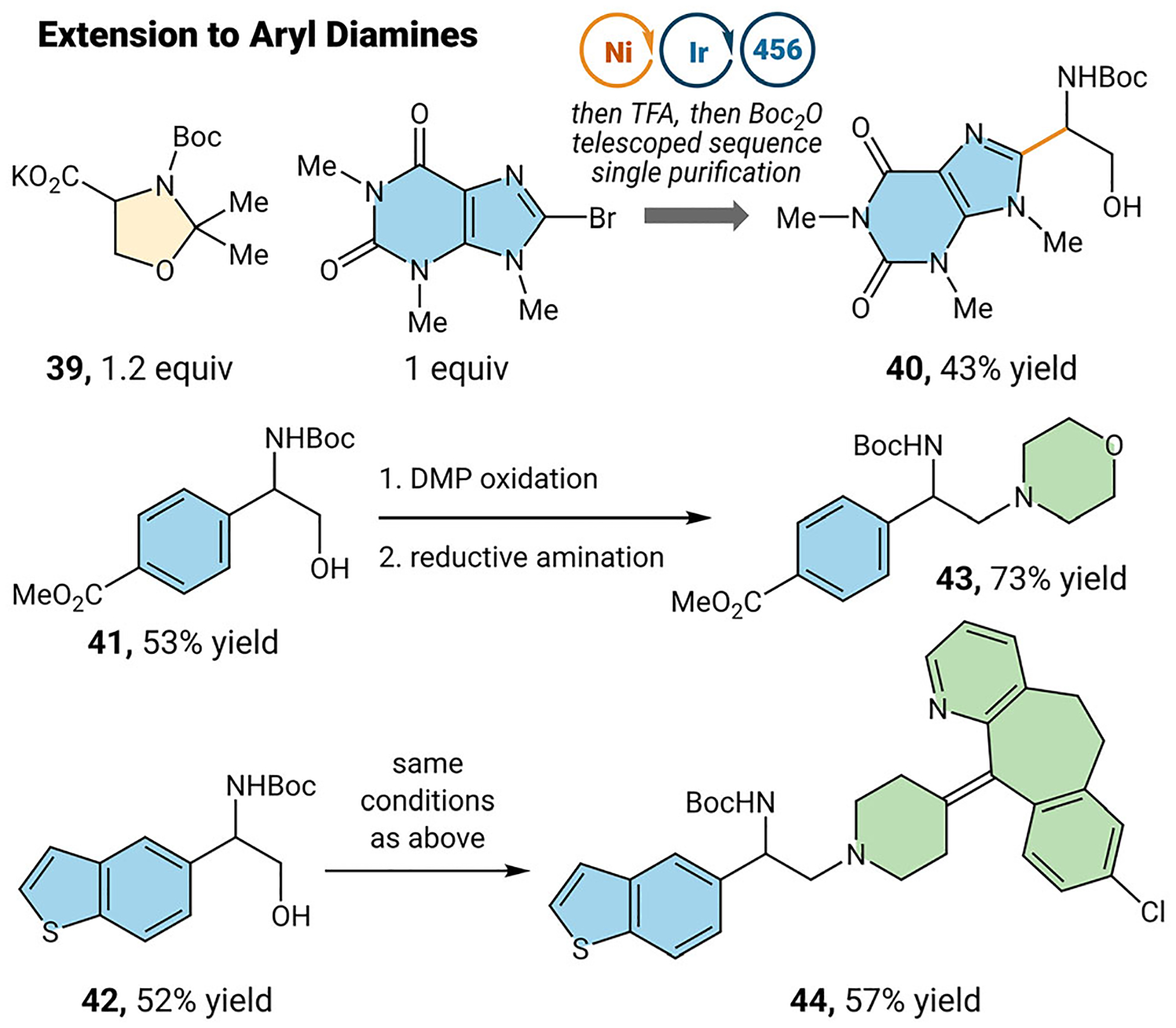
Synthesis of aryl 1,2-diamines. See Supporting Information for reaction details.

Lastly, we wanted to demonstrate further applications of this strategy in the synthesis of diamine compounds (Figure 4). 1,2-diamines are prevalent across a variety of natural products [32], drug molecules [33], and versatile ligand scaffolds [34]. However, it can be difficult to selectively functionalize each nitrogen due to similarities in reactivity. Using a cross-coupling/substitutive strategy, we can leverage an electrophile and a nucleophilic nitrogen to selectively access differentially substituted diamine compounds. For example, L-serine derived reagent **39** can be used to couple with aryl and heteroaryl bromides. This crude mixture can be telescoped into mild acid deprotection and *N*-Boc protection to access reversed amino alcohols **40**, **41**, and **42** (43% to 53% yield). Furthermore, once deprotected, the alcohol can be oxidized to the aldehyde, and reductive amination affords the terminally substituted diamine compound **43** in good yield. Additionally, we can again take complex drug molecules like desloratadine and use this as a nucleophilic amine to access complex diamine **44**.

In conclusion, we have established a combinatorial synthesis of aryl ethanolamines using dioxolanyl radicals as a linchpin through a photoredox-mediated nickel cross-coupling/substitution protocol. Leveraging the vast amount of available heteroaryl bromides, we have demonstrated coupling across a wide variety of both electron-deficient and electron-rich aromatics and heteroaromatics, representing thirteen distinct heterocyclic classes. These acetals can easily be deprotected, functionalized, and derivatized to access a wide scope of aryl ethanolamines from commercially available, complex amine building blocks. Lastly, we further demonstrated this strategy to access differentially substituted aryl diamine products. This protocol establishes a streamlined modular approach to the synthesis of aryl ethanolamines, offering a practical platform for rapid exploration in drug discovery.

## Supplementary Material

SI

Additional supporting information can be found online in the Supporting Information section. Supporting Fig. S1: Solvent Screen. Supporting Fig. S2: Nickel Loading. Supporting Fig. S3: Additive Screening. Supporting Fig. S4: Control Experiments and Water Additive Screening. Supporting Fig. S5: Base Screening. Supporting Fig. S6: Solvent Screening.

## Figures and Tables

**TABLE 1 |:** Optimization of methods for Ni cross-coupling reaction. See Supporting Information for reaction details.

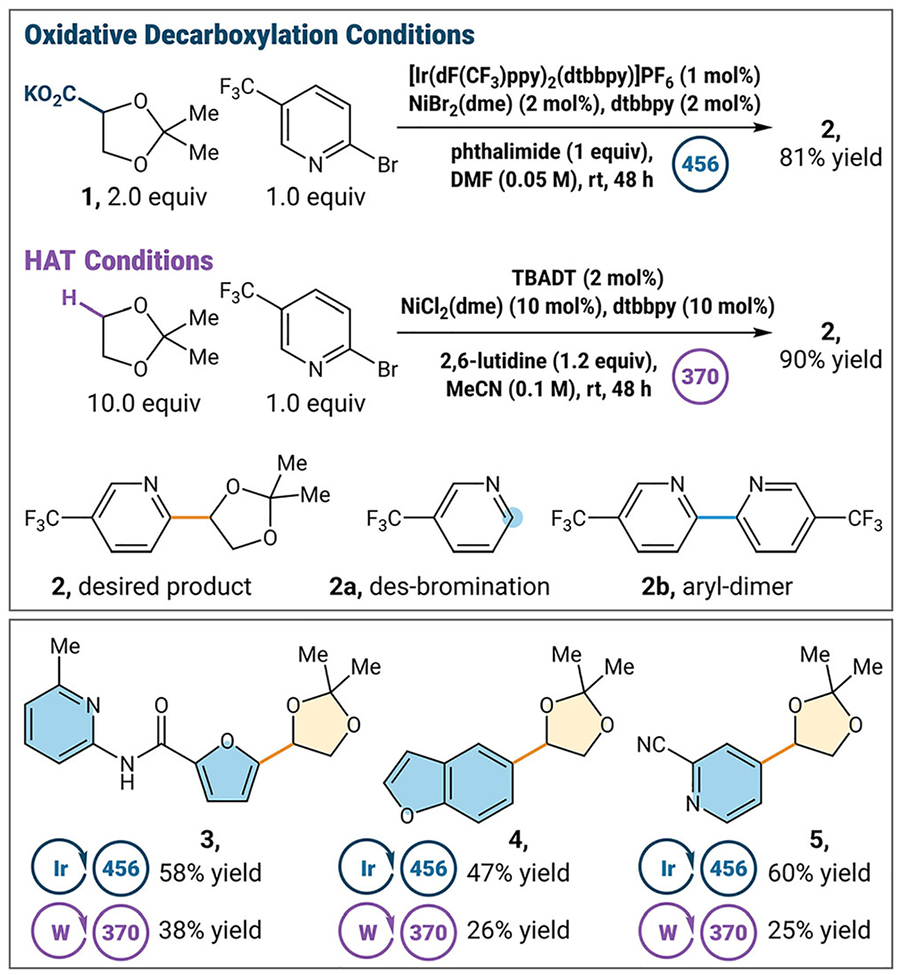

## Data Availability

The data that support the findings of this study are available in the Supporting Information of this communication.

## References

[1] Julianto TS, Rachmawaty FJ, Tamhid HA, and Putri BS, “The Potential Antimalarial Drug of Aryl Amino Alcohol Derivatives From Eugenol: Synthesis, In-vitro and In-silico Analysis of Bioactivity,” RASAYAN Journal of Chemistry 16 (2023): 1425–1434.

[2] Quiliano M, Mendoza A, Fong KY, , “Exploring the Scope of New Arylamino Alcohol Derivatives: Synthesis, Antimalarial Evaluation, Toxicological Studies, and Target Exploration,” International Journal for Parasitology: Drugs and Drug Resistance 6 (2016): 184–198. 27718413 10.1016/j.ijpddr.2016.09.004 PMC5061469

[3] Godefroi EF, Heeres J, Van Cutsem J, and Janssen PAJ,“Preparation and Antimycotic Properties of Derivatives of 1-Phenethylimidazole,” Journal of Medicinal Chemistry 12 (1969): 784–791. 4897900 10.1021/jm00305a014

[4] Espinel-Ingroff A, “Antifungal Agents,” In: Encyclopedia of Microbiology, (ed. Schaechter M), (Academic Press, 2009), 205–222.

[5] Schumacher DP, Clark JE, Murphy BL, and Fischer PA, “An Efficient Synthesis of Florfenicol,” The Journal of Organic Chemistry 55 (1990): 5291–5294.

[6] Gillet AD and Abdel-Monem MM, “Acid-Labile Derivatives of Chloramphenicol as Potential Latentiation Forms,” Journal of Medicinal Chemistry 16 (1973): 992–995. 4745515 10.1021/jm00267a008

[7] Kaufmann H, Norcliffe-Kaufmann L, and Palma J-A, “Droxidopa in Neurogenic Orthostatic Hypotension,” Expert Review of Cardiovascular Therapy 13 (2015): 875–891. 26092297 10.1586/14779072.2015.1057504 PMC4509799

[8] O’Donnell J, Zeppenfeld D, McConnell E, Pena S, and Nedergaard M, “Norepinephrine: A Neuromodulator That Boosts the Function of Multiple Cell Types to Optimize CNS Performance,” Neurochemical Research 37 (2012): 2496–2512. 22717696 10.1007/s11064-012-0818-x PMC3548657

[9] Jacobson KA, Marr-Leisy D, Rosenkranz RP, Verlander MS, Melmon KL, and Goodman M, “Conjugates of Catecholamines. 1. N-Alkyl-Functionalized Carboxylic Acid Congeners and Amides Related to Isoproterenol,” Journal of Medicinal Chemistry 26 (1983): 492–499. 6300399 10.1021/jm00358a007

[10] Xing G, Woo AY-H, Pan L, Lin B, and Cheng M-S, “Recent Advances in *β*_2_-Agonists for Treatment of Chronic Respiratory Diseases and Heart Failure,” Journal of Medicinal Chemistry 63 (2020): 15218–15242. 33213146 10.1021/acs.jmedchem.0c01195

[11] Tang K-J, Zhao Y, Tao X, , “Catecholamine Derivatives: Natural Occurrence, Structural Diversity, and Biological Activity,” Journal of Natural Products 86 (2023): 2592–2619. 37856864 10.1021/acs.jnatprod.3c00465

[12] Kamble VT and Joshi NS, “Synthesis of *β*-Amino Alcohols by ring Opening of Epoxides with Amines Catalyzed by Cyanuric Chloride under Mild and Solvent-Free Conditions,” Green Chemistry Letters and Reviews 3 (2010): 275–281.

[13] Tyagi A, Yadav N, Khan J, Mondal S, Hazra CK, “Brønsted Acid-Catalysed Epoxide Ring-Opening Using Amine Nucleophiles: A Facile Access to *β*-Amino Alcohols,” Chemistry-An Asian Journal 17 (2022): e202200379. 35485456 10.1002/asia.202200379

[14] Demko ZP, Bartsch M, and Sharpless KB, “Primary Amides. A General Nitrogen Source for Catalytic Asymmetric Aminohydroxylation of Olefins,” Organic Letters 2 (2000): 2221–2223. 10930248 10.1021/ol000098m

[15] Prileschajew N, “Oxydation ungesättigter Verbindungen Mittels Organischer Superoxyde,” Berichte der deutschen chemischen Gesellschaft 42 (1909): 4811–4815.

[16] Buchanan DJ, Dixon DJ, Scott MS, and Lainé DI, “Short Asymmetric Syntheses of Bioactive β-Aryl Ethanolamine Derivatives via the Highly Diastereoselective Delta Lactol Oxy-Michael Addition,” Tetrahedron: Asymmetry 15 (2004): 195–197.

[17] Bergmeier SC, “The Synthesis of Vicinal Amino Alcohols,” Tetrahedron 56 (2000): 2561–2576.

[18] Kim HY and Oh K, “Brucine-Derived Amino Alcohol Catalyzed Asymmetric Henry Reaction: An Orthogonal Enantioselectivity Approach,” Organic Letters 11 (2009): 5682–5685. 20000444 10.1021/ol902380z

[19] Alvarez-Casao Y, Marques-Lopez E, and Herrera RP, “Organocatalytic Enantioselective Henry Reactions,” Symmetry 3 (2011): 220–245.

[20] Chang JJ, Kumar M, Okamoto M, Vergara-Pimentel ES, Kim DK, “Unified Hydrogen Atom Transfer Approach To Construct Vicinal Functionality,” Organic Letters 27 (2025): 4417–4422. 39913345 10.1021/acs.orglett.4c04767 PMC12054531

[21] Reaxys search from November 2025 of commercially available aryl fragments: aryl bromides (8,000,000), aryl aldehydes (900,000), and aryl alkenes (90,000).

[22] Sun J, Endo H, Emmanuel MA, Oderinde MS, Kawamata Y, and Baran PS, “Simplified Modular Access to Enantiopure 1,2-Aminoalcohols via Ni-Electrocatalytic Decarboxylative Arylation,” Journal of the American Chemical Society 146 (2024): 6209–6216. 38387466 10.1021/jacs.3c14119 PMC10962872

[23] Prieto Kullmer CN, Kautzky JA, Krska SW, Nowak T, Dreher SD, and MacMillan DWC, “Accelerating Reaction Generality and Mechanistic Insight through Additive Mapping,” Science 376 (2022): 532–539. 35482871 10.1126/science.abn1885 PMC9673503

[24] Xu S, Ping Y, Su Y, Guo H, Luo A, and Kong W, “A Modular Approach to Catalytic Stereoselective Synthesis of Chiral 1,2-Diols and 1,3-Diols,” Nature Communications 16 (2025): 364. 10.1038/s41467-024-55744-3 PMC11699147 39754022

[25] Zuo Z, Ahneman DT, Chu L, Terrett JA, Doyle AG, and MacMillan DWC, “Merging Photoredox with Nickel Catalysis: Coupling of *α*-Carboxyl sp^3^-Carbons with Aryl Halides,” Science 345 (2014): 437–440. 24903563 10.1126/science.1255525 PMC4296524

[26] Garry OL, Heilmann M, Chen J, , “Rapid Access to 2-Substituted Bicyclo[1.1.1]pentanes,” Journal of the American Chemical Society 145 (2023): 3092–3100. 36696089 10.1021/jacs.2c12163 PMC10680143

[27] He H and Zhong W, (2022). “Cdk2 inhibitors,” WO-2022155941-A1. Filed 25 January 2021, Published 28 July 2022.

[28] Mirabi B, Marchese AD, and Lautens M, “Nickel-Catalyzed Reductive Cross-Coupling of Heteroaryl Chlorides and Aryl Chlorides,” ACS Catalysis 11 (2021): 12785–12793.

[29] Capaldo L, Merli D, Fagnoni M, and Ravelli D, “Visible Light Uranyl Photocatalysis: Direct C–H to C–C Bond Conversion,” ACS Catalysis 9 (2019): 3054–3058.

[30] Guillaume M and Lang Y, “Further Improvements of the Dibutyl Tin Oxide-Catalyzed Regioselective Diol Tosylation,” Tetrahedron Letters 51 (2010): 579–582.

[31] Muramatsu W and Takemoto Y, “Selectivity Switch in the Catalytic Functionalization of Nonprotected Carbohydrates: Selective Synthesis in the Presence of Anomeric and Structurally Similar Carbohydrates under Mild Conditions,” The Journal of Organic Chemistry 78 (2013): 2336–2345. 23360236 10.1021/jo3024279

[32] Singh MP, Petersen PJ, Jacobus NV, Maiese WM, Greenstein M, and Steinberg DA, “Mechanistic Studies and Biological Activity of Bioxalomycin Alpha 2, a Novel Antibiotic Produced by Streptomyces Viridodiastaticus subsp. “litoralis” LL-31F508,” Antimicrobial Agents and Chemotherapy 38 (1994): 1808–1812. 7527199 10.1128/aac.38.8.1808 PMC284640

[33] Hatanaka M and Ishimaru T, “Synthetic Penicillins. Heterocyclic Analogs of Ampicillin. Structure-Activity Relations,” Journal of Medicinal Chemistry 16 (1973): 978–984. 4200871 10.1021/jm00267a005

[34] Endo K and Grubbs RH, “Chelated Ruthenium Catalysts for Z-Selective Olefin Metathesis,” Journal of the American Chemical Society 133 (2011): 8525–8527. 21563826 10.1021/ja202818v PMC3121191

