# Supplementary material for: Substitutive Approach Toward Heteroaromatic Amino Alcohols Accessed Through Dioxolanyl Radical Linchpin
Source: Adv Synth Catal. Author manuscript; Available in PMC 2026 May 20. (PMC13186441; doi:10.1002/adsc.70405)

Supporting Information

Substitutive Approach Towards Heteroaromatic Cyclic Amino Alcohols Accessed Through Dioxolanyl Linchpin

Justin J. Chang, Munnu Kumar, Ryan C. Kashatus, Dylan J. Tomaselli, Daniel K. Kim*

Department of Chemistry, Temple University
1901 North 13th Street, Philadelphia, Pennsylvania, 19122, United States

* Corresponding author. Email: danielkim@temple.edu
‡Denotes equal author contributions

Table of Contents

General Information 2

Reaction Optimization and Control Experiments 4

General Procedure for the Preparation of Starting Materials and Characterization. 8

Preparation of Acetonide Reagents and Characterization. 13

General Procedure for Decarboxylative Arylation and Characterization of All Products (2-20) 14

General Procedure for Substitution Reaction and Characterization of All Products (21-38) 27

General Procedure for Telescoped Decarboxylative Alkylation, Deprotection, and Characterization of Amino Alcohols 45

General Procedure for Telescoped Oxidation-Reductive Amination Sequence, and Characterization of Diamines (43-44) 48

References 50

^{1}H, ^{13}C NMR and ^{19}F NMR spectra of all compounds 51

General Information

Commercially available reagents were used without additional purification, unless otherwise indicated. Reaction vials (8 mL and 20 mL) were purchased from Fischer Scientific, oven dried overnight, and cooled to room temperature prior to use. Photocatalysts were purchased and used as received. Ir[dF(CF_3)ppy]$_2$(dtbbpy)PF_6 (Oakwood Chemical, PN: 099347) and Ir(ppy)$_3$ (Combi-Blocks, PN: QG-1453). Solvents were purified using Pure Process Technology 5-Solvent Purification System (DMF, MeCN, THF, DCM, Et_2O). Other solvents such as acetone and 1,4-dioxane were purchased as anhydrous solvents and stored in the glovebox. DMSO was purchased and used as received (no special care taken with regards to air and moisture).

Thin layer chromatography was carried out using TLC Silica gel 60 F_{254} plates (Sigma Aldrich, PN: 1.05715.0001). For flash column chromatography, bulk silica gel (230–400 mesh) was used (Natland International Corporation, PN: 80001-20). Purification was carried out using ACS grade solvents.

Nuclear magnetic resonance spectra (^{1}H NMR, ^{13}C NMR) were recorded on a Bruker Unity 400 MHz, 500 MHz, or 600 MHz spectrometers in $CDCl_3$ or D_2O solutions. ^{1}H NMR Data are reported in terms of chemical shift (δ, ppm), multiplicity (s = singlet, d = doublet, t = triplet, q = quartet, m = multiplet, br = broad), coupling constant (Hz), and integration. Chemical shifts are reported as parts per million (ppm) standardized to the resulting spectra were internally referenced to the residual proteo-solvent signals (7.26 ppm for $CDCl_3$; 4.79 ppm for D_2O). ^{13}C NMR data are reported in terms of chemical shift (δ, ppm), multiplicity (q = quartet), and coupling constant (Hz) when there is coupling. ^{19}F nuclei: Decoupled and Coupled. ^{19}F NMR spectroscopy was performed on a Bruker Unity 400 MHz or 500 MHz spectrometers in the same deuterated solvent as ^{1}H NMR and the resulting spectra are unreferenced.

High Resolution Mass Spectroscopy (HRMS): Accurate masses for derivatized products were conducted on an Agilent 6520 Accurate-Mass Q-TOF LC/MS. Samples were taken up in a suitable solvent (MeCN) for analysis. Accurate mass measurement (AMM) analyses were conducted on an LCT Premier XE, time-of-flight, LCMS with electrospray ionization (ESI-TOF). Samples were taken up in a suitable solvent for analysis. The signals were mass measured against an internal lock

mass reference of perfluorotributylamine (PFTBA) for EI-GCMS and leucine enkephalin for ESI-LCMS, positive and negative ion modes. Waters software calibrates the instruments and reports measurements, by use of neutral atomic masses. The mass of the electron was not included.

Photochemical reactions were carried out using Kessil PR160L 390 nm, 427 nm, 456 nm, 467 nm and 525 nm at 100% intensity. Reaction vials are set one cm away from the light source using Kessil's PR160 Rig with Fan Kit.

Reaction Optimization and Control Experiments

Nickel Cross-Coupling Optimization

Procedure for Optimization: To an 8 mL vial (**Vial 1**), equipped with a stir bar, was added respective photocatalyst, aryl bromide, potassium 2,2-dimethyl-1,3-dioxolane-4-carboxylate, and additive. To a separate 8 mL vial (**Vial 2**), nickel catalyst and ligand were added. Both vials were added into a nitrogen-filled glovebox. Respective solvent was added to each vial. **Vial 2** was allowed to stir for 10 min, at which point the contents of **Vial 2** were added to **Vial 1**. **Vial 1** was then sealed with electrical tape, removed from the glovebox, and irradiated with two 34W Kessil PR160 456 LEDs (1 cm away, with cooling from fans to keep reactions at room temperature) for 48 hours. Afterwards, the reactions are removed from the lights. The reaction mixture is diluted with water (50 mL), and extracted three times with EtOAc (30 mL). Trifluorotoluene was then added to the vial, which was allowed to stir for 15 minutes. After which, an aliquot of the reaction mixture was taken and crude yields were gathered using ^{19}F NMR.

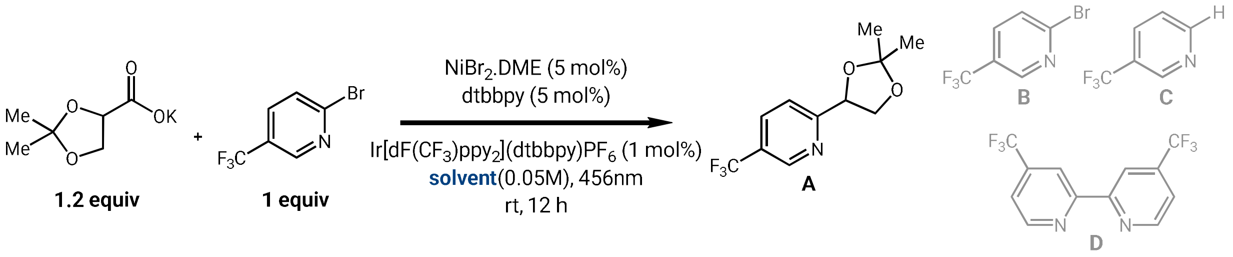

Entry	Reaction Conditions	Yield A	B	C	D
1	DMF	40%	12%	0%	2%
2	DMSO	30%	7%	0%	5%
3	DMA	27%	13%	0%	3%
4	ACN	0%	87%	0%	2%

Figure S1: Solvent Screen

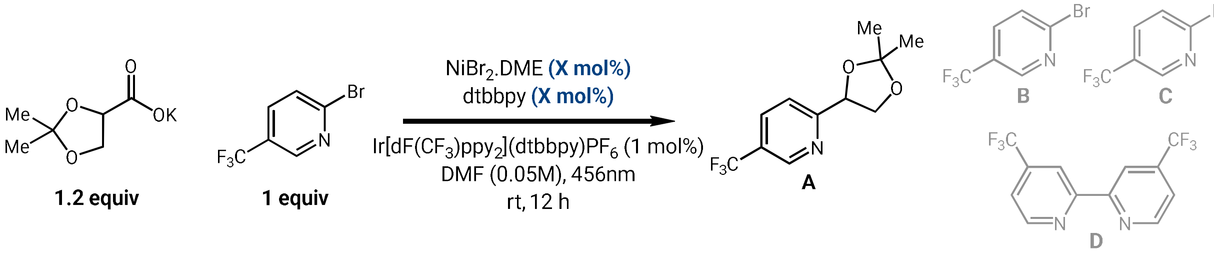

Entry	Reaction Conditions	Yield A	B	C	D
1	$NiBr_2$.DME (0.5 mol%), dtbbpy (0.5 mol%)	61%	16%	0%	4%
2	$NiBr_2$.DME (1 mol%), dtbbpy (1 mol%)	50%	33%	12%	0%
3	$NiBr_2$.DME (2 mol%), dtbbpy (2 mol%)	58%	23%	12%	4%
4	$NiBr_2$.DME (4 mol%), dtbbpy (4 mol%)	26%	21%	50%	2%
5	$NiBr_2$.DME (6 mol%), dtbbpy (6 mol%)	7%	44%	0%	10%
6	$NiBr_2$.DME (8 mol%), dtbbpy (8 mol%)	37%	12%	34%	5%

Figure S2: Nickel Loading

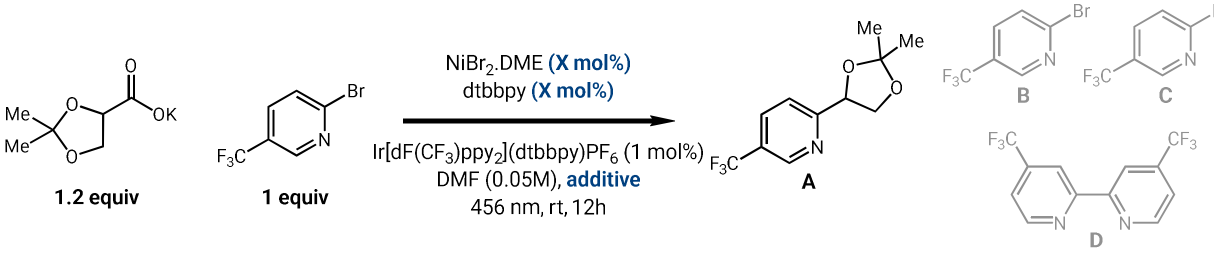

Entry	Reaction Conditions	Yield A	B	C	D
1	$NiBr_2$.DME (0.5 mol%), dtbbpy (0.5 mol%), Cs_2CO_3 (1.2 equiv)	0%	100%	0%	11%
2	$NiBr_2$.DME (1 mol%), dtbbpy (1 mol%), Cs_2CO_3 (1.2 equiv)	0%	100%	0%	17%
3	$NiBr_2$.DME (2 mol%), dtbbpy (2 mol%), Cs_2CO_3 (1.2 equiv)	0%	67%	0%	13%
4	$NiBr_2$.DME (2 mol%), dtbbpy (2 mol%), Phthalimide (1 equiv), Cs_2CO_3 (1.2 equiv)	0%	93%	0%	7%
5	$NiBr_2$.DME (0.5 mol%), dtbbpy (0.5 mol%), Phthalimide (1 equiv)	81%	7%	7%	0%
6	$NiBr_2$.DME (2 mol%), dtbppy (2 mol%), Phthalimide (1 equiv)	56%	8%	6%	1%
7	$NiBr_2$.DME (5 mol%), dtbbpy (5 mol%), Phthalimide (1 equiv)	65%	17%	9%	5%
8	$NiBr_2$.DME (10 mol%), dtbbpy (10 mol%), Phthalimide (1 equiv)	49%	22%	29%	0%

Figure S3: Additive Screening

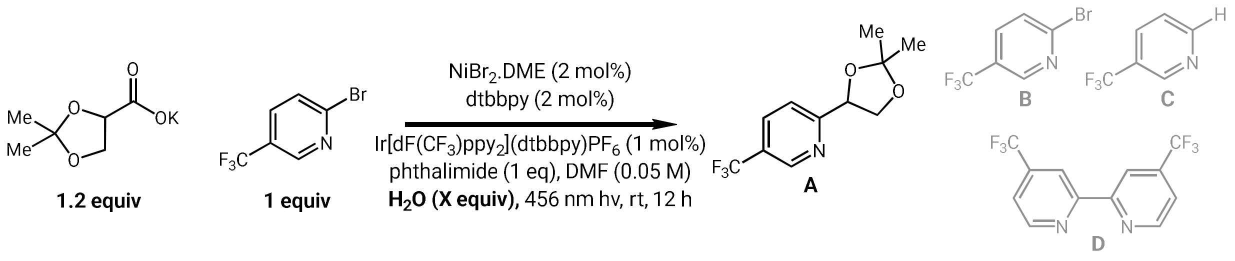

Entry	Reaction Conditions	Yield			
		A	B	C	D
1	No light	0%	100%	0%	0%
2	No [Ir(dF(CF$_3$))$_2$dtbbpy]PF$_6$	0%	96%	0%	0%
3	Under air atmosphere	0%	20%	2%	0%
4	10 equiv H_2O	17%	6%	14%	0%
5	25 equiv H_2O	12%	5%	14%	0%
6	50 equiv H_2O	2%	3%	5%	1%

Figure S4: Control Experiments and Water Additive Screening

Substitution Optimization

Procedure for Optimization: To an 8 mL vial equipped with a stir bar was added respective diol, tin catalyst, *p*-toluenesulfonyl chloride, and DCM. The vial was capped and stirred until TLC indicated complete consumption of starting material. The reaction mixture was washed with water, extracted three times with DCM (20 mL), dried with sodium sulfate, filtered, and evaporated under reduced pressure. To the crude residue, respective amine, solvent, and base were added. The reactions were heated for 16 hours. 1,3,5-Trimethoxybenzene (internal standard, 0.5 mmol) is then added to the reaction mixture. The mixture is stirred for another 15 min, then evaporated under reduced pressure, and the reaction yield was determined from ^{1}H NMR.

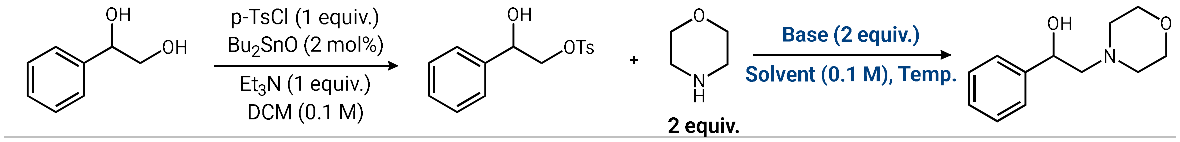

Entry	Reaction Conditions	Yield
1	Et_3N, DCM, 40°C	0%
2	Na_2CO_3, DCM, 40°C	0%
3	DIPEA, DCM, 40°C	0%
4	DBU, DCM, 40°C	6%
5	NaOMe, DCM, 40°C	11%
6	Na_2CO_3, EtOH, 90°C	55%
7	Na_2CO_3, DMF, 90°C	53%
8	NaOMe, DMSO, 90°C	35%

Figure S5: Base Screening

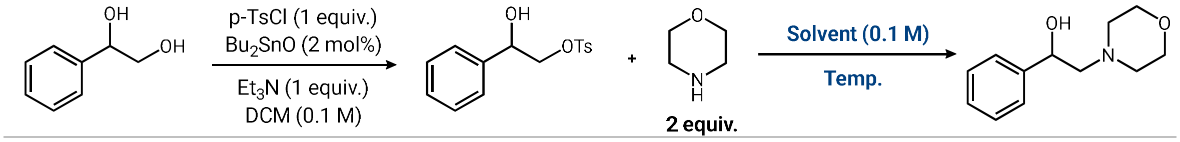

Entry	Reaction Conditions	Yield
1	THF, 60°C	43%
2	EtOH, 75°C	83%
3	DMF, 75°C	62%
4	DMSO, 75°C	33%
5	DCM, 40°C	19%

Figure S6: Solvent Screening

General Procedure for the Preparation of Starting Materials and Characterization.

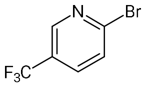

S2

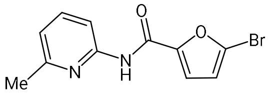

S3

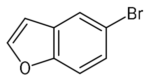

S4

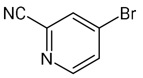

S5

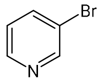

S6

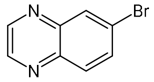

S7

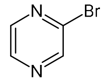

S8

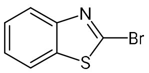

S9

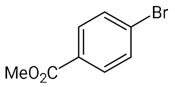

S10

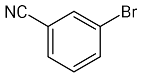

S11

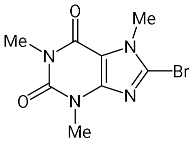

S12

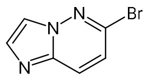

S13

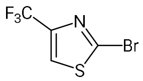

S14

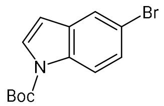

S15

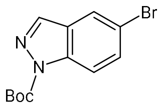

S16

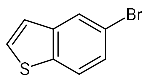

S17

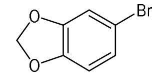

S18

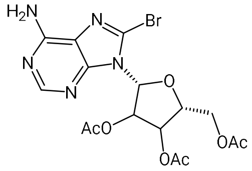

S19

Reagents **S2, S4, S5, S6, S7, S8, S9, S10, S11, S13, S14, S17,** and **S18** were purchased and used as received from Aldrich, TCI, Combi-Blocks, ChemScene or Oakwood Chemicals.

Reagents **S3, S12, S15, S16, and S19** were synthesized according to the procedures reported below.

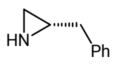
S22

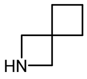
S23

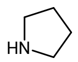
S24

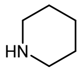
S25

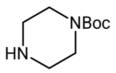
S26

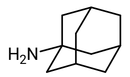
S27

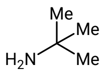
S28

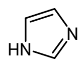
S29

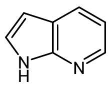
S30

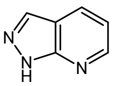
S31

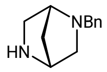
S32

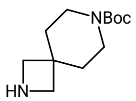
S33

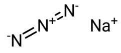
S34

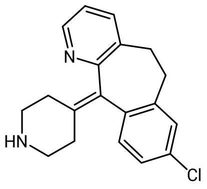
S35

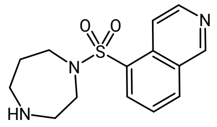
S36

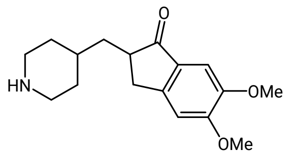
S37

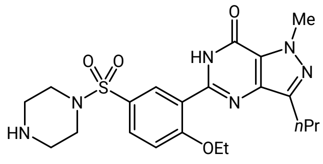
S38

Reagents **S23, S24, S25, S28, S29, S30, S31, S32, S33, S34, S35, S36, S37,** and **S38** were purchased and used as received from Aldrich, TCI, Combi-Blocks, ChemScene or Oakwood Chemicals.

Reagent **S22** was synthesized according to the procedures reported below.

5-bromo-N-(6-methylpyridin-2-yl)furan-2-carboxamide (S3)

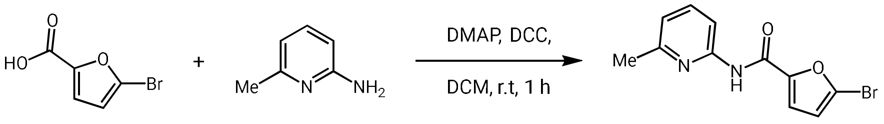

To a flame-dried flask was added 5-bromofuran-2-carboxylic acid (1.0 g, 1.0 equiv) followed by DCC (1.13 g, 1.05 equiv) and DMAP (31 mg, 5 mol %). The flask was placed under nitrogen before dry DCM was added (10 mL, 0.5 M) and the solution was stirred for 30 minutes. 6-methylpyridin-2-amine (517 mg, 1.05 equiv) was added and the resulting mixture was stirred for 1 hour. Solids were removed by vacuum filtration and washed with DCM. The filtrate was concentrated in vacuo and the crude material was purified by flash column chromatography (EtOAc/Hex = 3 :7) to afford 5-bromo-N-(6-methylpyridin-2-yl)furan-2-carboxamide as a white solid, 65% yield, 955 mg.

^{1}H NMR (500 MHz, CDCl$_3$) ***δ*** 8.62 (s, 1H), 8.09 (dt, J = 8.3, 0.7 Hz, 1H), 7.62 (dd, J = 8.2, 7.5 Hz, 1H), 7.20 (d, J = 3.5 Hz, 1H), 6.93 (d, J = 7.4 Hz, 1H), 6.50 (d, J = 3.5 Hz, 1H), 2.48 (s, 2H).

^{13}C NMR (126 MHz, CDCl$_3$) *δ* 157.20, 155.04, 150.17, 149.04, 138.90, 125.59, 119.81, 118.12, 114.80, 111.17, 24.14

8-bromo-1,3,7-trimethyl-3,7-dihydro-1H-purine-2,6-dione (S3)

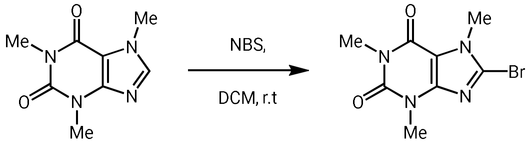

Prepared following the literature procedure,[1] to a solution of caffeine (1.0 g, 1.0 eq) in DCM (15 mL,0.35 M) was added NBS (1.83 g, 2.0 eq). Once all solids dissolved, water (5 mL, 50.0 eq) was added, and the solution was stirred at room temperature for 5 days. To the resulting yellow mixture was added 2M aq NaOH and the solution became colorless. The aqueous layer was extracted 3x with DCM. The combined organic layer was washed with brine, dried with sodium sulfate and evaporated under reduced pressure to yield 8-bromo-1,3,7-trimethyl-3,7-dihydro-1H-purine-2,6-dione as a white solid, 98% yield, 1.30 g.

^{1}H NMR (500 MHz, $CDCl_3$) δ 3.96 (s, 3H), 3.56 (s, 3H), 3.40 (s, 3H).

General Procedure for the Synthesis of Boc Protected S15, S16

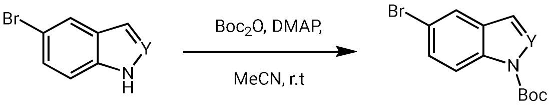

Following the procedure reported in literature,[2] to a solution of free amine (1.0 eq) in acetonitrile (0.1 M) was added 4-dimethylaminopyridine (10 mol%) followed by di-tert-butyl dicarbonate (1.0 eq). After completion of the reaction as monitored by TLC, the reaction mixture was concentrated in vacuo. The resulting oil was purified by flash column chromatography on silica gel to afford SX and SX.

tert-butyl 5-bromo-1H-indole-1-carboxylate (S15)

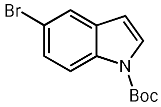

Prepared following the general procedure from 5-bromo-1H-indole (300 mg, 1.53 mmol). The crude material was purified by silica plug eluting with hexanes to afford 440 mg of SX in 97% yield, isolated as a white solid.

^{1}H NMR (500 MHz, $CDCl_3$) δ 8.02 (d, J = 8.6 Hz, 1H), 7.69 (d, J = 1.9 Hz, 1H), 7.59 (d, J = 3.8 Hz, 1H), 7.40 (dd, J = 8.8, 2.0 Hz, 1H), 6.50 (dd, J = 3.7, 0.8 Hz, 1H), 1.67 (s, 9H).

tert-butyl 5-bromo-1H-indazole-1-carboxylate (S16)

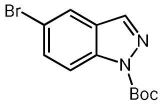

Prepared following the general procedure from 5-bromo-1H-indazole (1.0 g, 5.07 mmol). The crude material was purified by flash column chromatography (EtOAc/Hex = 3:7) to afford 1.43 g of **SX** in 95% yield, isolated as a orange oil.

^{1}H NMR (500 MHz, $CDCl_3$) *δ* 8.11 (d, J = 0.9 Hz, 1H), 8.08 (d, J = 8.9 Hz, 1H), 7.87 (d, J = 1.7 Hz, 1H), 7.61 (dd, J = 8.9, 1.9 Hz, 1H), 1.72 (s, 9H)

(2R,3R,4R,5R)-2-(acetoxymethyl)-5-(6-amino-8-bromo-9H-purin-9-yl)tetrahydrofuran-3,4-diyl diacetate (S19)

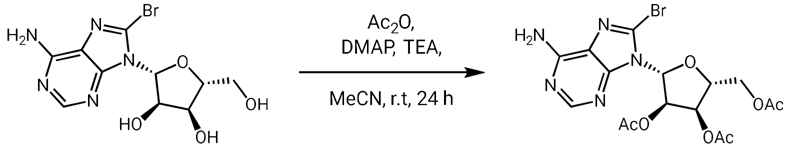

Prepared by modification of the literature procedure,[3] To a solution of 8-bromo-adenosine (1.0 g, 1 equiv) in MeCN (15 mL, 0.2 M) was added DMAP (141 mg, 40 mol %) and TEA (1.61 mL, 4.0 equiv). Acetic anhydride (1.19 g, 4.0 equiv) was added and the solution was stirred at room temperature for 24 hours. The reaction was quenched with water and the aqueous layer was extracted 3x with chloroform. The combined organic layer was washed with water and brine, dried over sodium sulfate and evaporated under reduced pressure. The solid residue was recrystallized from hot ethanol to afford (2R,3R,4R,5R)-2-(acetoxymethyl)-5-(6-amino-8-bromo-9H-purin-9-yl)tetrahydrofuran-3,4-diyl diacetate as a white solid, 93% yield, 1.26 g.

^{1}H NMR (500 MHz, CDCl3) *δ* 8.32 (s, 1H), 6.34 (dd, J = 6.0, 4.3 Hz, 1H), 6.10 (d, J = 4.3 Hz, 1H), 5.94 (t, J = 5.9 Hz, 1H), 5.79 – 5.55 (m, 2H), 4.52 (dd, J = 11.8, 3.5 Hz, 1H), 4.39 (td, J = 5.9, 3.5 Hz, 1H), 4.33 (dd, J = 11.8, 6.0 Hz, 1H), 2.16 (s, 3H), 2.11 (s, 3H), 2.05 (s, 3H).

(S)-2-benzylaziridine (S22)

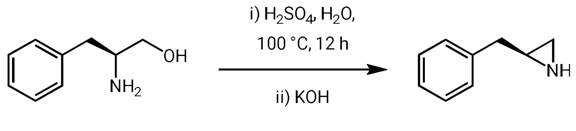

Prepared according to literature procedure,[4] to a solution of concentrated sulfuric acid (1.35 g, 1.05 eq) and water (3 mL, 4.40 M) was added (S)-(−)-2-Amino-3-phenyl-1-propanol (2.0 g, 1.0 eq). The reaction mixture was heated to reflux for 12 hours. After cooling, the solution was adjusted to pH 10 using potassium hydroxide pellets and stirred for 30 minutes. The aqueous layer was extracted 3x with DCM. The combined organic layer was washed with brine, dried with sodium sulfate and evaporated under reduced pressure to yield (S)-2-benzylaziridine as a colorless oil, 60% yield, 713 mg.

^{1}H NMR (500 MHz, CDCl$_3$) δ 7.57 – 7.06 (m, 5H), 3.64 (dd, J = 10.8, 3.7 Hz, 1H), 3.40 (dd, J = 10.8, 7.2 Hz, 1H), 3.15 (d, J = 10.0 Hz, 1H), 2.80 (d, J = 5.6 Hz, 1H), 2.64 (s, 1H), 2.57 (dd, J = 13.5, 8.4 Hz, 1H).

Tert-butyl piperazine-1-carboxylate (S26)

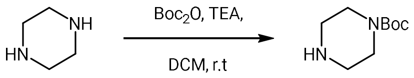

Prepared according to the literature procedure,[5] to a solution of piperazine (1.0 g, 1 equiv.) in dry DCM (10 mL) was added triethylamine (TEA) (1.62 mL, 2.0 equiv.) and di-tert-butyl decarbonate (2.53 g, 1.0 equiv) and the solution was stirred at room temperature for 2 hours. The solution was diluted with water and extracted with ethyl acetate three times. The combined organic extract was washed once with saturated sodium bicarbonate and once with brine, dried with sodium sulfate and evaporated under reduced pressure to afford Tert-butyl piperazine-1-carboxylate as a white solid, 90% yield, 1.94 g.

^{1}H NMR (500 MHz, CDCl$_3$) δ 3.54 – 3.23 (m, 4H), 2.80 (t, J = 5.1 Hz, 4H), 1.45 (s, 9H).

Preparation of Acetonide Reagents and Characterization.

potassium 2,2-dimethyl-1,3-dioxolane-4-carboxylate (1)

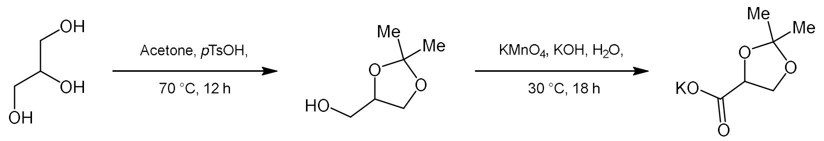

Prepared by modification of the following literature procedures,[6,7] To an oven dried round-bottom flask was added glycerol (10 g, 1.0 equiv.) followed by acetone (100 mL) then p-toluenesulfonic acid (413 mg, 0.02 equiv.) and the resulting solution was heated at reflux for 12 hours. The mixture was concentrated in vacuo, and the resulting oil was filtered through a pad of celite (3-4 cm thick) and eluted with hexanes. After removal of the hexanes in vacuo, solketal was obtained as a clear oil, 9.57 g, 67% yield, and used directly in the next step.

To a flask containing solketal (9.57g, 1 equiv.), potassium hydroxide (4.88 g, 1.20 equiv.) and water (100 mL) at 0 °C was slowly added a solution of potassium permanganate (34.30 g, 3.0 equiv.) in water (100 mL) over 5 minutes. After 30 minutes, the ice bath was removed, and the solution was stirred for 18 hours at room temperature. The resulting solution was filtered through a pad of celite (3-4 cm thick) and the solids were washed with additional water. The resulting filtrate was concentrated in vacuo, and the resulting white solid was further dried by heating at 70 °C under vacuum until dry. The final yield of potassium 2,2-dimethyl-1,3-dioxolane-4-carboxylate was 12.0 g, 90% yield. (60% yield over two steps)

^{1}H NMR (400 MHz, MeOD) *δ* 4.41 (t, J = 7.3 Hz, 1H), 4.21 (t, J = 7.5 Hz, 1H), 3.87 (dd, J = 8.0, 7.2 Hz, 1H), 1.43 (s, 3H), 1.35 (s, 3H).

potassium 3-(tert-butoxycarbonyl)-2,2-dimethyloxazolidine-4-carboxylate (39)

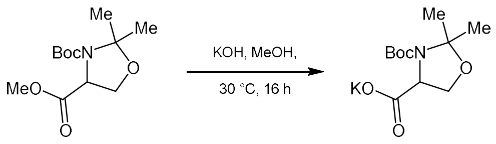

To a solution of KOH (0.41 g, 1.0 equiv.) in dry methanol (15 mL) at 0 °C, was added 3-(tert-butyl) 4-methyl 2,2-dimethyloxazolidine-3,4-dicarboxylate (2.0 g, 1.0 equiv.). After 30 minutes, the ice bath was removed and the solution was stirred for 16 hours at room temperature. The solvent was removed in vacuo to yield potassium 3-(tert-butoxycarbonyl)-2,2-dimethyloxazolidine-4-carboxylate as a white solid, 2.18 g, 100% yield.

^{1}H NMR (400 MHz, MeOD) *δ* 4.23 (dd, J = 7.0, 3.2 Hz, 1H), 4.14 (dd, J = 8.4, 7.2 Hz, 1H), 3.96 (dd, J = 8.3, 3.6 Hz, 1H), 1.64 (s, 3H), 1.49 (s, 3H), 1.44 (s, 9H).

General Procedure for Decarboxylative Arylation and Characterization of All Products (2-20)

Conditions: To an 40 mL vial (**Vial 1**), equipped with a stir bar, was added Ir[dF(CF_3)ppy]$_2$(dtbpy)PF_6 (11 mg, 0.01 mmol, 1 mol %), acetal acid (368 mg, 2.0 mmol, 2 eq.), and phthalimide (147 mg, 1.0 mmol. 1 eq.). To a separate 40 mL vial (**Vial 2**), $NiBr_2$•DME (6.1 mg, 0.02 mmol, 2 mol %) and dtbbpy (5.4 mg, 0.02 mmol, 2 mol%) were added. Both vials were added into a nitrogen-filled glovebox. 10 mL of DMF was added to each vial (0.05 M total). The liquid aryl bromides (1 mmol, 1 eq) were added to **Vial 1**. Note: if the aryl bromide is a solid, it is added to **Vial 1** before entering the glovebox. **Vial 2** was allowed to stir for 10 min, at which point the contents of **Vial 2** were added to **Vial 1**. **Vial 1** was then sealed with electrical tape, removed from the glovebox, and irradiated with two 34W Kessil PR160 456 LEDs (1 cm away, with cooling from fans to keep reactions at room temperature) for 48 hours. Afterwards, the reactions are removed from the lights. The reaction mixture is diluted with water (50 mL), and extracted three times with EtOAc (30 mL). The combined organic layers were then washed once with a saturated aqueous LiCl solution (20 mL), and once with a 1 M aqueous solution of KOH (20 mL). Afterwards, the organic layers were dried with $NaSO_4$ and evaporated under reduced pressure. 1,3,5-trimethoxybenzene (internal standard, 57 mg, 0.34 mmol) was added to obtain crude yields. Afterwards, the material was purified using flash column chromatography.

2-(2,2-dimethyl-1,3-dioxolan-4-yl)-5-(trifluoromethyl)pyridine (2)

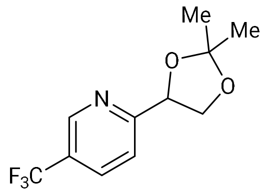

Prepared following the general procedure condition. The crude material was purified by flash column chromatography (EtOAc/Hex = 1:9) to afford 200 mg of **2** in 81% yield, isolated as a yellow oil.

^{1}H NMR (500 MHz, CDCl3) δ 8.96 – 8.67 (m, 1H), 7.95 (dd, J = 8.3, 2.3 Hz, 1H), 7.69 (dq, J = 8.2, 0.8 Hz, 1H), 5.25 (t, J = 6.6 Hz, 1H), 4.50 (dd, J = 8.5, 7.0 Hz, 1H), 3.98 (dd, J = 8.5, 6.3 Hz, 1H), 1.53 (s, 3H), 1.51 (s, 3H).

^{13}C NMR (126 MHz, CDCl3) δ 164.7, 146.1 (q, J = 4.1 Hz), 134.1 (q, J = 3.5 Hz), 125.7 (q, J = 33.1 Hz), 123.6 (q, J = 272.4 Hz), 119.9, 110.9, 77.9, 70.2, 26.6, 25.7.

^{19}F NMR (471 MHz, CDCl3) δ -62.30.

HRMS AMM (ESI-TOF) m/z calcd for $C_{11}H_{13}F_3NO_2^+$ $[M+H]^+$ 248.08984, found 248.08929

5-(2,2-dimethyl-1,3-dioxolan-4-yl)-N-(6-methylpyridin-2-yl)furan-2-carboxamide (3)

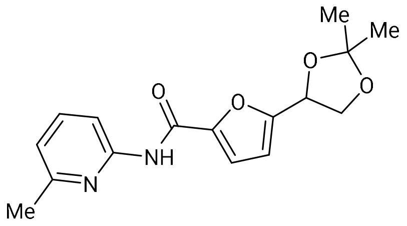

Prepared following the general procedure condition with the following changes. During workup, the organic was not treated with 1 M KOH. The crude material was purified by flash column chromatography (EtOAc/Hex = 2:8) to afford 175 mg of **3** in 58% yield, isolated as a white solid.

^{1}H NMR (500 MHz, $CDCl_3$) δ 8.63 (s, 1H), 8.11 (d, J = 8.2 Hz, 1H), 7.62 (t, J = 7.9 Hz, 1H), 7.22 (d, J = 3.5 Hz, 1H), 6.93 (d, J = 7.4 Hz, 1H), 6.51 (dd, J = 3.5, 0.6 Hz, 1H), 5.14 (t, J = 6.5 Hz, 1H), 4.32 (dd, J = 8.5, 6.5 Hz, 1H), 4.10 (dd, J = 8.4, 6.5 Hz, 1H), 2.48 (s, 3H), 1.52 (s, 3H), 1.46 (s, 3H).

^{13}C NMR (126 MHz, CDCl3) δ 157.1, 156.1, 155.7, 150.3, 147.3, 138.9, 119.7, 116.7, 111.2, 110.7, 110.3, 71.3, 68.4, 26.6, 25.8, 24.2.

HRMS AMM (ESI-TOF) m/z calcd for $C_{16}H_{19}N_2O_4^+$ $[M+H]^+$ 303.13448, found 303.13445

5-(2,2-dimethyl-1,3-dioxolan-4-yl)benzofuran (4)

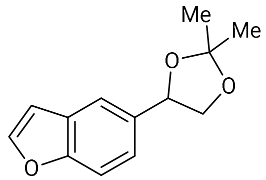

Prepared following the general procedure condition. The crude material was purified by flash column chromatography (EtOAc/Hex = 5:95) to afford 102 mg of **4** in 47% yield, isolated as a clear oil.

^{1}H NMR (500 MHz, CDCl3) *δ* 7.63 (t, J = 2.1 Hz, 1H), 7.49 (dd, J = 8.5, 0.9 Hz, 1H), 7.37 – 7.30 (m, 1H), 6.76 (dd, J = 2.2, 1.0 Hz, 1H), 5.17 (dd, J = 8.1, 6.1 Hz, 1H), 4.33 (dd, J = 8.2, 6.2 Hz, 1H), 3.74 (t, J = 8.2 Hz, 1H), 1.59 (s, 3H), 1.51 (s, 3H).

^{13}C NMR (126 MHz, CDCl3) *δ* 154.9, 145.7, 133.6, 127.7, 122.8, 119.2, 111.6, 109.8, 106.7, 78.3, 72.2, 26.8, 26.1.

HRMS AMM (ESI-TOF) m/z calcd for $C_{13}H_{15}O_3^+$ $[M+H]^+$ 219.1021, found 219.0988

4-(2,2-dimethyl-1,3-dioxolan-4-yl)picolinonitrile (5)

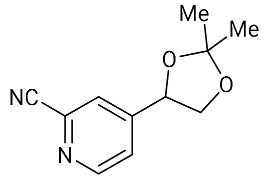

Prepared following the general procedure condition with the following modifications. The reaction was run on 0.2 mmol scale. The crude material was purified by preparatory TLC (3:1 mixture EtOAc to EtOH with 2% aq. ammonium hydroxide added/Hex = 5:95) to afford 25 mg of **5** in 60% yield, isolated as a colorless oil.

^{1}H NMR (500 MHz, CDCl3) *δ* 8.68 (dd, J = 5.1, 0.9 Hz, 1H), 7.70 (dt, J = 1.6, 0.8 Hz, 1H), 7.47 (ddd, J = 5.1, 1.7, 0.7 Hz, 1H), 5.10 (t, J = 6.9 Hz, 1H), 4.42 (dd, J = 8.4, 6.6 Hz, 1H), 3.70 (dd, J = 8.5, 7.0 Hz, 1H), 1.55 (d, J = 0.8 Hz, 3H), 1.49 (d, J = 0.8 Hz, 3H).

^{13}C NMR (126 MHz, CDCl3) *δ* 151.2, 151.0, 134.3, 125.6, 123.9, 117.1, 111.0, 75.5, 70.7, 26.3, 25.6.

HRMS AMM (ESI-TOF) m/z calcd for $C_{11}H_{13}N_2O_2^+$$[M+H]^+$ 205.09770, found 205.09691

3-(2,2-dimethyl-1,3-dioxolan-4-yl)pyridine (6)

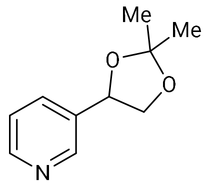

Prepared following the general procedure condition. The crude material was purified by flash column chromatography (EtOAc/Hex = 1:9) to afford 95 mg of **6** in 53% yield, isolated as a yellow oil.

^{1}H NMR (500 MHz, CDCl3) δ 8.65 – 8.37 (m, 2H), 7.83 – 7.44 (m, 1H), 7.30 (dd, J = 7.9, 4.7 Hz, 1H), 5.09 (dd, J = 7.7, 6.3 Hz, 1H), 4.34 (dd, J = 8.3, 6.3 Hz, 1H), 3.72 (dd, J = 8.3, 7.7 Hz, 1H), 1.54 (s, 3H), 1.48 (s, 3H).

^{13}C NMR (126 MHz, CDCl3) δ 149.6, 148.2, 135.0, 134.0, 123.7, 110.3, 75.8, 71.5, 26.6, 25.9.

HRMS AMM (ESI-TOF) m/z calcd for $C_{10}H_{14}NO_2^{+}[M+H]^{+}$ 180.1025, found 180.1057

6-(2,2-dimethyl-1,3-dioxolan-4-yl)quinoxaline (7)

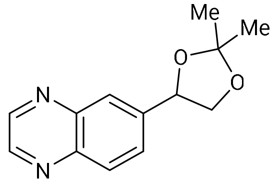

Prepared following the general procedure condition. The crude material was purified by flash column chromatography (EtOAc/Hex = 1:9) to afford 163 mg of **7** in 71% yield, isolated as a clear oil.

^{1}H NMR (500 MHz, CDCl3) δ 8.95 – 8.67 (m, 2H), 8.39 – 7.97 (m, 2H), 7.79 (dd, J = 8.7, 2.0 Hz, 1H), 5.32 (t, J = 7.0 Hz, 1H), 4.44 (dd, J = 8.3, 6.4 Hz, 1H), 4.09 – 3.55 (m, 1H), 1.61 (s, 3H), 1.53 (s, 3H).

^{13}C NMR (126 MHz, CDCl3) δ 145.4, 145.1, 143.0, 142.8, 142.0, 130.0, 128.2, 126.6, 110.5, 77.5, 71.6, 26.7, 26.0.

HRMS AMM (ESI-TOF) m/z calcd for $C_{13}H_{15}N2O_2^{+}[M+H]^{+}$ 231.11335, found 231.11336

2-(2,2-dimethyl-1,3-dioxolan-4-yl)pyrazine (8)

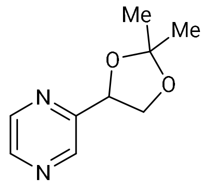

Prepared following the general procedure condition. The crude material was purified by flash column chromatography (EtOAc/Hex = 2:8) to afford 53 mg of **8** in 30% yield, isolated as a yellow oil.

^{1}H NMR (500 MHz, CDCl3) δ 8.80 (d, J = 1.6 Hz, 1H), 8.50 (d, J = 2.6 Hz, 1H), 8.49 (dd, J = 2.5, 1.4 Hz, 1H), 5.21 (t, J = 6.6 Hz, 1H), 4.46 (dd, J = 8.5, 6.8 Hz, 1H), 4.02 (dd, J = 8.5, 6.3 Hz, 1H), 1.53 (s, 3H), 1.49 (s, 3H).

^{13}C NMR (126 MHz, CDCl3) δ 155.6, 144.0, 143.7, 142.9, 110.9, 76.7, 70.0, 26.5, 25.6.

HRMS AMM (ESI-TOF) m/z calcd for $C_9H_{13}N_2O_2^+$[M+H]$^+$ 181.09770, found 181.09685

2-(2,2-dimethyl-1,3-dioxolan-4-yl)benzo[d]thiazole (9)

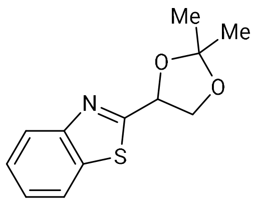

Prepared following the general procedure condition. The crude material was purified by flash column chromatography (EtOAc/Hex = 5:95) to afford 143 mg of **9** in 61% yield, isolated as a white solid.

^{1}H NMR (500 MHz, CDCl3) δ 7.97 (dt, J = 8.2, 0.9 Hz, 1H), 7.90 (dt, J = 7.9, 1.0 Hz, 1H), 7.48 (ddd, J = 8.2, 7.2, 1.2 Hz, 1H), 7.39 (ddd, J = 8.2, 7.2, 1.2 Hz, 1H), 5.48 (dd, J = 6.8, 5.4 Hz, 1H), 4.52 (dd, J = 8.7, 6.9 Hz, 1H), 4.20 (dd, J = 8.7, 5.4 Hz, 1H), 1.62 (s, 3H), 1.51 (s, 3H).

^{13}C NMR (126 MHz, CDCl3) δ 173.5, 153.6, 134.8, 126.3, 125.2, 123.1, 122.0, 111.5, 76.0, 70.3, 26.5, 25.5.

HRMS AMM (ESI-TOF) m/z calcd for $C_{12}H_{14}NO_2S^+$[M+H]$^+$ 236.07452, found 236.07368

methyl 4-(2,2-dimethyl-1,3-dioxolan-4-yl)benzoate (10)

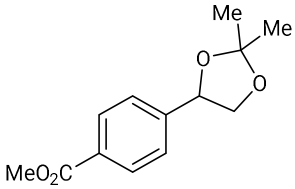

Prepared following the general procedure condition. The crude material was purified by flash column chromatography (EtOAc/Hex = 5:95) to afford 181 mg of **10** in 77% yield, isolated as a colorless oil.

^{1}H NMR (500 MHz, CDCl3) *δ* 8.02 (d, J = 8.4 Hz, 1H), 7.43 (d, J = 8.3 Hz, 1H), 5.12 (dd, J = 7.9, 6.3 Hz, 1H), 4.34 (dd, J = 8.2, 6.4 Hz, 1H), 3.91 (s, 3H), 3.68 (t, J = 8.1 Hz, 1H), 1.55 (s, 3H), 1.49 (s, 3H).

^{13}C NMR (126 MHz, CDCl3) *δ* 166.9, 144.6, 130.0, 129.9, 126.1, 110.2, 77.5, 71.6, 52.3, 26.6, 26.0.

HRMS AMM (ESI-TOF) m/z calcd for : $C_{13}H_{17}O_4^+$[M+H]$^+$ 237.11268, found 237.11249

3-(2,2-dimethyl-1,3-dioxolan-4-yl)benzonitrile (11)

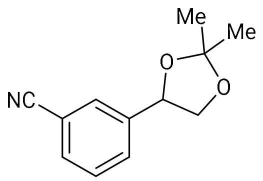

Prepared following the general procedure condition. The crude material was purified by flash column chromatography (EtOAc/Hex = 1:9) to afford 175 mg of **11** in 58% yield, isolated as a clear oil.

^{1}H NMR (500 MHz, CDCl3) *δ* 7.68 (s, 1H), 7.62 – 7.59 (m, 1H), 7.60 – 7.57 (m, 1H), 7.47 (t, J = 7.8 Hz, 1H), 5.09 (dd, J = 7.5, 6.4 Hz, 1H), 4.35 (dd, J = 8.3, 6.3 Hz, 1H), 3.67 (dd, J = 8.3, 7.6 Hz, 1H), 1.56 (s, 3H), 1.49 (s, 3H).

^{13}C NMR (126 MHz, CDCl3) *δ* 141.4, 131.8, 130.6, 129.9, 129.5, 118.8, 112.9, 110.5, 71.5, 29.9, 26.6, 25.9.

HRMS AMM (ESI-TOF) m/z calcd for $C_{12}H_{14}NO_2^+$[M+H]$^+$204.10245, found 204.10182

8-(2,2-dimethyl-1,3-dioxolan-4-yl)-1,3,7-trimethyl-3,7-dihydro-1H-purine-2,6-dione (12)

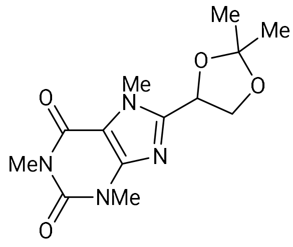

Prepared following the general procedure condition. The crude material was purified by flash column chromatography (EtOAc/Hex = 2:8) to afford 135 mg of **12** in 46% yield, isolated as a white solid.

^{1}H NMR (500 MHz, CDCl3) δ 5.18 (t, J = 6.6 Hz, 1H), 4.62 (dd, J = 8.5, 6.4 Hz, 1H), 4.36 (dd, J = 8.5, 6.8 Hz, 1H), 4.06 (s, 3H), 3.55 (s, 3H), 3.39 (s, 3H), 1.46 (s, 3H), 1.42 (s, 3H).

^{13}C NMR (126 MHz, CDCl3) δ 155.6, 151.8, 149.9, 147.5, 111.2, 108.8, 70.0, 67.4, 32.6, 29.9, 28.1, 26.2, 25.6.

HRMS AMM (ESI-TOF) m/z calcd for $C_{13}H_{19}N_4O_4^+[M+H]^+$ 295.14063, found 295.13971

6-(2,2-dimethyl-1,3-dioxolan-4-yl)imidazo[1,2-b]pyridazine (13)

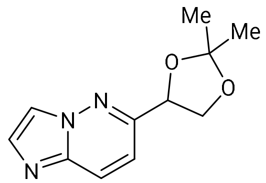

Prepared following the general procedure condition. The crude material was purified by flash column chromatography (EtOAc/Hex = 3:7) to afford 81 mg of **13** in 37% yield, isolated as a clear oil.

^{1}H NMR (500 MHz, CDCl3) δ 8.32 (d, J = 4.6 Hz, 1H), 7.98 (d, J = 1.3 Hz, 1H), 7.72 (d, J = 1.3 Hz, 1H), 7.21 (dd, J = 4.6, 1.3 Hz, 1H), 5.70 (ddd, J = 7.5, 6.5, 1.3 Hz, 1H), 4.75 (dd, J = 8.6, 7.2 Hz, 1H), 3.93 (dd, J = 8.6, 6.5 Hz, 1H), 1.57 (s, 3H), 1.54 (s, 3H).

^{13}C NMR (126 MHz, CDCl3) δ 143.5, 139.5, 137.5, 133.2, 117.1, 112.0, 110.4, 72.7, 70.0, 26.5, 25.5.

HRMS AMM (ESI-TOF) m/z calcd for : $C_{11}H_{14}N_3O_2^+[M+H]^+$220.10860, found 220.10781

2-(2,2-dimethyl-1,3-dioxolan-4-yl)-4-(trifluoromethyl)thiazole (14)

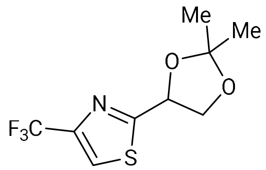

Prepared following the general procedure condition. The crude material was purified by flash column chromatography (EtOAc/Hex = 5:95) to afford 103 mg of **14** in 41% yield, isolated as a clear oil.

^{1}H NMR (500 MHz, CDCl3) δ 7.72 (d, J = 0.9 Hz, 1H), 5.38 (dd, J = 6.8, 4.9 Hz, 1H), 4.47 (dd, J = 8.9, 6.7 Hz, 1H), 4.13 (dd, J = 8.8, 4.9 Hz, 1H), 1.59 (s, 3H), 1.47 (s, 3H).

^{13}C NMR (126 MHz, CDCl3) δ 175.42, 145.25 (q, *J* = 37.3 Hz), 121.07 (q, *J* = 3.5 Hz), 119.31, 111.54, 75.44, 70.37, 26.60, 25.24.

^{19}F NMR (471 MHz, CDCl3) δ -64.0

HRMS AMM (ESI-TOF) m/z calcd for $C_9H_{11}F_3NO_2S^+$[M+H]$^+$254.04626, found 254.04537

tert-butyl 5-(2,2-dimethyl-1,3-dioxolan-4-yl)-1H-indole-1-carboxylate (15)

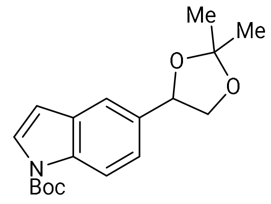

Prepared following the general procedure condition. The crude material was purified by flash column chromatography (EtOAc/Hex = 5:95) to afford 175 mg of **15** in 58% yield, isolated as a clear oil.

¹H NMR (500 MHz, CDCl3) δ 8.12 (s, 1H), 7.60 (d, J = 3.6 Hz, 1H), 7.58 (d, J = 1.7 Hz, 1H), 7.31 (dd, J = 8.6, 1.8 Hz, 1H), 6.56 (dd, J = 3.7, 0.7 Hz, 1H), 5.17 (dd, J = 8.2, 6.1 Hz, 1H), 4.32 (dd, J = 8.2, 6.1 Hz, 1H), 3.74 (t, J = 8.2 Hz, 1H), 1.67 (s, 9H), 1.59 (s, 3H), 1.51 (s, 3H).

¹³C NMR (126 MHz, CDCl3) δ 122.6, 118.8, 115.4, 109.8, 107.4, 83.9, 78.4, 72.2, 149.8, 135.1, 133.4, 130.8, 126.6, 28.3, 26.8, 26.2.

HRMS AMM (ESI-TOF) m/z calcd for $C_{18}H_{23}NNaO_4^+$[M+Na]$^+$340.15248, found 340.15177

tert-butyl 5-(2,2-dimethyl-1,3-dioxolan-4-yl)-1H-indazole-1-carboxylate (16)

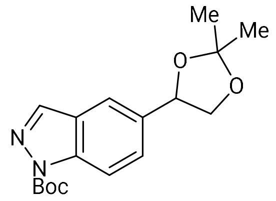

Prepared following the general procedure condition. The crude material was purified by flash column chromatography (EtOAc/Hex = 1:9) to afford 232 mg of **16** in 73% yield, isolated as a clear oil.

¹H NMR (500 MHz, CDCl3) δ 8.17 (d, J = 9.7 Hz, 2H), 7.75 (dt, J = 1.5, 0.7 Hz, 1H), 7.52 (dd, J = 8.7, 1.6 Hz, 1H), 5.20 (dd, J = 7.9, 6.2 Hz, 1H), 4.35 (dd, J = 8.3, 6.2 Hz, 1H), 3.73 (t, J = 8.1 Hz, 1H), 1.72 (s, 9H), 1.59 (s, 3H), 1.51 (s, 3H).

¹³C NMR (126 MHz, CDCl3) δ 149.2, 139.7, 139.6, 135.1, 127.4, 126.1, 118.7, 114.9, 110.1, 85.1, 77.8, 71.9, 28.3, 26.7, 26.0.

HRMS AMM (ESI-TOF) m/z calcd for $C_{17}H_{22}N_2NaO_4^+$[M+Na]$^+$341.14773, found 341.14676

4-(benzo[b]thiophen-5-yl)-2,2-dimethyl-1,3-dioxolane (17)

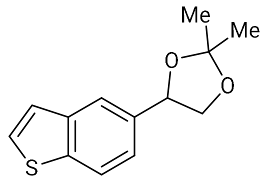

Prepared following the general procedure condition. The crude material was purified by flash column chromatography (EtOAc/Hex = 2:8) to afford 140 mg of **17** in 60% yield, isolated as a clear oil.

^{1}H NMR (500 MHz, CDCl3) δ 7.87 (d, J = 8.3 Hz, 1H), 7.85 – 7.82 (m, 1H), 7.46 (d, J = 5.4 Hz, 1H), 7.35 (dd, J = 8.3, 1.8 Hz, 1H), 7.33 (dd, J = 5.5, 0.8 Hz, 1H), 5.21 (dd, J = 8.1, 6.2 Hz, 1H), 4.36 (dd, J = 8.2, 6.2 Hz, 1H), 3.76 (t, J = 8.1 Hz, 1H), 1.60 (s, 3H), 1.53 (s, 3H).

^{13}C NMR (126 MHz, CDCl3) δ 139.9, 139.5, 135.4, 127.2, 123.9, 122.8, 122.6, 121.4, 109.9, 78.2, 72.0, 26.8, 26.1.

HRMS AMM (ESI-TOF) m/z calcd for $C_{13}H_{15}O_2S^+$ $[M+H]^+$ 235.0793, found 235.0805

5-(2,2-dimethyl-1,3-dioxolan-4-yl)benzo[d][1,3]dioxole (18)

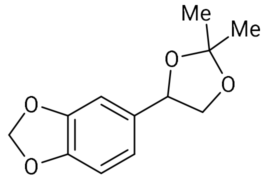

Prepared following the general procedure condition. The crude material was purified by flash column chromatography (EtOAc/Hex = 2:98) to afford 148 mg of **18** in 67% yield, isolated as a clear oil.

^{1}H NMR (500 MHz, CDCl3) δ 6.89 (d, J = 1.6 Hz, 1H), 6.81 (dd, J = 8.0, 1.7 Hz, 1H), 6.77 (d, J = 8.0 Hz, 1H), 5.95 (s, 2H), 4.98 (dd, J = 8.0, 6.1 Hz, 1H), 4.25 (dd, J = 8.3, 6.2 Hz, 1H), 3.66 (t, J = 8.1 Hz, 1H), 1.54 (s, 3H), 1.47 (s, 3H).

^{13}C NMR (126 MHz, CDCl3) δ 148.1, 147.6, 133.0, 120.1, 109.8, 108.3, 106.8, 101.2, 78.0, 71.8, 26.8, 26.1.

HRMS AMM (ESI-TOF) m/z calcd for $C_{12}H_{15}O_4^+$ $[M+H]^+$ 223.0970, found 223.0943

(2R,3R,4R,5R)-2-(6-acetamido-8-(2,2-dimethyl-1,3-dioxolan-4-yl)-9H-purin-9-yl)-5-(acetoxymethyl)tetrahydrofuran-3,4-diyl diacetate (19)

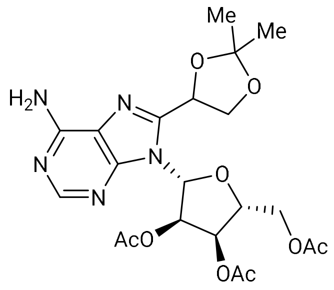

Prepared following the general procedure condition with the following changes. The reaction was run on 0.2 mmol scale. During workup, the organic was not treated with 1 M KOH. The crude material was purified by flash column chromatography (3:1 mixture EtOAc to EtOH with 2% aq. ammonium hydroxide added /Hex = 2:3) to afford 33 mg of **19** in 33% yield as a 2:1 mixture of diastereomers, isolated as a yellow oil.

^{1}H NMR (500 MHz, CDCl3):

Major Diastereomer: δ 8.33 (s, 1H), 6.44 (dd, J = 5.9, 3.9 Hz, 1H), 6.38 (d, J = 3.9 Hz, 1H), 5.99 (t, J = 5.9 Hz, 1H), 5.64 (s, 1H), 5.33 (dd, J = 6.9, 5.7 Hz, 1H), 4.75 (dd, J = 8.6, 5.7 Hz, 1H), 4.56 – 4.47 (m, 1H), 4.46 – 4.28 (m, 2H), 2.13 (s, 3H), 2.09 (s, 3H), 2.04 (s, 3H), 2.01 (s, 3H), 1.46 (s, 3H), 1.37 (s, 3H).

Minor Diastereomer: δ 8.33 (s, 1H), 6.41 (d, J = 4.6 Hz, 1H), 6.09 (dd, J = 6.3, 4.5 Hz, 1H), 5.85 (t, J = 6.1 Hz, 1H), 5.69 (s, 1H), 5.37 (dd, J = 6.9, 5.7 Hz, 1H), 4.77 (dd, J = 8.5, 5.7 Hz, 1H), 4.56 – 4.47 (m, 1H), 4.44 – 4.23 (m, 3H), 2.13 (s, 3H), 2.08 (s, 3H), 2.07 (s, 3H), 2.01 (s, 3H), 1.49 (s, 3H), 1.44 (s, 3H).

^{13}C NMR (126 MHz, CDCl3):

Major Diastereomer: δ 170.7, 169.6, 155.2, 152.7, 151.0, 149.6, 134.3, 123.6, 111.2, 87.2, 79.9, 72.4, 70.7, 70.6, 67.0, 63.2, 26.1, 25.5, 20.7, 20.6, 20.4.

Minor Diastereomer δ 169.6, 169.5, 152.7, 151.3, 149.2, 134.3, 123.6, 118.8, 111.2, 87.0, 79.6, 73.2, 71.2, 70.3, 67.1, 63.0, 26.1, 25.3, 20.8, 20.6, 20.5.

HRMS AMM (ESI-TOF) m/z calcd for $C_{21}H_{28}N_5O_9{}^+$[M+H]$^+$494.18870, found 494.18853

1-(5-(trifluoromethyl)pyridin-2-yl)ethane-1,2-diol (20)

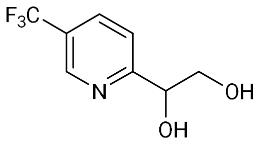

Prepared following the general procedure condition with the following changes. The crude material was purified by flash column chromatography (EtOAc/Hex = 1:9) to afford 200 mg of **2** in 81% yield, isolated as a yellow oil. Following purification, the material was subjected to acid deprotection. (1 M HCl in MeOH, 10 mL, 0.1 M). The mixture was heated to 65 °C for 3 hours. Afterwards, the reaction was quenched with saturated aqueous sodium bicarbonate (30 mL), and extracted three times with EtOAc (25 mL). Afterwards, the organic layers were combined, washed with saturated aqueous brine, and dried over $NaSO_4$. The organic layer was evaporated under reduced pressure. The material was used without any further purification to afford 168 mg of **20** in 81% yield, isolated as a yellow solid.

^{1}H NMR (500 MHz, CDCl3) δ 8.87 – 8.77 (m, 1H), 7.98 (dt, J = 8.3, 1.5 Hz, 1H), 7.55 (dt, J = 8.2, 0.8 Hz, 1H), 4.91 (t, J = 4.8 Hz, 1H), 3.99 (dd, J = 11.4, 4.0 Hz, 1H), 3.83 (dd, J = 11.4, 5.5 Hz, 1H).

^{13}C NMR (126 MHz, CDCl3) δ 163.4 (d, J = 1.7 Hz), 145.6 (q, J = 4.1 Hz), 134.3 (q, J = 3.4 Hz), 126.1 (q, J = 33.1 Hz), 124.6, 121.0, 73.5, 66.9.

^{19}F NMR (471 MHz, CDCl3) δ -62.33.

HRMS AMM (ESI-TOF) m/z calcd for $C_8H_9F_3NO_2^+$[M+H]$^+$ 208.05854, found 208.05780

General Procedure for Substitution Reaction and Characterization of All Products (21-38)

Condition A: To an 8 mL vial equipped with a stir bar was added alcohol (42 mg, 0.2 mmol, 1 eq.), Bu_2SnO (1 mg, 0.004mmol, 2 mol %), p-TsCl (38mg, 0.2 mmol, 1 eq.), Et_3N (29 µL,0.2 mmol, 1 eq.) and DCM (2 mL, 0.1M). The vial was capped and stirred until TLC indicated complete consumption of starting material. The reaction mixture was washed with water and extracted with DCM (20 mL) 3 times. The organic layers were collected, dried with sodium sulfate, filtered, and evaporated under reduced pressure and then transferred to a 20 mL vial equipped with a stir bar. The amine (0.4 mmol, 2 eq.) was added, followed by EtOH (2 mL, 0.1 M), and the reaction mixture was stirred at 75°C for 16 hours. The mixture is stirred for another 15 min, then evaporated under reduced pressure, and the crude material was purified by flash chromatography.

Condition B: To an 8 mL vial equipped with a stir bar was added alcohol (42 mg, 0.2 mmol, 1 eq.), Bu_2SnO (1 mg, 0.004mmol, 2 mol %), p-TsCl (38mg, 0.2 mmol, 1 eq.), Et_3N (29 µL,0.2 mmol, 1 eq.) and DCM (2 mL, 0.1M). The vial was capped and stirred until TLC indicated complete consumption of starting material. The reaction mixture was washed with water and extracted with DCM (20 mL) 3 times. The organic layers were collected, dried with sodium sulfate, filtered, and evaporated under reduced pressure and then transferred to a 20 mL vial equipped with a stir bar. The amine (0.2 mmol, 1 eq.) was added, followed by Cs_2CO_3 (196 mg, 0.6 mmol, 3 eq.) and DMSO (2 mL, 0.1M), and the reaction mixture was stirred at 60°C for 16 hours. 1,3,5-Trimethoxybenzene (internal standard,14 mg, 0.2 mmol) is then added to the reaction mixture and stirred for another 15 min. The crude reaction mixture was washed with water and extracted with EtOAc. The organic layers were collected, dried with sodium sulfate, filtered, and evaporated under reduced pressure. The crude material was purified by flash chromatography.

Condition C: To an 8 mL vial equipped with a stir bar was added alcohol (42 mg, 0.2 mmol, 1 eq.), Bu_2SnO (1 mg, 0.004mmol, 2 mol %), p-TsCl (38mg, 0.2 mmol, 1 eq.), Et_3N (29 µL,0.2 mmol, 1 eq.) and DCM (2 mL, 0.1M). The vial was capped and stirred until TLC indicated complete consumption of starting material. The reaction mixture was washed with water and extracted with DCM (20 mL) 3 times. The organic layers were collected, dried with sodium sulfate, filtered, and evaporated under reduced pressure and then transferred to a 4 mL vial equipped with a stir bar. Sodium azide (0.6 mmol, 3 eq.) was added, followed by DMF (167 µL, 1.2 M), and the reaction mixture was stirred at 90°C for 16 hours. The crude reaction mixture was washed with water and extracted with EtOAc. The organic layers were collected, dried with sodium sulfate, filtered, and evaporated under reduced pressure. The crude material was purified by flash chromatography.

2-morpholino-1-(5-(trifluoromethyl)pyridin-2-yl)ethan-1-ol (21)

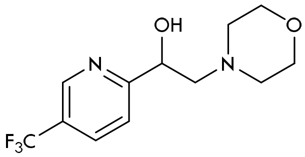

Prepared following the general procedure condition A. The crude material was purified by flash column chromatography (EtOAc/Hex = 90:100) to afford 45 mg of **21** in 82% yield, isolated as a yellow solid.

^{1}H NMR (500 MHz, CDCl3) δ 8.78 (dt, J = 2.0, 0.9 Hz, 1H), 7.94 (dd, J = 8.3, 2.3 Hz, 1H), 7.72 (d, J = 8.3 Hz, 1H), 4.89 (dd, J = 10.1, 3.9 Hz, 1H), 3.75 (m, 5H), 2.85 (dd, J = 12.5, 3.9 Hz, 1H), 2.79 – 2.67 (m, 2H), 2.50 (dd, J = 12.5, 10.0 Hz, 3H).

^{13}C NMR (126 MHz, CDCl3) δ δ 165.8, 145.8 (q, J = 4.1 Hz), 134.1 (q, J = 3.5 Hz), 125.5 (q, J = 33.0 Hz), 123.7 (q, J = 272.2 Hz), 120.2, 69.3, 67.1, 64.7, 53.6.

^{19}F NMR (471 MHz, CDCl3) δ -62.3.

HRMS AMM (ESI-TOF) m/z calcd for $C_{12}H_{16}F_3N_2O_2^+[M+H]^+$ 277.1164, found 277.1155

2-((S)-2-benzylaziridin-1-yl)-1-(5-(trifluoromethyl)pyridin-2-yl)ethan-1-ol (22)

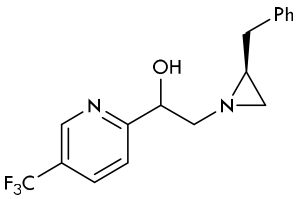

Prepared following the general procedure condition A. The crude material was purified by flash column chromatography (5-20% MeOH/DCM with 1% v/v conc. aq. ammonium hydroxide in MeOH) to afford 42 mg of **22** in 65% yield isolated as a 1:1 mixture of diastereomers as a yellow oil.

^{1}H NMR (500 MHz, CDCl3) *δ* 8.67 (d, J = 2.4 Hz, 1H), 8.64 (d, J = 2.2 Hz, 1H), 7.87 (ddd, J = 7.8, 4.9, 2.3 Hz, 2H), 7.70 (d, J = 7.9 Hz, 1H), 7.61 (d, J = 8.1 Hz, 1H), 7.56 (d, J = 8.2 Hz, 1H), 7.25 – 7.18 (m, 3H), 7.16 – 7.06 (m, 3H), 4.99 (td, J = 7.5, 3.8 Hz, 2H), 3.69 (dd, J = 11.4, 3.5 Hz, 2H), 3.48 (td, J = 11.4, 6.1 Hz, 2H), 3.34 – 3.20 (m, 2H), 3.06 (ddd, J = 7.3, 5.4, 2.3 Hz, 2H), 3.03 – 2.95 (m, 2H), 2.85 (ddd, J = 13.6, 9.2, 6.5 Hz, 2H), 2.75 (dt, J = 13.7, 6.8 Hz, 2H).

^{13}C NMR (126 MHz, CDCl3) *δ* 165.1, 165.0, 145.7 – 145.3 (m), 137.8, 134.2 (dt, *J* = 6.8, 3.5 Hz), 129.3, 129.3, 129.1, 128.8, 128.7, 126.8, 126.7, 126.0, 125.8, 125.5, 124.6, 71.6, 71.5, 62.3, 62.0, 61.1, 60.9, 52.5, 52.5, 37.1, 37.1.

^{19}F NMR (471 MHz, CDCl3) *δ* -62.3 (*diastereomer 1*), -62.3 (*diastereomer 2*).

HRMS AMM (ESI-TOF) m/z calcd for $C_{17}H_{18}F_3N_2O^+$[M+H] 323.1371, found 323.1341

2-(2-azaspiro[3.3]heptan-2-yl)-1-(5-(trifluoromethyl)pyridin-2-yl)ethan-1-ol (23)

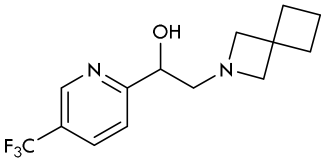

Prepared following the general procedure condition A. The crude material was purified by flash column chromatography (5-20% MeOH/DCM with 1% v/v conc. aq. ammonium hydroxide in MeOH) to afford 38 mg of **23** in 67% yield, isolated as a yellow solid.

^{1}H NMR (500 MHz, CDCl3) δ 8.77 (dd, J = 2.2, 1.1 Hz, 1H), 7.91 (dd, J = 8.3, 2.4 Hz, 1H), 7.68 (d, J = 8.2 Hz, 1H), 4.67 (dd, J = 8.5, 3.9 Hz, 1H), 3.22 (dd, J = 23.6, 6.5 Hz, 4H), 2.82 (dd, J = 11.9, 3.9 Hz, 1H), 2.64 (dd, J = 11.9, 8.5 Hz, 1H), 2.06 (t, J = 7.7 Hz, 4H), 1.84 – 1.69 (m, 2H).

^{13}C NMR (126 MHz, CDCl3) δ 166.2, 145.6 (q, J = 4.1 Hz), 133.9 (q, J = 3.5 Hz), 125.3 (q, J = 32.9 Hz), 122.6 (d, J = 2.1 Hz), 120.3, 71.2, 67.4, 65.7, 39.5, 33.1, 16.8.

^{19}F NMR (471 MHz, CDCl3) δ -62.2.

HRMS AMM (ESI-TOF) m/z calcd for $C_{14}H_{18}F_3N_2O^+$[M+H] 287.1371, found 287.1364

2-(pyrrolidin-1-yl)-1-(5-(trifluoromethyl)pyridin-2-yl)ethan-1-ol (24)

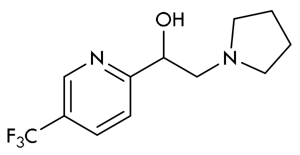

Prepared following the general procedure condition A. The crude material was purified by flash column chromatography (5-20% MeOH/DCM with 1% v/v conc. aq. ammonium hydroxide in MeOH) to afford 39 mg of **24** in 75% yield, isolated as a yellow solid.

^{1}H NMR (500 MHz, CDCl3) δ 8.79 (dt, J = 2.0, 0.9 Hz, 1H), 7.94 (dd, J = 8.3, 2.3 Hz, 1H), 7.73 (d, J = 8.3 Hz, 1H), 4.85 (dd, J = 9.5, 4.2 Hz, 1H), 2.84 (dd, J = 12.1, 4.1 Hz, 1H), 2.77 (dd, J = 12.1, 9.6 Hz, 1H), 2.74 – 2.71 (m, 2H), 2.64 – 2.50 (m, 2H), 1.86 – 1.76 (m, 4H).

^{13}C NMR (126 MHz, CDCl3) δ 166.3, 145.7 (q, J = 4.1 Hz), 134.0 (q, J = 3.4 Hz), 125.4 (q, J = 32.9 Hz), 123.8 (q, J = 272.1 Hz), 120.2, 71.1, 62.1, 54.1, 23.9.

^{19}F NMR (471 MHz, CDCl3) δ -62.2.

HRMS AMM (ESI-TOF) m/z calcd for $C_{12}H_{16}F_3N_2O^+[M+H]^+$ 261.12147, found 261.12078

2-(piperidin-1-yl)-1-(5-(trifluoromethyl)pyridin-2-yl)ethan-1-ol (25)

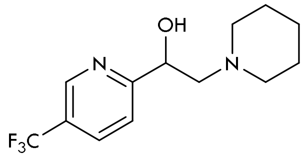

Prepared following the general procedure condition A. The crude material was purified by flash column chromatography (1-5% MeOH/DCM with 1% v/v conc. aq. ammonium hydroxide in MeOH) to afford 40 mg of **25** in 73% yield, isolated as a yellow solid.

^{1}H NMR (500 MHz, CDCl3) δ 8.77 (d, J = 2.3 Hz, 1H), 7.93 (dd, J = 8.3, 2.3 Hz, 1H), 7.73 (d, J = 8.3 Hz, 1H), 4.85 (dd, J = 10.1, 4.1 Hz, 1H), 2.79 (dd, J = 12.4, 4.0 Hz, 1H), 2.72 – 2.61 (m, 2H), 2.40 (dd, J = 12.4, 10.1 Hz, 3H), 1.68 – 1.53 (m, 5H), 1.47 (m, 2H).

^{13}C NMR (126 MHz, CDCl3) δ 166.7, 145.7 (q, J = 4.1 Hz), 134.0 (q, J = 3.5 Hz), 125.3 (q, J = 32.9 Hz), 123.8 (q, J = 272.1 Hz), 120.1, 69.4, 64.8, 54.6, 26.2, 24.3.

^{19}F NMR (471 MHz, CDCl3) δ -62.2.

HRMS AMM (ESI-TOF) m/z calcd for $C_{13}H_{18}F_3N_2O^+[M+H]^+$ 275.1371, found 275.1362

tert-butyl 4-(2-hydroxy-2-(5-(trifluoromethyl)pyridin-2-yl)ethyl)piperazine-1-carboxylate (26)

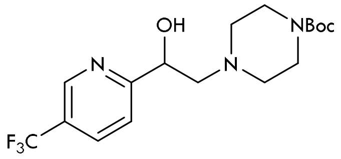

Prepared following the general procedure condition A. The crude material was purified by flash column chromatography (EtOAc/Hex = 40:60) to afford 39 mg of **26** in 72% yield, isolated as a yellow solid.

^{1}H NMR (500 MHz, CDCl3) δ 8.76 (dd, J = 2.2, 1.1 Hz, 1H), 7.93 (dd, J = 8.3, 2.3 Hz, 1H), 7.71 (d, J = 8.2 Hz, 1H), 4.88 (dd, J = 10.0, 3.9 Hz, 1H), 3.45 (m, 4H), 2.82 (dd, J = 12.6, 3.9 Hz, 1H), 2.67 (m, 2H), 2.50 (dd, J = 12.6, 10.0 Hz, 1H), 2.41 (m, 2H), 1.44 (s, 9H).

^{13}C NMR (126 MHz, CDCl3) δ 165.8, 154.8, 145.8 (q, J = 4.1 Hz), 134.1 (q, J = 3.5 Hz), 125.5 (q, J = 33.0 Hz), 123.6 (q, J = 272.1 Hz), 120.2, 80.0, 69.5, 64.3, 53.0, 28.5.

^{19}F NMR (471 MHz, CDCl3) δ -62.3.

HRMS AMM (ESI-TOF) m/z calcd for $C_{17}H_{25}F_3N_3O_3{}^+[M+H]^+$ 376.1843, found 376.1848

2-(((3s,5s,7s)-adamantan-1-yl)amino)-1-(5-(trifluoromethyl)pyridin-2-yl)ethan-1-ol (27)

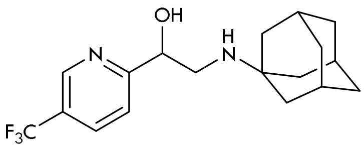

Prepared following the general procedure condition A. The crude material was purified by flash column chromatography (1-5% MeOH/DCM with 1% v/v conc. aq. ammonium hydroxide in MeOH) to afford 48 mg of **27** in 71% yield, isolated as a yellow solid.

^{1}H NMR (500 MHz, CDCl3) δ 8.76 (s, 1H), 7.92 (dd, J = 8.3, 2.3 Hz, 1H), 7.73 (d, J = 8.2 Hz, 1H), 5.29 – 5.11 (m, 2H), 4.99 (dd, J = 8.7, 3.7 Hz, 1H), 3.33 (dd, J = 12.1, 3.7 Hz, 1H), 2.85 (dd, J = 12.0, 8.7 Hz, 1H), 2.09 (s, 3H), 1.81 – 1.71 (m, 6H), 1.69 – 1.55 (m, 6H).

^{13}C NMR (126 MHz, CDCl3) δ 165.7, 145.7 (q, J = 4.0 Hz), 134.0 (q, J = 3.5 Hz), 129.0, 125.5 (q, J = 33.0 Hz), 123.7 (q, J = 272.2 Hz), 120.6, 71.1, 53.5, 46.2, 41.3, 36.3, 29.4.

^{19}F NMR (471 MHz, CDCl3) δ -62.3.

HRMS AMM (ESI-TOF) m/z calcd for $C_{18}H_{24}F_{3}N_{2}O^{+}[M+H]^{+}$ 341.1835, found 341.1844

2-(tert-butylamino)-1-(5-(trifluoromethyl)pyridin-2-yl)ethan-1-ol (28)

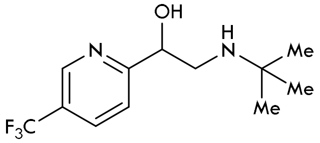

Prepared following the general procedure condition A. The crude material was purified by flash column chromatography (5-20% MeOH/DCM with 1% v/v conc. Et_3N in MeOH) to afford 48 mg of **28** in 89% yield, isolated as a yellow solid.

^{1}H NMR (500 MHz, CDCl3) δ 8.76 (dt, J = 2.0, 1.0 Hz, 1H), 7.92 (dd, J = 8.3, 2.3 Hz, 1H), 7.69 (d, J = 8.2 Hz, 1H), 4.86 (dd, J = 8.4, 3.9 Hz, 1H), 3.95 (s, 3H), 3.14 (dd, J = 11.9, 3.9 Hz, 1H), 2.74 (dd, J = 11.9, 8.3 Hz, 1H), 1.12 (s, 9H).

^{13}C NMR (126 MHz, CDCl3) δ 166.1, 145.7 (q, J = 4.1 Hz), 134.0 (q, J = 3.5 Hz), 125.4 (q, J = 33.1 Hz), 123.7 (q, J = 272.3 Hz), 120.4, 71.9, 51.5, 48.5, 28.8.

^{19}F NMR (471 MHz, CDCl3) δ -62.3.

HRMS AMM (ESI-TOF) m/z calcd for $C_{12}H_{18}F_3N_2O^+$ $[M+H]^+$ 263.1366, found 263.1369

2-(1H-imidazol-1-yl)-1-(5-(trifluoromethyl)pyridin-2-yl)ethan-1-ol (29)

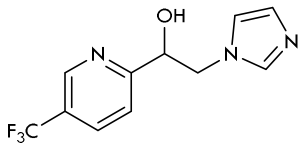

Prepared following the general procedure condition B. The crude material was purified by flash column chromatography (1-10% MeOH/DCM with 1% v/v conc. Et_3N in MeOH) to afford 22 mg of **29** in 49% yield, isolated as a yellow solid.

^{1}H NMR (500 MHz, CDCl3) δ 8.98 (d, J = 2.2 Hz, 1H), 8.10 (dd, J = 8.2, 2.3 Hz, 1H), 7.71 (d, J = 8.2 Hz, 1H), 7.59 (s, 1H), 7.12 (s, 1H), 7.07 (s, 1H), 5.25 (dd, J = 7.0, 3.7 Hz, 1H), 4.61 (dd, J = 14.1, 3.7 Hz, 1H), 4.36 (dd, J = 14.2, 7.0 Hz, 1H).

^{13}C NMR (126 MHz, CDCl3) δ 164.0, 145.9 (d, J = 4.2 Hz), 133.9 (d, J = 3.5 Hz), 132.7, 130.8, 120.56, 117.3, 115.9, 73.8, 54.4, 29.8.

^{19}F NMR (471 MHz, CDCl3) δ -62.3.

HRMS AMM (ESI-TOF) m/z calcd for $C_{11}H_{11}F_3N_3O^+[M+H]^+$ 258.0849, found 258.0845

2-(1H-pyrrolo[2,3-b]pyridin-1-yl)-1-(5-(trifluoromethyl)pyridin-2-yl)ethan-1-ol (30)

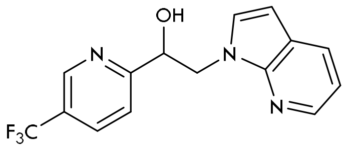

Prepared following the general procedure condition B. The crude material was purified by flash column chromatography (1-10% MeOH/DCM with 1% v/v conc. Et_3N in MeOH) to afford 26 mg of **30** in 43% yield, isolated as a yellow solid.

^{1}H NMR (500 MHz, CDCl3) δ 8.80 (dt, J = 2.0, 0.9 Hz, 1H), 8.27 (dd, J = 4.8, 1.5 Hz, 1H), 7.91 (dd, J = 7.9, 1.5 Hz, 1H), 7.82 (dd, J = 8.3, 2.3 Hz, 1H), 7.62 (dq, J = 8.2, 0.8 Hz, 1H), 7.09 (dd, J = 7.8, 4.8 Hz, 1H), 7.07 (d, J = 3.5 Hz, 1H), 6.36 (d, J = 3.5 Hz, 1H), 5.31 (dd, J = 6.3, 2.2 Hz, 1H), 4.84 (dd, J = 14.5, 2.3 Hz, 1H), 4.68 (dd, J = 14.5, 6.3 Hz, 1H).

^{13}C NMR (126 MHz, CDCl3) δ 165.2, 147.9, 145.6 (d, J = 4.1 Hz), 142.1, 133.9 (d, J = 3.6 Hz), 130.3, 129.8, 125.5, 125.2, 121.4, 120.6, 116.2, 99.9, 74.8, 53.7.

^{19}F NMR (471 MHz, CDCl3) δ -62.2.

HRMS AMM (ESI-TOF) m/z calcd for $C_{15}H_{13}F_3N_3O^+[M+H]^+$ 308.1005, found 308.1005

2-(1H-pyrazolo[3,4-b]pyridin-1-yl)-1-(5-(trifluoromethyl)pyridin-2-yl)ethan-1-ol (31)

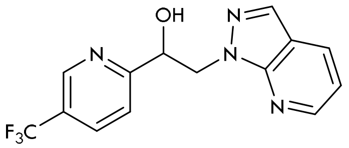

Prepared following the general procedure condition B. The crude material was purified by flash column chromatography (1-10% MeOH/DCM with 1% v/v conc. Et_3N in MeOH) to afford 22 mg of **31** in 43% yield, isolated as a yellow solid.

^{1}H NMR (500 MHz, CDCl3) *δ* 8.83 (s, 1H), 8.52 (dd, *J* = 4.6, 1.5 Hz, 1H), 8.09 (dd, *J* = 8.1, 1.5 Hz, 1H), 8.02 (s, 1H), 7.84 (dd, *J* = 8.2, 2.2 Hz, 1H), 7.56 (d, *J* = 8.3 Hz, 1H), 7.16 (dd, *J* = 8.1, 4.6 Hz, 1H), 5.40 (dd, *J* = 7.2, 3.0 Hz, 1H), 5.11 (dd, *J* = 14.3, 3.1 Hz, 1H), 4.87 (dd, *J* = 14.3, 7.2 Hz, 1H).

^{13}C NMR (126 MHz, CDCl3) *δ* 164.0, 150.6, 148.8, 145.9 (q, J = 4.2 Hz), 133.9 (q, J = 3.5 Hz), 132.7, 130.8, 125.8, 120.6, 117.3, 115.9, 73.8, 54.4, 29.8.

^{19}F NMR (471 MHz, CDCl3) *δ* -62.3.

HRMS AMM (ESI-TOF) m/z calcd for $C_{14}H_{12}F_3N_4O^+[M+H]^+$ 309.0958, found 309.0958

2-((1R,4R)-5-benzyl-2,5-diazabicyclo[2.2.1]heptan-2-yl)-1-(5-(trifluoromethyl)pyridin-2-yl)ethan-1-ol (32)

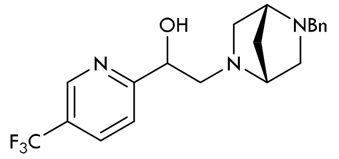

Prepared following the general procedure condition A. The crude material was purified by flash column chromatography (1-10% MeOH/DCM with 1% v/v conc. Et_3N in MeOH) to afford 48 mg of **32** in 67% yield isolated as a 1:1 mixture of diastereomers as a yellow solid.

^{1}H NMR (500 MHz, CDCl3) δ 8.75 (dd, J = 2.3, 1.3 Hz, 2H), 7.89 (ddd, J = 8.0, 5.3, 2.3 Hz, 2H), 7.70 (dd, J = 8.3, 5.0 Hz, 2H), 7.32 – 7.24 (m, 6H), 7.19 (t, J = 7.8 Hz, 4H), 4.68 (ddd, J = 14.7, 9.0, 4.2 Hz, 2H), 3.66 (qd, J = 13.4, 8.6 Hz, 4H), 3.38 – 3.23 (m, 2H), 3.11 (dd, J = 12.0, 4.0 Hz, 2H), 2.98 (dd, J = 12.2, 4.4 Hz, 2H), 2.91 – 2.74 (m, 4H), 2.76 – 2.62 (m, 6H), 2.58 – 2.42 (m, 2H), 1.83 – 1.53 (m, 4H).

^{13}C NMR (126 MHz, CDCl3) δ 166.6, 166.3, 145.7, 145.7, 145.7, 139.8, 139.7, 134.0, 133.9, 133.9, 133.9, 128.6, 128.5, 128.4, 128.4, 127.0, 126.9, 125.5, 125.4, 125.2, 125.1, 124.8, 122.6, 120.2, 120.1, 71.3, 71.0, 63.7, 63.2, 61.8, 61.5, 61.5, 61.2, 58.5, 58.4, 58.0, 57.3, 56.8, 56.7, 34.5, 33.8.

^{19}F NMR (471 MHz, CDCl3) δ -62.2 (*diastereomer 1*), -62.2 (*diastereomer 2*).

HRMS AMM (ESI-TOF) m/z calcd for $C_{20}H_{23}F_3N_3O^+$ $[M+H]^+$ 378.1788, found 378.1785

tert-butyl 2-(2-hydroxy-2-(5-(trifluoromethyl)pyridin-2-yl)ethyl)-2,7-diazaspiro[3.5]nonane-7-carboxylate (33)

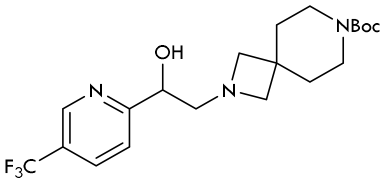

Prepared following the general procedure condition A. The crude material was purified by flash column chromatography (1-10% MeOH/DCM with 1% v/v conc. Et_3N in MeOH) to afford 48 mg of **33** in 58% yield, isolated as a yellow solid.

^{1}H NMR (500 MHz, CDCl3) δ 8.78 (dt, J = 1.8, 0.9 Hz, 1H), 7.93 (dd, J = 8.4, 2.3 Hz, 1H), 7.68 (dd, J = 8.3, 0.9 Hz, 1H), 4.68 (dd, J = 8.5, 4.1 Hz, 1H), 3.37 – 3.25 (m, 4H), 3.12 (d, J = 6.9 Hz, 2H), 3.07 (d, J = 6.9 Hz, 2H), 2.92 (dd, J = 11.9, 4.1 Hz, 1H), 2.72 (dd, J = 12.0, 8.5 Hz, 1H), 1.69 (dd, J = 6.8, 4.6 Hz, 4H), 1.44 (s, 9H).

^{13}C NMR (126 MHz, CDCl3) δ 165.8, 155.0, 145.7 (q, J = 4.2 Hz), 134.0 (q, J = 3.6 Hz), 126.15 – 124.30 (m), 122.6, 122.4, 120.3, 79.6, 71.0, 65.4, 64.5, 35.9, 35.1, 28.6.

^{19}F NMR (471 MHz, CDCl3) δ -62.2.

HRMS AMM (ESI-TOF) m/z calcd for $C_{20}H_{29}F_3N_3O_3^+$[M+H]$^+$ 416.2156, found 416.2150

2-azido-1-(5-(trifluoromethyl)pyridin-2-yl)ethan-1-ol (34)

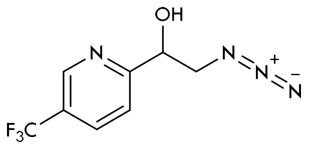

Prepared following the general procedure condition A with the following changes . Used 3 equivalents of sodium azide in 0.5 M DMF. The crude material was purified by flash column chromatography (EtOAc/Hex = 30:70) to afford 29 mg of **34** in 62% yield, isolated as a clear oil.

^{1}H NMR (500 MHz, CDCl3) δ 9.00 – 8.73 (m, 1H), 7.98 (dd, J = 8.3, 2.3 Hz, 1H), 7.54 (d, J = 8.2 Hz, 1H), 5.00 (dd, J = 6.1, 4.2 Hz, 1H), 3.69 (dd, J = 12.6, 4.1 Hz, 1H), 3.57 (dd, J = 12.6, 6.1 Hz, 1H).

^{13}C NMR (126 MHz, CDCl3) δ 162.6, 145.8 (q, J = 4.1 Hz), 134.4 (q, J = 3.4 Hz), 126.4 (q, J = 33.6 Hz), 123.5 (q, J = 272.3 Hz), 120.8, 72.6, 56.9.

^{19}F NMR (471 MHz, CDCl3) δ -62.3.

HRMS AMM (ESI-TOF) m/z calcd for $C_8H_8F_3N_4O^+[M+H]^+$ 233.0650, found 233.0641

2-(4-(8-chloro-5,6-dihydro-11H-benzo[5,6]cyclohepta[1,2-b]pyridin-11-ylidene)piperidin-1-yl)-1-(5-(trifluoromethyl)pyridin-2-yl)ethan-1-ol (35)

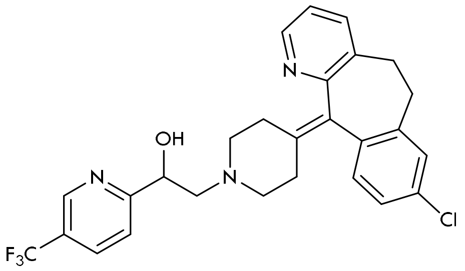

Prepared following the general procedure condition A. The crude material was purified by flash column chromatography (1-10% MeOH/DCM with 1% v/v conc. Et_3N in MeOH) to afford 67 mg of **35** in 67% yield, isolated as a yellow solid.

^{1}H NMR (500 MHz, CDCl3) δ 8.77 (dd, J = 2.0, 1.0 Hz, 1H), 8.40 (dt, J = 5.1, 1.5 Hz, 1H), 7.93 (dd, J = 8.3, 2.5 Hz, 1H), 7.74 (d, J = 8.6 Hz, 1H), 7.43 (ddd, J = 7.8, 3.5, 1.8 Hz, 1H), 7.16 (dd, J = 3.2, 1.4 Hz, 1H), 7.13 (s, 2H), 7.09 (ddd, J = 7.8, 4.8, 3.3 Hz, 1H), 4.86 (dd, J = 9.9, 4.0 Hz, 1H), 3.50 – 3.25 (m, 2H), 3.10 – 2.92 (m, 1H), 2.91 – 2.72 (m, 3H), 2.63 – 2.31 (m, 6H), 2.26 (m, 1H).

^{13}C NMR (126 MHz, CDCl3) δ 166.3, 157.5 (d, J = 7.6 Hz), 146.8, 145.7 (d, J = 4.1 Hz), 139.7 (d, J = 3.5 Hz), 138.1 (d, J = 6.3 Hz), 137.9 (d, J = 5.9 Hz), 137.5 (d, J = 6.0 Hz), 134.3 – 133.9 (m), 133.5 (d, J = 14.6 Hz), 132.9 (d, J = 2.2 Hz), 130.8 (d, J = 4.2 Hz), 129.1 (d, J = 5.4 Hz), 126.2, 125.6, 125.3, 124.8, 122.6, 122.3 (d, J = 1.6 Hz), 120.2 (d, J = 2.6 Hz), 69.6 (d, J = 1.9 Hz), 63.9 (d, J = 3.1 Hz), 55.6, 54.2 (d, J = 6.3 Hz), 46.0, 31.9 (d, J = 2.5 Hz), 31.6 (d, J = 3.6 Hz), 31.1 (dd, J = 28.5, 15.7 Hz).

^{19}F NMR (471 MHz, CDCl3) δ -62.2.

HRMS AMM (ESI-TOF) m/z calcd for $C_{27}H_{26}ClF_3N_3O^+[M+H]^+$ 500.1711, found 500.1700

2-(4-(isoquinolin-5-ylsulfonyl)-1,4-diazepan-1-yl)-1-(5-(trifluoromethyl)pyridin-2-yl)ethan-1-ol (35)

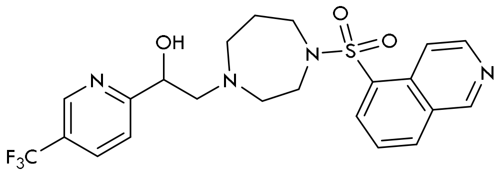

Prepared following the general procedure condition A. The crude material was purified by flash column chromatography (1-10% MeOH/DCM with 1% v/v conc. Et_3N in MeOH) to afford 57mg of **36** in 57% yield, isolated as a yellow solid.

^{1}H NMR (500 MHz, CDCl3) δ 9.34 (s, 1H), 8.75 (dd, J = 2.2, 1.1 Hz, 1H), 8.68 (d, J = 6.1 Hz, 1H), 8.41 (d, J = 6.1 Hz, 1H), 8.35 (dd, J = 7.4, 1.2 Hz, 1H), 8.19 (dt, J = 8.3, 1.1 Hz, 1H), 7.92 (dd, J = 8.3, 2.3 Hz, 1H), 7.77 – 7.64 (m, 2H), 4.77 (dd, J = 9.6, 3.9 Hz, 1H), 3.60 – 3.37 (m, 4H), 3.05 (dd, J = 12.8, 3.9 Hz, 1H), 2.99 – 2.71 (m, 4H), 2.54 (dd, J = 12.8, 9.6 Hz, 1H), 1.91 – 1.79 (m, 2H).

^{13}C NMR (126 MHz, CDCl3) δ 165.7, 153.4, 145.7 (q, J = 4.1 Hz), 145.3, 134.4, 134.0 (q, J = 3.4 Hz), 133.6, 133.3, 131.7, 129.3, 126.0, 120.2, 117.6, 70.3, 63.4, 56.5, 54.5, 48.2, 47.1, 28.7.

^{19}F NMR (471 MHz, CDCl3) δ -62.2.

HRMS AMM (ESI-TOF) m/z calcd for $C_{22}H_{23}F_3N_4O_3S^+[M+H]^+$ 480.1443, found 480.1453

2-((1-(2-hydroxy-2-(5-(trifluoromethyl)pyridin-2-yl)ethyl)piperidin-4-yl)methyl)-5,6-dimethoxy-2,3-dihydro-1H-inden-1-one (37)

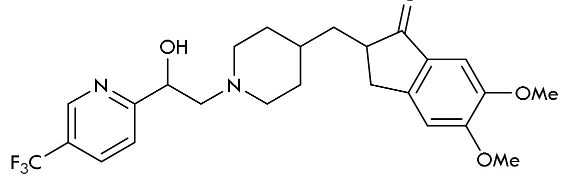

Prepared following the general procedure condition A. The crude material was purified by flash column chromatography (1-10% MeOH/DCM with 1% v/v conc. Et_3N in MeOH) to afford 72 mg of **33** in 74% yield, as a 1:1 mixture of diastereomers, isolated together as a yellow solid.

^{1}H NMR (500 MHz, CDCl3) ***δ*** 8.75 (dd, J = 2.3, 1.2 Hz, 2H), 7.92 (dd, J = 8.3, 2.3 Hz, 2H), 7.72 (d, J = 8.2 Hz, 2H), 7.14 (s, 2H), 6.84 (s, 2H), 4.85 (dd, J = 10.1, 4.0 Hz, 2H), 3.94 (s, 6H), 3.88 (s, 6H), 3.23 (ddd, J = 17.6, 8.1, 3.3 Hz, 2H), 3.17 – 3.07 (m, 2H), 2.80 (dd, J = 12.4, 4.0 Hz, 8H), 2.72 – 2.61 (m, 2H), 2.45 (dd, J = 12.5, 10.1 Hz, 2H), 2.32 (tdd, J = 11.6, 5.3, 2.7 Hz, 2H), 2.11 (tdd, J = 11.6, 4.8, 2.6 Hz, 2H), 1.94 – 1.83 (m, 2H), 1.83 – 1.65 (m, 4H), 1.57 – 1.48 (m, 2H), 1.45 – 1.17 (m, 6H).

^{13}C NMR (126 MHz, CDCl3) *δ* 207.6, 166.4, 155.5, 149.5, 148.7, 145.6 (q, J = 4.1 Hz), 133.9 (q, J = 3.4 Hz), 129.3, 125.3, 125.1, 124.7, 122.5, 120.0, 107.4, 104.4, 69.4, 64.3 (d, J = 2.2 Hz), 56.2 (d, J = 15.4 Hz), 55.3, 52.3, 45.3 (d, J = 3.4 Hz), 38.6 (d, J = 5.3 Hz), 34.2 (d, J = 7.8 Hz), 33.3 (d, J = 4.3 Hz), 33.1 (d, J = 31.7 Hz), 31.9 (d, J = 37.6 Hz).

^{19}F NMR (471 MHz, CDCl3) *δ* -62.2.

HRMS AMM (ESI-TOF) m/z calcd for $C_{25}H_{30}F_3N_2O_4^+$[M+H]$^+$ 479.2152, found 479.2140

5-(2-ethoxy-5-((4-(2-hydroxy-2-(5-(trifluoromethyl)pyridin-2-yl)ethyl)piperazin-1-yl)sulfonyl)phenyl)-1-methyl-3-propyl-1,6-dihydro-7H-pyrazolo[4,3-d]pyrimidin-7-one (38)

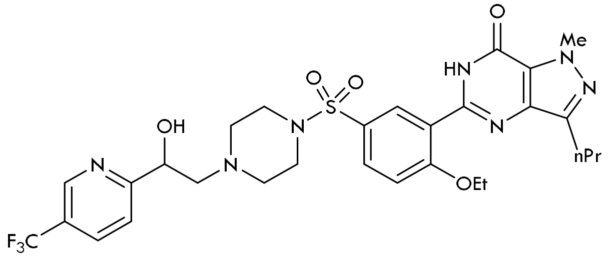

Prepared following the general procedure condition A. The crude material was purified by flash column chromatography (1-10% MeOH/DCM with 1% v/v conc. Et_3N in MeOH) to afford 110 mg of **38** in 85% yield, isolated as a yellow solid.

^{1}H NMR (500 MHz, CDCl3) δ 10.85 (s, 1H), 8.80 (d, J = 2.4 Hz, 1H), 8.75 (dd, J = 2.3, 1.1 Hz, 1H), 7.90 (dd, J = 8.4, 2.3 Hz, 1H), 7.83 (dd, J = 8.7, 2.4 Hz, 1H), 7.61 (d, J = 8.3 Hz, 1H), 7.17 (d, J = 8.8 Hz, 1H), 4.81 (dd, J = 10.1, 3.6 Hz, 1H), 4.38 (q, J = 6.9 Hz, 2H), 4.26 (s, 3H), 3.90 (s, 1H), 3.13 (s, 4H), 3.03 – 2.87 (m, 2H), 2.84 (dd, J = 12.6, 3.9 Hz, 2H), 2.60 (dt, J = 10.8, 4.8 Hz, 2H), 2.50 (dd, J = 12.8, 10.0 Hz, 1H), 1.85 (q, J = 7.5 Hz, 2H), 1.65 (t, J = 7.0 Hz, 3H), 1.01 (t, J = 7.4 Hz, 3H).

^{13}C NMR (126 MHz, CDCl3) δ 165.2, 159.5, 153.7, 147.1, 146.5, 145.8 (d, J = 4.0 Hz), 138.5, 134.1 (d, J = 3.3 Hz), 131.8, 131.2, 129.0, 125.8, 125.5, 124.6, 122.5, 121.3, 120.1, 113.3, 69.6, 66.2, 63.9, 52.3, 46.2, 38.3, 27.8, 22.4, 14.7, 14.2.

^{19}F NMR (471 MHz, CDCl3) δ -62.3.

HRMS AMM (ESI-TOF) m/z calcd for $C_{29}H_{35}F_3N_7O_5S^+$[M+H]$^+$ 650.2367, found 650.2338

General Procedure for Telescoped Decarboxylative Alkylation, Deprotection, and Characterization of Amino Alcohols (40-42)

Conditions: To an 20 mL vial (**Vial 1**), equipped with a stir bar, was added Ir[dF(CF_3)ppy]$_2$(dtbpy)PF_6 (11 mg, 0.01 mmol, 1 mol %), potassium 3-(tert-butoxycarbonyl)-2,2-dimethyloxazolidine-4-carboxylate (340 mg, 1.2 mmol, 1.2 eq.), and phthalimide (147 mg, 1.0 mmol. 1 eq.). To a separate 20 mL vial (**Vial 2**), $NiBr_2$·DME (6.1 mg, 0.02 mmol, 2 mol %) and dtbbpy (5.4 mg, 0.02 mmol, 2 mol%) were added. Both vials were added into a nitrogen-filled glovebox. 5 mL of DMF was added to each vial (0.1 M total). The liquid aryl bromides (1 mmol, 1 eq) were added to **Vial 1**. Note: if the aryl bromide is a solid, it is added to **Vial 1** before entering the glovebox. **Vial 2** was allowed to stir for 10 min, at which point the contents of **Vial 2** were added to **Vial 1**. **Vial 1** was then sealed with electrical tape, removed from the glovebox, and irradiated with two 34W Kessil PR160 456 LEDs (1 cm away, with cooling from fans to keep reactions at room temperature) for 48 hours. Afterwards, the reactions are removed from the lights. The reaction mixture is diluted with water (50 mL), and extracted three times with EtOAc (30 mL). The combined organic layers were then washed once with a saturated aqueous LiCl solution (20 mL), and once with a 1 M aqueous solution of KOH (20 mL). Afterwards, the organic layers were dried with sodium sulfate, filtered, and evaporated under reduced pressure. The material was passed through a silica plug and the organic solvent was evaporated under reduced pressure. To the residue, 10 mL of DCM, 2 mL of water, and 1 mL of trifluoroacetic acid (TFA) was added. The mixture was stirred at 50 °C for 2 hr, after which the material was evaporated under reduced pressure. Residual TFA was azeotroped off with small volumes of methanol. This mixture was then diluted with DCM (5 mL, 0.2 M), to which di-tert-butyl decarbonate (436 mg, 2 mmol, 2 eq) and triethylamine (280 μL, 2 mmol, 2 eq) was added. The reaction was stirred for 2 hr at room temperature, then quenched with 10 mL water. The reaction was extracted three times with DCM (30 mL total), and the mixture was dried with sodium sulfate, filtered, and evaporated under reduced pressure. Products were purified with flash column chromatography.

5-(2-ethoxy-5-((4-(2-hydroxy-2-(5-(trifluoromethyl)pyridin-2-yl)ethyl)piperazin-1-yl)sulfonyl)phenyl)-1-methyl-3-propyl-1,6-dihydro-7H-pyrazolo[4,3-d]pyrimidin-7-one (40)

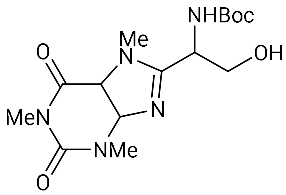

Prepared following the general procedure condition. The eluent used for the silica plug was 200 mL of EtOAc/Hex = 70:30. The crude material was purified by flash column chromatography (EtOAc/Hex = 60:40) to afford 152 mg in 43% yield as a white solid.

`**^{1}H NMR (500 MHz, CDCl3)** δ 5.54 (d, J = 9.2 Hz, 1H), 4.97 (dd, J = 8.3, 4.5 Hz, 1H), 4.05 (s, 3H), 3.90 (dd, J = 11.5, 4.3 Hz, 1H), 3.54 (s, 3H), 3.40 (s, 3H), 1.44 (s, 9H).

^{13}C NMR (126 MHz, CDCl3) δ 155.5, 152.0, 151.7, 147.2, 107.8, 80.8, 64.5, 60.6, 46.7, 32.4, 30.0, 28.4, 28.2.

HRMS AMM (ESI-TOF) m/z calcd for $C_{15}H_{21}NNaO_5^+[M+H]^+$ 318.1312, found 318.1311

methyl 4-(1-((tert-butoxycarbonyl)amino)-2-hydroxyethyl)benzoate (41)

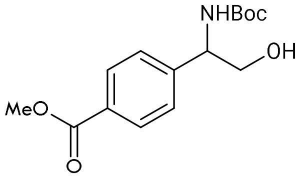

Prepared following the general procedure condition. The eluent used for the silica plug was 200 mL of EtOAc/Hex = 5:95. The crude material was purified by flash column chromatography (EtOAc/Hex = 30:70) to afford 156 mg in 53% yield as a clear oil.

^{1}H NMR (500 MHz, CDCl3) δ 8.03 (d, J = 8.4 Hz, 2H), 7.38 (d, J = 8.2 Hz, 2H), 5.33 (d, J = 7.2 Hz, 1H), 4.83 (s, 1H), 3.91 (s, 3H), 3.88 (d, J = 4.1 Hz, 1H), 2.17 (s, 9H).

^{13}C NMR (126 MHz, CDCl3) δ 166.9, 144.6, 130.0, 129.9, 126.1, 110.2, 77.5, 71.6, 52.3, 26.6, 26.0.

HRMS AMM (ESI-TOF) m/z calcd for $C_{15}H_{22}NO_5^+[M+H]^+$ 296.1498, found 296.1519

tert-butyl (1-(benzo[b]thiophen-5-yl)-2-hydroxyethyl)carbamate (42)

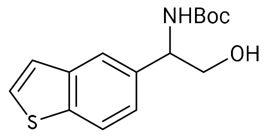

Prepared following the general procedure condition. The eluent used for the silica plug was 200 mL of EtOAc/Hex = 5:95. The crude material was purified by flash column chromatography (EtOAc/Hex = 20:80) to afford 152 mg in 52% yield as a white solid

^{1}H NMR (500 MHz, CDCl3) δ 7.87 (d, J = 8.4 Hz, 1H), 7.77 (d, J = 1.7 Hz, 1H), 7.47 (d, J = 5.4 Hz, 1H), 7.32 (dd, J = 5.5, 0.8 Hz, 1H), 7.28 (dd, J = 8.4, 1.8 Hz, 1H), 4.91 (s, 1H), 3.91 (d, J = 4.8 Hz, 2H), 1.44 (s, 9H).

^{13}C NMR (126 MHz, CDCl3) δ 156.2, 140.1, 139.2, 135.9, 127.4, 123.9, 123.1, 121.7, 80.2, 67.2, 57.0, 29.8, 28.5.

HRMS AMM (ESI-TOF) m/z calcd for $C_{15}H_{20}NO_3S^+$ $[M+H]^+$ 294.1164, found 294.1181

General Procedure for Telescoped Oxidation-Reductive Amination Sequence, and Characterization of Diamines (43-44)

Conditions: To an 8 mL vial, Dess Martin Periodinane (84.8 mg, 0.2 mmol, 1 eq) was added to aryl amino alcohol (0.2 mmol, 1 eq) in a solution of DCM (2 mL, 0.1 M). The reaction mixture was allowed to stir for 16 h, after which it was quenched with 2 mL of saturated sodium bicarbonate and 2 mL of saturated sodium thiosulfate. This stirred for 30 minutes, which was then extracted using DCM three times (18 mL total), washed with saturated brine (10 mL), dried with sodium sulfate, filtered, and evaporated under reduced pressure. To the crude residue, acetic acid (50 μL, 0.8 mmol, 4 eq) was added alongside amine substrate (0.4 mmol, 2 eq) and methanol (2 mL, 0.1 M). This stirred for 2 hr at room temperature, after which sodium cyanoborohydide (25 mg, 0.4 mmol, 2 eq) was added. This then stirred for 16 hr at room temperature. This mixture was quenched with water (10 mL), extracted three times with DCM (30 mL total), dried with sodium sulfate, filtered, and evaporated under reduced pressure. Each compound was purified via flash column chromatography.

methyl 4-(1-((tert-butoxycarbonyl)amino)-2-morpholinoethyl)benzoate (43)

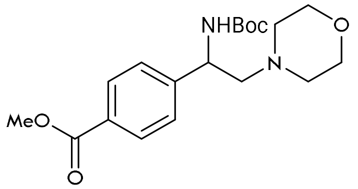

Prepared following the general procedure condition. The crude material was purified by flash column chromatography (EtOAc/Hex = 60:40) to afford 72.8 mg in 73% yield as a clear oil.

^{1}H NMR (400 MHz, CDCl3) δ 8.06 – 7.86 (m, 2H), 7.36 (dd, J = 8.2, 5.9 Hz, 2H), 5.56 (s, 1H), 4.95 – 4.54 (m, 1H), 3.89 (s, 3H), 3.76 – 3.59 (m, 4H), 2.71 – 2.19 (m, 5H), 1.41 (s, 9H).

^{13}C NMR (126 MHz, CDCl3) δ 167.0, 155.8, 130.1, 130.0, 130.0, 129.2, 126.7, 126.2, 66.9, 63.9, 52.3, 52.2, 28.4, 28.4·

HRMS AMM (ESI-TOF) m/z calcd for $C_{19}H_{29}N_2O_5^+$[M+H]$^+$ 365.2071, found 365.2076

tert-butyl (1-(benzo[b]thiophen-5-yl)-2-(4-(8-chloro-5,6-dihydro-11H-benzo[5,6]cyclohepta[1,2-b]pyridin-11-ylidene)piperidin-1-yl)ethyl)carbamate (44)

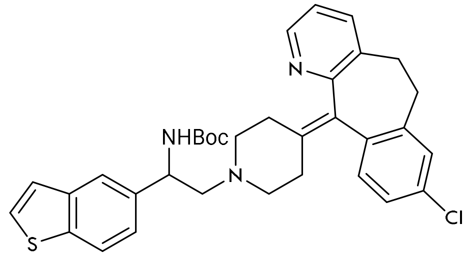

Prepared following the general procedure condition. The crude material was purified by flash column chromatography (EtOAc = 100) to afford 67 mg in 57% yield as a yellow oil.

^{1}H NMR (500 MHz, CDCl3) δ 8.39 (dd, J = 4.8, 1.7 Hz, 1H), 7.81 (d, J = 8.3 Hz, 1H), 7.74 (s, 1H), 7.50 – 7.33 (m, 2H), 7.30 – 7.27 (m, 1H), 7.23 – 6.94 (m, 4H), 5.71 (s, 1H), 4.77 (s, 1H), 3.57 – 3.23 (m, 2H), 3.07 – 2.72 (m, 3H), 2.73 – 2.16 (m, 8H), 1.40 (s, 9H).

^{13}C NMR (126 MHz, CDCl3) δ 171.2, 155.9, 155.9, 146.7, 146.7, 139.9, 139.6, 138.6, 137.8, 137.4, 133.4, 132.8, 130.8, 129.0, 126.8, 126.1, 123.8, 122.7, 122.6, 122.2, 121.1, 79.5, 60.4, 31.8, 31.5, 29.7, 28.4, 21.1, 14.2.

HRMS AMM (ESI-TOF) m/z calcd for $C_{34}H_{37}ClN_3O_2S^+$[M+H]$^+$ 586.2290, found 586.2284

References

(1) Rad, M. S.; Somayeh Behrouz; Abdo-Reza Nekoei. 8-Bromocaffeine (8-BC): A New Versatile Reagent for Conversion of Aldoximes into Nitriles. *Synlett* **2012**, *23* (08), 1191–1198. https://doi.org/10.1055/s-0031-129036.

(2) Furuya, T.; Ritter, T. Fluorination of Boronic Acids Mediated by Silver(I) Triflate. *Organic letters* **2009**, *11* (13), 2860–2863. https://doi.org/10.1021/ol901113t.

(3) Matuszewski, M.; Sochacka, E. Stability Studies on the Newly Discovered Cyclic Form of TRNA N6-Threonylcarbamoyladenosine (Ct6A). *Bioorganic & Medicinal Chemistry Letters* **2014**, *24* (12), 2703–2706. https://doi.org/10.1016/j.bmcl.2014.04.048.

(4) Xu, J. A New and Expeditious Asymmetric Synthesis of (R)- and (S)-2-Aminoalkanesulfonic Acids from Chiral Amino Alcohols. *Tetrahedron: Asymmetry* **2002**, *13* (11), 1129–1134. https://doi.org/10.1016/S0957-4166(02)00312-9.

(5) Lujan, B.; Zhang, M.; Cao, Y.; Kacker, A.; Mai, L.; Wu, S.; Alexander, T.; Huang, W.; Kou, K. G. M. Semisynthesis of Bersavine and Berbamine Derivatives That Target the CaMKIIγ:cMyc Axis for Lymphoma Therapy. *Organic & Biomolecular Chemistry* **2025**, *23* (18), 4403–4408. https://doi.org/10.1039/d5ob00310e.

(6) He, D. Y.; Li, Z. J.; Li, Z. J.; Liu, Y. Q.; Qiu, D. X.; Cai, M. S. Studies on Carbohydrates X. A New Method for the Preparation of Isopropylidene Saccharides. *Synthetic Communications* **1992**, *22* (18), 2653–2658. https://doi.org/10.1080/00397919208021665.

(7) Giorgio Milli; Pellegrini, A.; Listro, R.; Fasolini, M.; Pagano, K.; Ragona, L.; Giampiero Pietrocola; Linciano, P.; Collina, S. New LsrK Ligands as AI-2 Quorum Sensing Interfering Compounds against Biofilm Formation. *Journal of Medicinal Chemistry* **2024**. https://doi.org/10.1021/acs.jmedchem.4c01266.

^{1}H, ^{13}C NMR and ^{19}F NMR spectra of all compounds

5-bromo-N-(6-methylpyridin-2-yl)furan-2-carboxamide (S3)

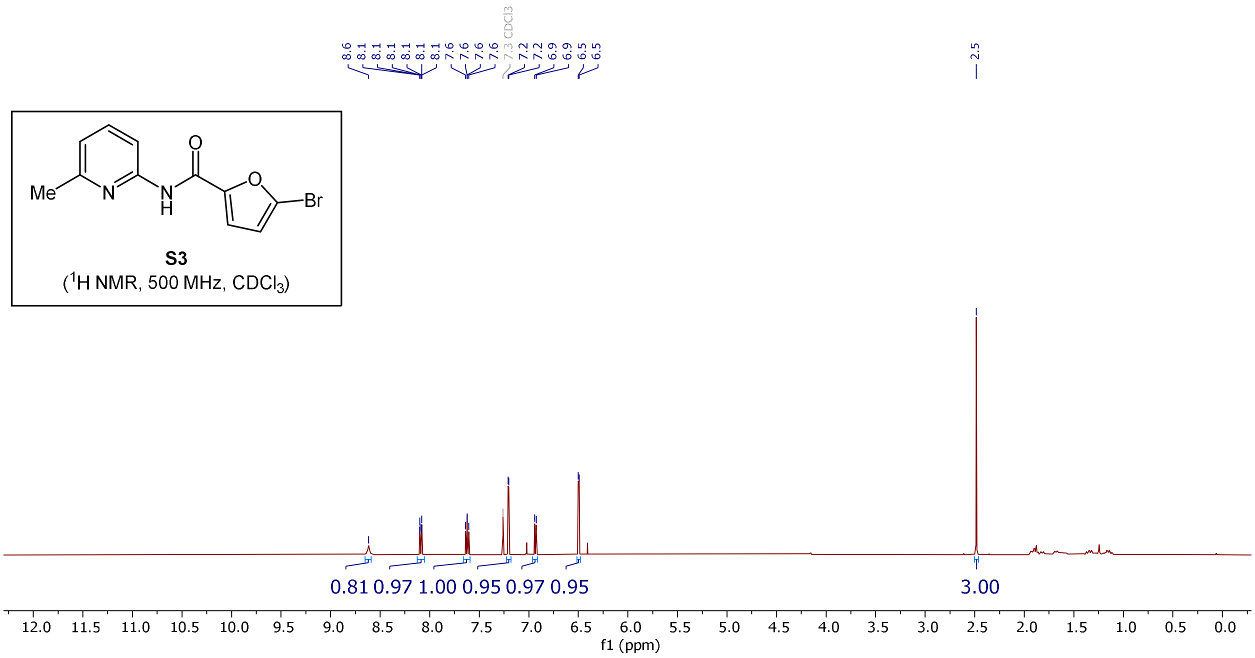

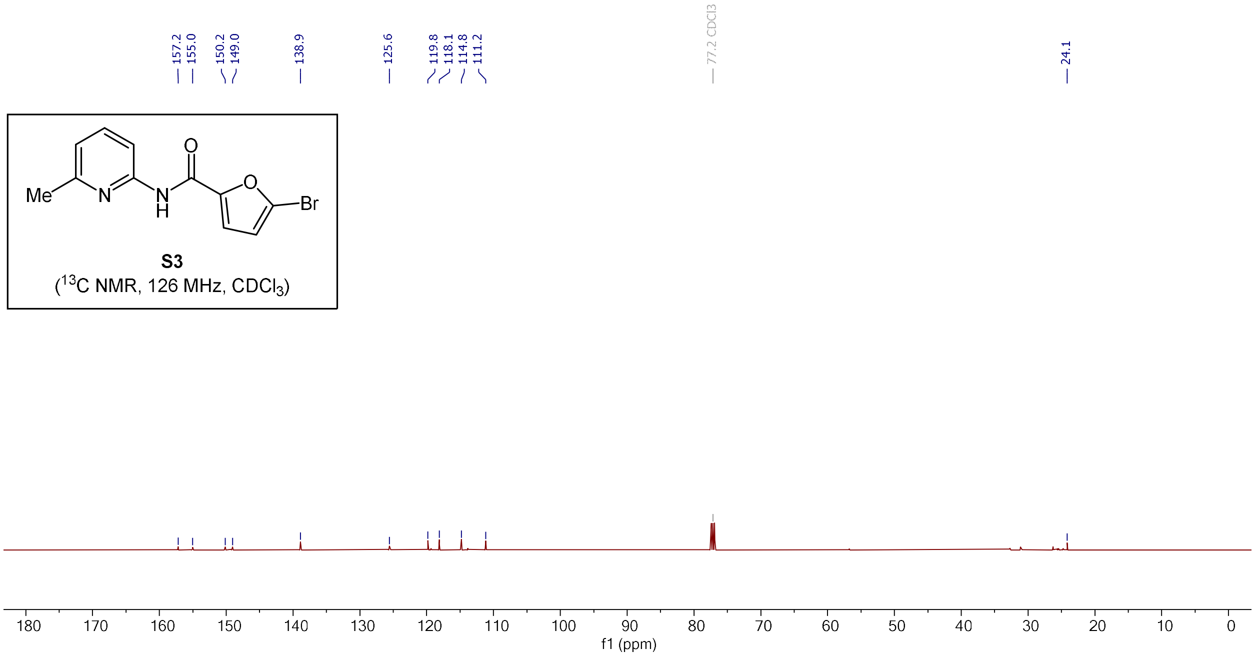

8-bromo-1,3,7-trimethyl-3,7-dihydro-1H-purine-2,6-dione (S12)

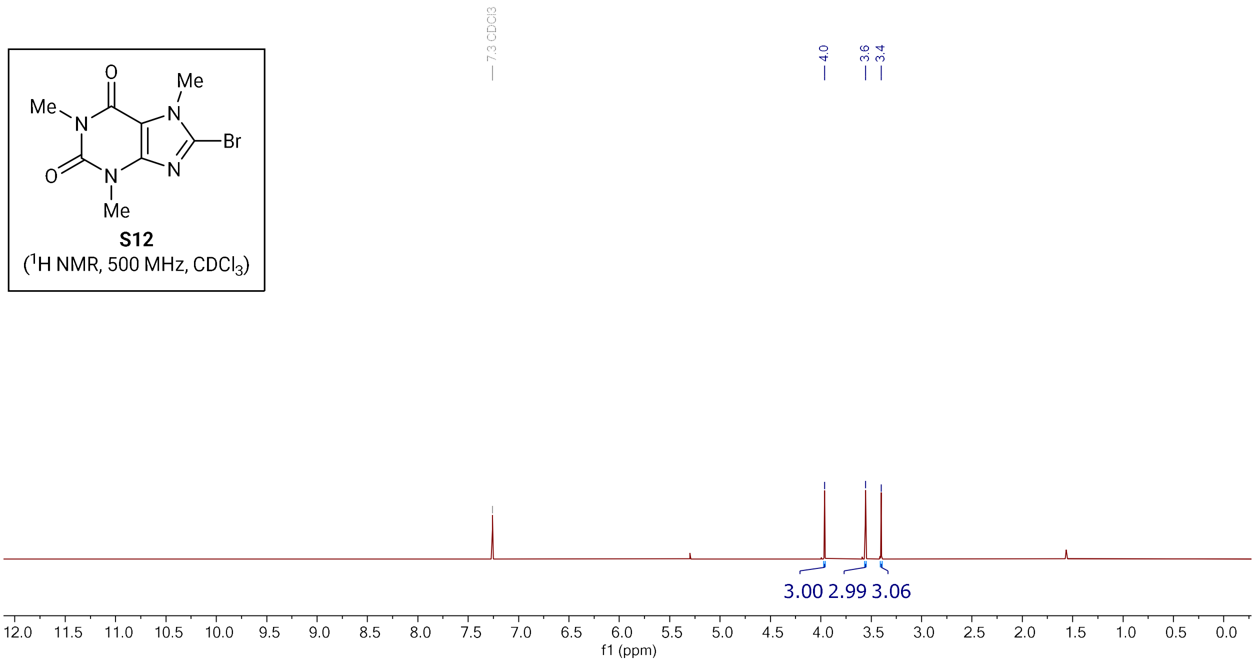

tert-butyl 5-bromo-1H-indole-1-carboxylate (S15)

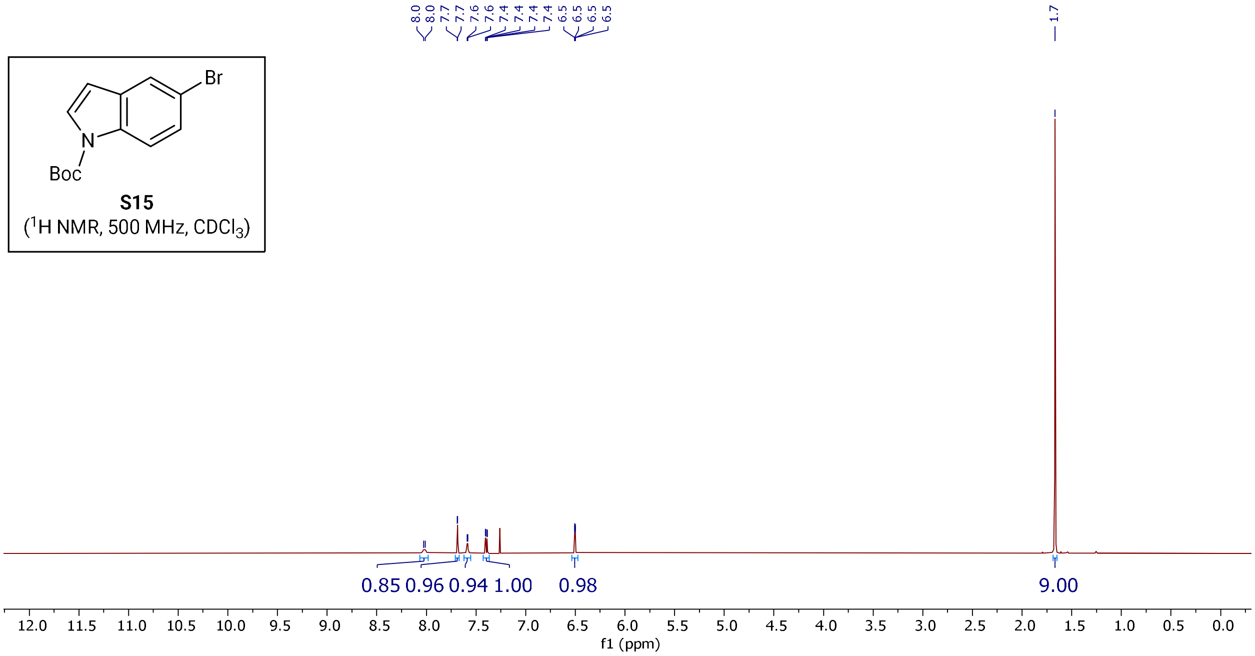

tert-butyl 5-bromo-1H-indazole-1-carboxylate (S16)

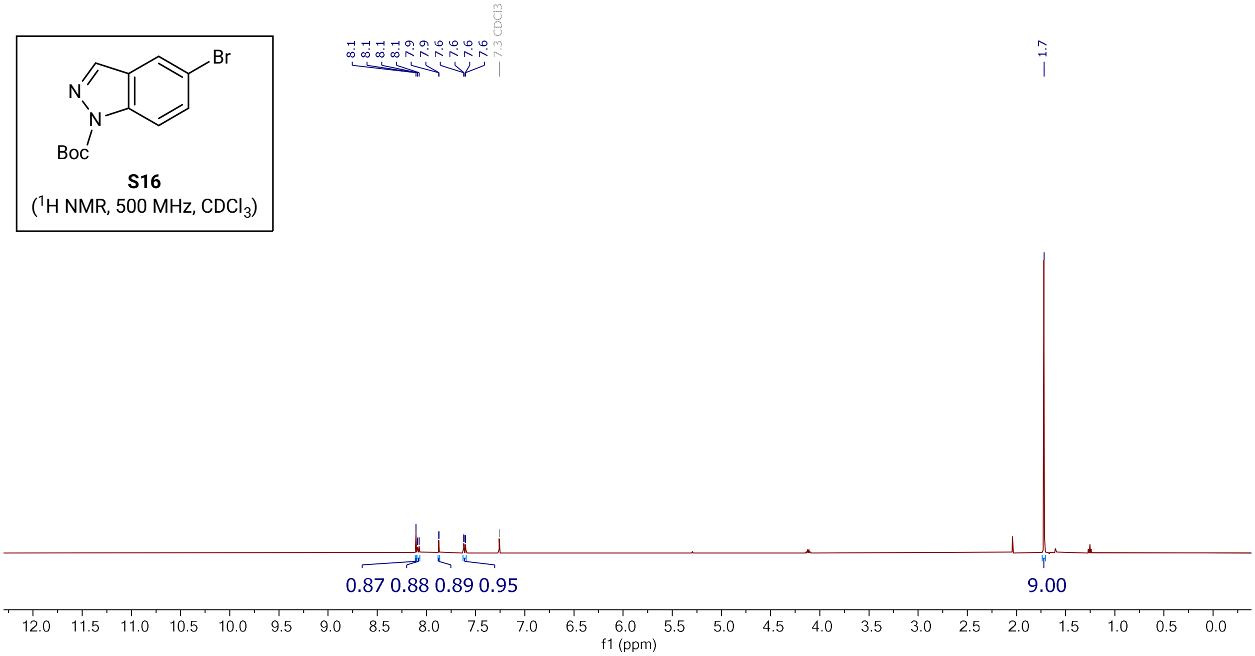

(2R,3R,4R,5R)-2-(acetoxymethyl)-5-(6-amino-8-bromo-9H-purin-9-yl)tetrahydrofuran-3,4-diyl diacetate (S19)

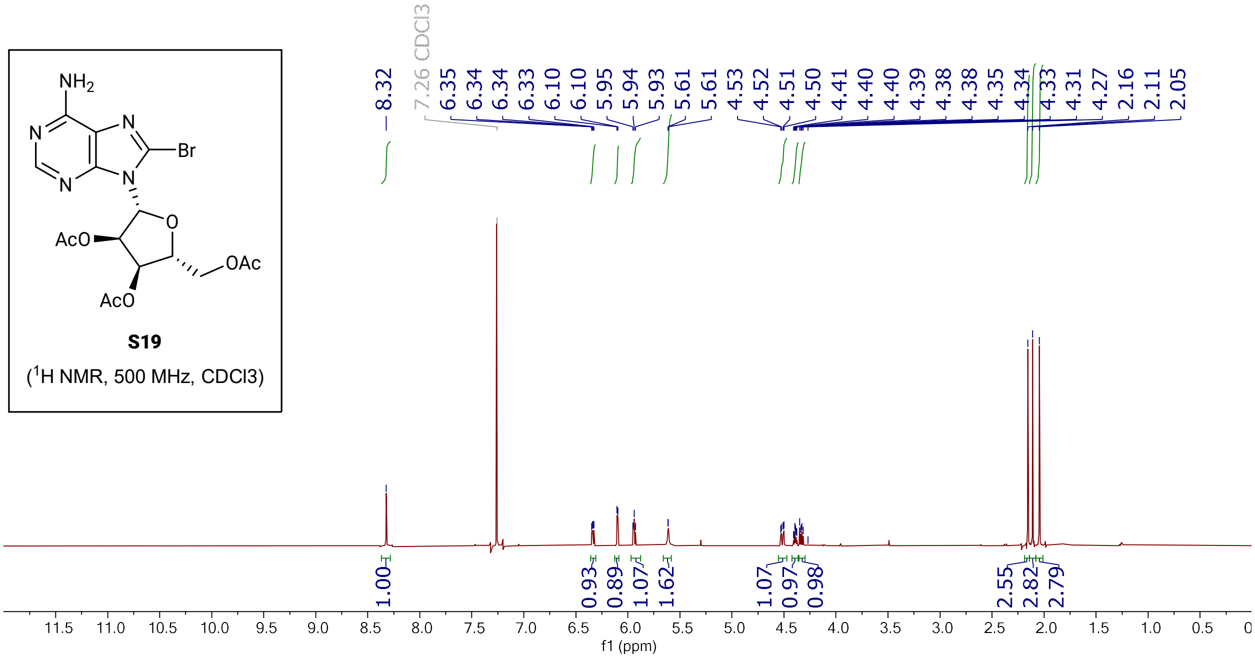

(S)-2-benzylaziridine (S22)

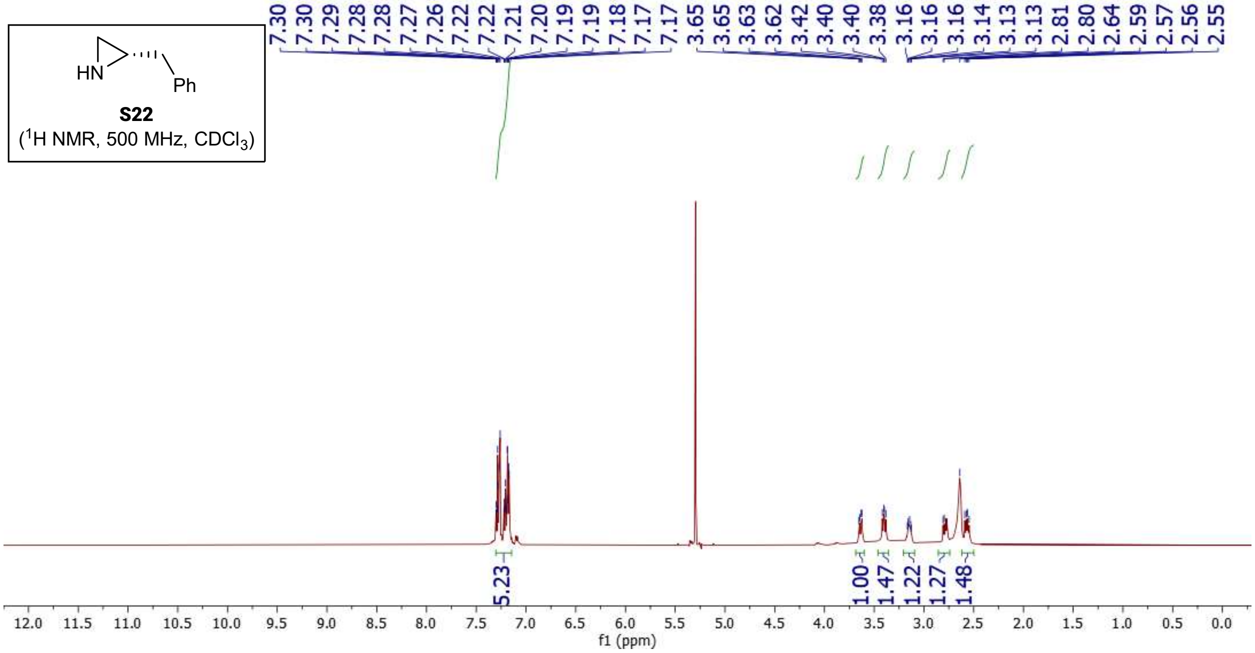

Tert-butyl piperazine-1-carboxylate (S26)

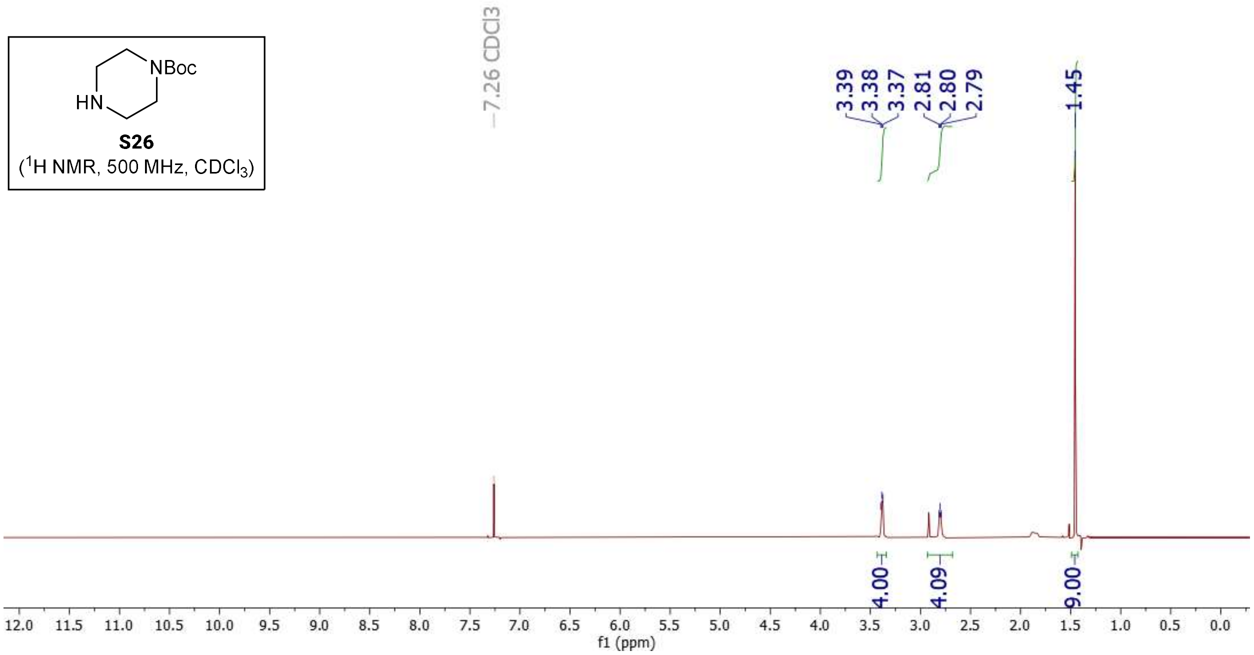

potassium 2,2-dimethyl-1,3-dioxolane-4-carboxylate (1)

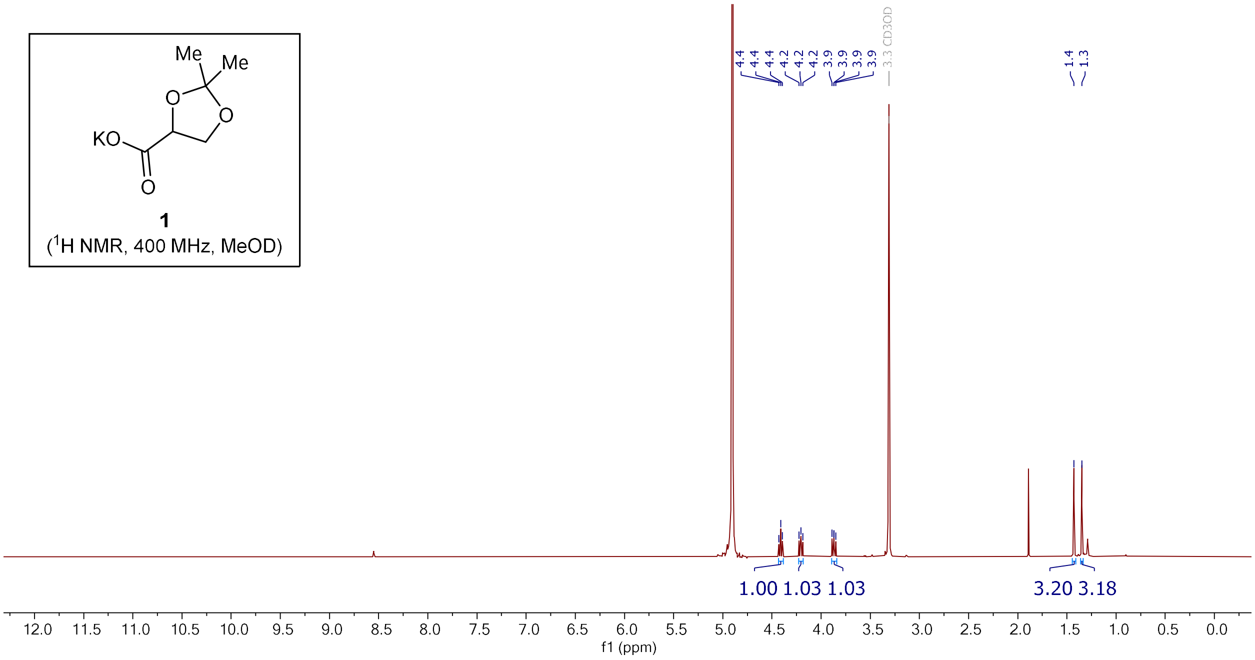

potassium 3-(tert-butoxycarbonyl)-2,2-dimethyloxazolidine-4-carboxylate (39)

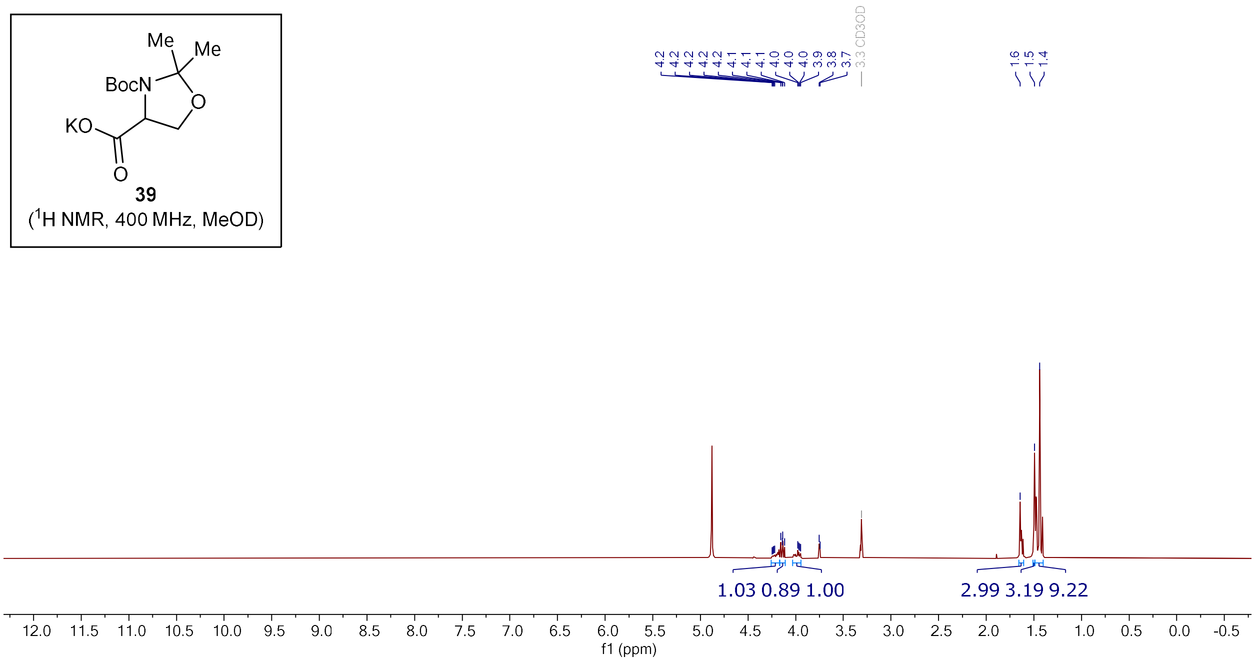

2-(2,2-dimethyl-1,3-dioxolan-4-yl)-5-(trifluoromethyl)pyridine (2)

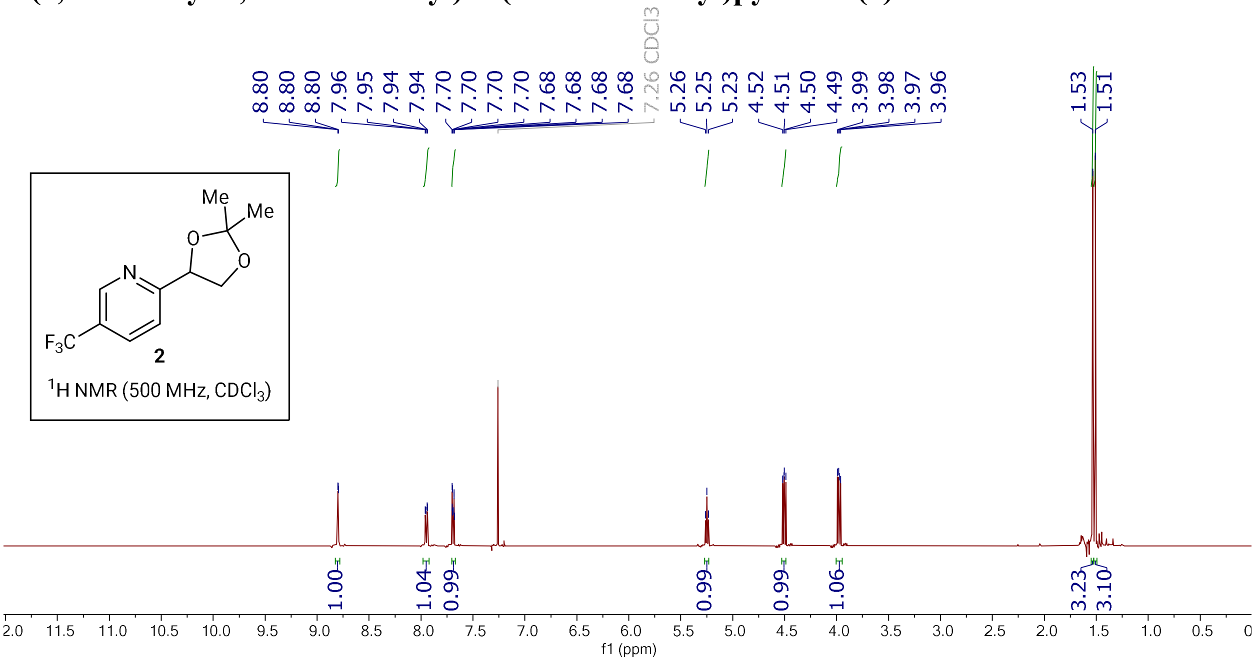

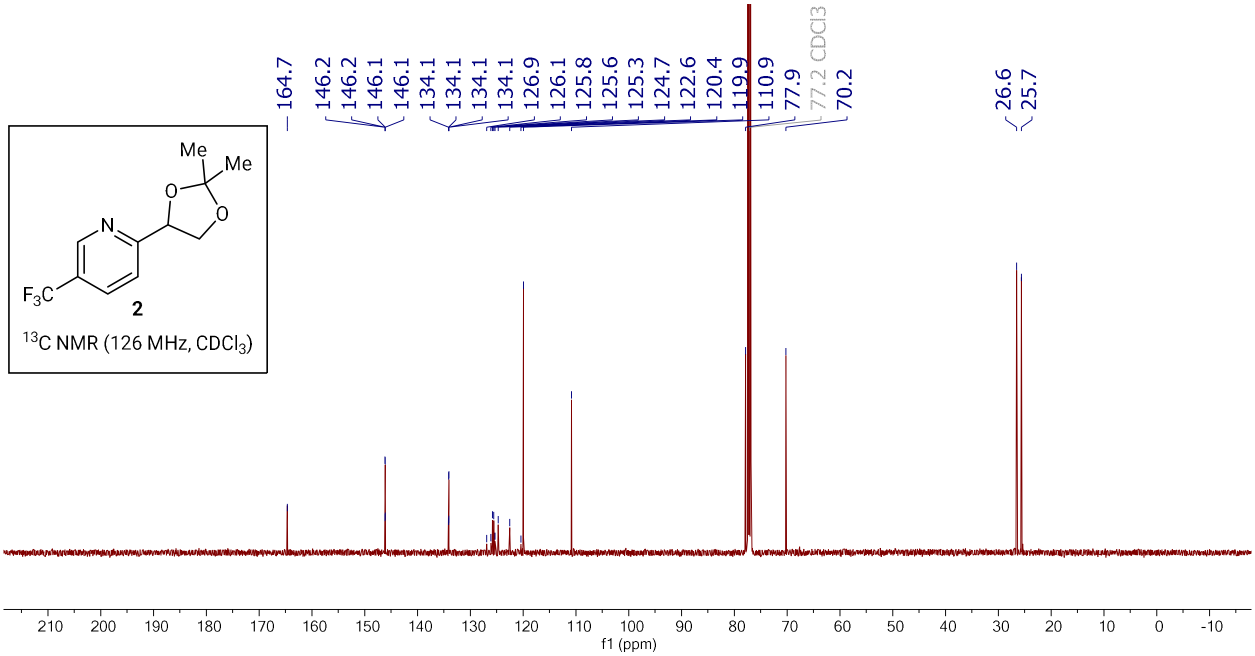

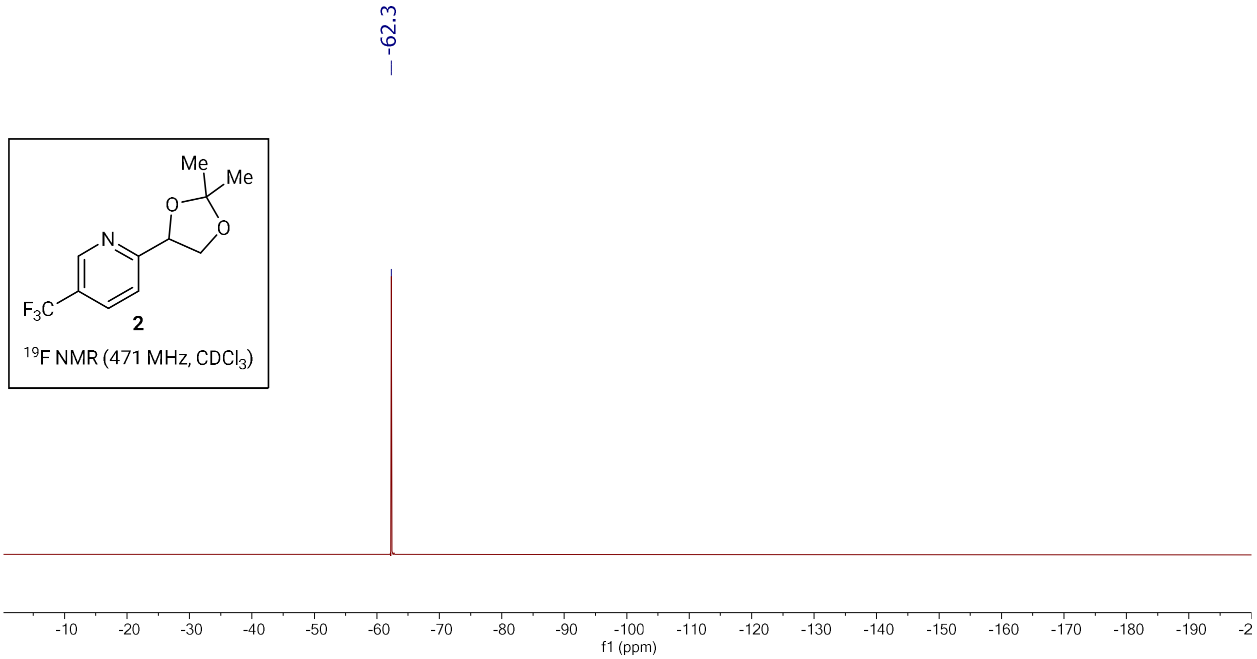

-62.3
Me
Me
O
O
N
F3C
2
19F NMR (471 MHz, CDCl3)
-10
-20
-30
-40
-50
-60
-70
-80
-90
-100
-110
-120
-130
-140
-150
-160
-170
-180
-190
-2
f1 (ppm)

5-(2,2-dimethyl-1,3-dioxolan-4-yl)-N-(6-methylpyridin-2-yl)furan-2-carboxamide (3)

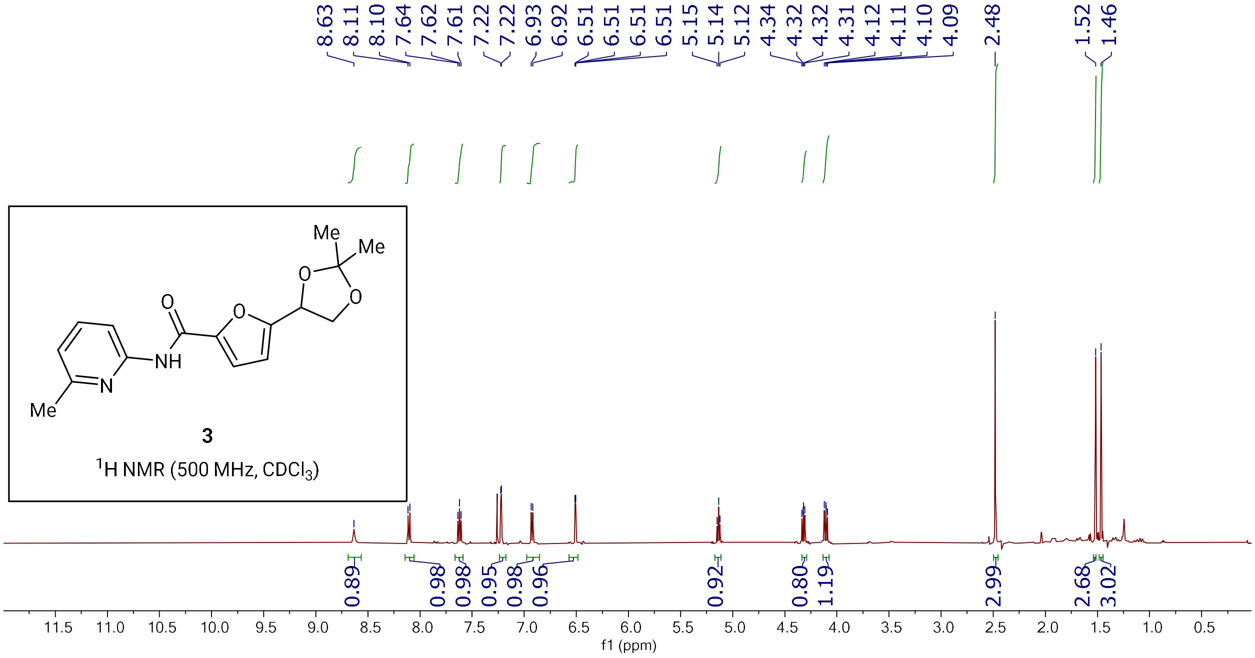

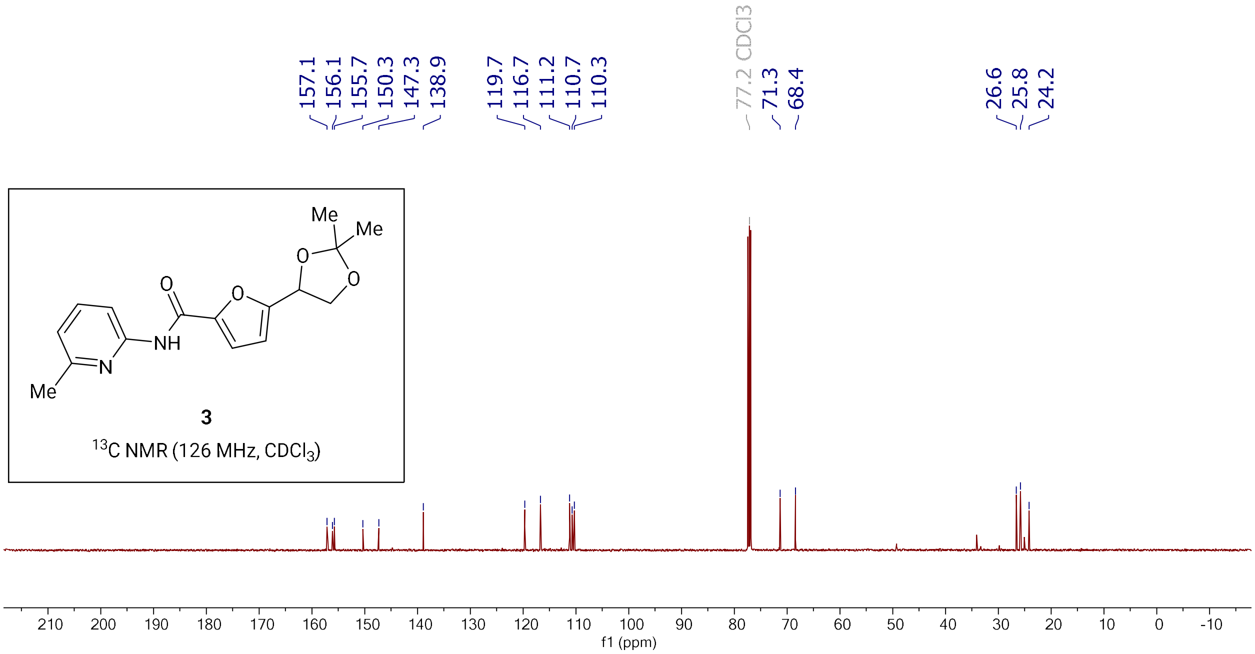

5-(2,2-dimethyl-1,3-dioxolan-4-yl)benzofuran (4)

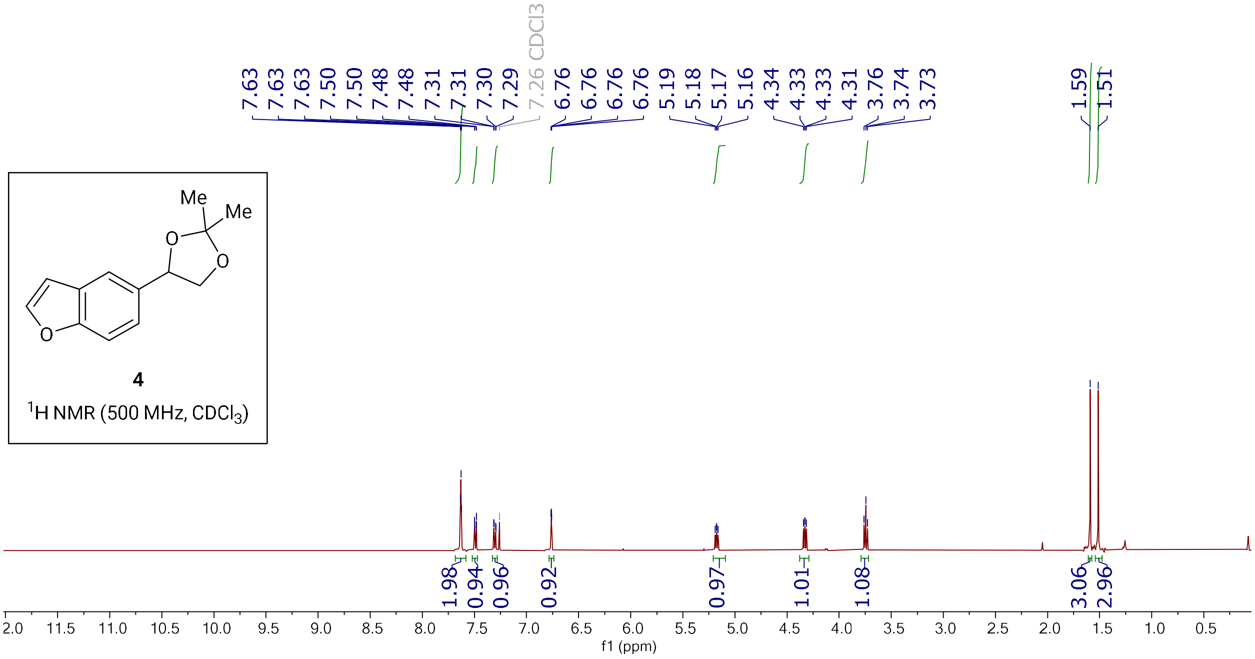

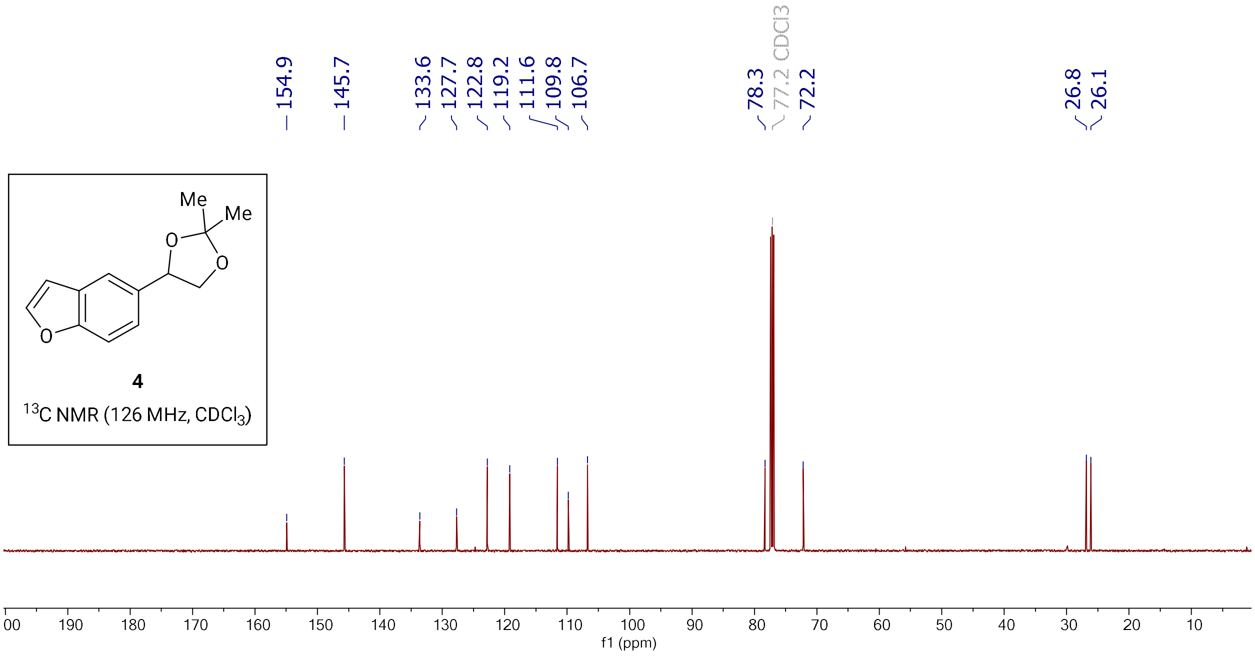

4-(2,2-dimethyl-1,3-dioxolan-4-yl)picolinonitrile (5)

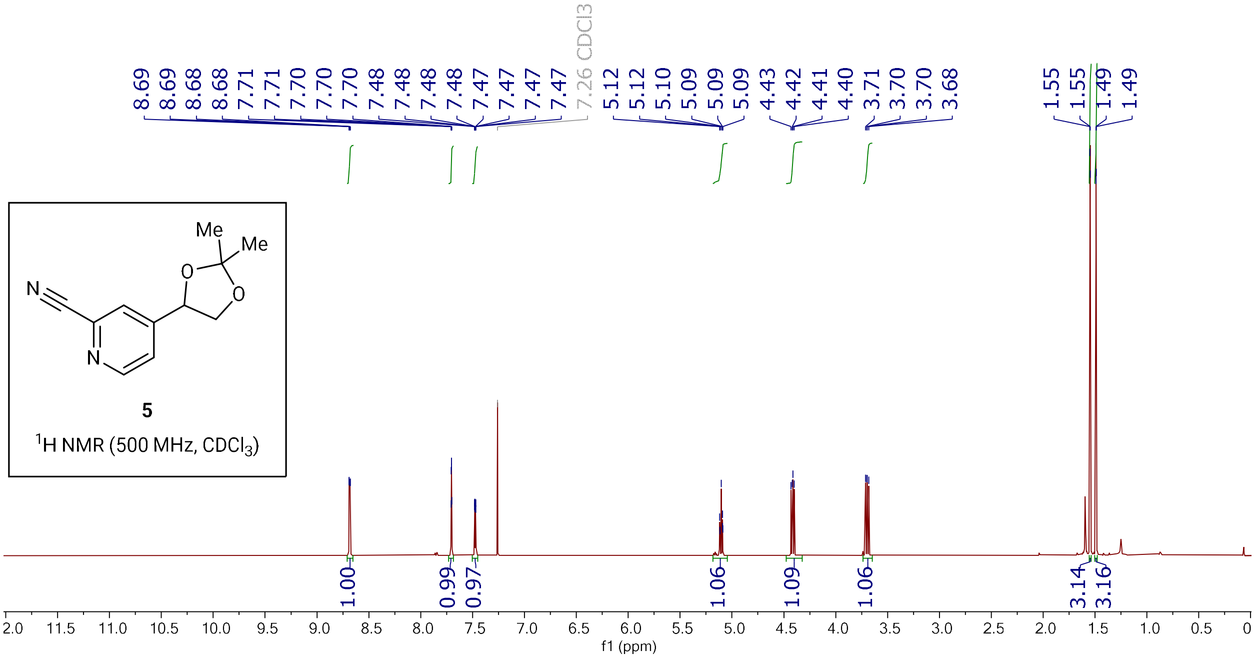

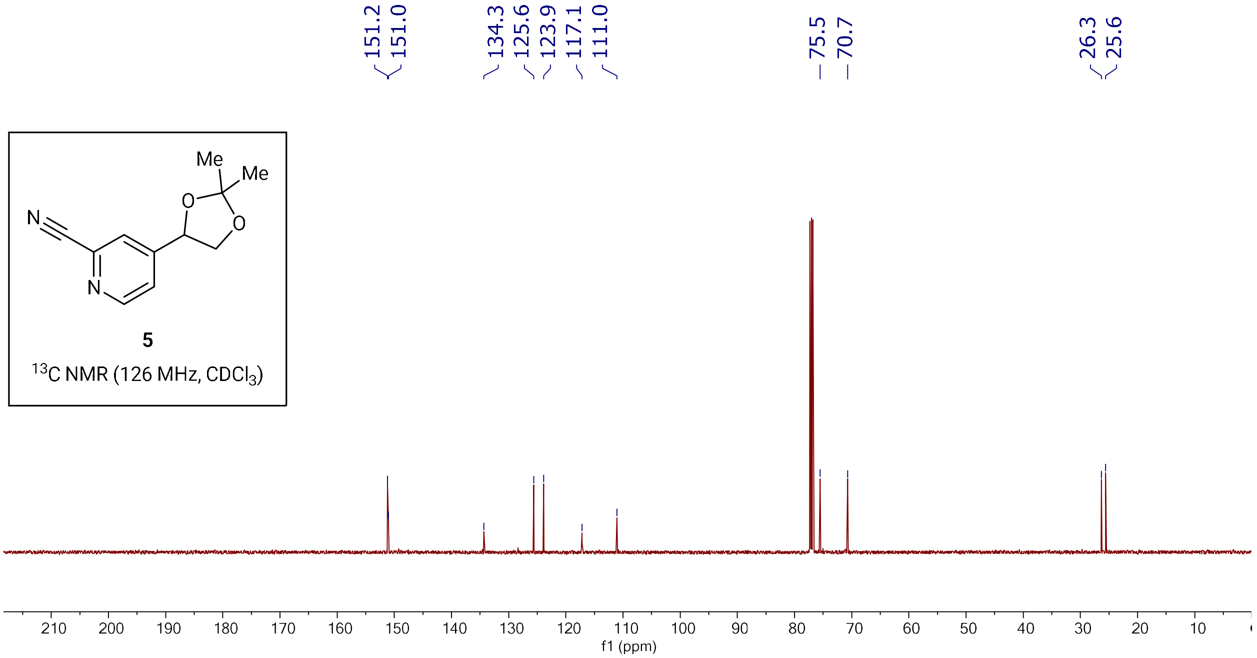

3-(2,2-dimethyl-1,3-dioxolan-4-yl)pyridine (6)

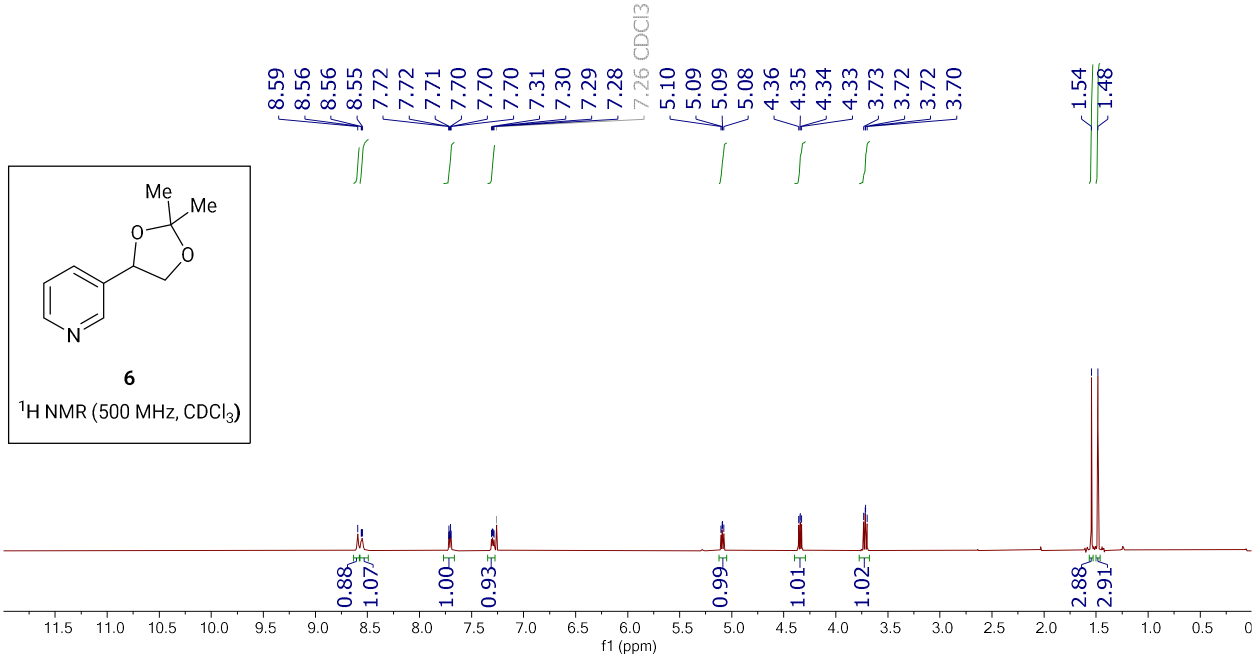

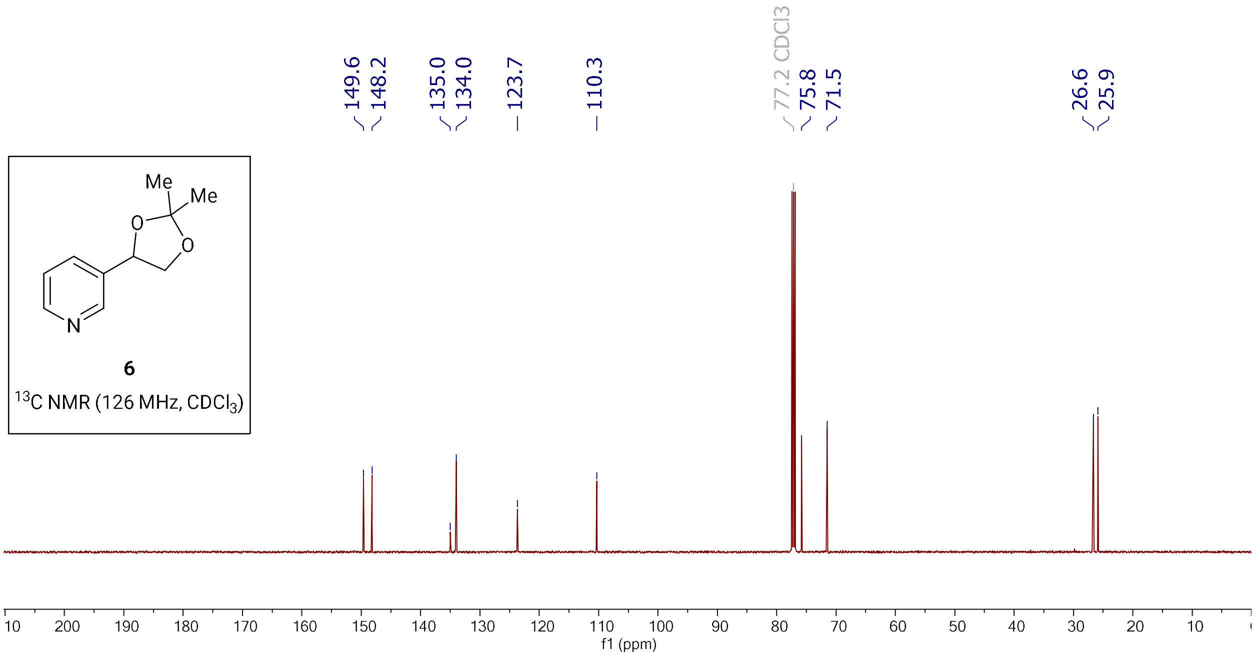

6-(2,2-dimethyl-1,3-dioxolan-4-yl)quinoxaline (7)

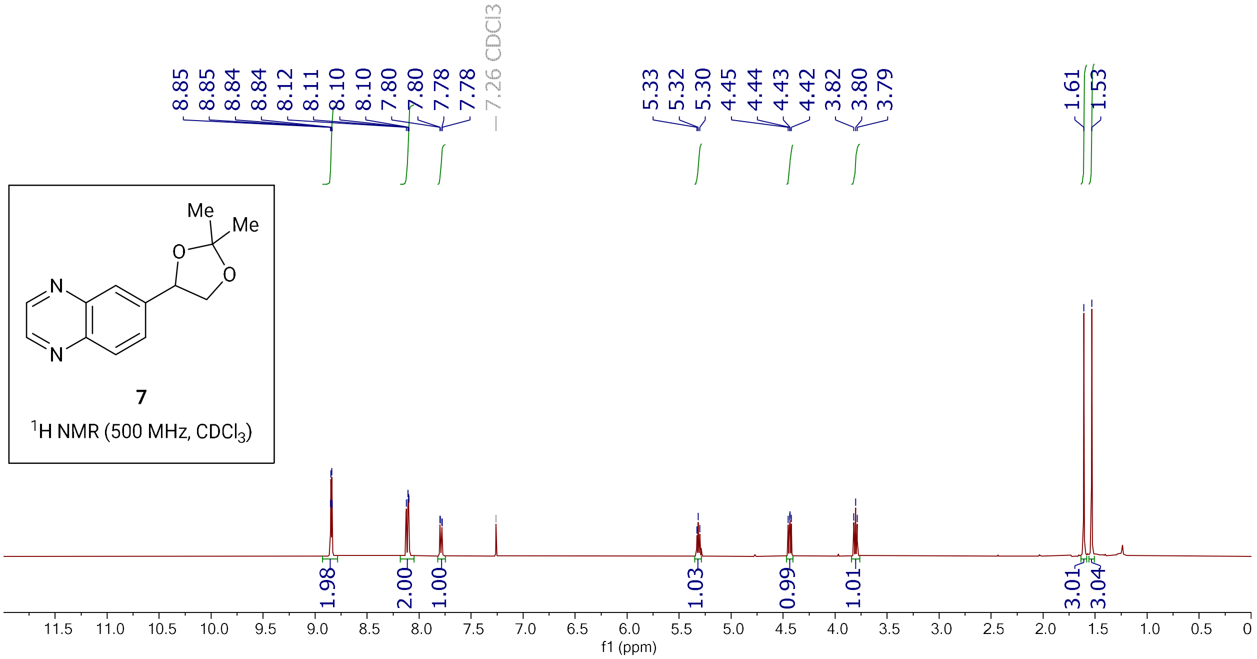

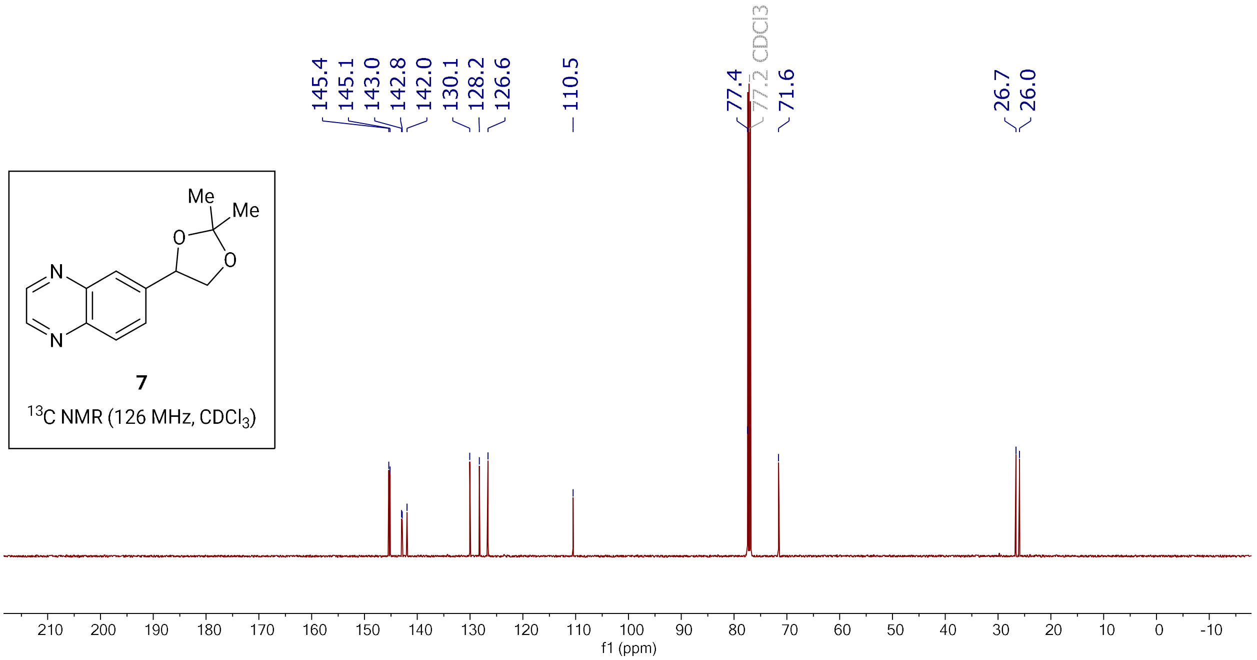

2-(2,2-dimethyl-1,3-dioxolan-4-yl)pyrazine (8)

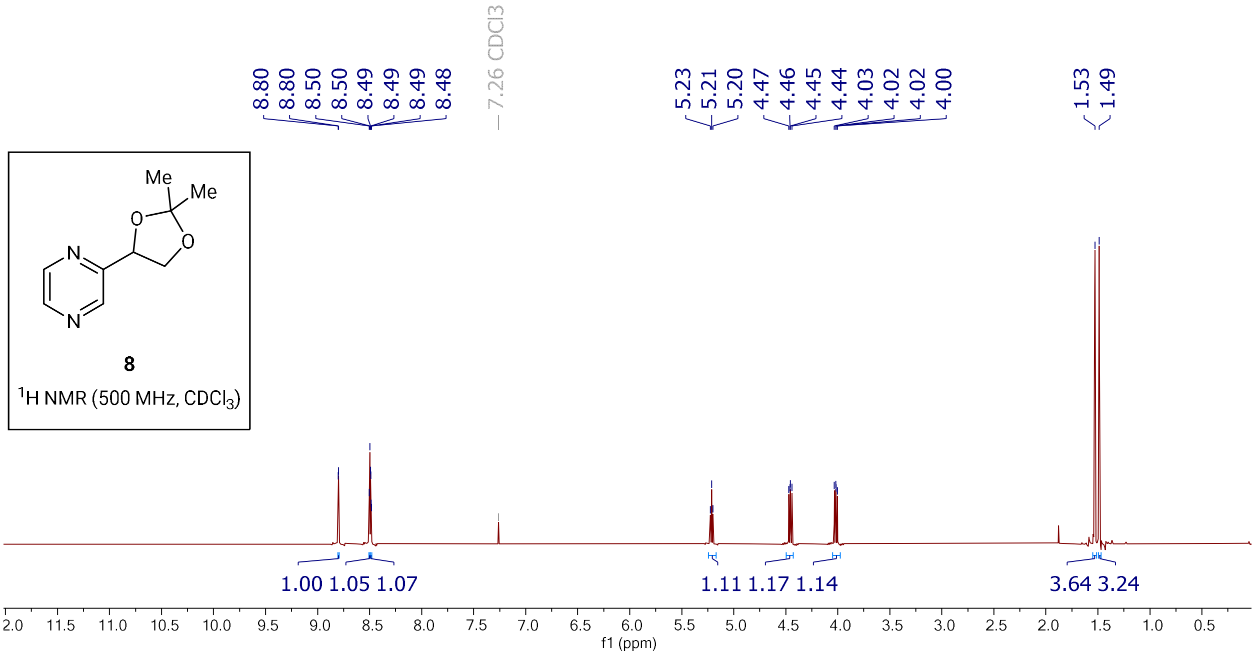

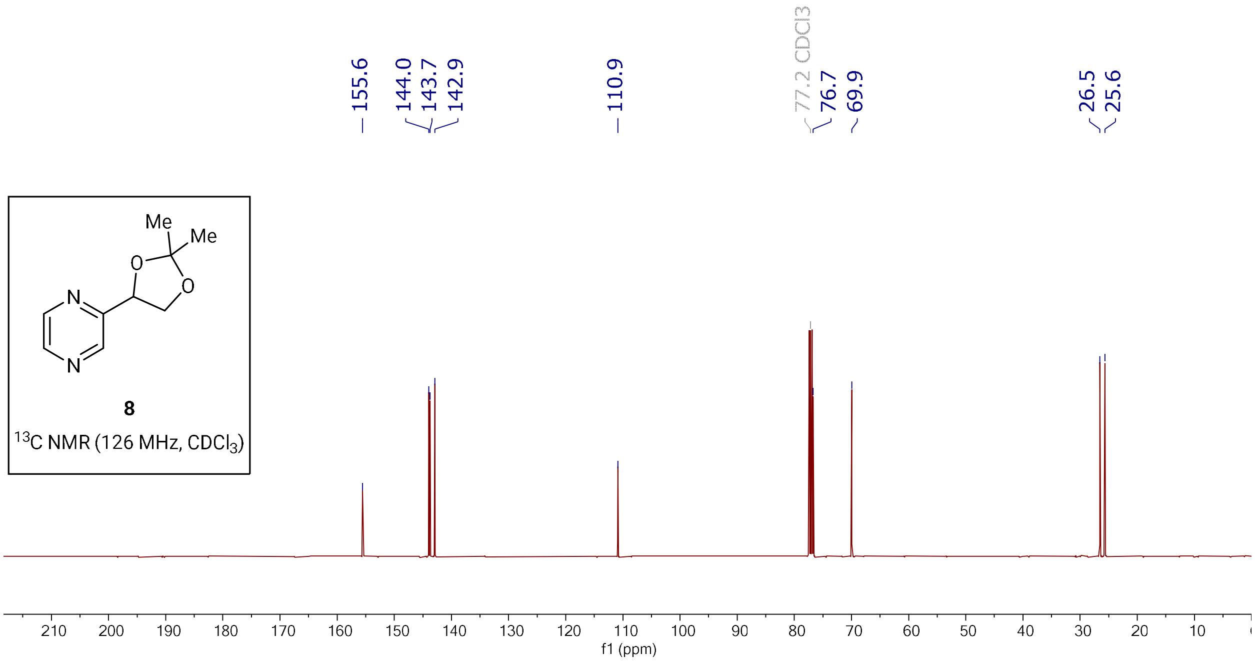

2-(2,2-dimethyl-1,3-dioxolan-4-yl)benzo[d]thiazole (9)

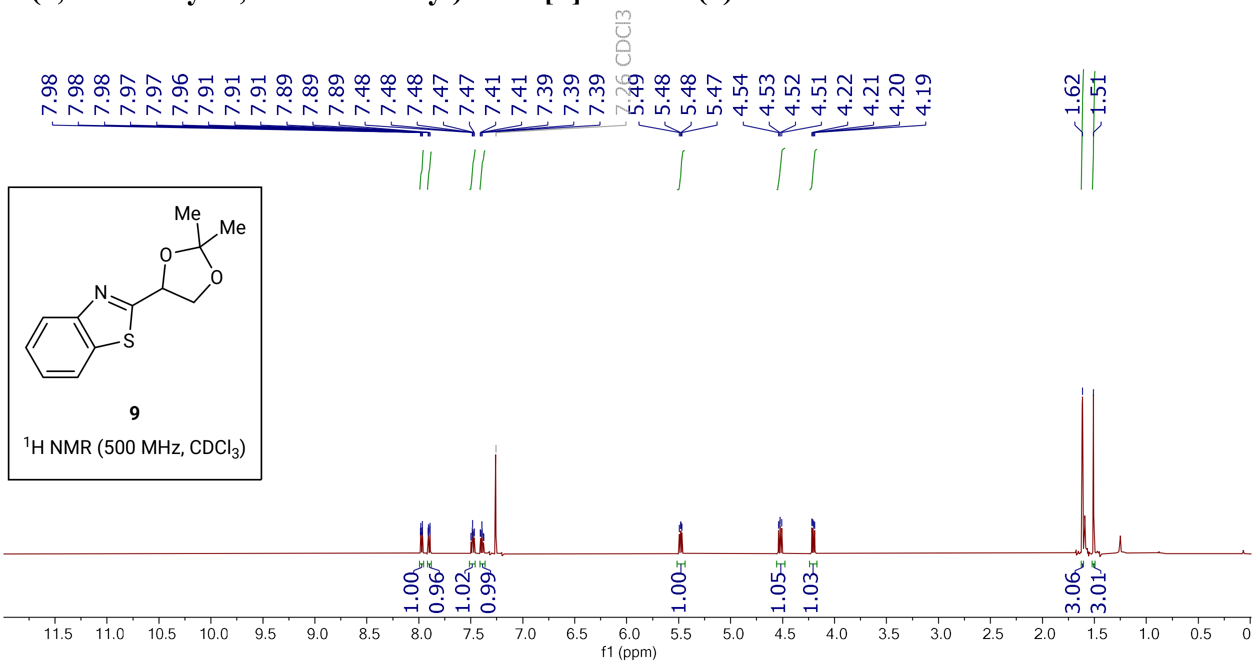

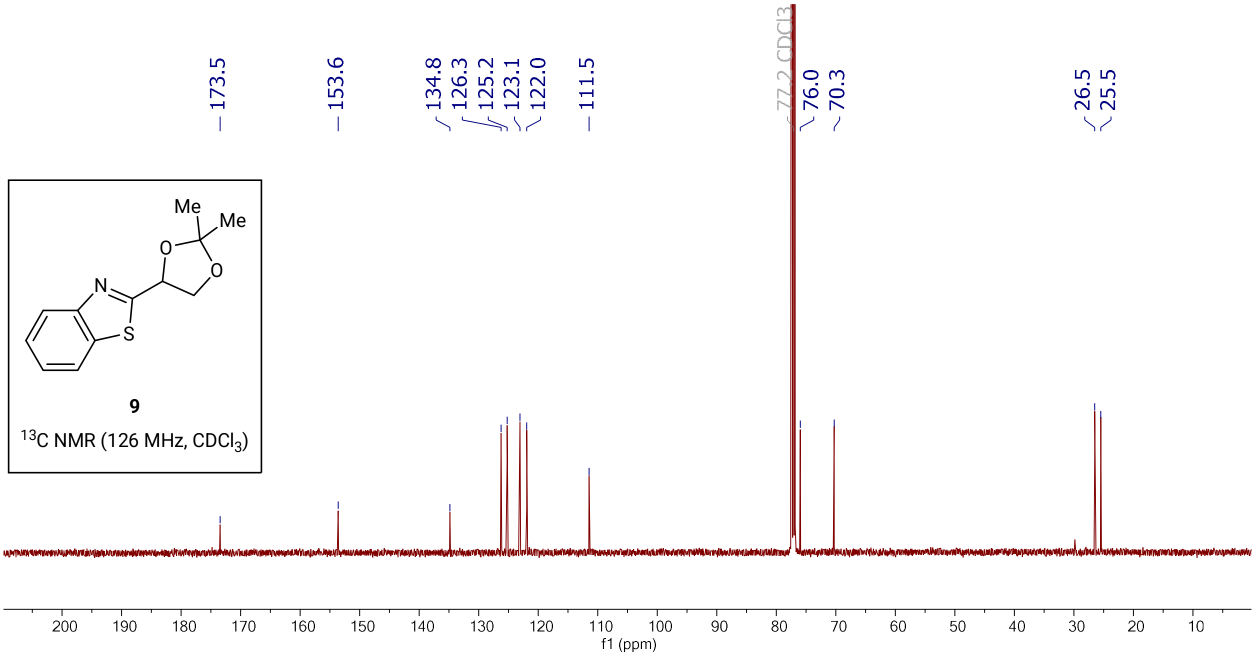

methyl 4-(2,2-dimethyl-1,3-dioxolan-4-yl)benzoate (10)

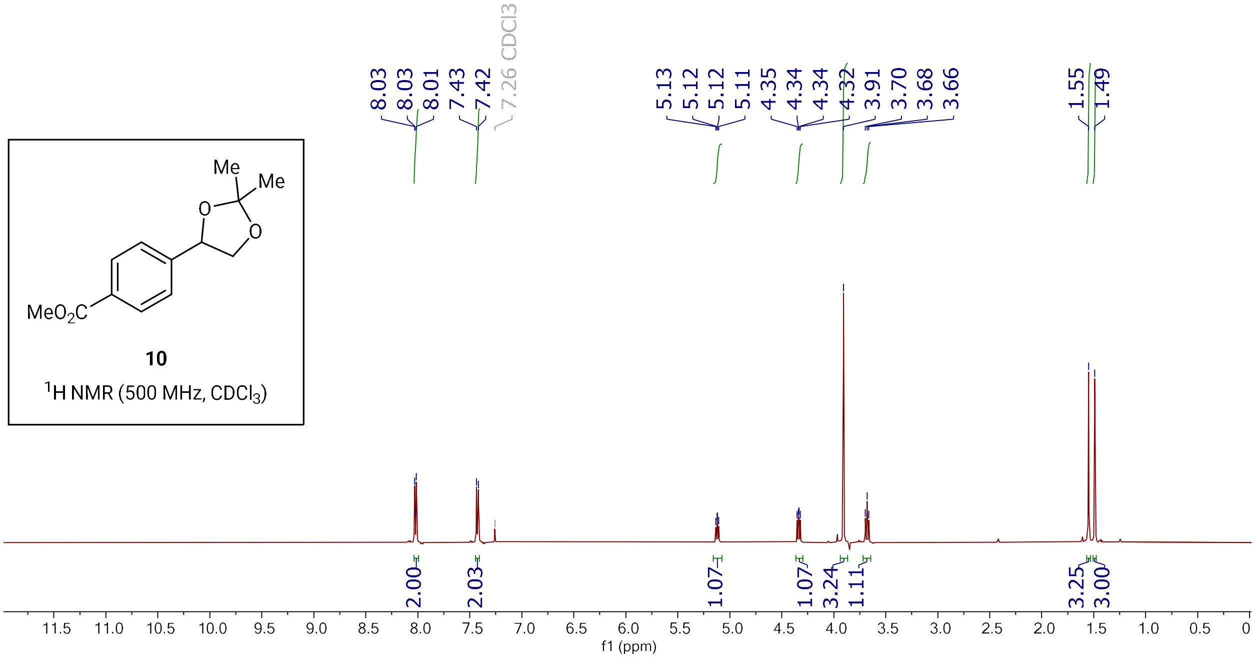

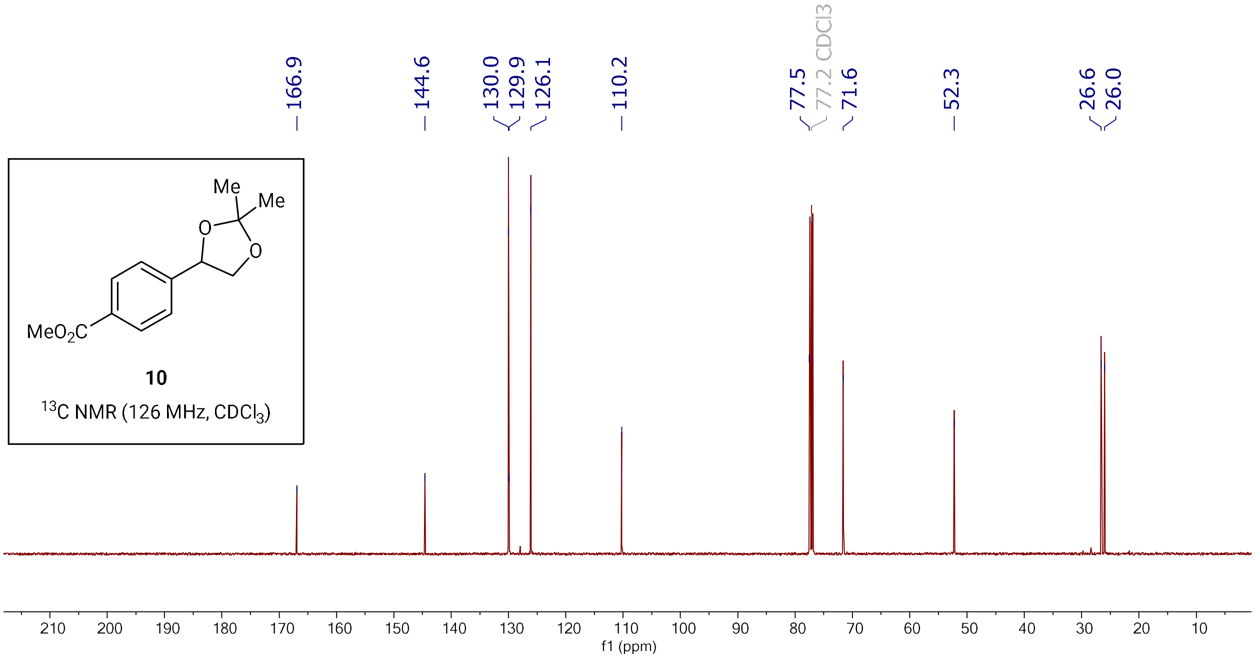

3-(2,2-dimethyl-1,3-dioxolan-4-yl)benzonitrile (11)

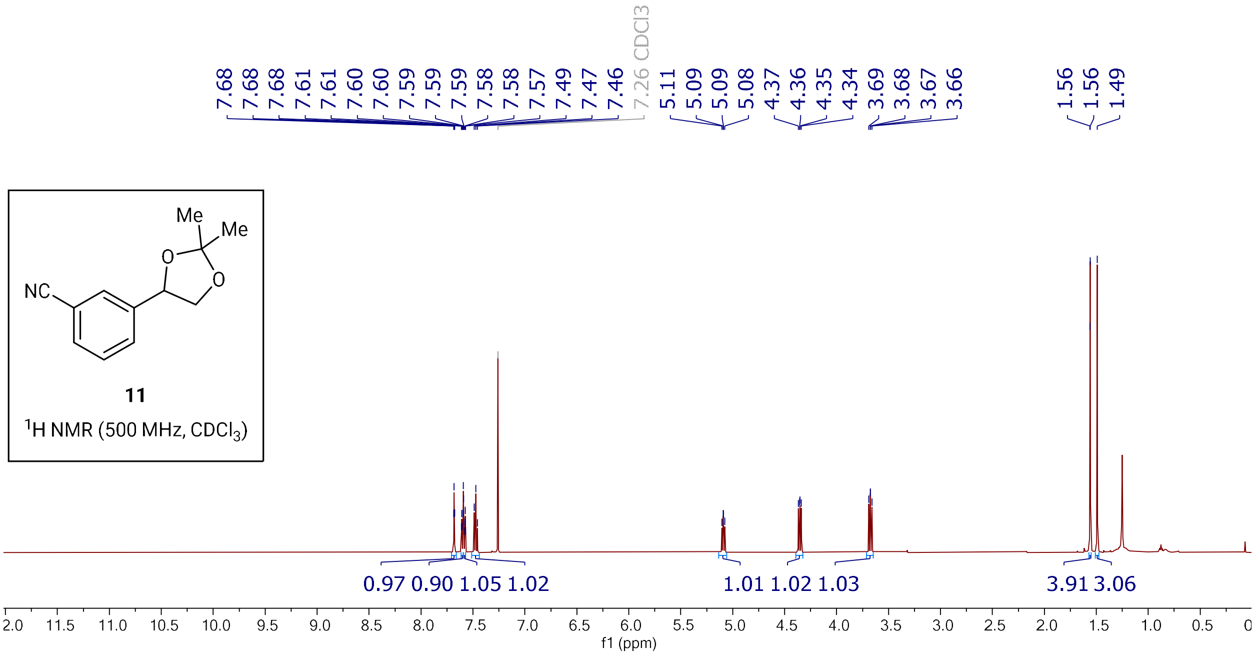

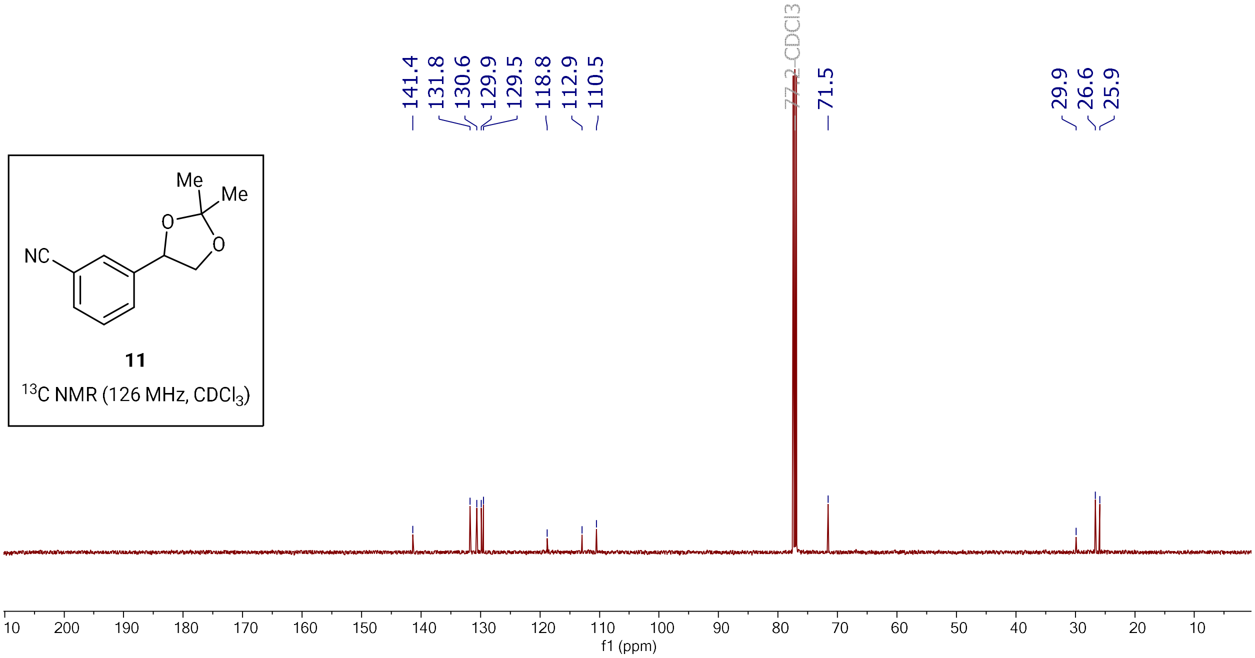

8-(2,2-dimethyl-1,3-dioxolan-4-yl)-1,3,7-trimethyl-3,7-dihydro-1H-purine-2,6-dione (12)

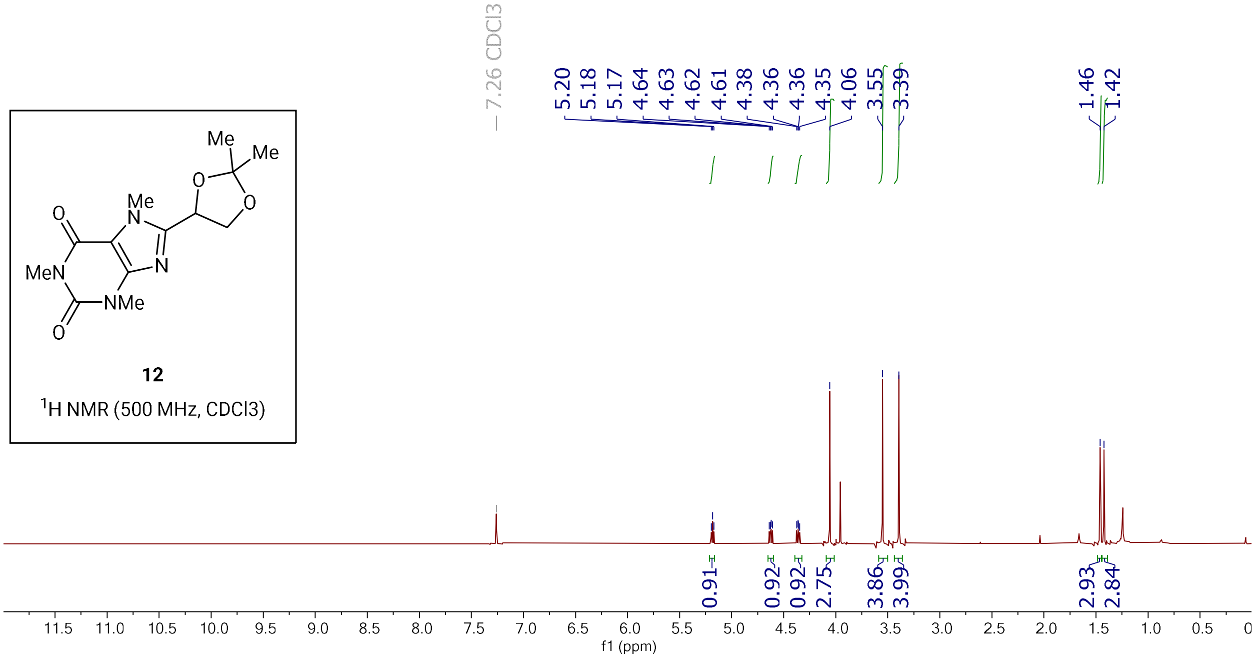

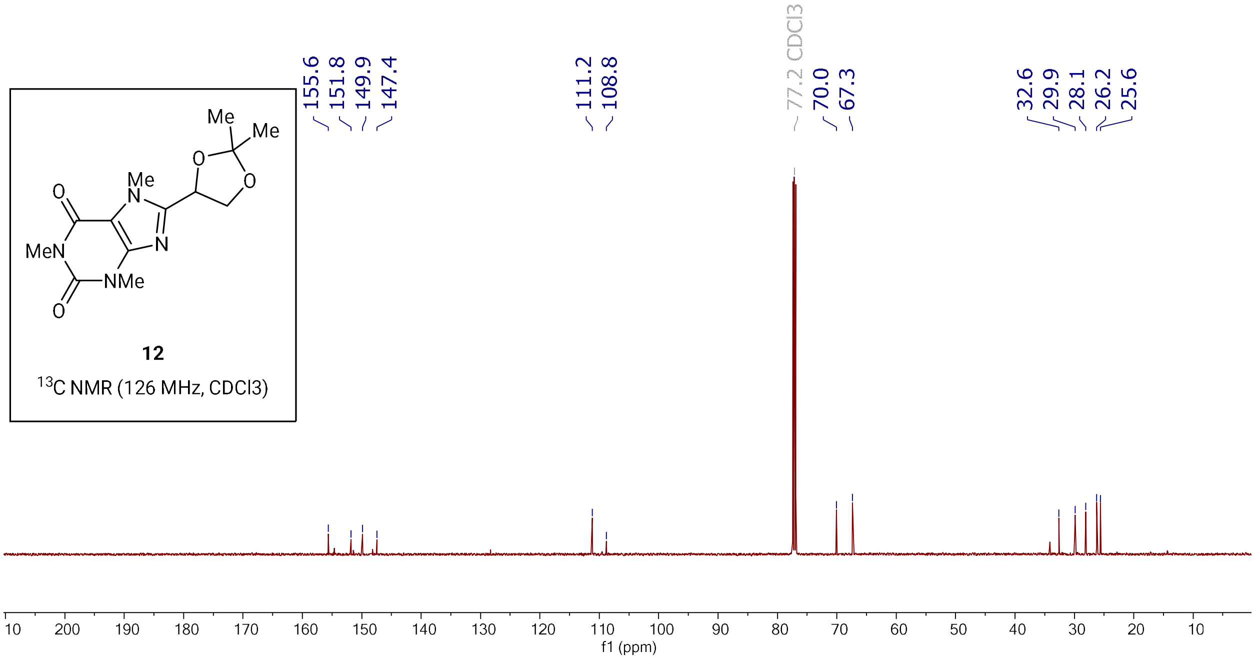

6-(2,2-dimethyl-1,3-dioxolan-4-yl)imidazo[1,2-b]pyridazine (13)

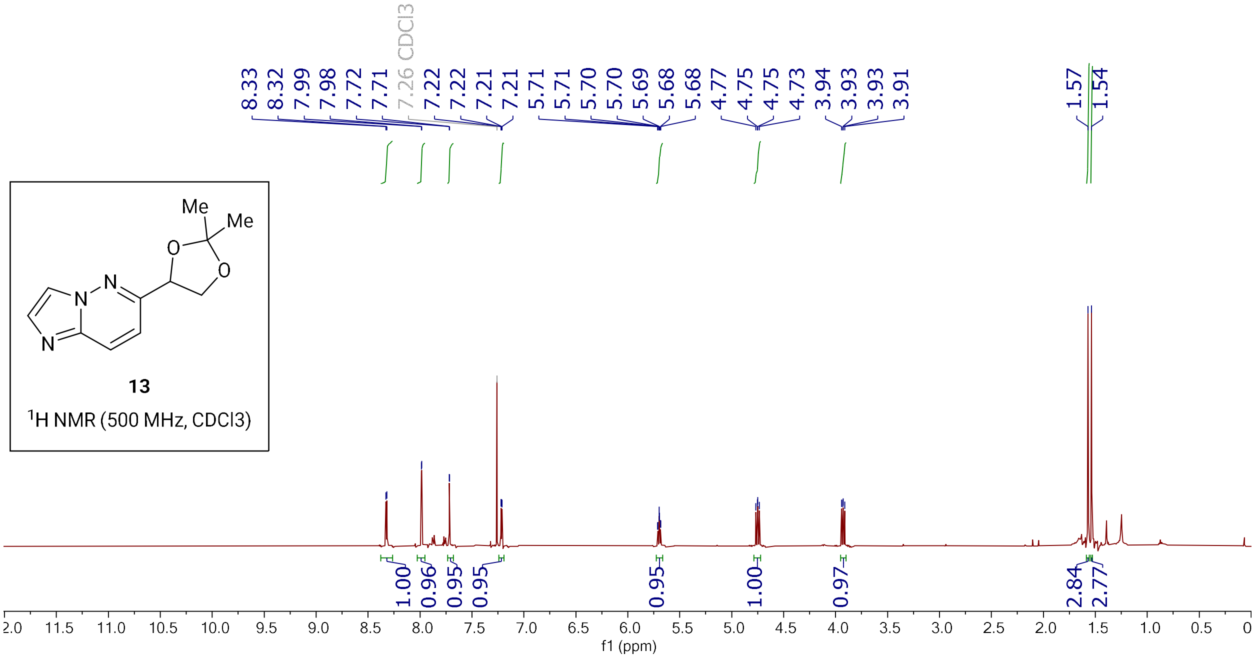

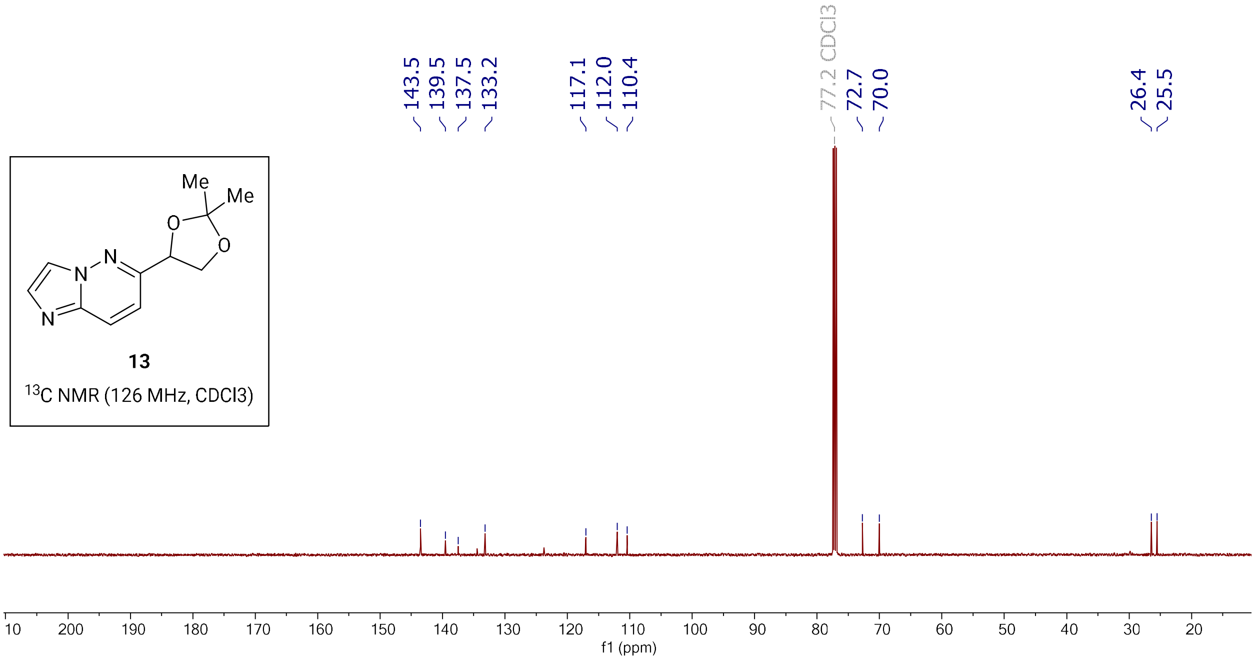

2-(2,2-dimethyl-1,3-dioxolan-4-yl)-4-(trifluoromethyl)thiazole (14)

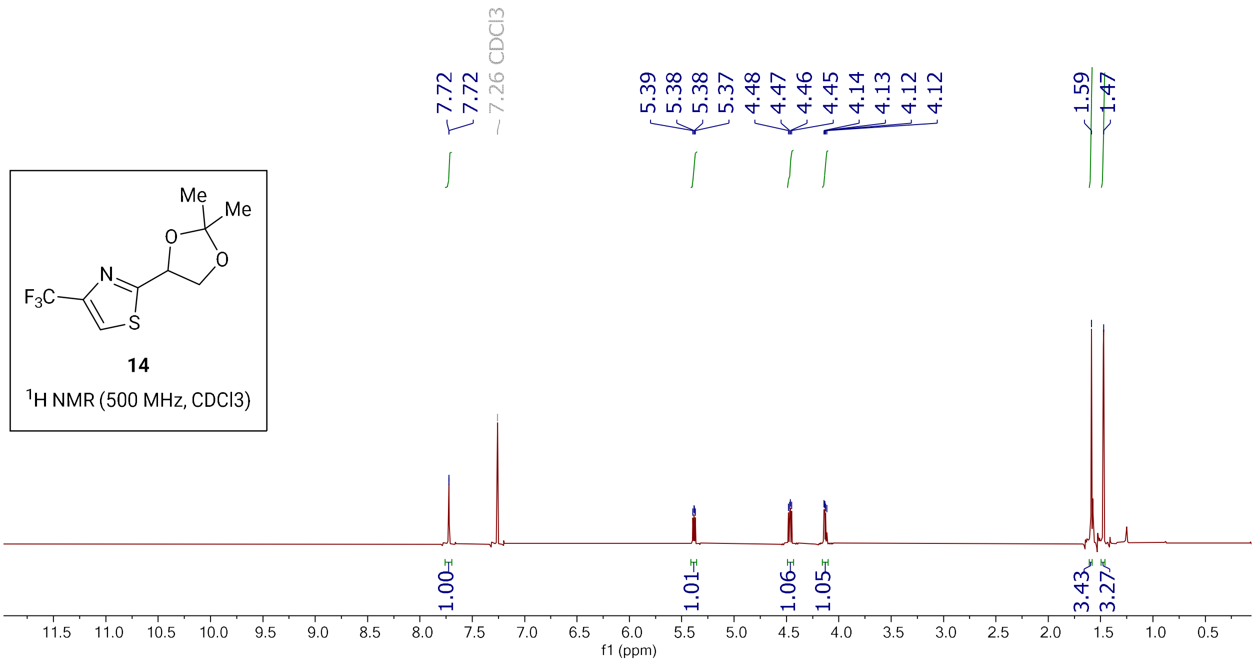

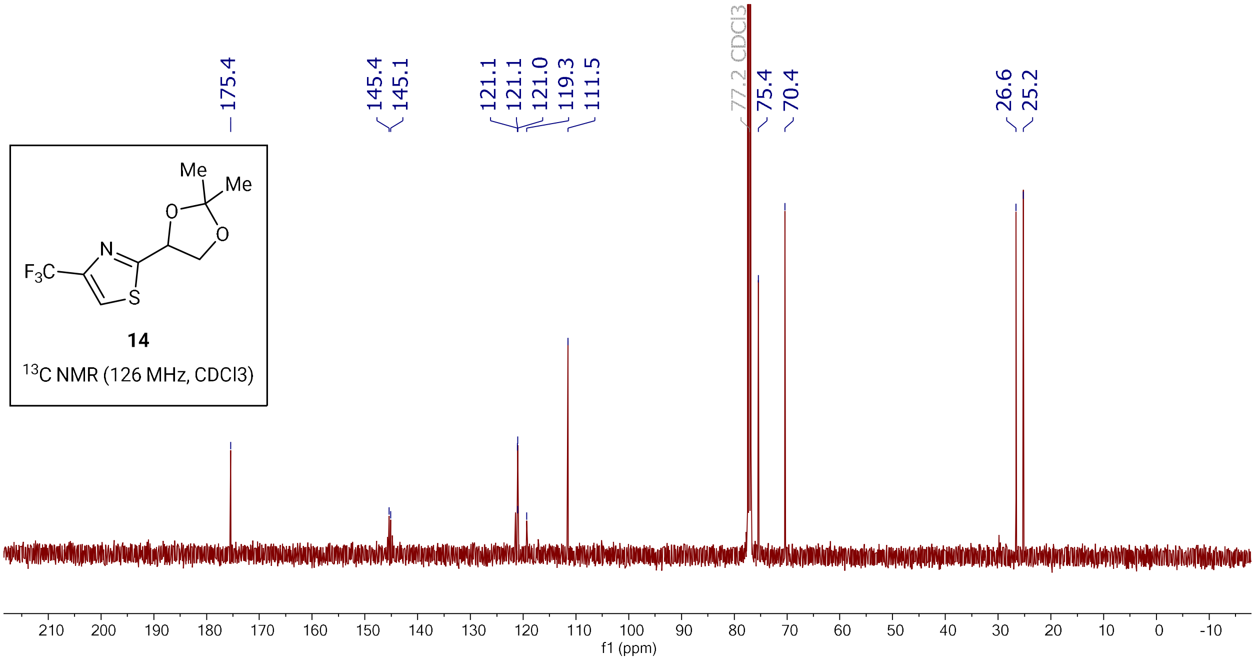

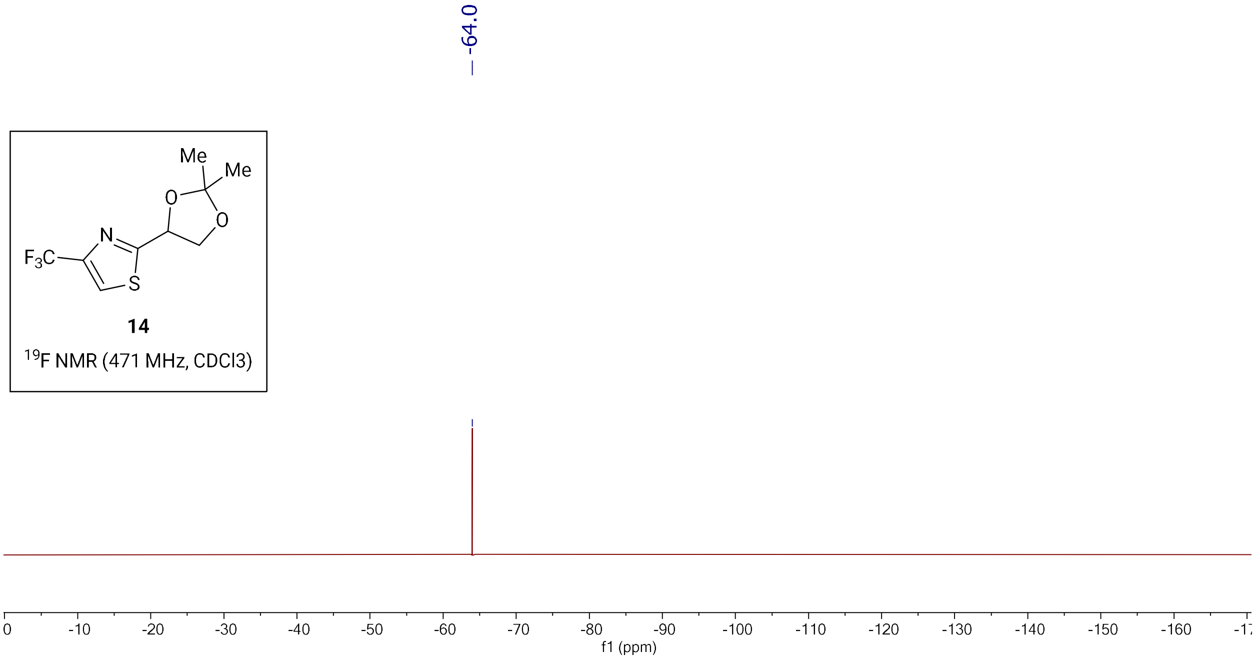
-64.0
Me
Me
O
O
N
F3C
S
14
19F NMR (471 MHz, CDCl3)
0
-10
-20
-30
-40
-50
-60
-70
-80
-90
-100
-110
-120
-130
-140
-150
-160
f1 (ppm)

tert-butyl 5-(2,2-dimethyl-1,3-dioxolan-4-yl)-1H-indole-1-carboxylate (15)

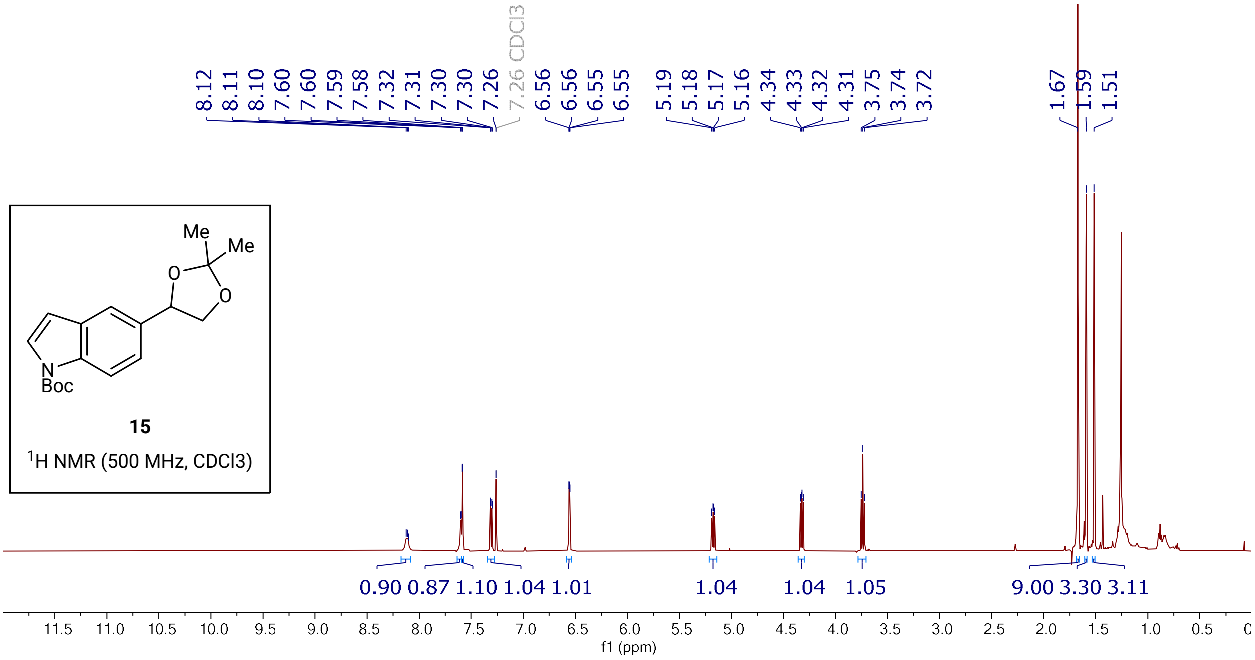

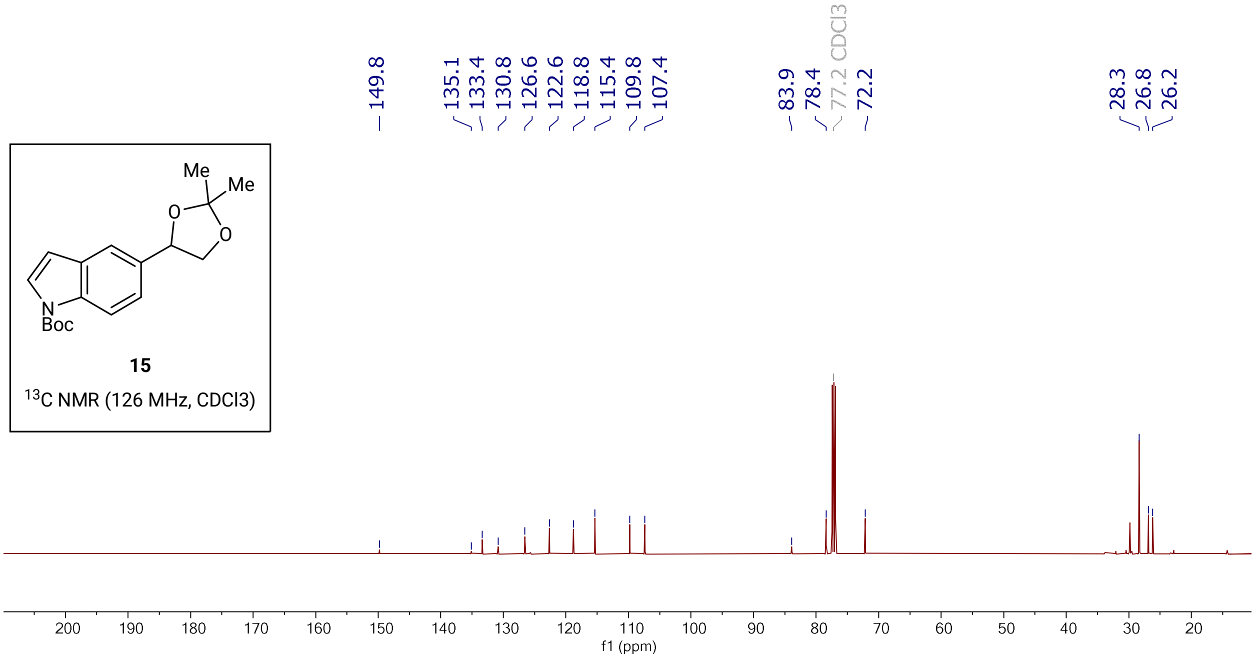

tert-butyl 5-(2,2-dimethyl-1,3-dioxolan-4-yl)-1H-indazole-1-carboxylate (16)

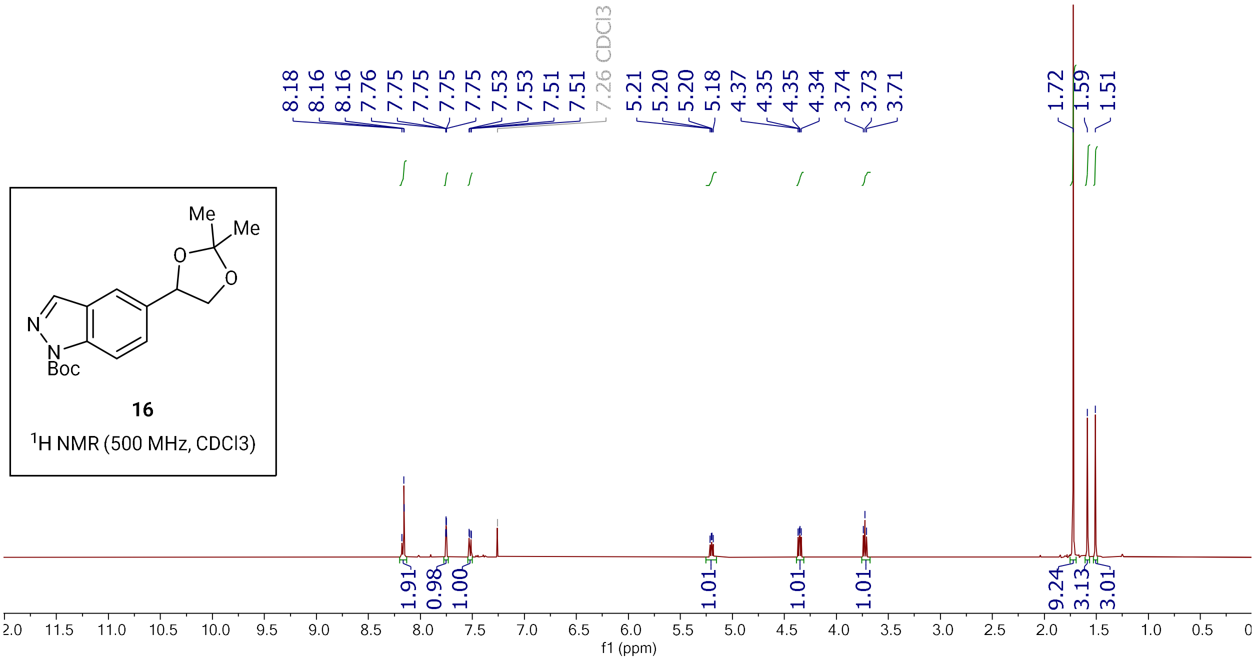

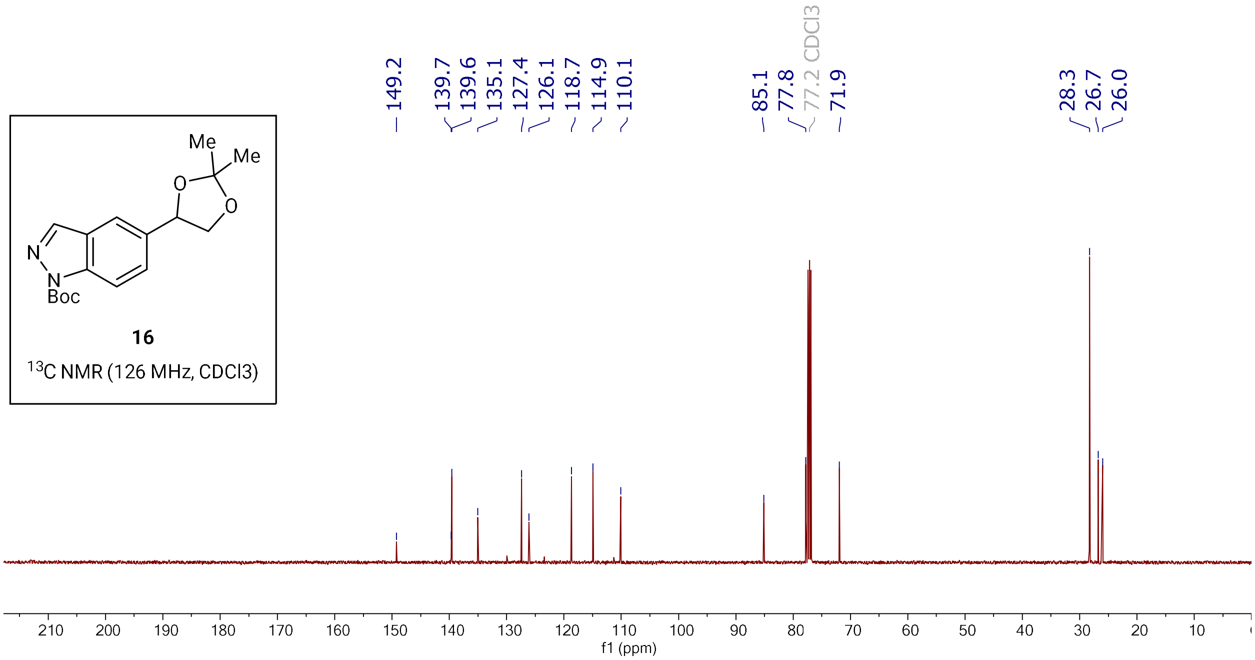

4-(benzo[b]thiophen-5-yl)-2,2-dimethyl-1,3-dioxolane (17)

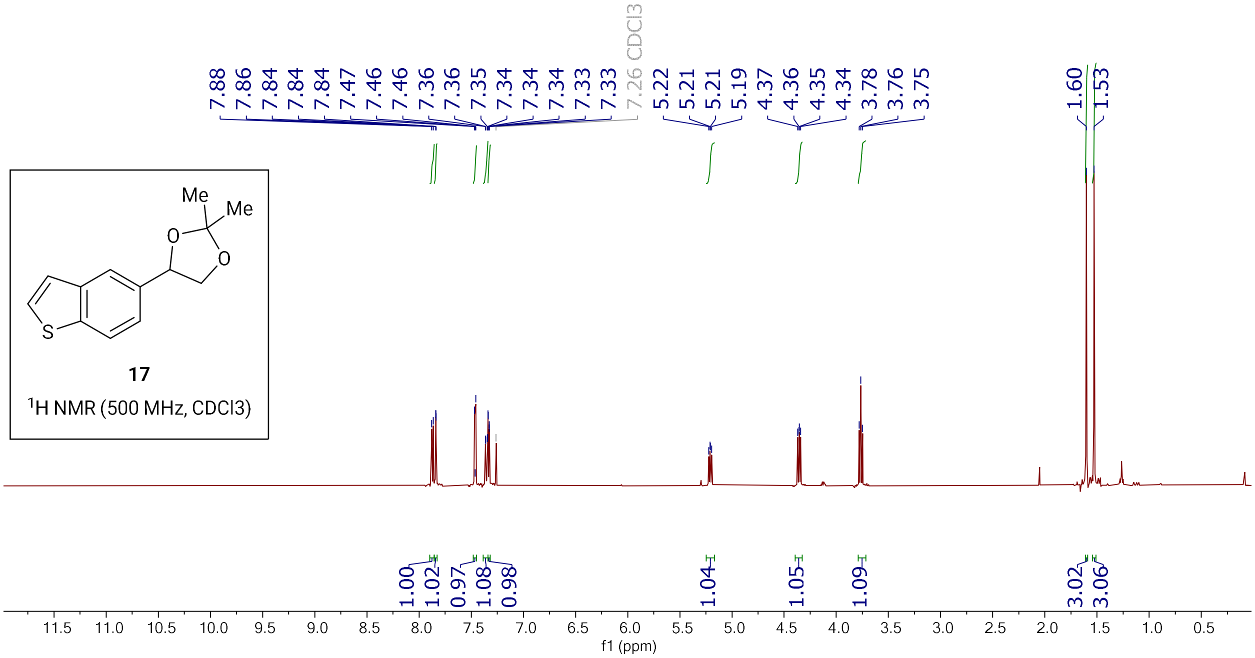

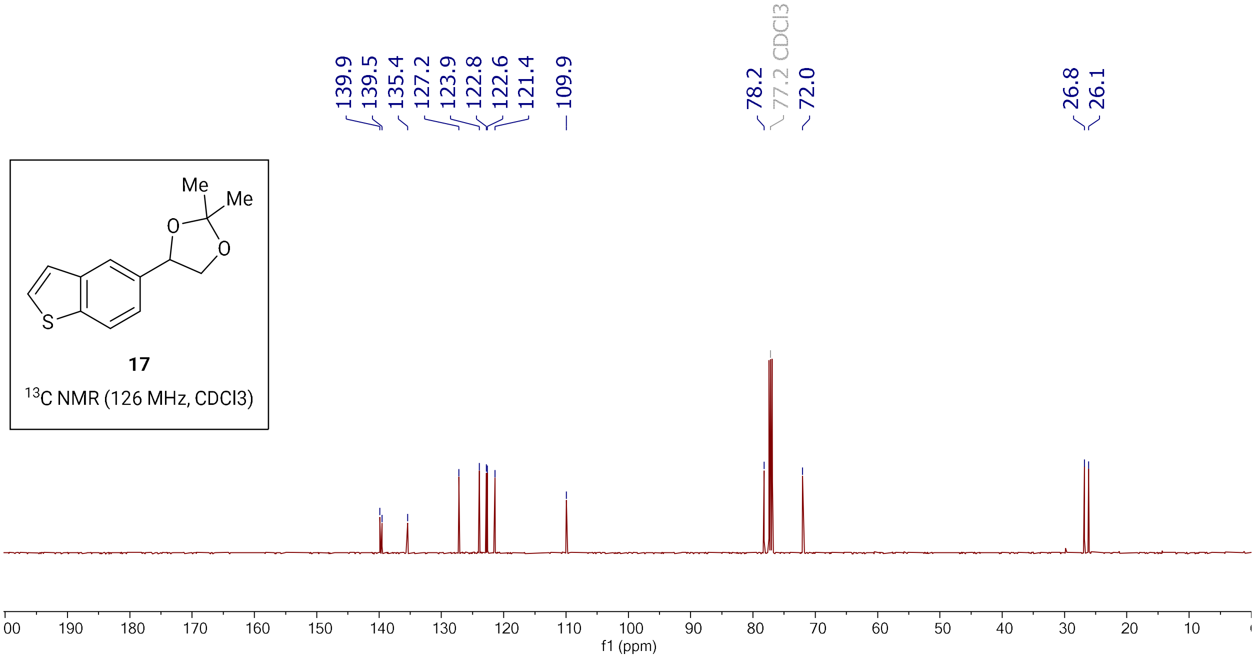

5-(2,2-dimethyl-1,3-dioxolan-4-yl)benzo[d][1,3]dioxole (18)

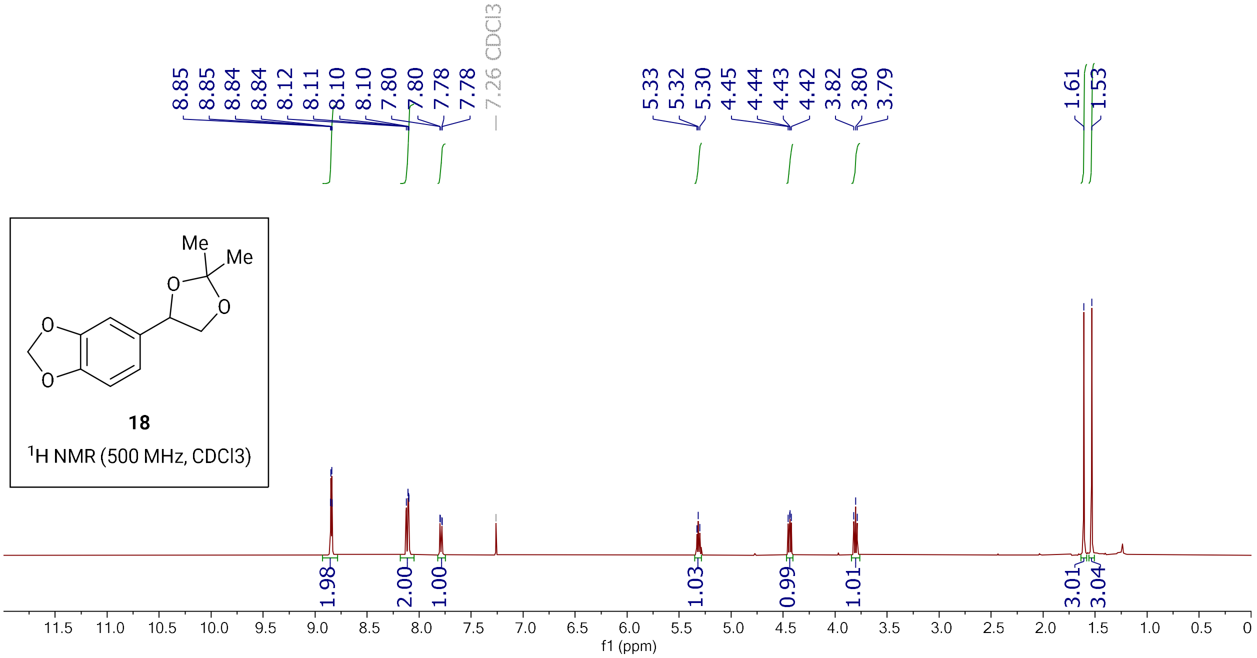

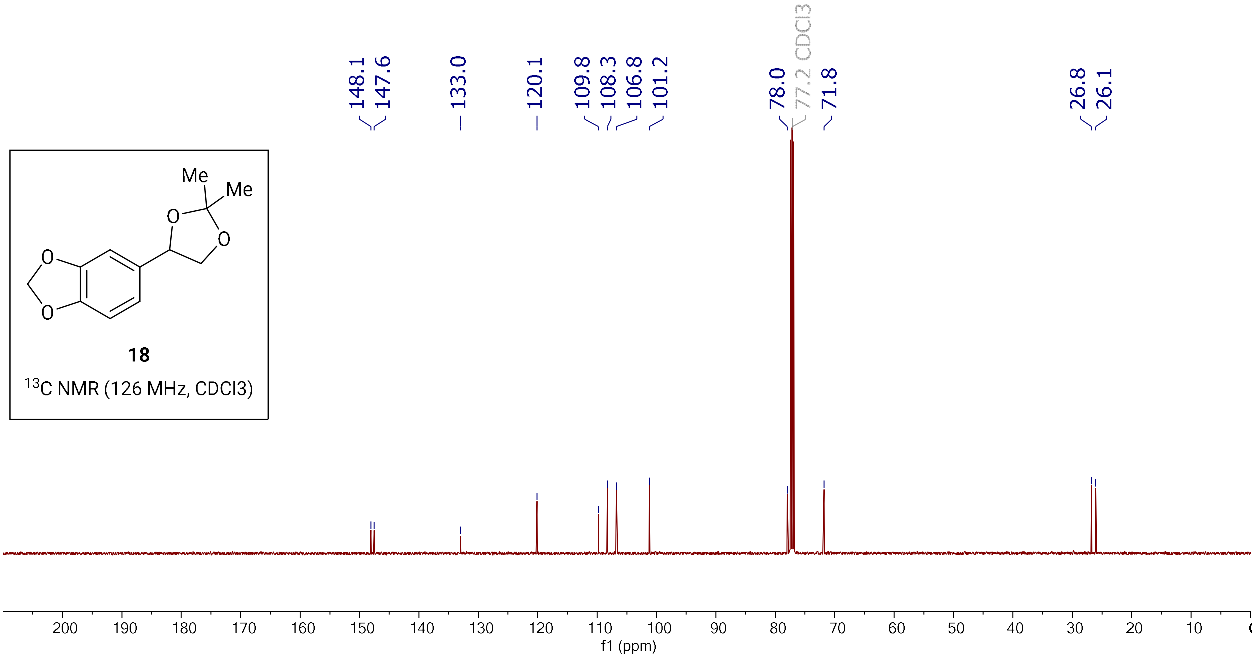

(2R,3R,4R,5R)-2-(6-acetamido-8-(2,2-dimethyl-1,3-dioxolan-4-yl)-9H-purin-9-yl)-5-(acetoxymethyl)tetrahydrofuran-3,4-diyl diacetate (19)

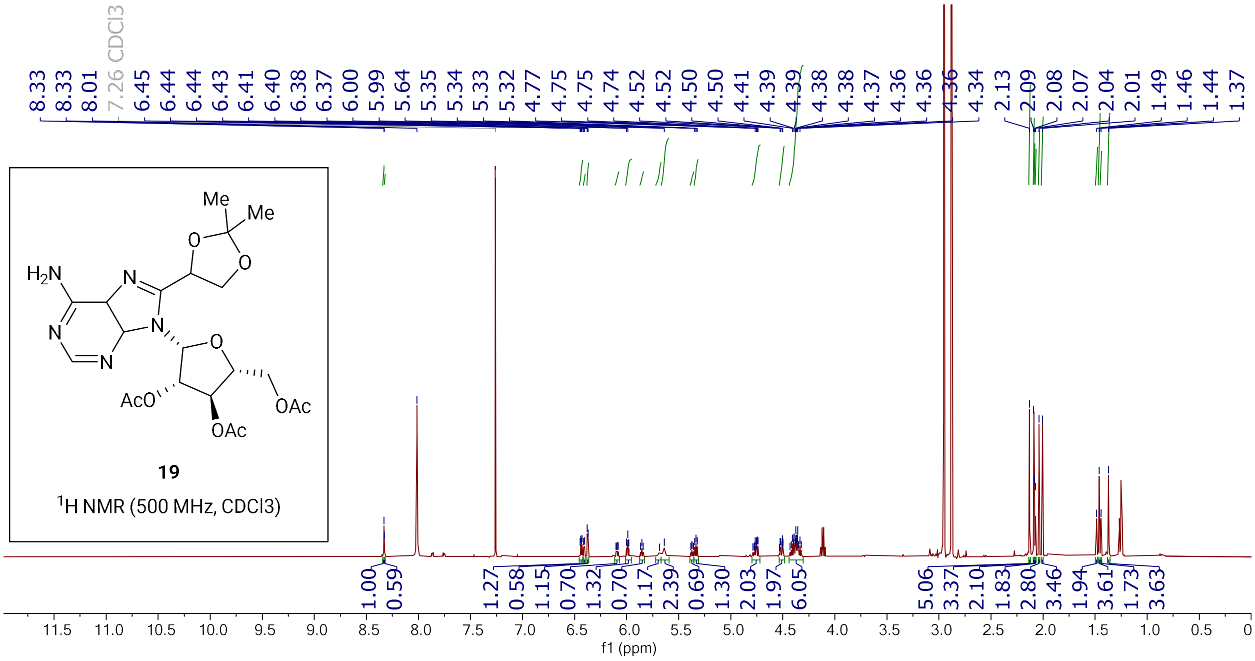

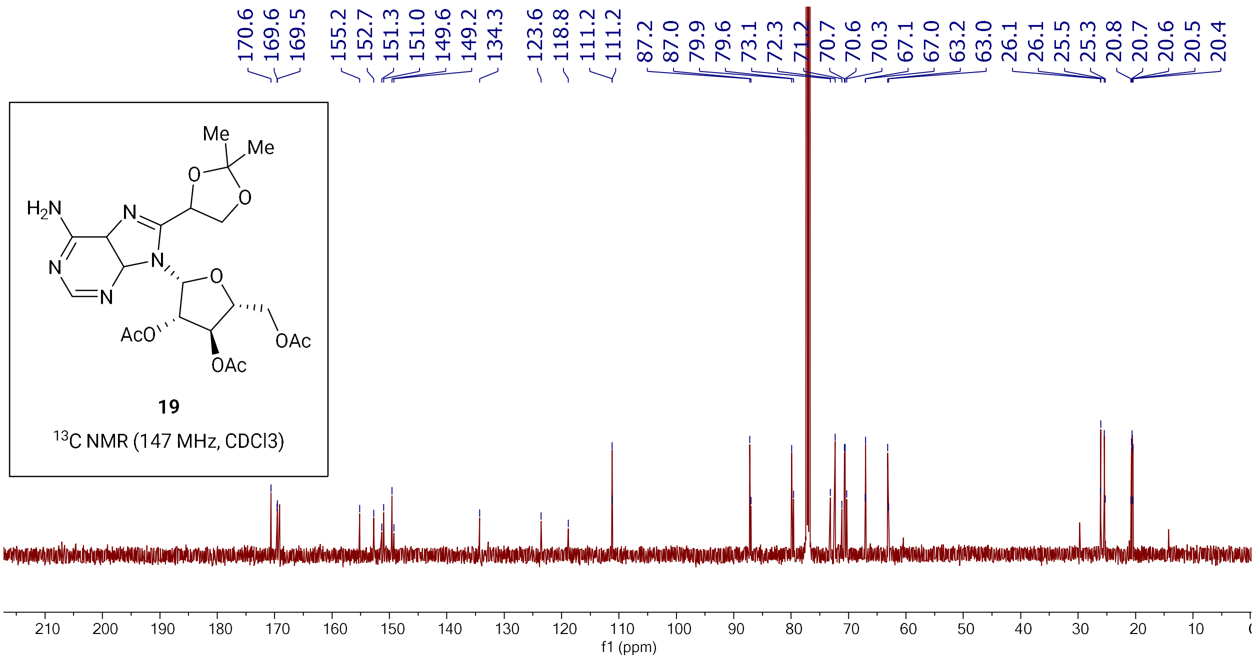

1-(5-(trifluoromethyl)pyridin-2-yl)ethane-1,2-diol (20)

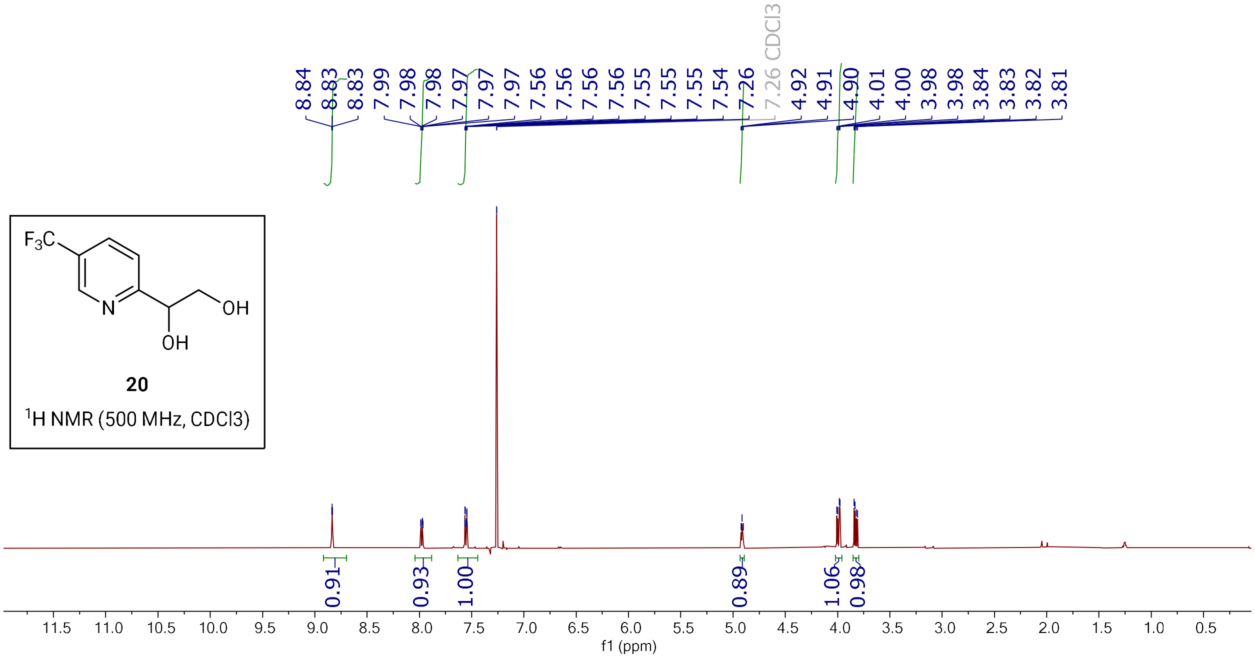

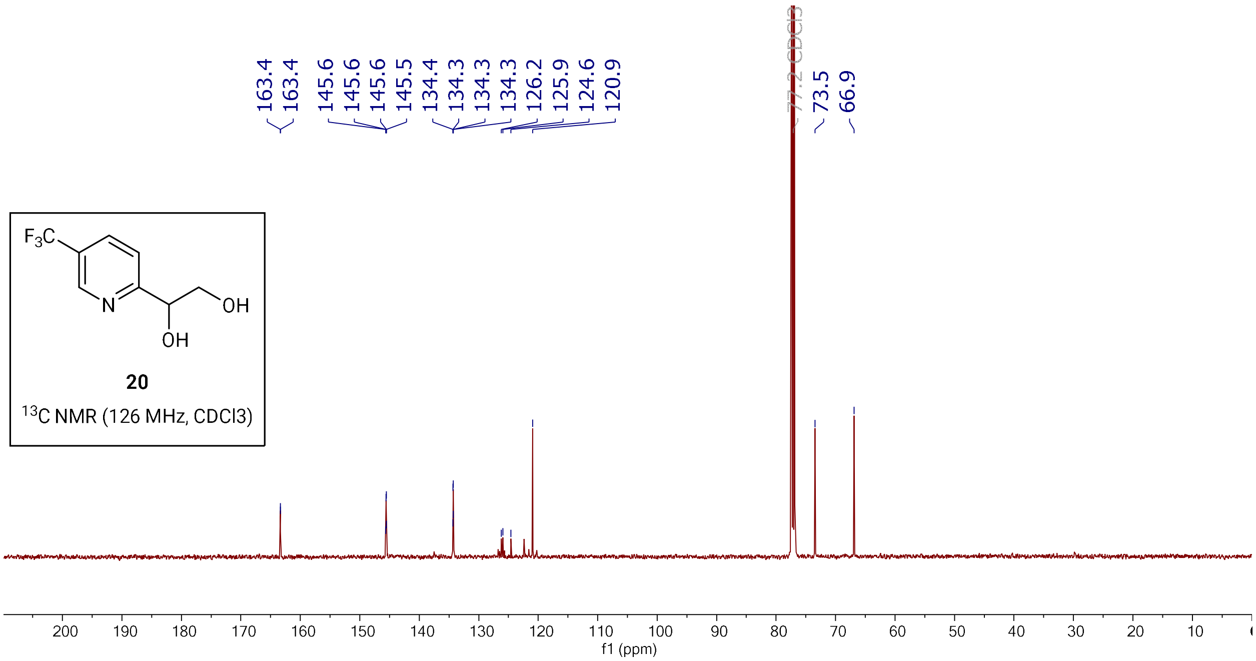

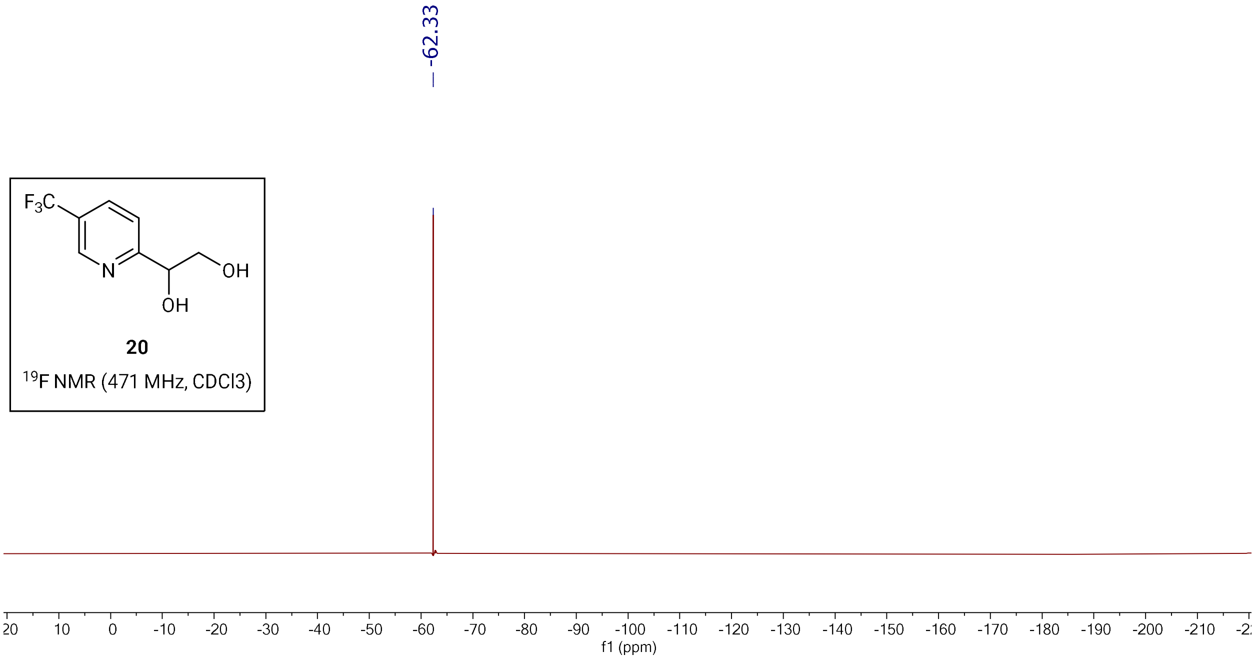

-62.33
F3C
N
OH
OH
20
19F NMR (471 MHz, CDCl3)
20
10
0
-10
-20
-30
-40
-50
-60
-70
-80
-90
-100
-110
-120
-130
-140
-150
-160
-170
-180
-190
-200
-210
f1 (ppm)

2-morpholino-1-(5-(trifluoromethyl)pyridin-2-yl)ethan-1-ol (21)

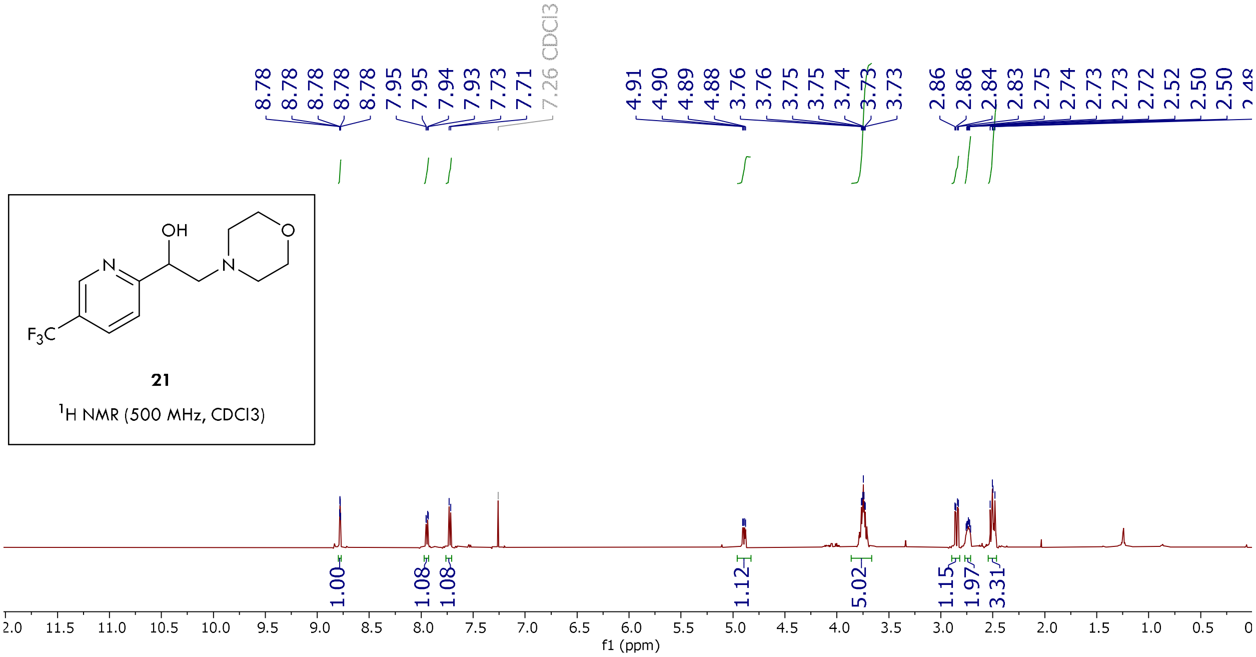

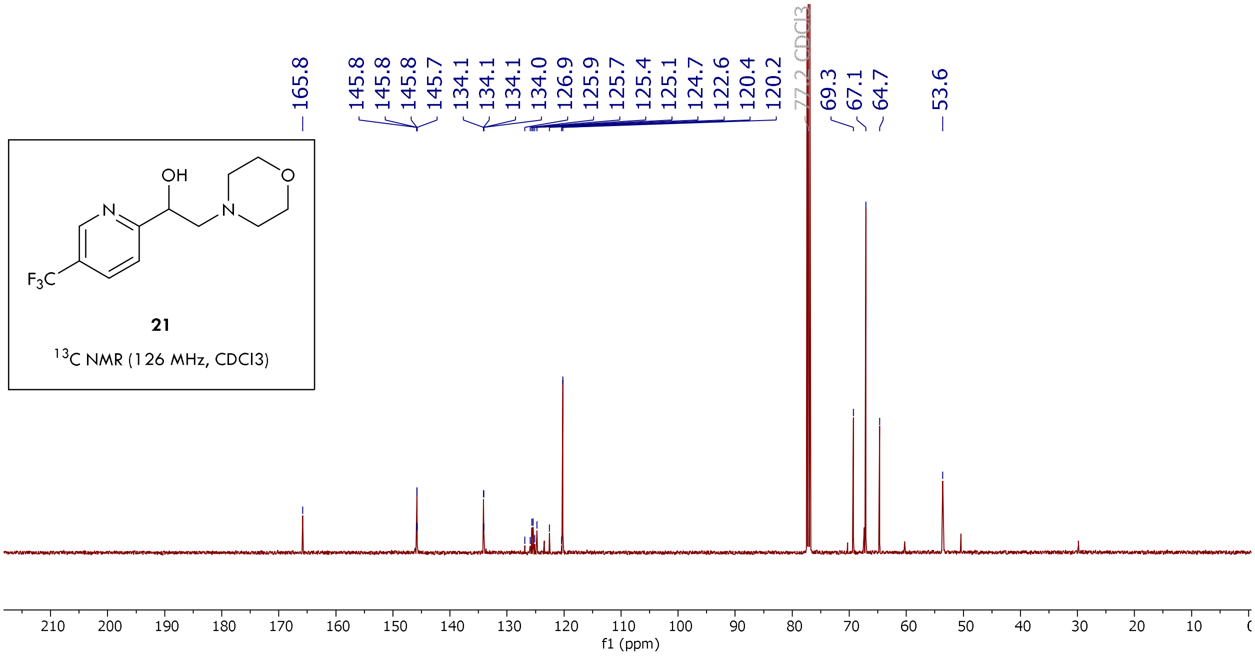

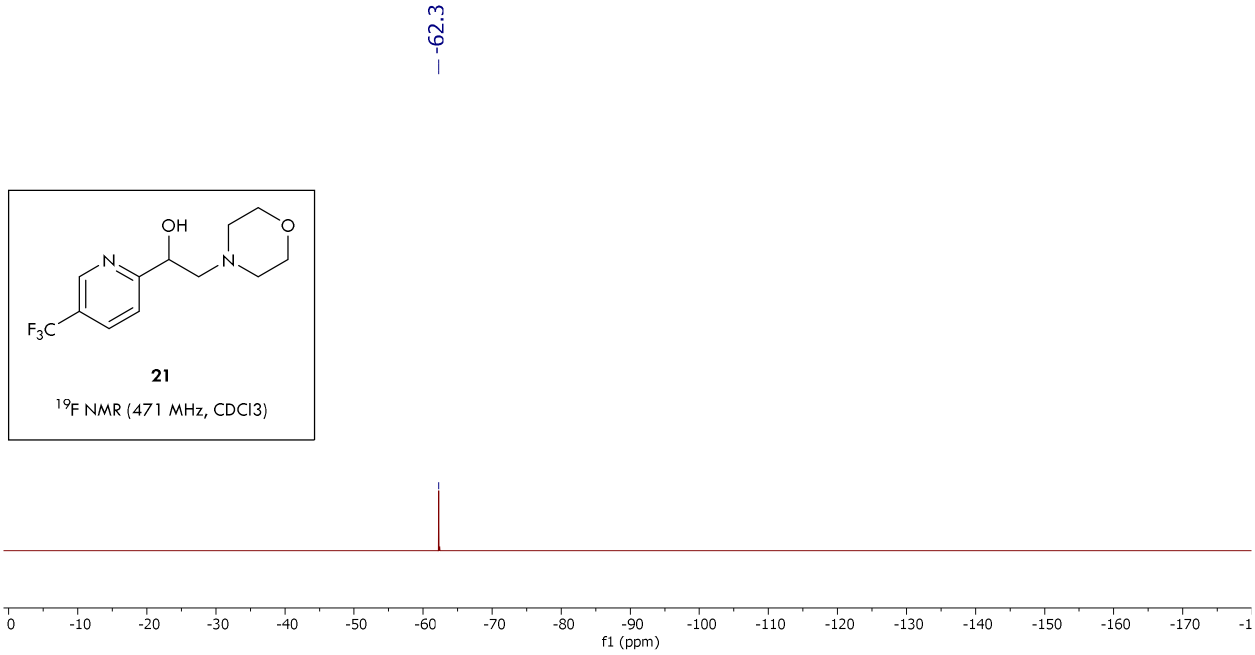
-62.3
OH
N
F3C
21
19F NMR (471 MHz, CDCl3)
0
-10
-20
-30
-40
-50
-60
-70
-80
-90
-100
-110
-120
-130
-140
-150
-160
-170
f1 (ppm)

2-((S)-2-benzylaziridin-1-yl)-1-(5-(trifluoromethyl)pyridin-2-yl)ethan-1-ol (22)

1H NMR (500 MHz, $CDCl_3$) δ 8.67 (d, J = 2.4 Hz, 1H), 8.64 (d, J = 2.2 Hz, 1H), 7.87 (ddd, J = 7.8, 4.9, 2.3 Hz, 2H), 7.70 (d, J = 7.9 Hz, 1H), 7.61 (d, J = 8.1 Hz, 1H), 7.56 (d, J = 8.2 Hz, 1H), 7.25 – 7.18 (m, 3H), 7.16 – 7.06 (m, 3H), 4.99 (td, J = 7.5, 3.8 Hz, 2H), 3.69 (dd, J = 11.4, 3.5 Hz, 2H), 3.48 (td, J = 11.4, 6.1 Hz, 2H), 3.34 – 3.20 (m, 2H), 3.06 (ddd, J = 7.3, 5.4, 2.3 Hz, 2H), 3.03 – 2.95 (m, 2H), 2.85 (ddd, J = 13.6, 9.2, 6.5 Hz, 2H), 2.75 (dt, J = 13.7, 6.8 Hz, 2H).

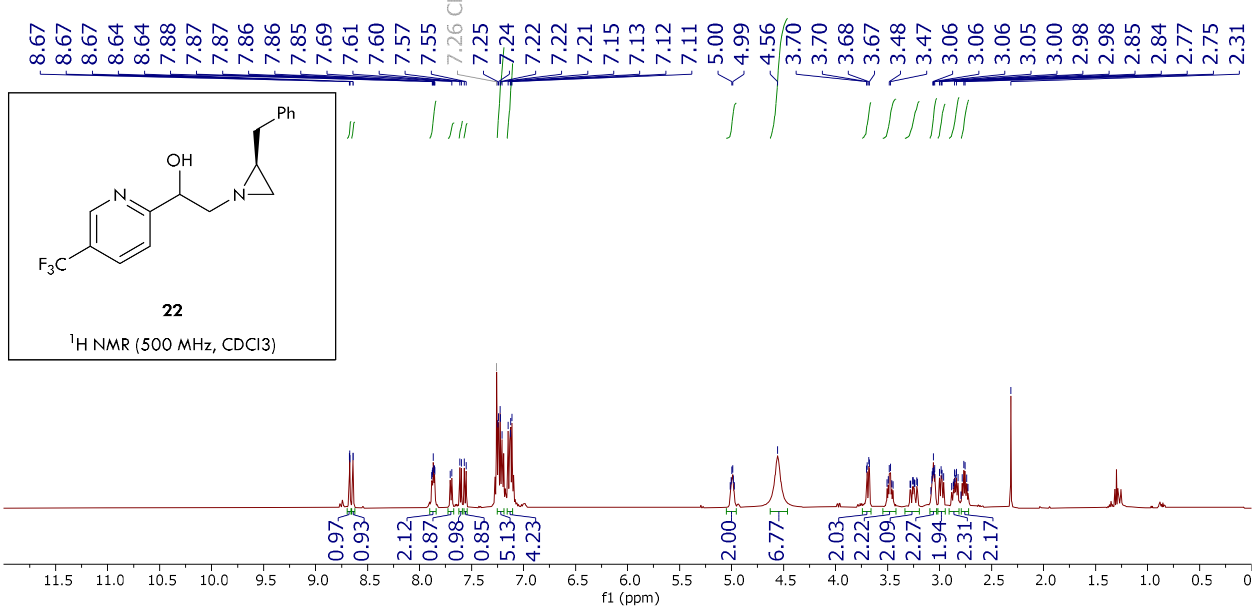

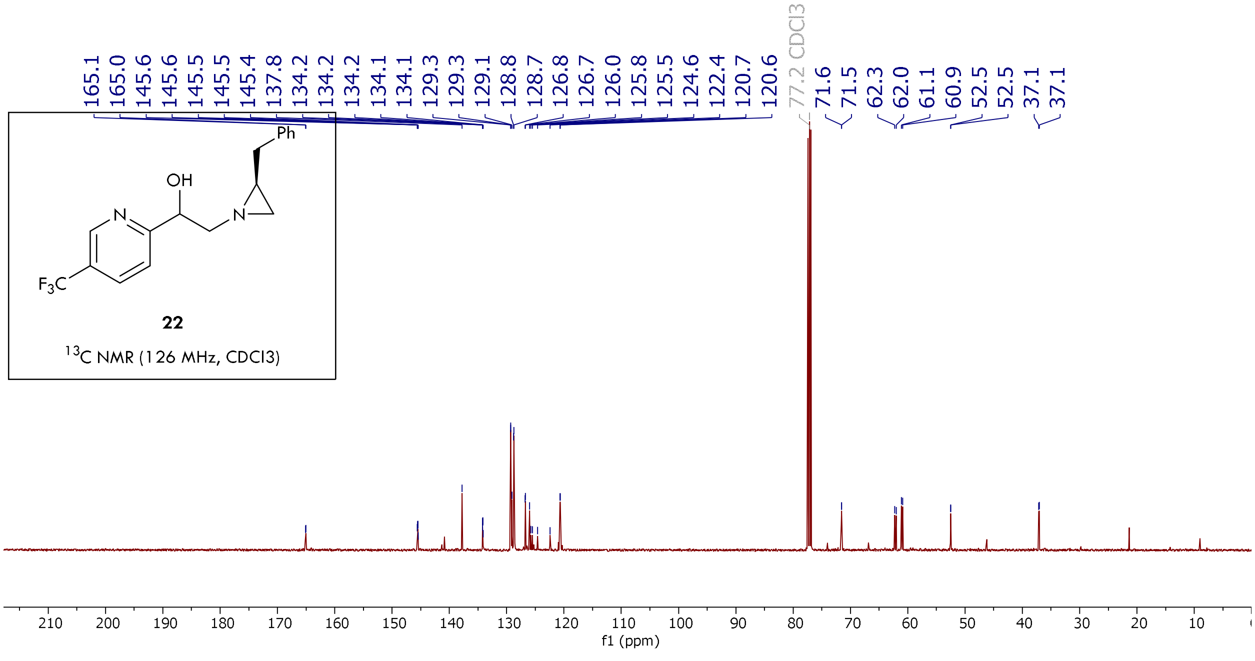

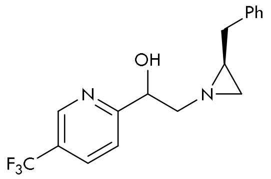

22

^{19}F NMR (471 MHz, CDCl3)

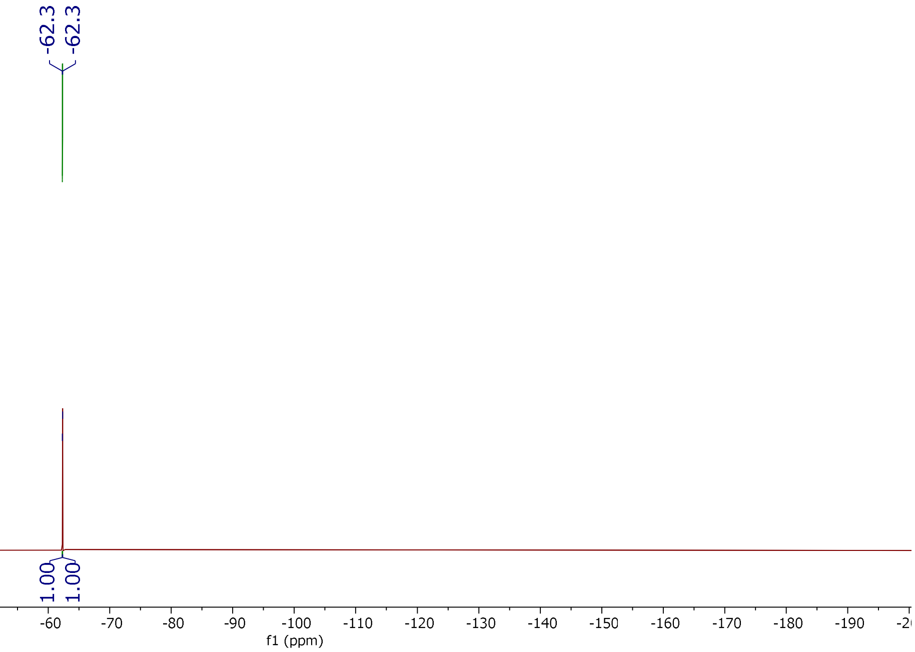

2-(2-azaspiro[3.3]heptan-2-yl)-1-(5-(trifluoromethyl)pyridin-2-yl)ethan-1-ol (23)

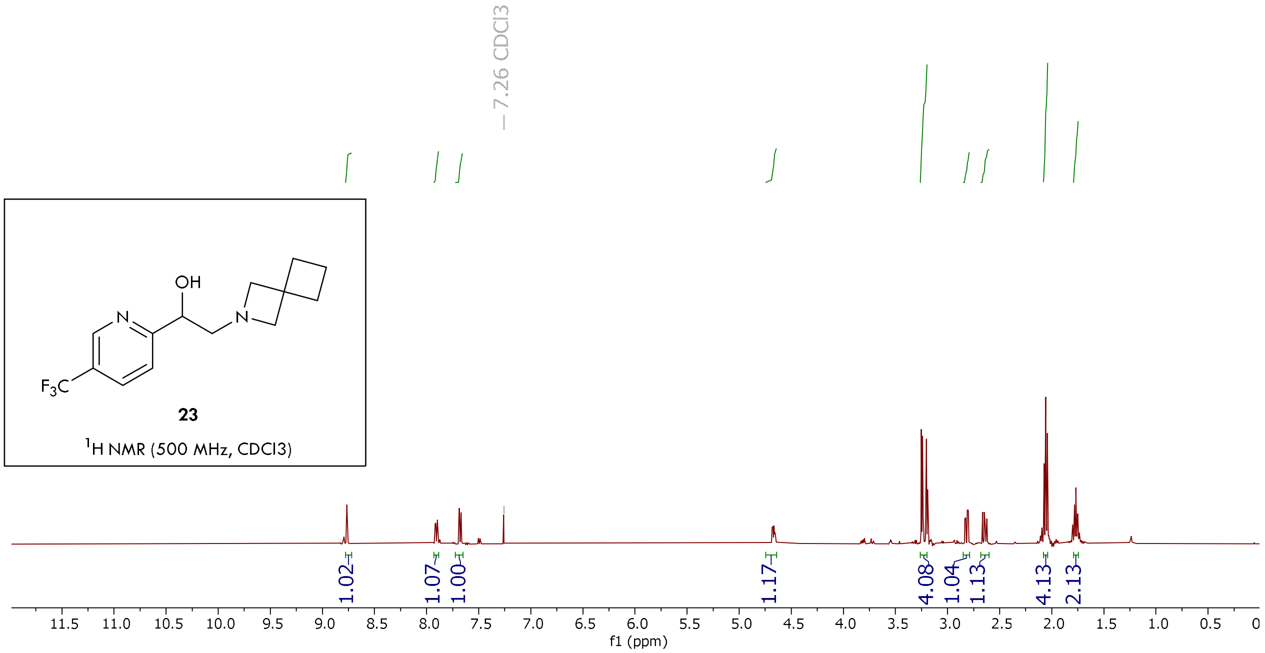

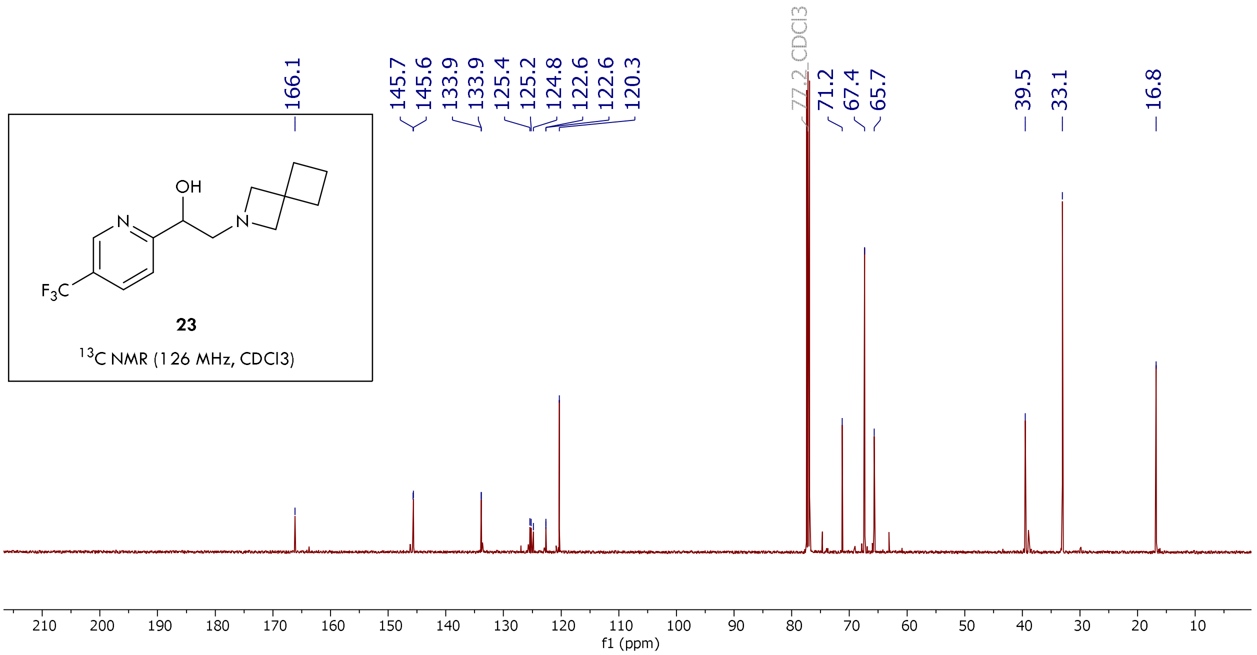

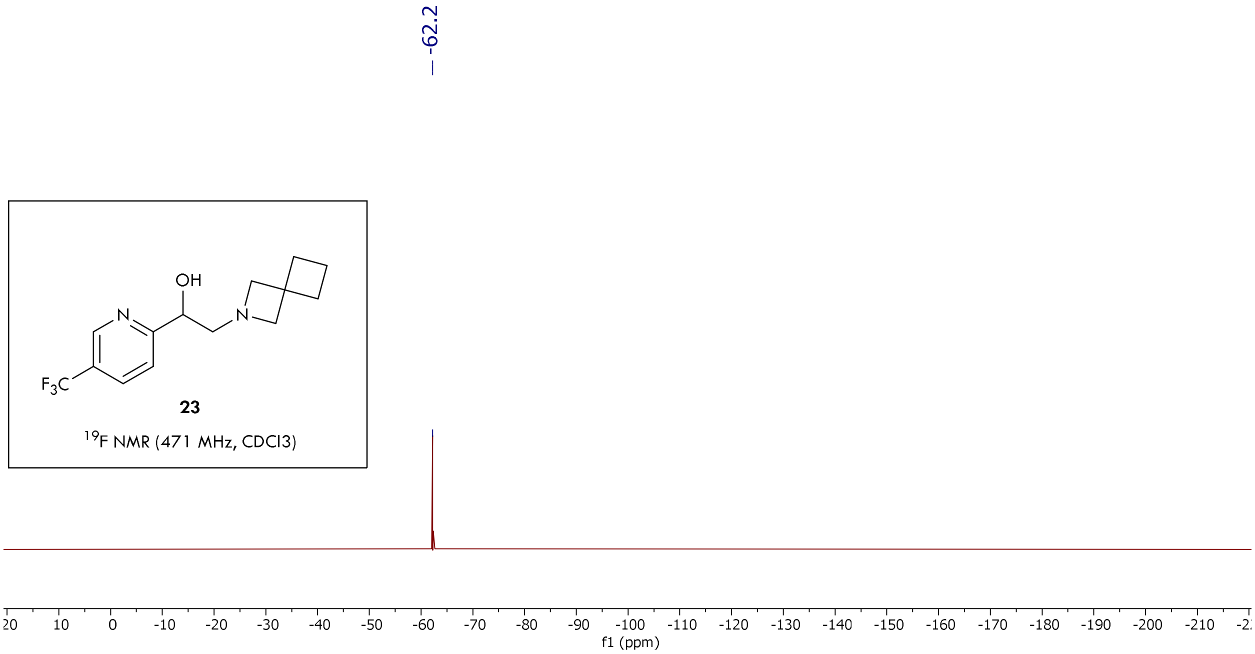
-62.2
OH
N
N
F3C
23
19F NMR (471 MHz, CDCl3)
20
10
0
-10
-20
-30
-40
-50
-60
-70
-80
-90
-100
-110
-120
-130
-140
-150
-160
-170
-180
-190
-200
-210
f1 (ppm)

2-(pyrrolidin-1-yl)-1-(5-(trifluoromethyl)pyridin-2-yl)ethan-1-ol (24)

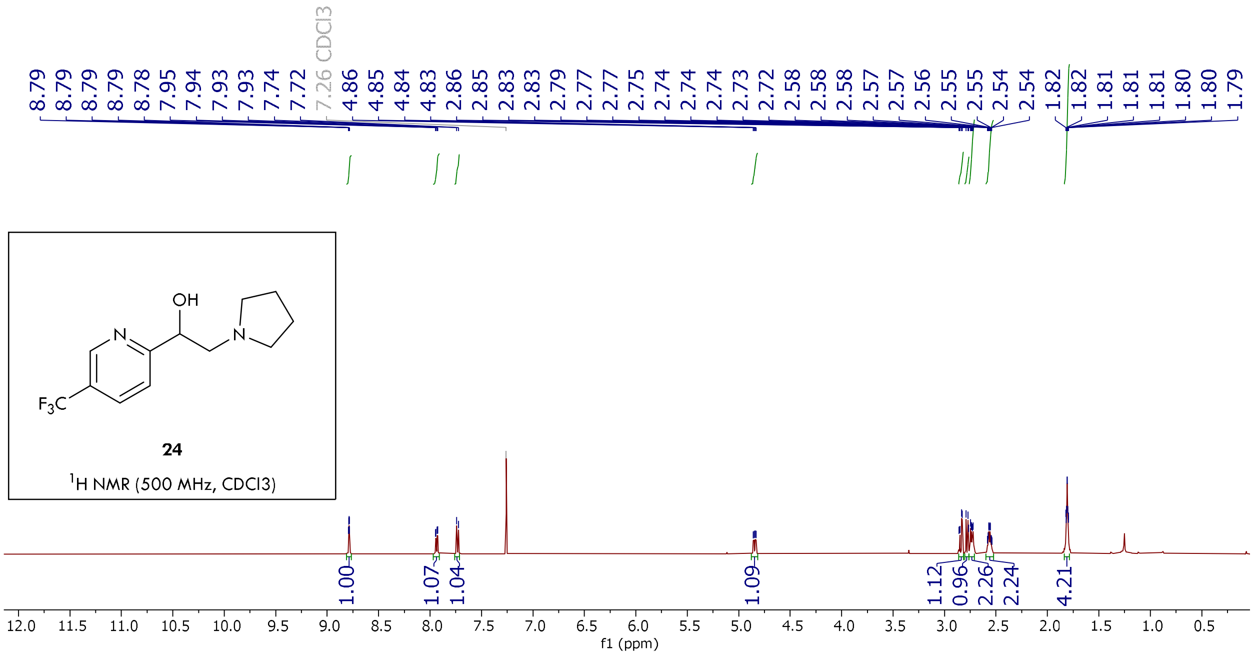

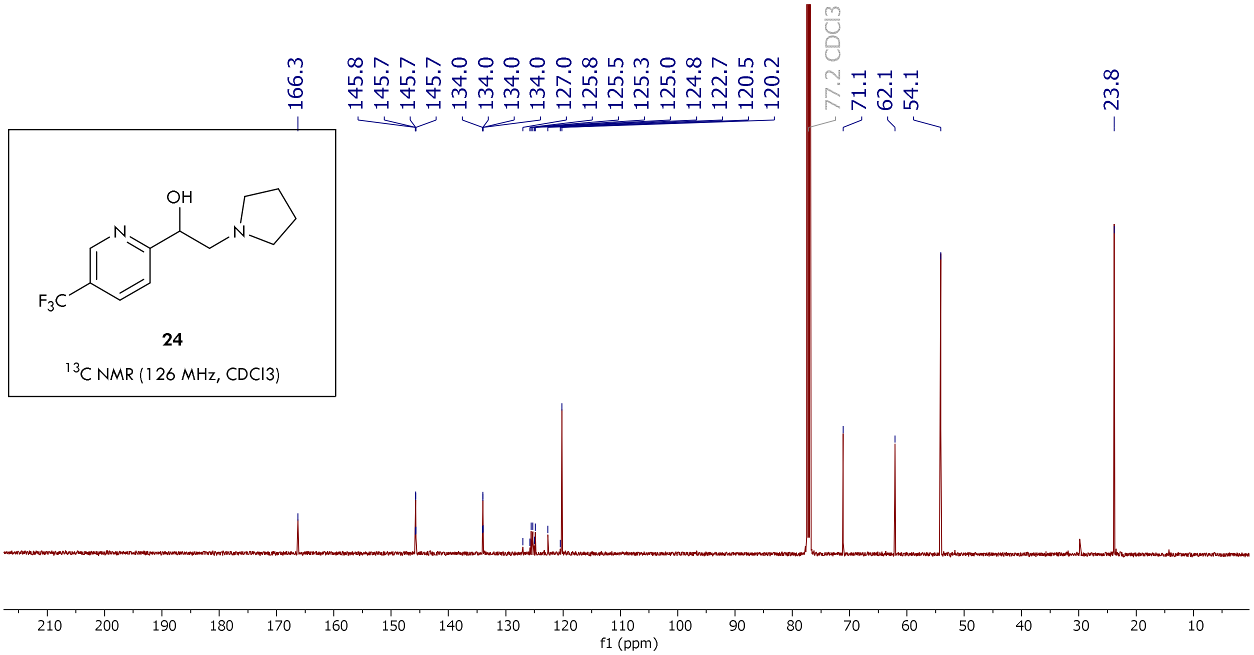

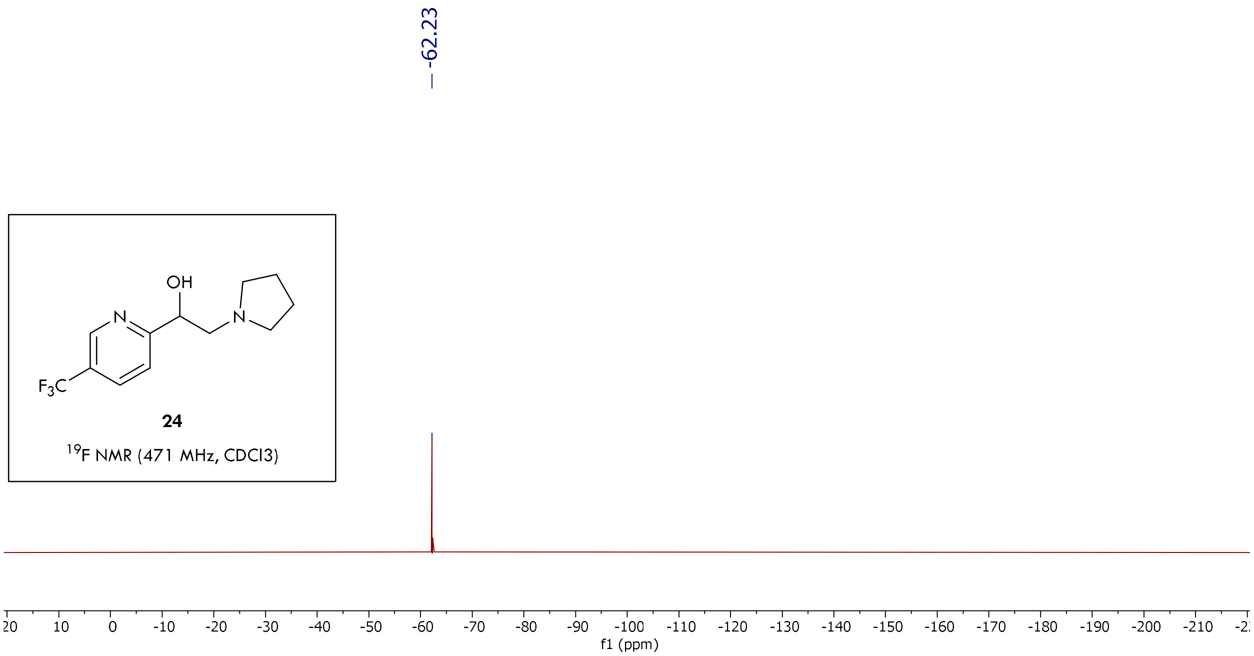
-62.23
OH
N
N
F3C
24
19F NMR (471 MHz, CDCl3)
20 10 0 -10 -20 -30 -40 -50 -60 -70 -80 -90 -100 -110 -120 -130 -140 -150 -160 -170 -180 -190 -200 -210
f1 (ppm)

2-(piperidin-1-yl)-1-(5-(trifluoromethyl)pyridin-2-yl)ethan-1-ol (25)

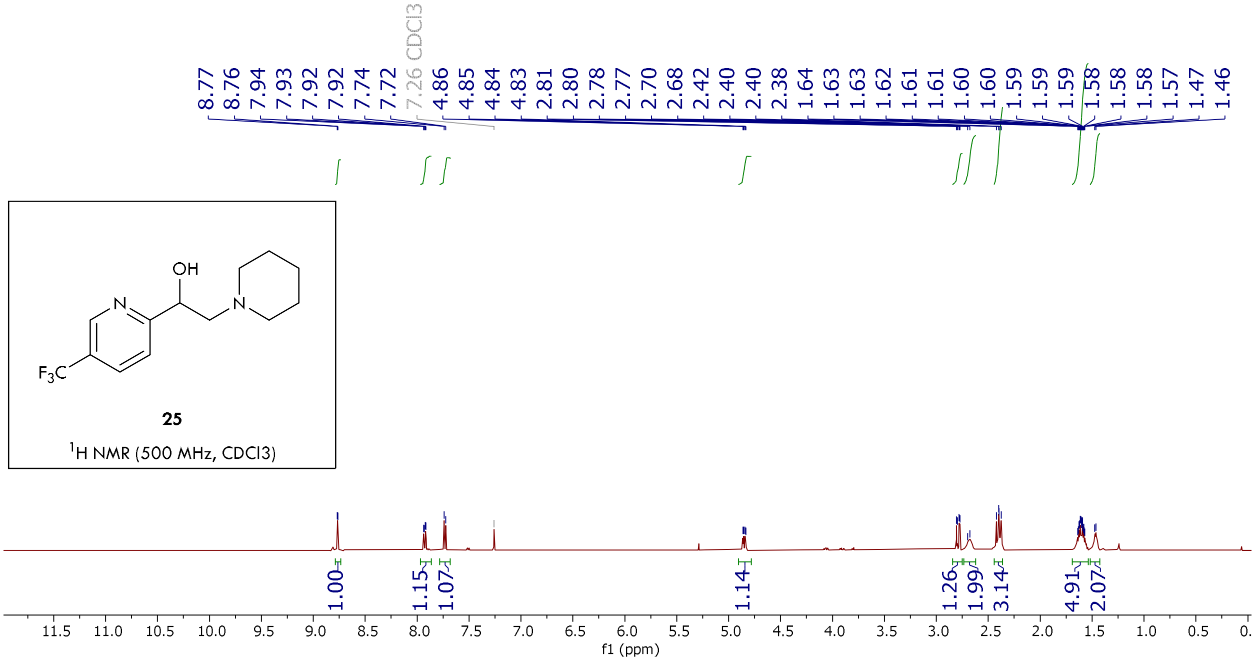

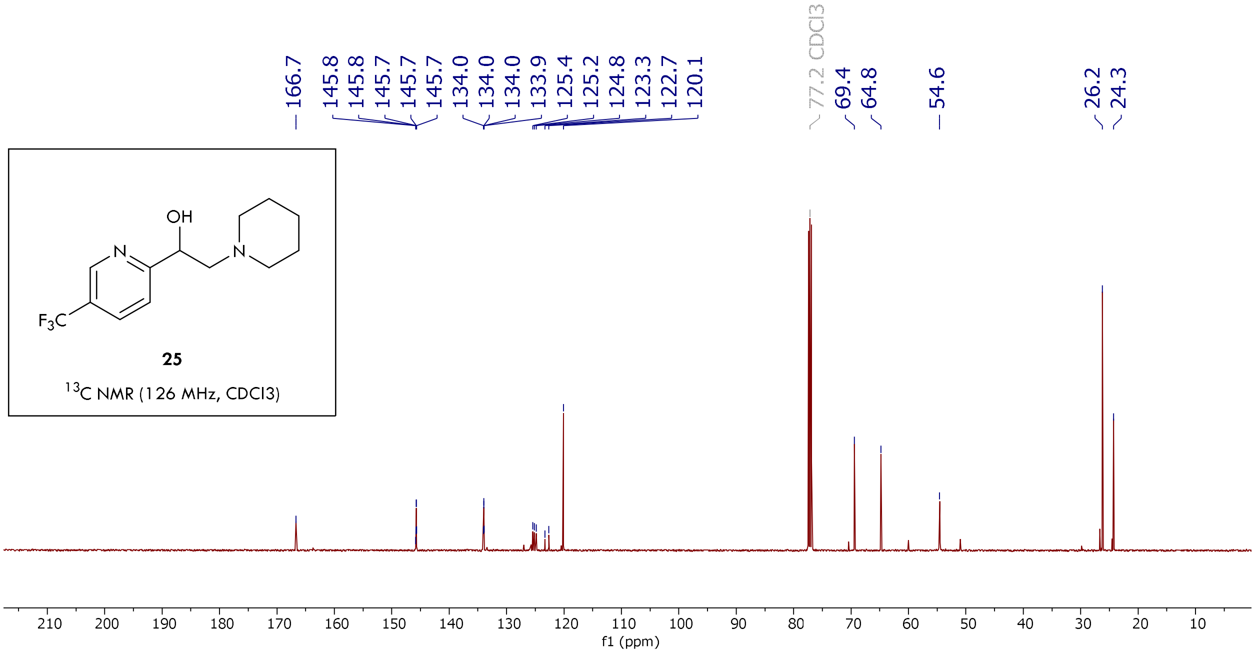

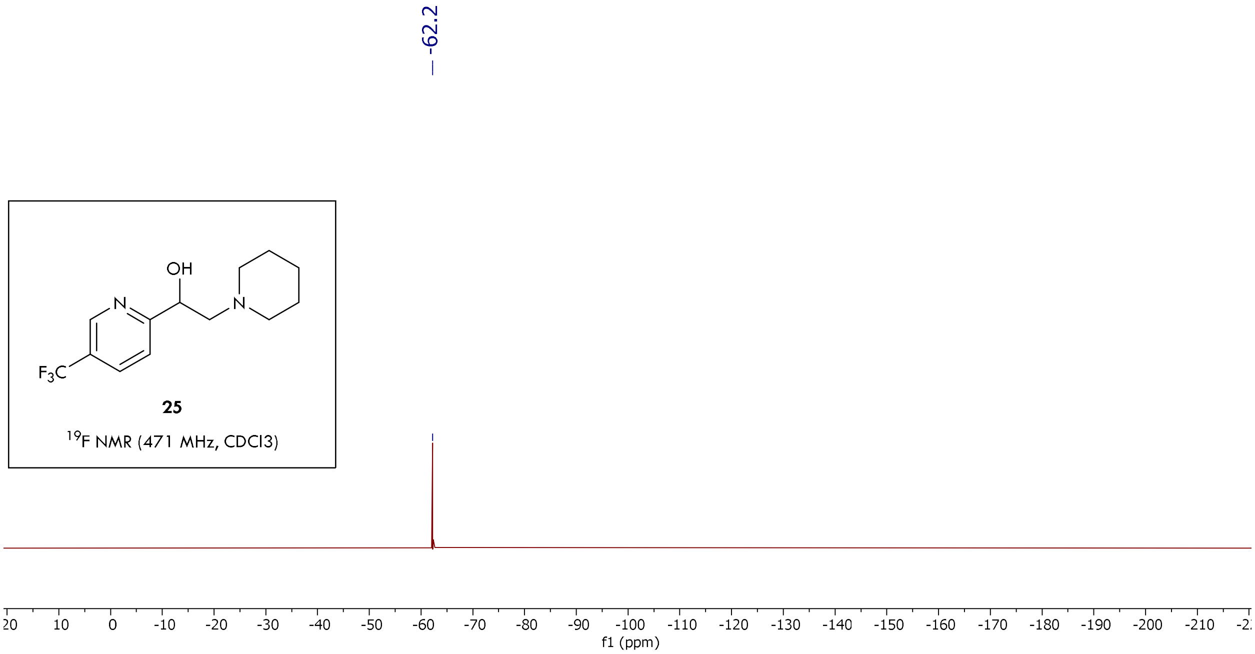
-62.2
OH
N
N
F3C
25
19F NMR (471 MHz, CDCl3)
20 10 0 -10 -20 -30 -40 -50 -60 -70 -80 -90 -100 -110 -120 -130 -140 -150 -160 -170 -180 -190 -200 -210
f1 (ppm)

tert-butyl 4-(2-hydroxy-2-(5-(trifluoromethyl)pyridin-2-yl)ethyl)piperazine-1-carboxylate (26)

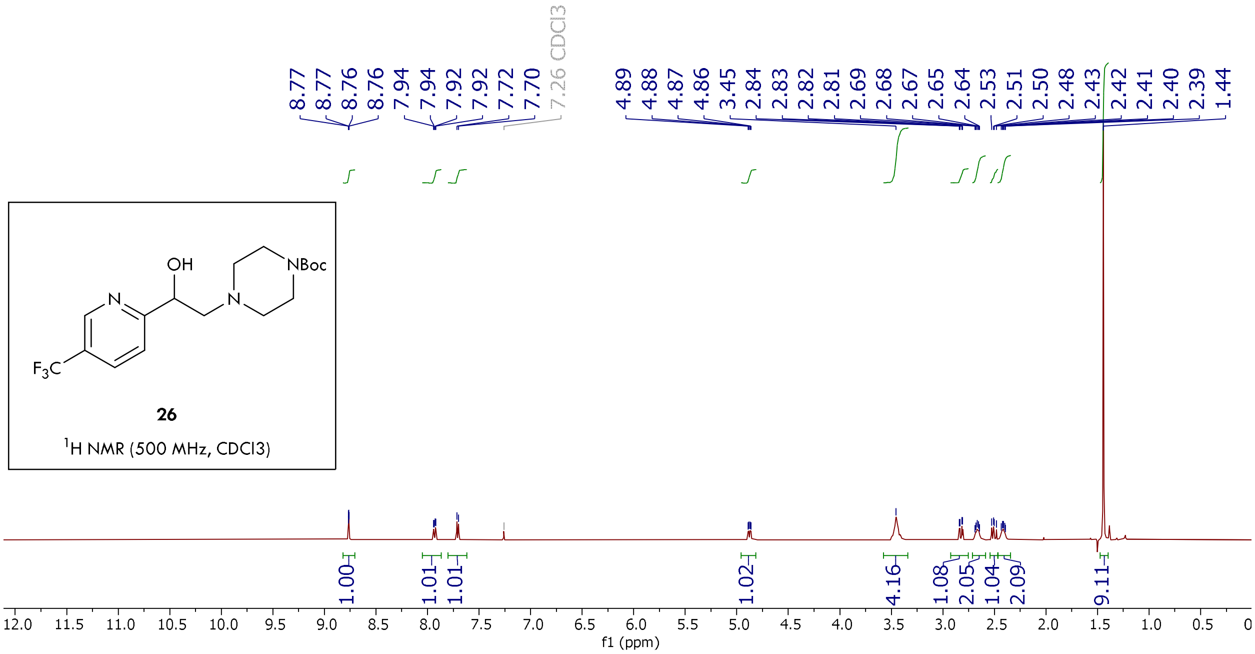

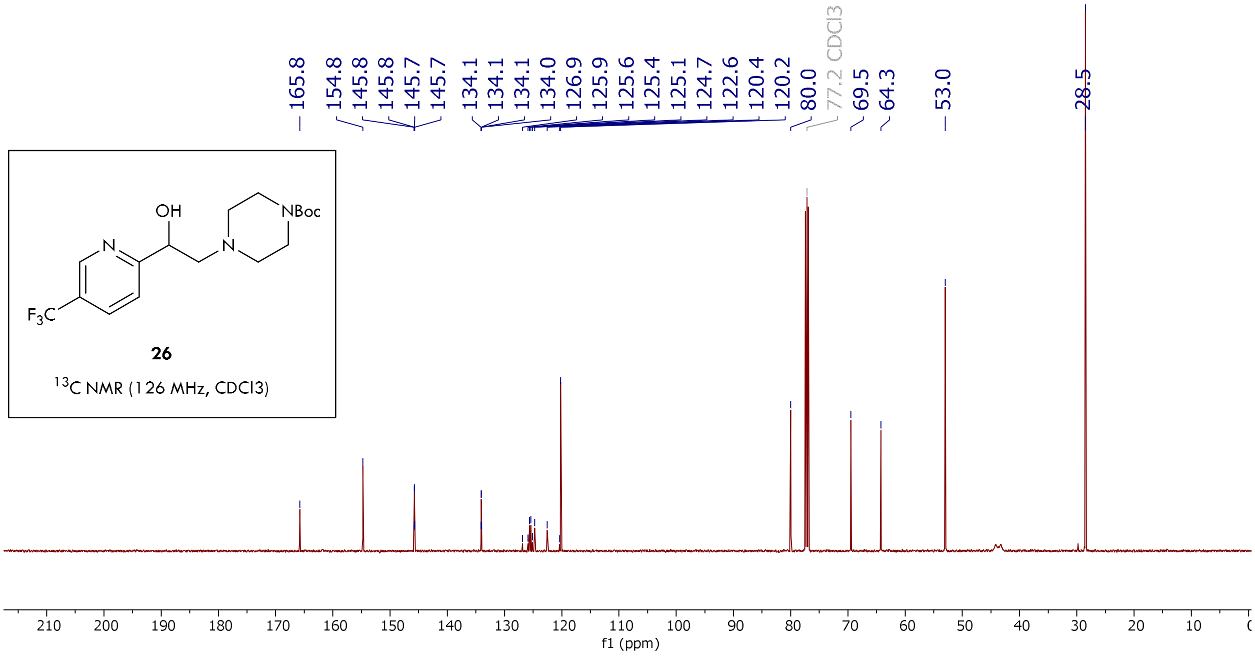

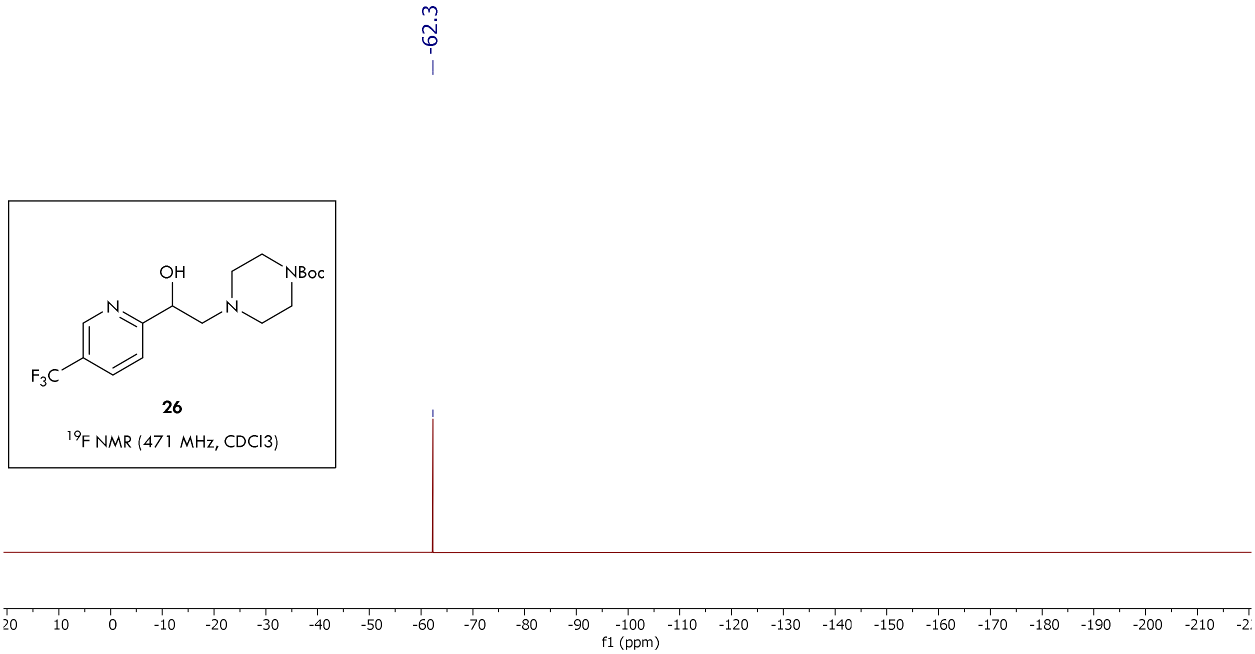
-62.3
OH
NBoc
N
N
F3C
26
19F NMR (471 MHz, CDCl3)
20
10
0
-10
-20
-30
-40
-50
-60
-70
-80
-90
-100
-110
-120
-130
-140
-150
-160
-170
-180
-190
-200
-210
f1 (ppm)

2-(((3s,5s,7s)-adamantan-1-yl)amino)-1-(5-(trifluoromethyl)pyridin-2-yl)ethan-1-ol (27)

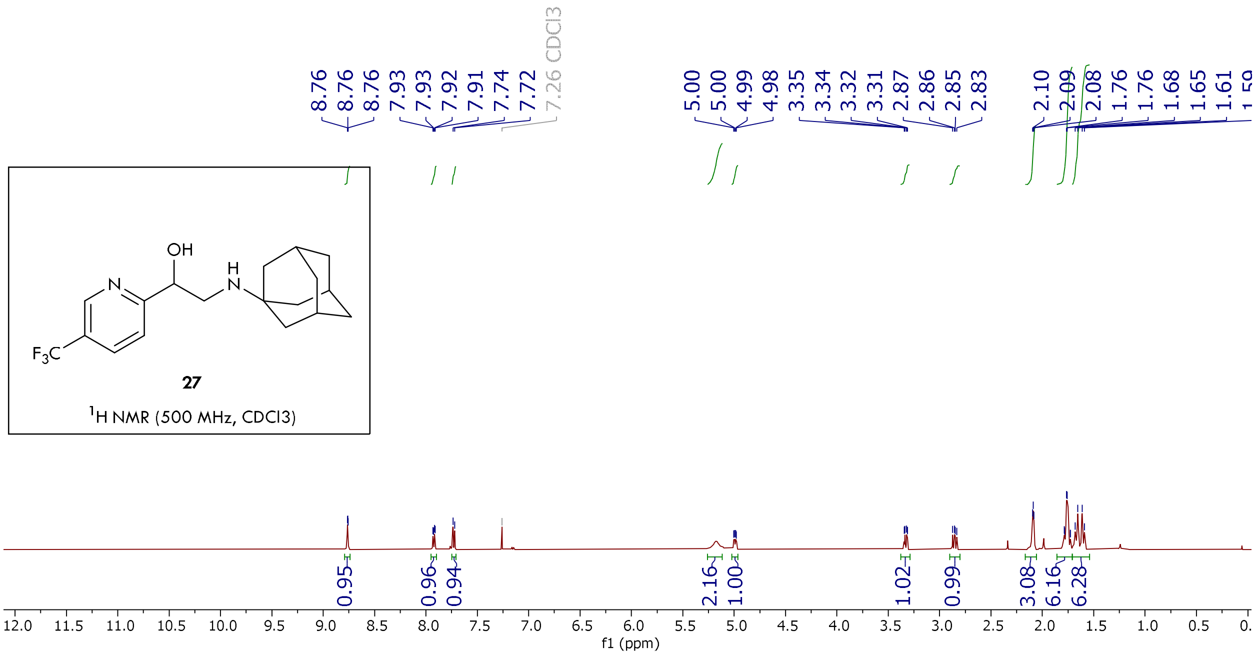

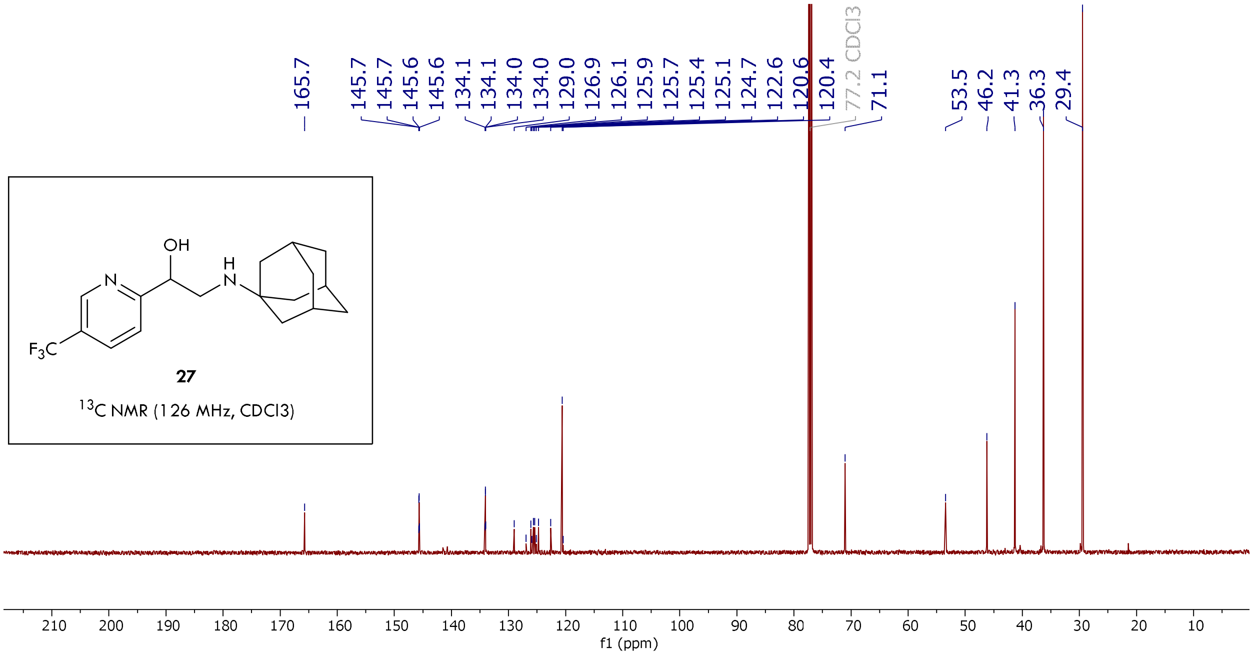

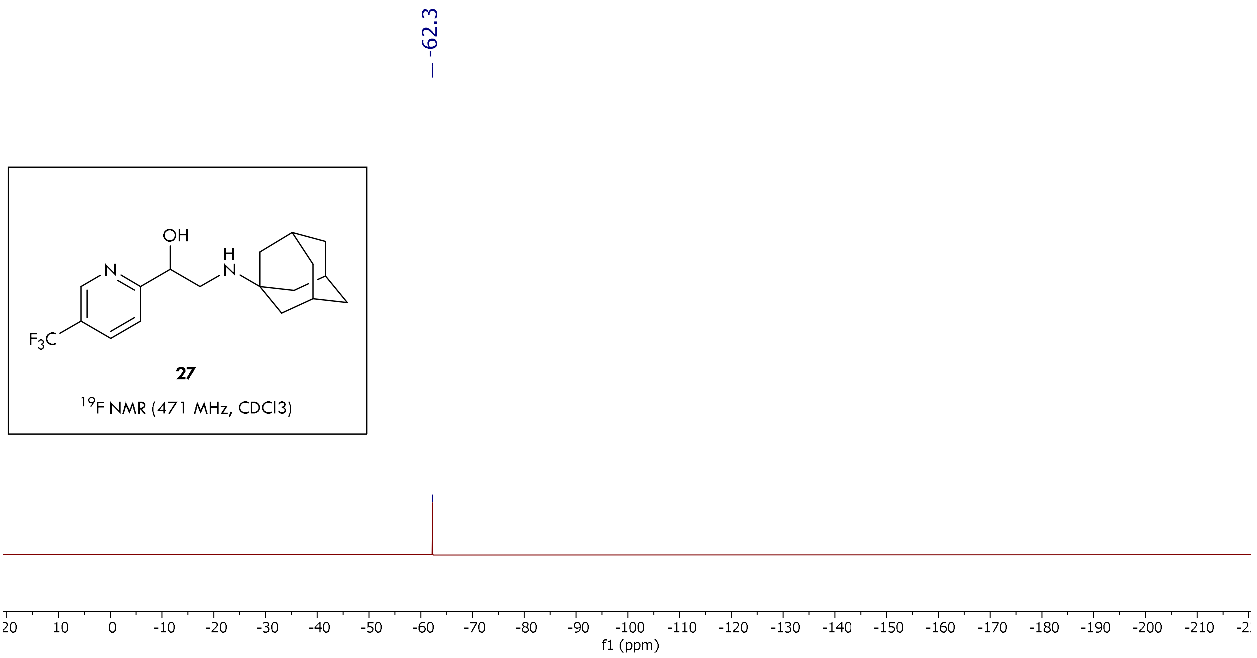
-62.3
OH
H
N
N
F3C
27
19F NMR (471 MHz, CDCl3)
f1 (ppm)

2-(tert-butylamino)-1-(5-(trifluoromethyl)pyridin-2-yl)ethan-1-ol (28)

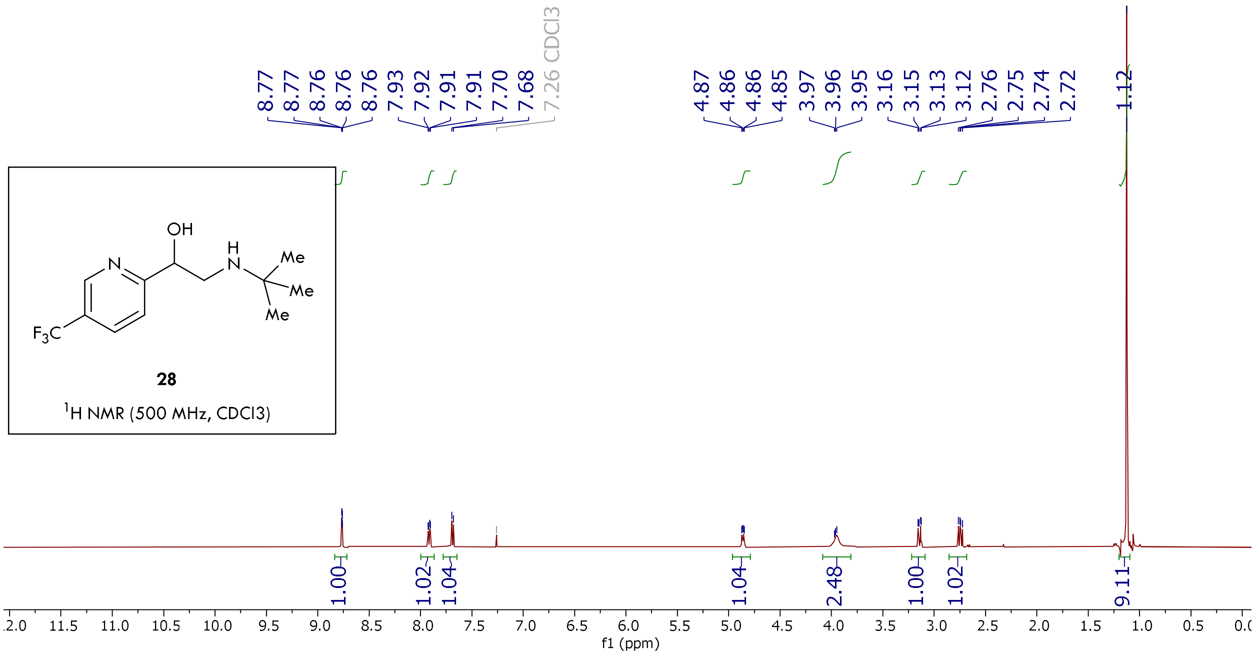

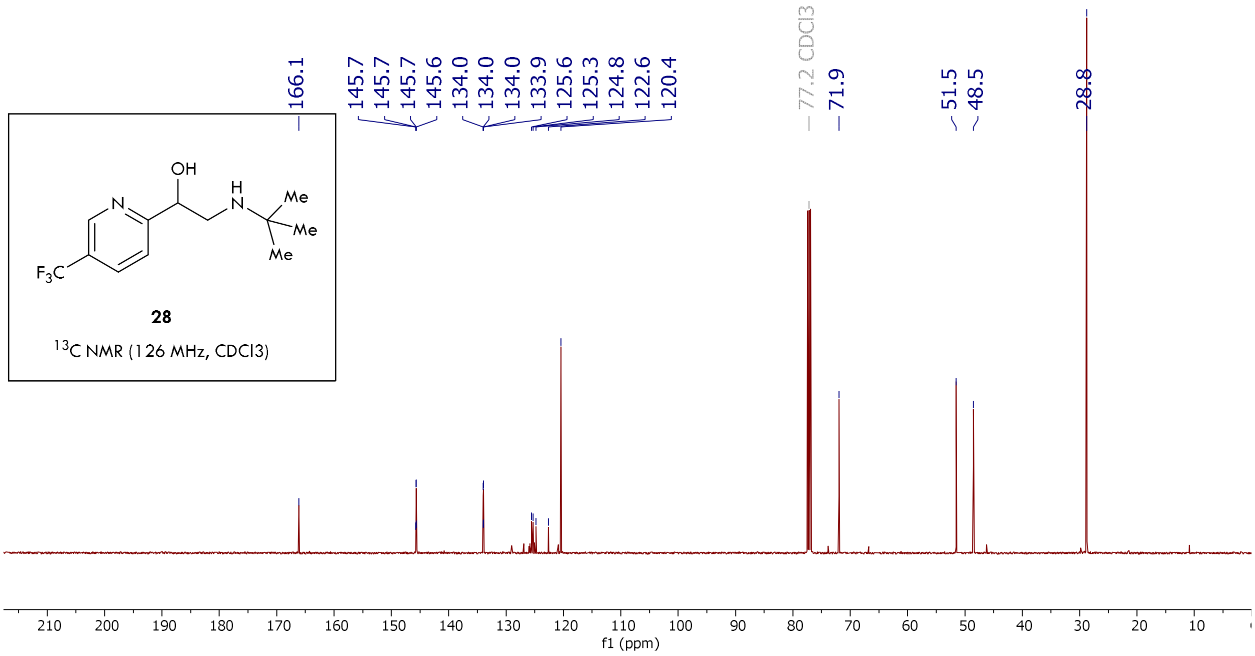

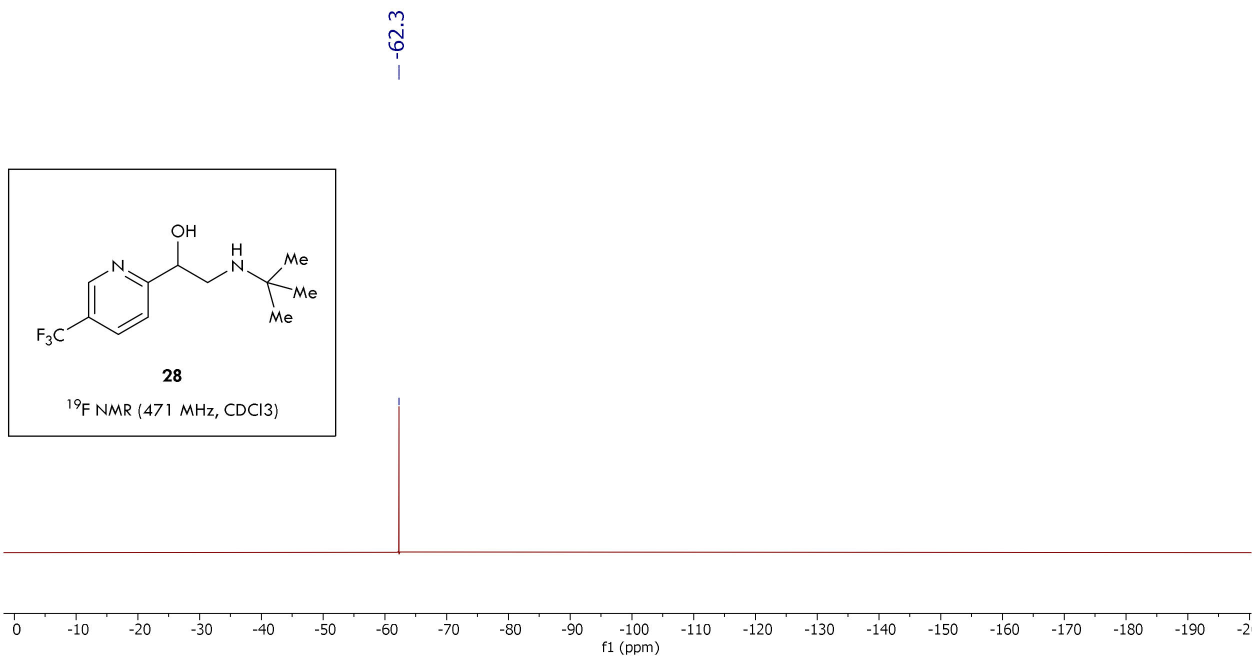

28

^{19}F NMR (471 MHz, CDCl3)

2-(1H-imidazol-1-yl)-1-(5-(trifluoromethyl)pyridin-2-yl)ethan-1-ol (29)

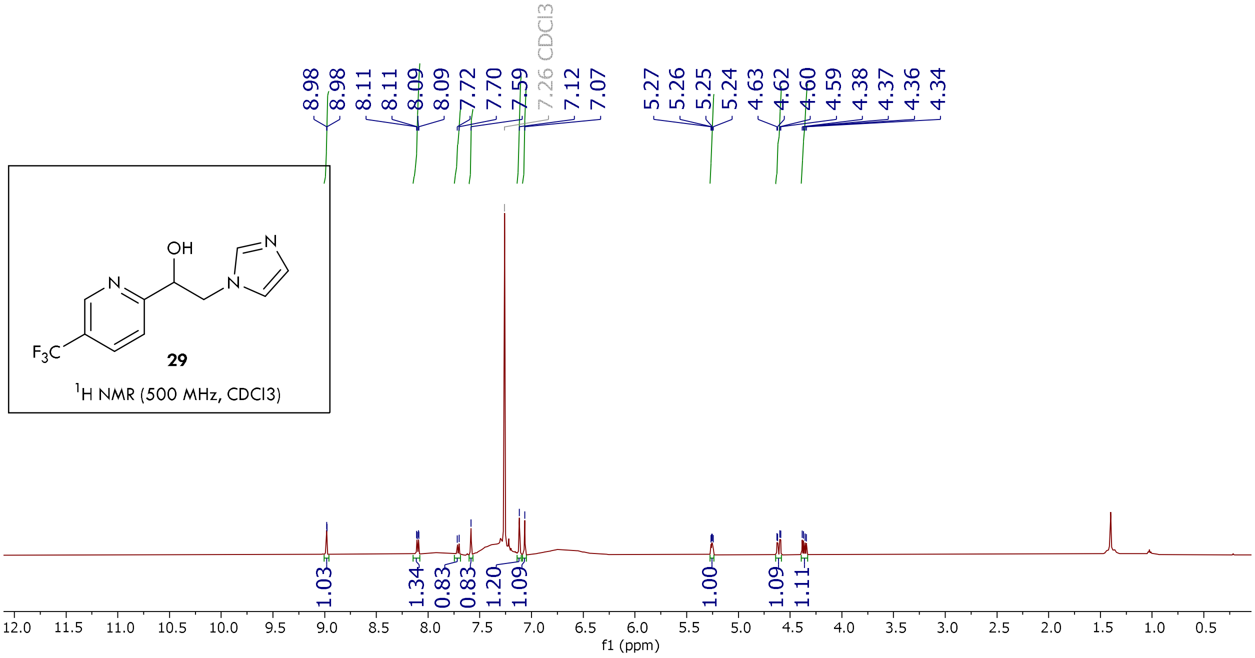

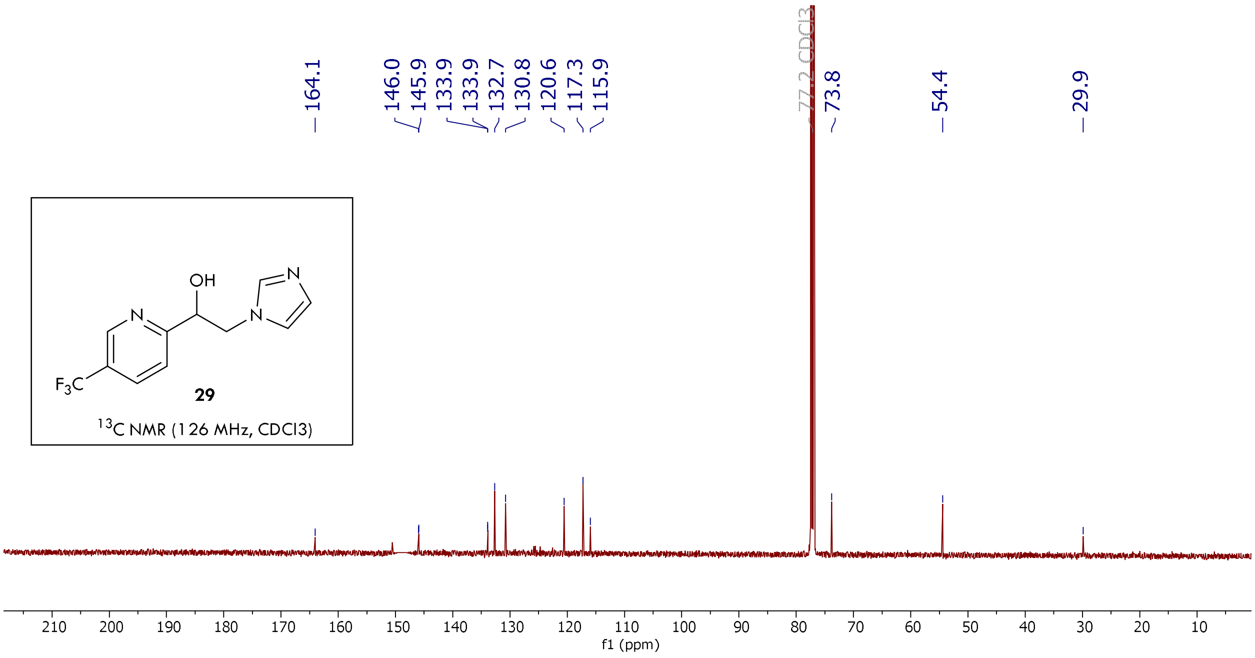

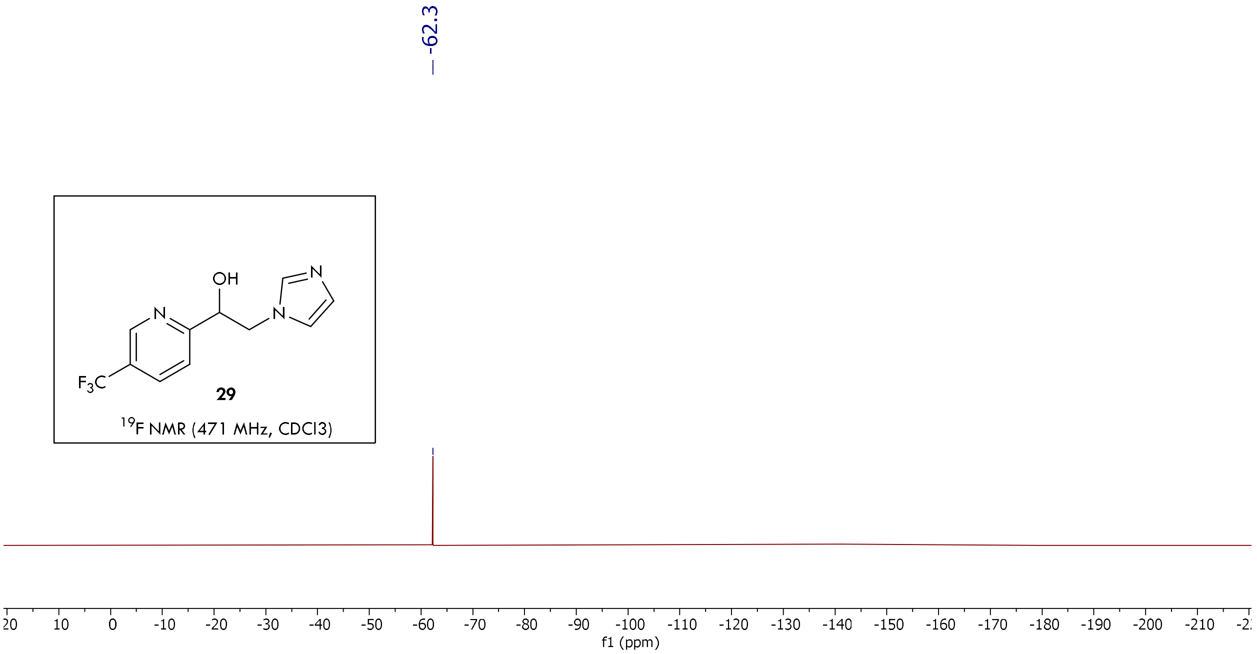

-62.3
OH
N
N
N
F3C
29
19F NMR (471 MHz, CDCl3)
20
10
0
-10
-20
-30
-40
-50
-60
-70
-80
-90
-100
-110
-120
-130
-140
-150
-160
-170
-180
-190
-200
-210
f1 (ppm)

2-(1H-pyrrolo[2,3-b]pyridin-1-yl)-1-(5-(trifluoromethyl)pyridin-2-yl)ethan-1-ol (30)

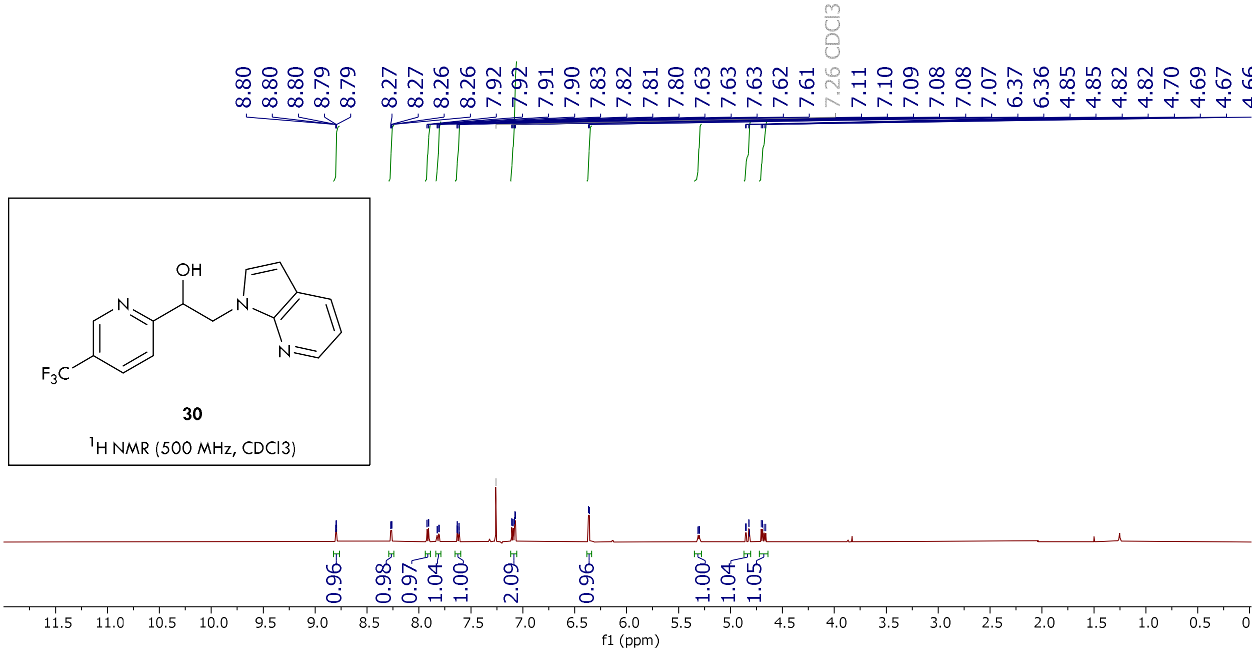

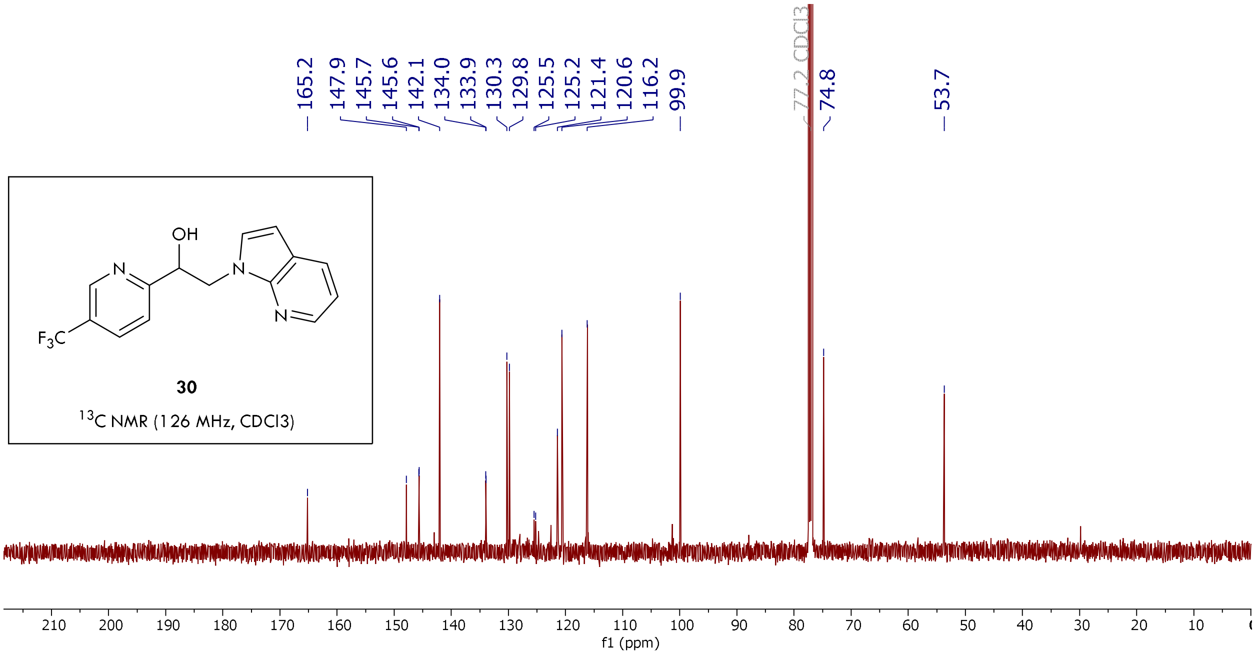

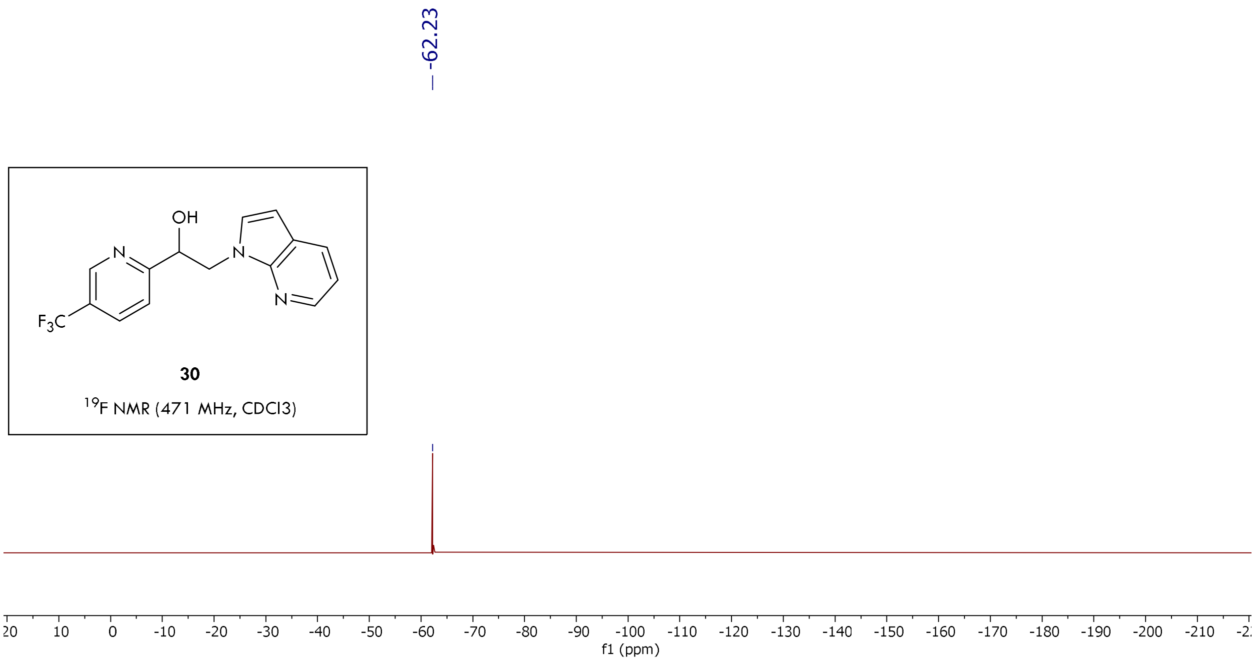

-62.23
OH
N
N
N
F3C
30
19F NMR (471 MHz, CDCl3)
20
10
0
-10
-20
-30
-40
-50
-60
-70
-80
-90
-100
-110
-120
-130
-140
-150
-160
-170
-180
-190
-200
-210
f1 (ppm)

2-(1H-pyrazolo[3,4-b]pyridin-1-yl)-1-(5-(trifluoromethyl)pyridin-2-yl)ethan-1-ol (31)

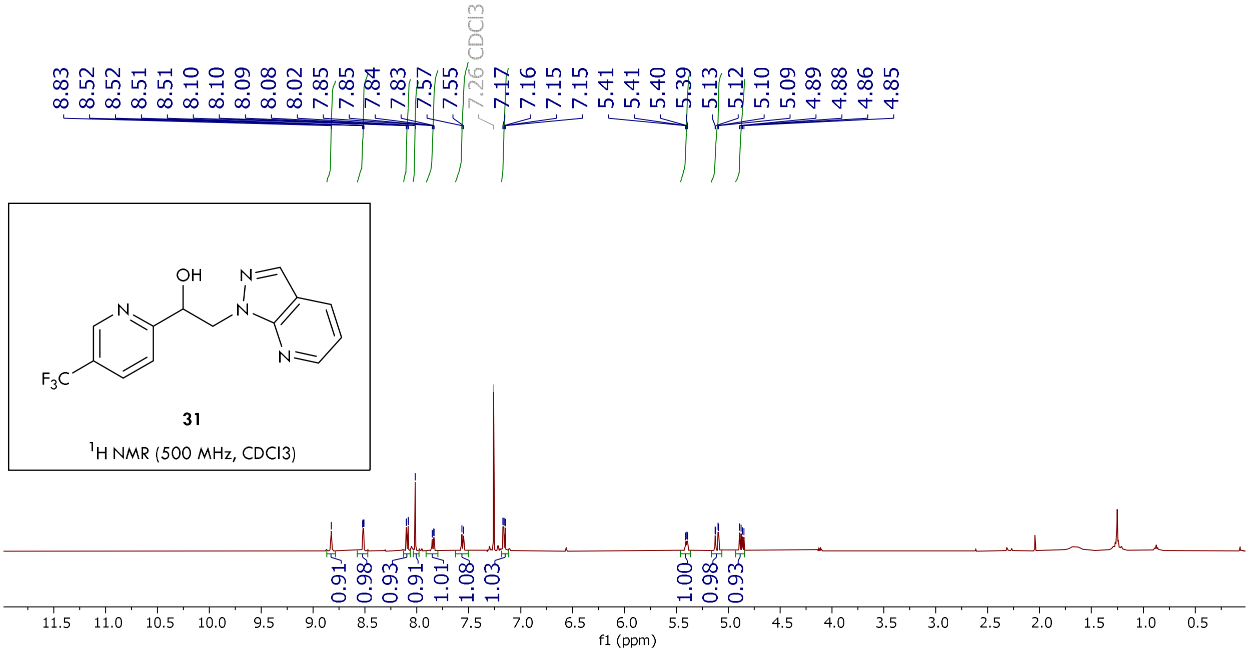

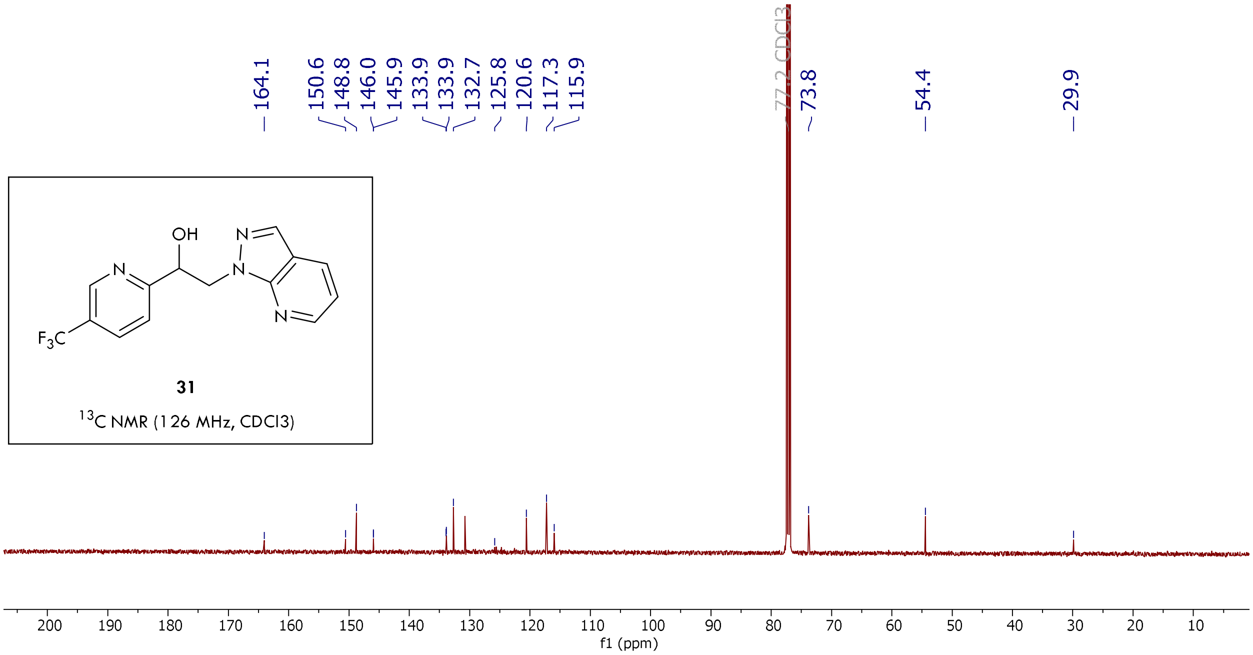

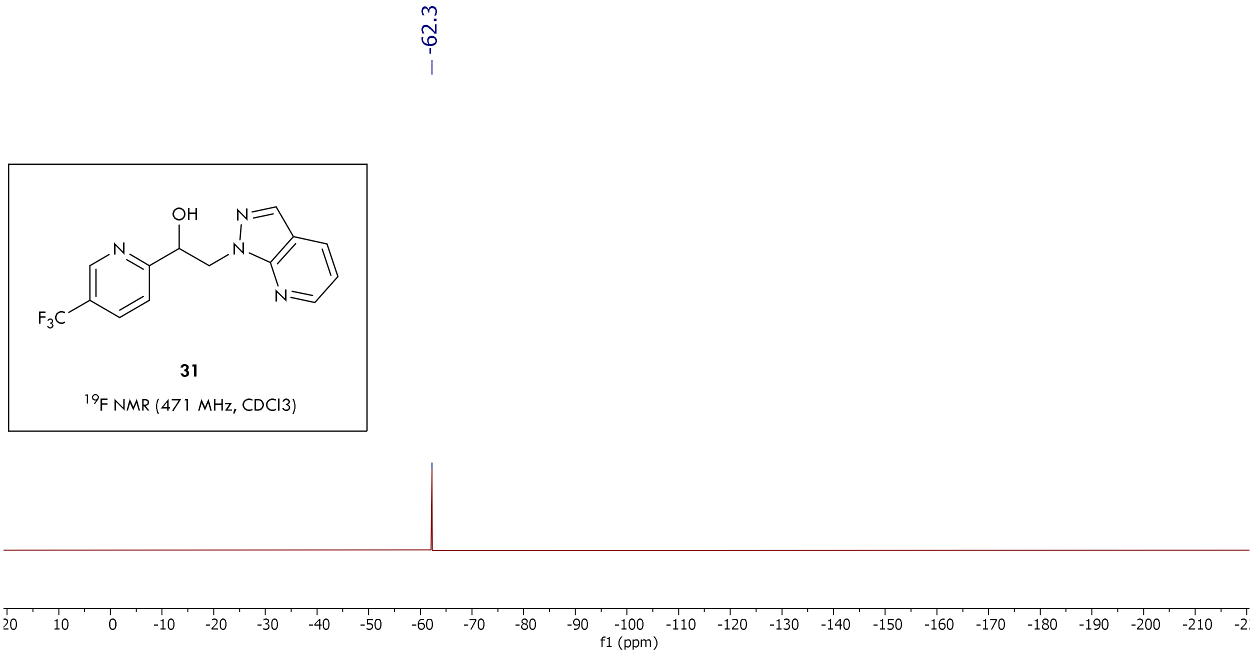
-62.3
OH
N
N
N
N
F3C
31
19F NMR (471 MHz, CDCl3)
20 10 0 -10 -20 -30 -40 -50 -60 -70 -80 -90 -100 -110 -120 -130 -140 -150 -160 -170 -180 -190 -200 -210
f1 (ppm)

2-((1R,4R)-5-benzyl-2,5-diazabicyclo[2.2.1]heptan-2-yl)-1-(5-(trifluoromethyl)pyridin-2-yl)ethan-1-ol (32)

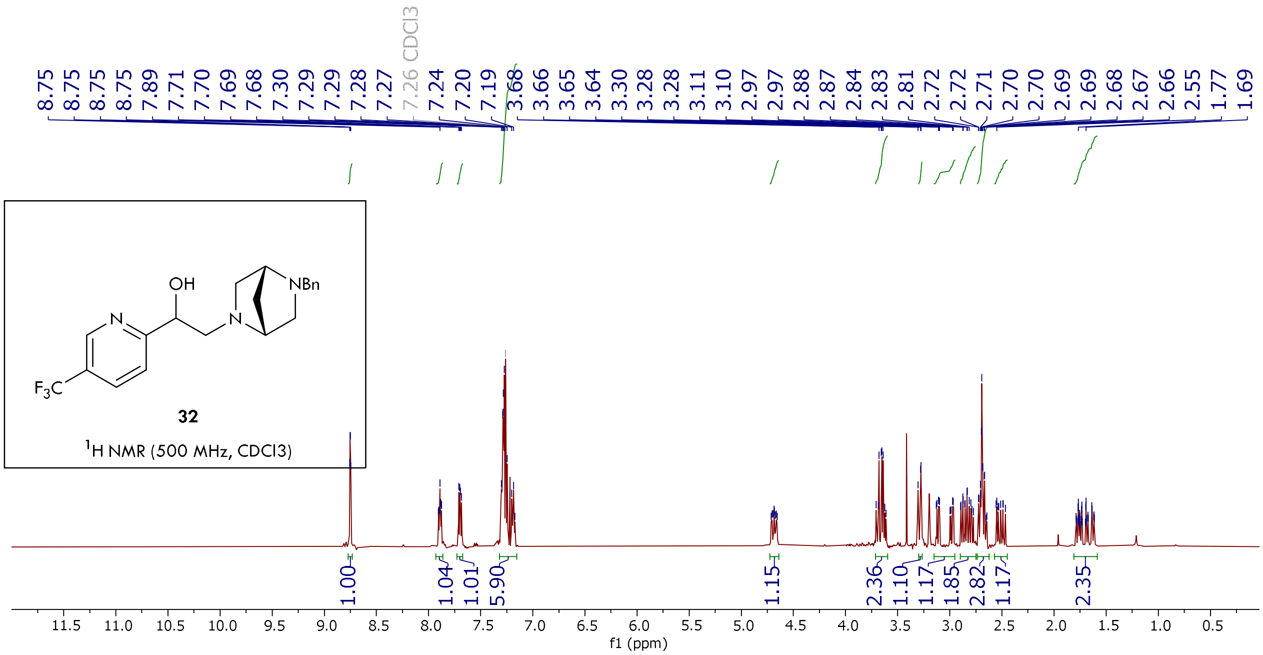

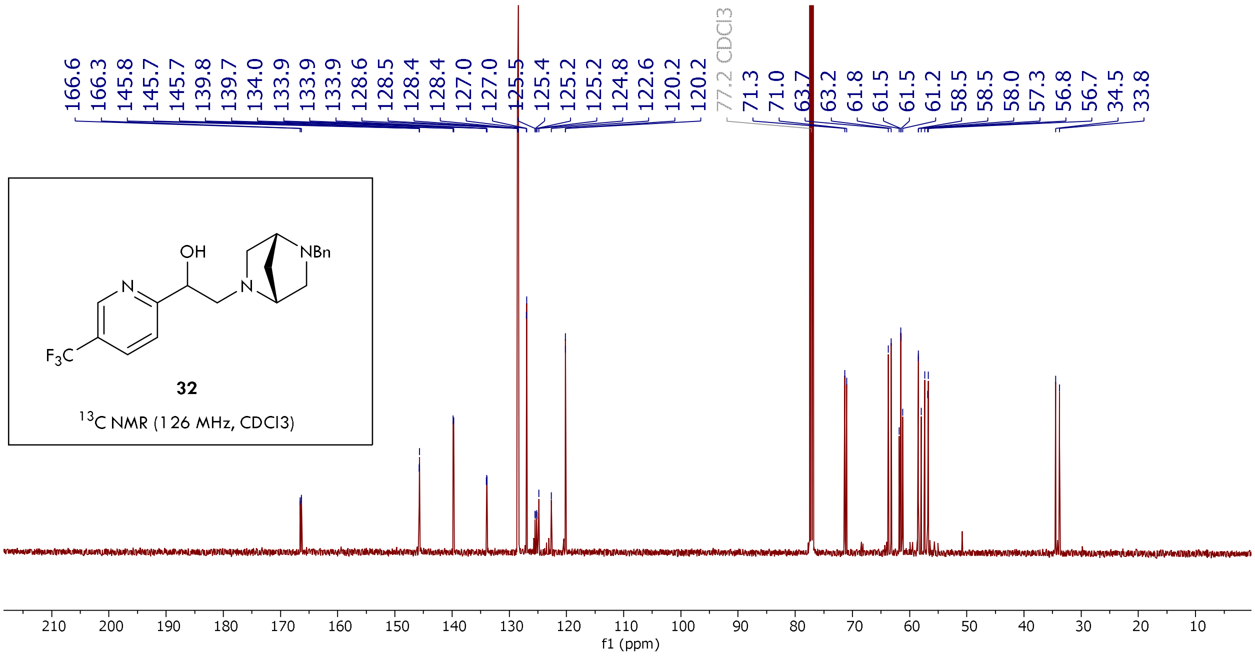

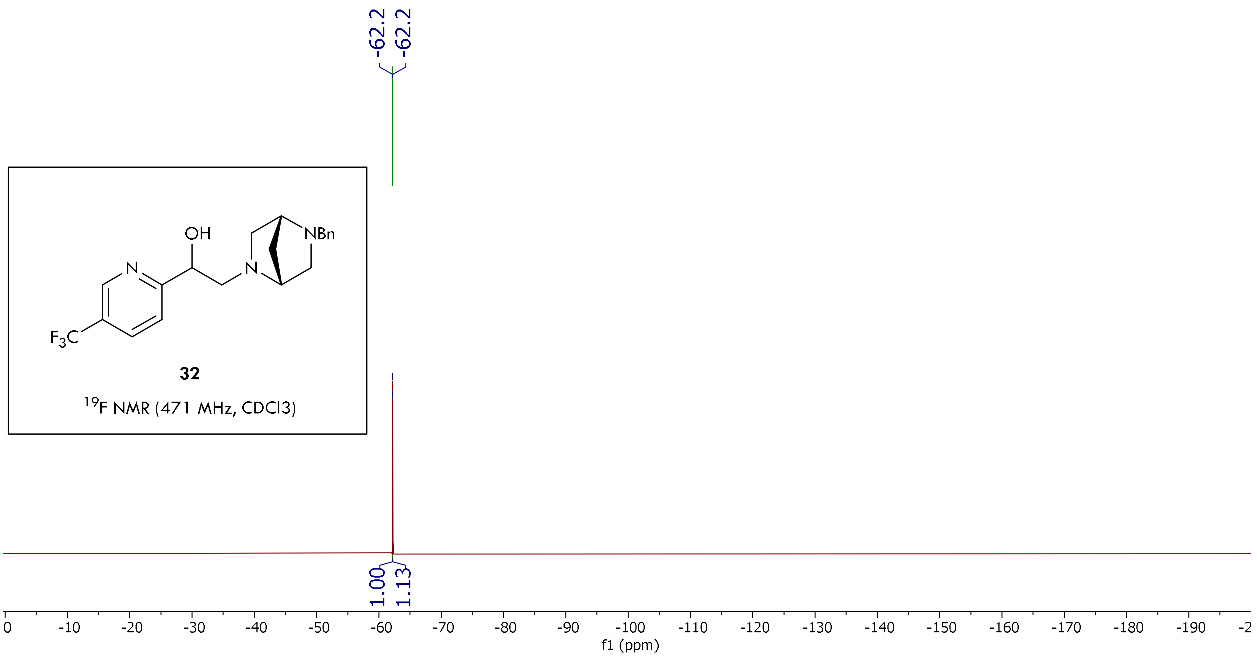
-62.2
-62.2
OH
NBn
N
N
F3C
32
19F NMR (471 MHz, CDCl3)
1.00
1.13
0 -10 -20 -30 -40 -50 -60 -70 -80 -90 -100 -110 -120 -130 -140 -150 -160 -170 -180 -190 -2
f1 (ppm)

tert-butyl 2-(2-hydroxy-2-(5-(trifluoromethyl)pyridin-2-yl)ethyl)-2,7-diazaspiro[3.5]nonane-7-carboxylate (33)

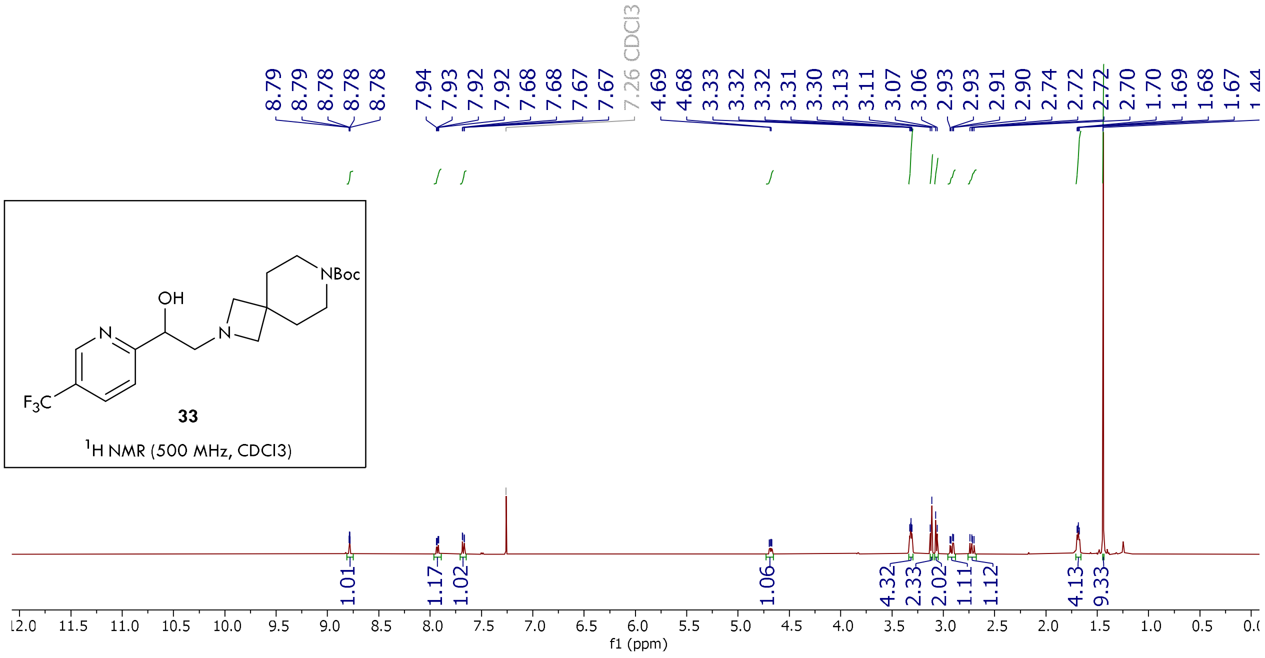

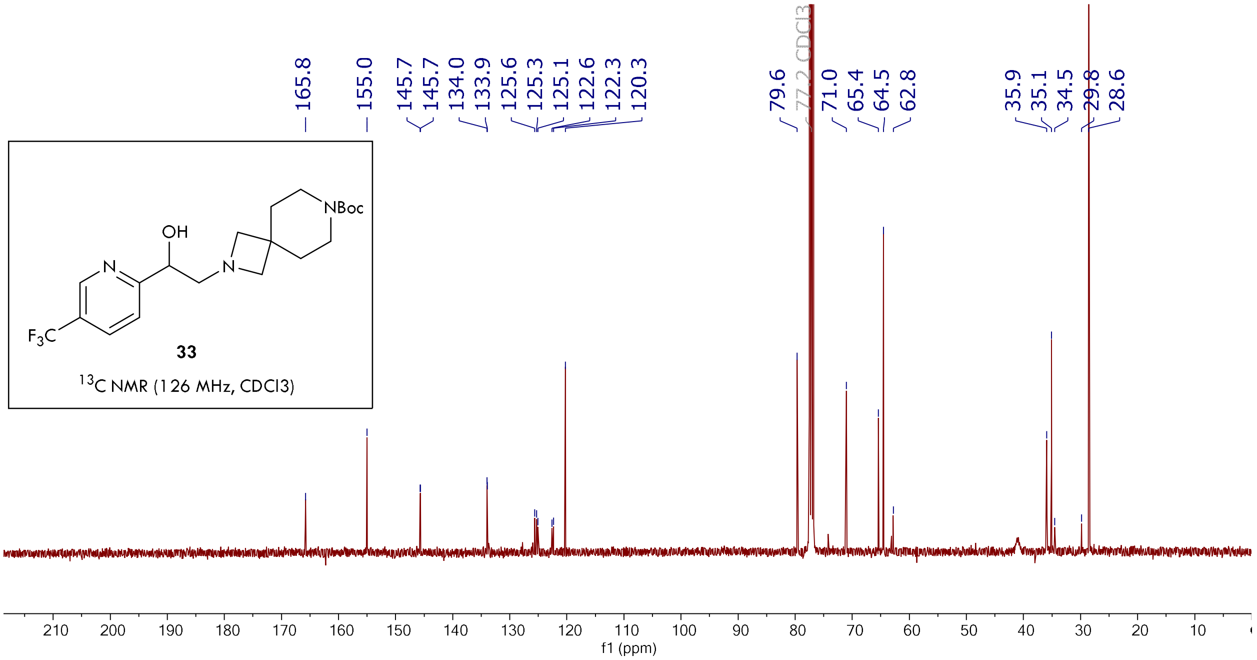

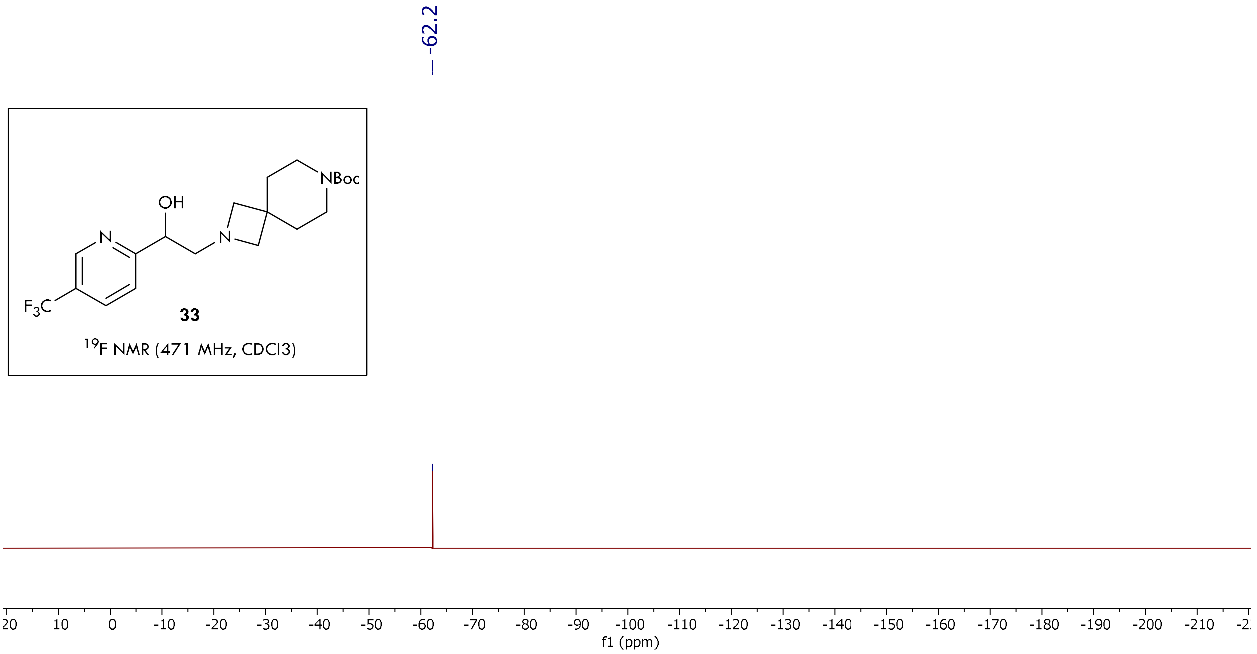

-62.2
OH
N
NBoc
N
F3C
33
19F NMR (471 MHz, CDCl3)
20
10
0
-10
-20
-30
-40
-50
-60
-70
-80
-90
-100
-110
-120
-130
-140
-150
-160
-170
-180
-190
-200
-210
f1 (ppm)

2-azido-1-(5-(trifluoromethyl)pyridin-2-yl)ethan-1-ol (34)

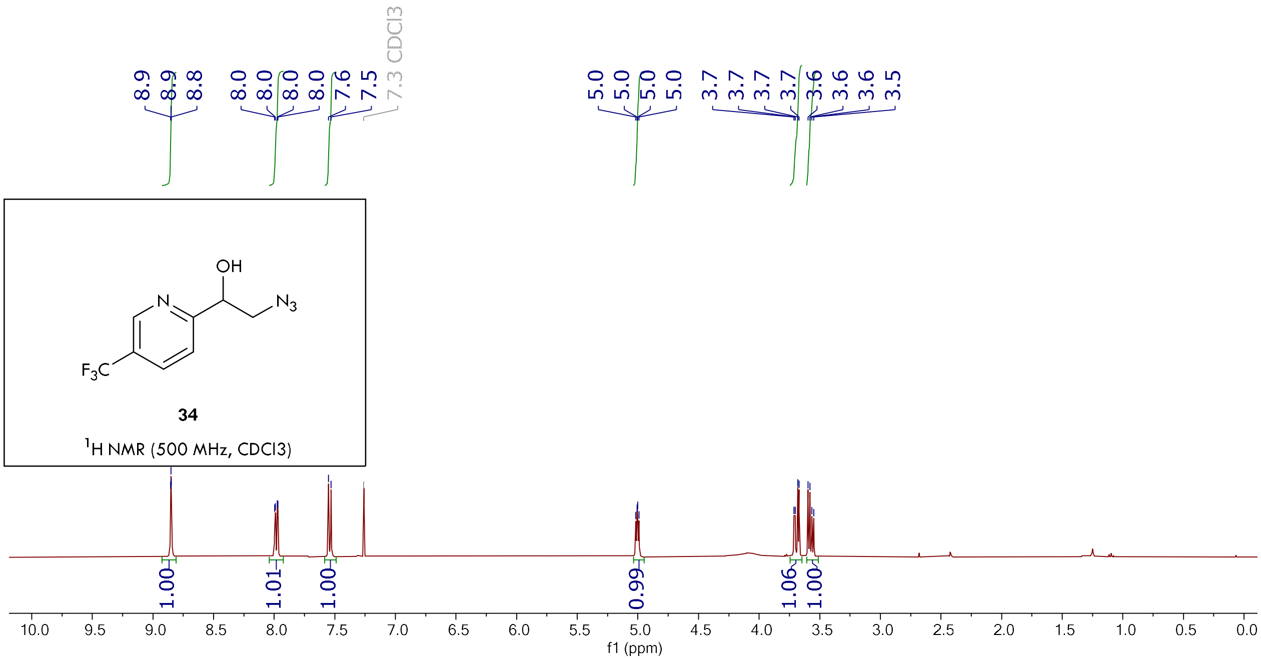

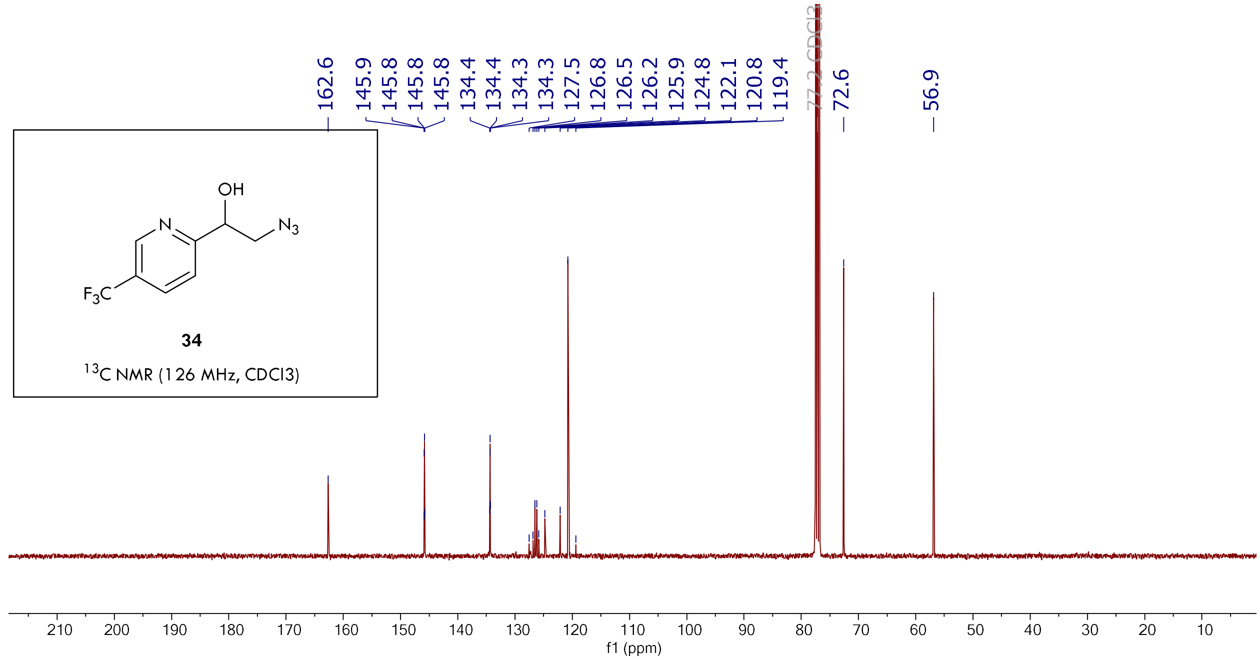

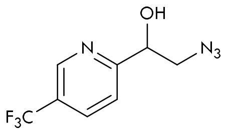

34

^{19}F NMR (471 MHz, CDCl3)

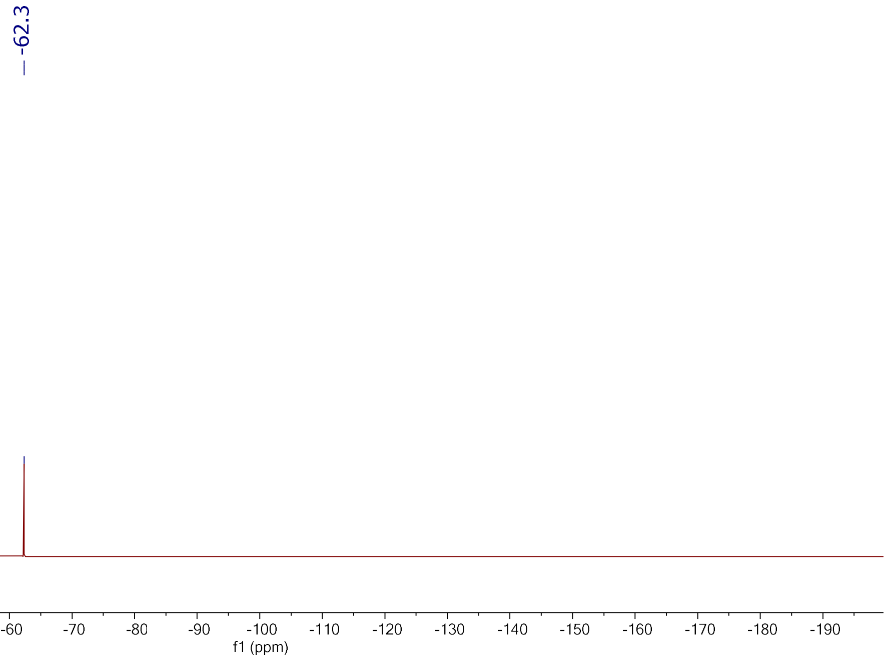

2-(4-(8-chloro-5,6-dihydro-11H-benzo[5,6]cyclohepta[1,2-b]pyridin-11-ylidene)piperidin-1-yl)-1-(5-(trifluoromethyl)pyridin-2-yl)ethan-1-ol (35)

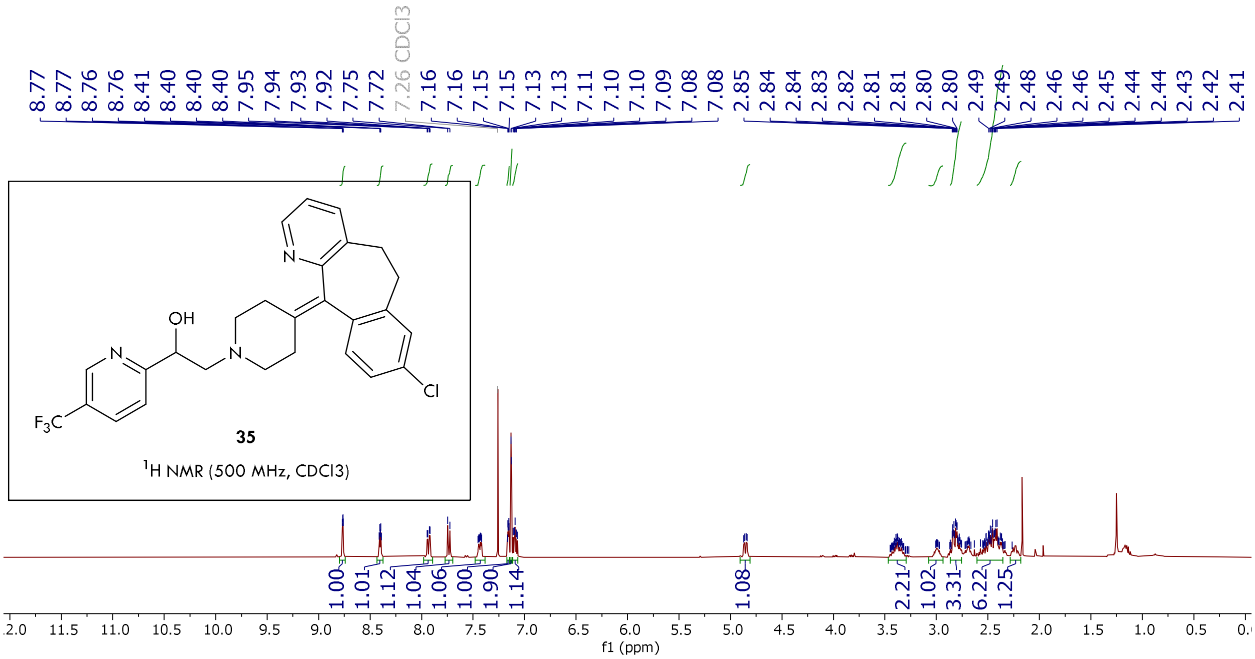

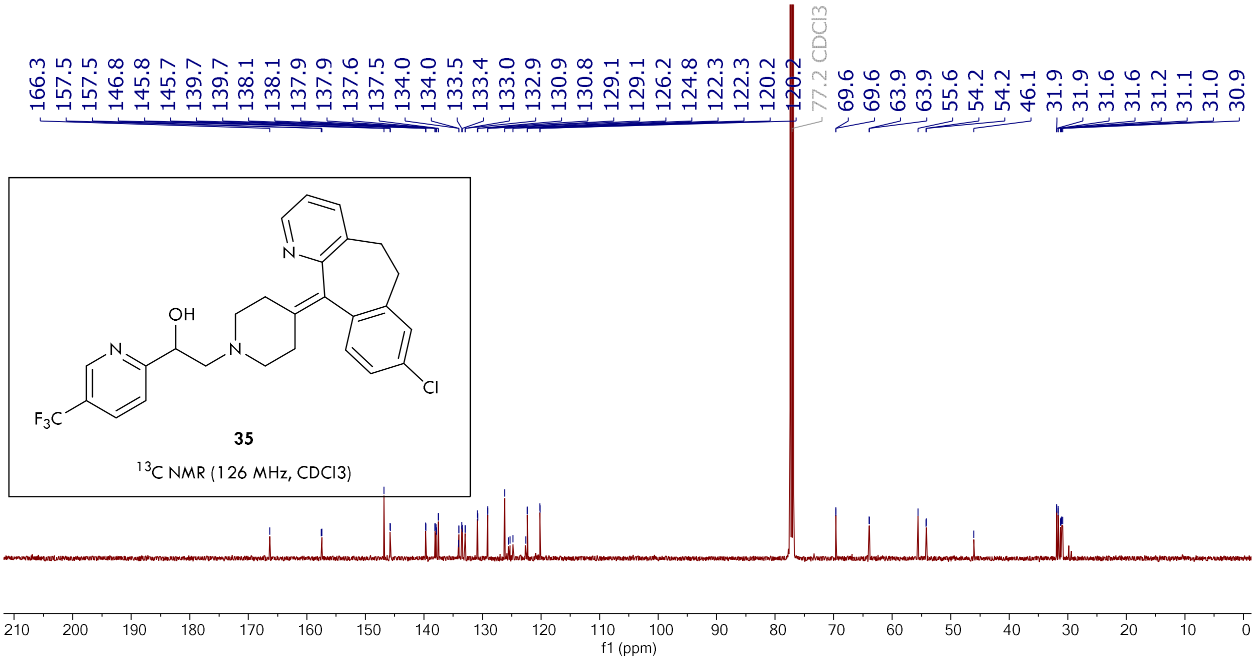

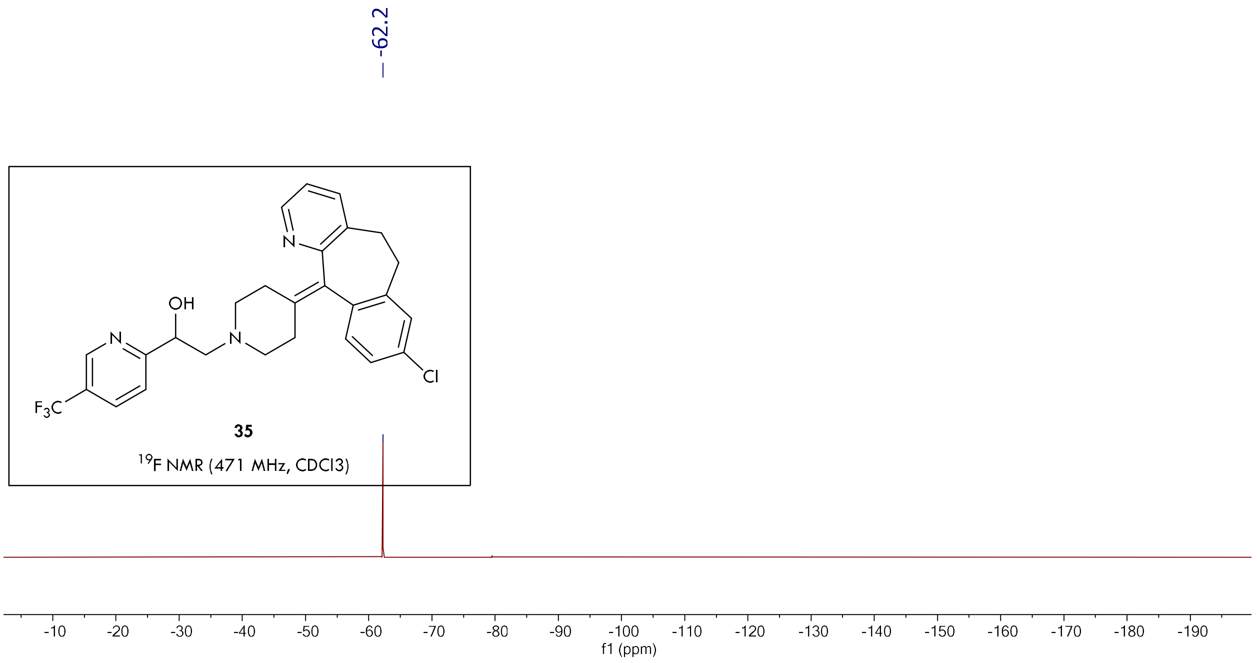

-62.2
OH
N
N
N
F3C
Cl
35
19F NMR (471 MHz, CDCl3)
-10
-20
-30
-40
-50
-60
-70
-80
-90
-100
-110
-120
-130
-140
-150
-160
-170
-180
-190
f1 (ppm)

2-(4-(isoquinolin-5-ylsulfonyl)-1,4-diazepan-1-yl)-1-(5-(trifluoromethyl)pyridin-2-yl)ethan-1-ol (36)

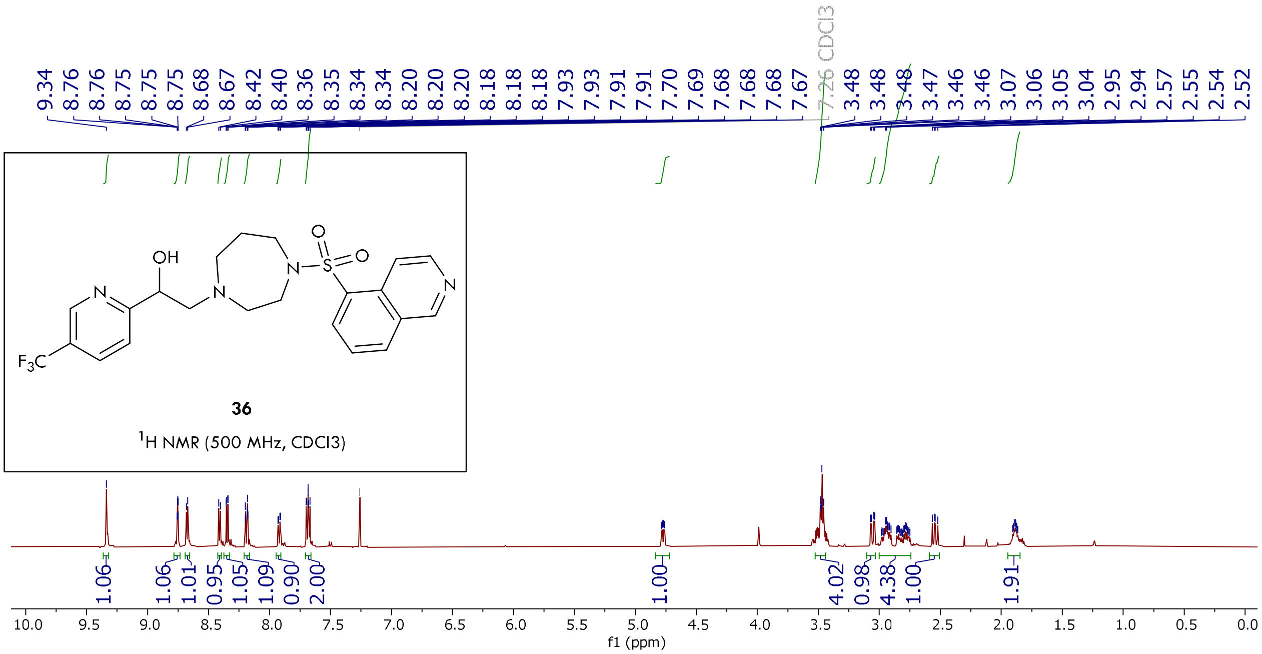

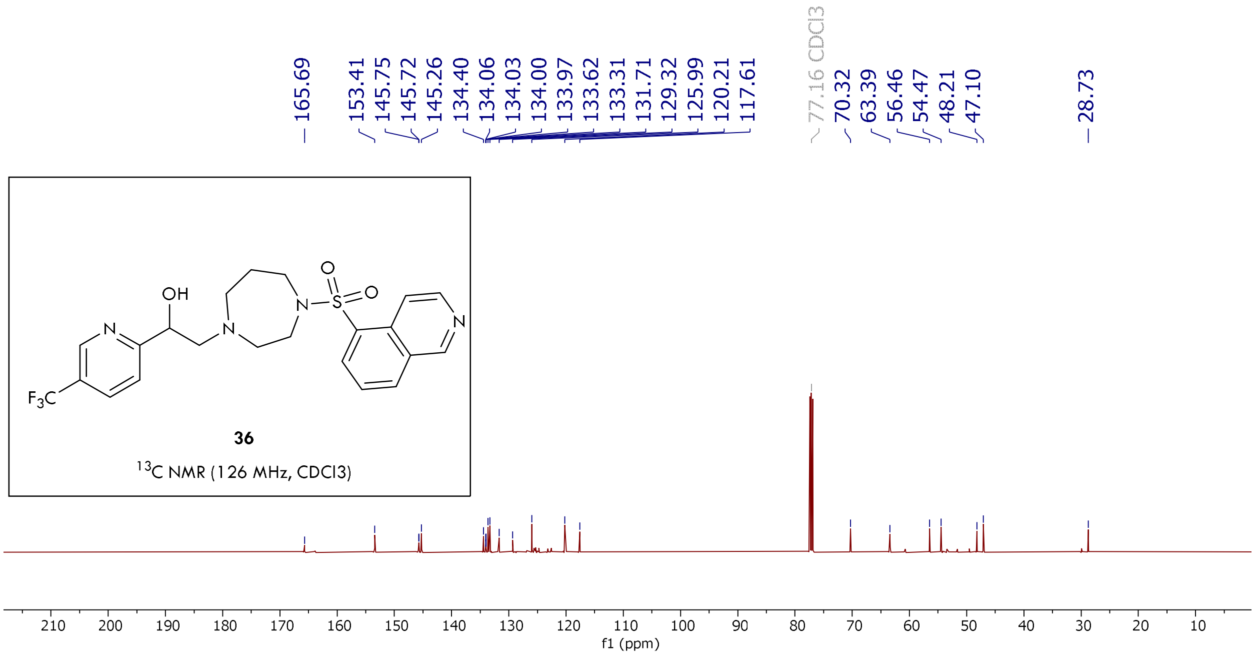

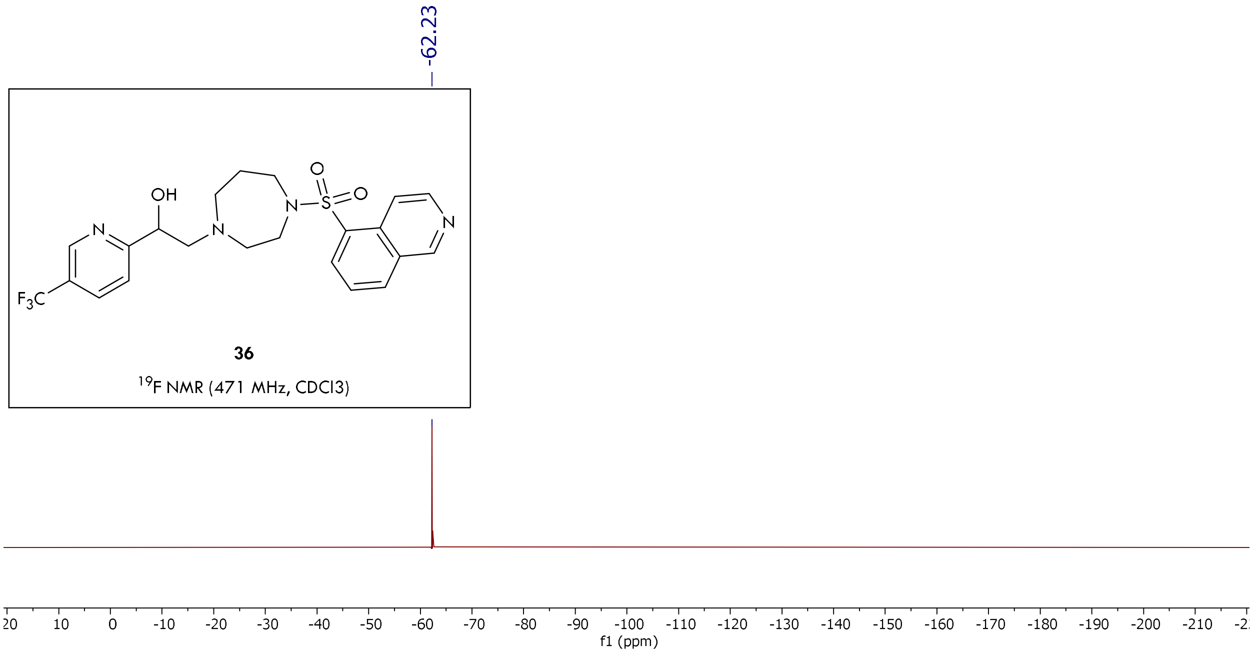
-62.23
OH
N
O
S
O
N
N
N
F3C
36
19F NMR (471 MHz, CDCl3)
20
10
0
-10
-20
-30
-40
-50
-60
-70
-80
-90
-100
-110
-120
-130
-140
-150
-160
-170
-180
-190
-200
-210
f1 (ppm)

2-((1-(2-hydroxy-2-(5-(trifluoromethyl)pyridin-2-yl)ethyl)piperidin-4-yl)methyl)-5,6-dimethoxy-2,3-dihydro-1H-inden-1-one (37)

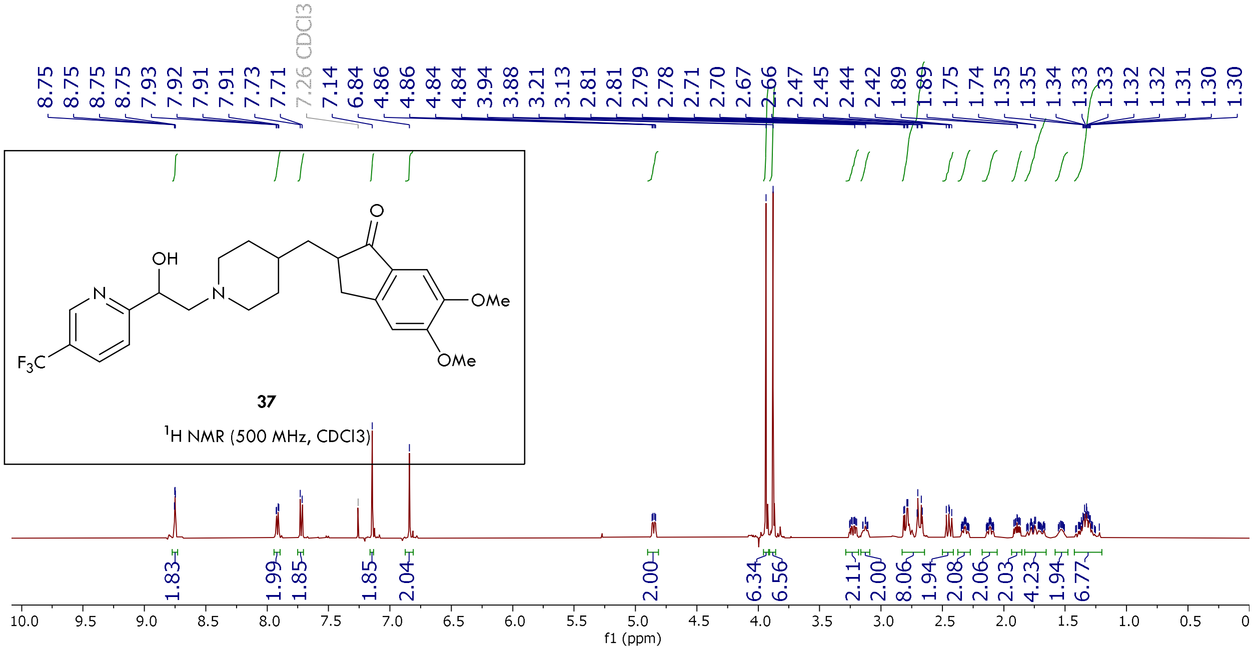

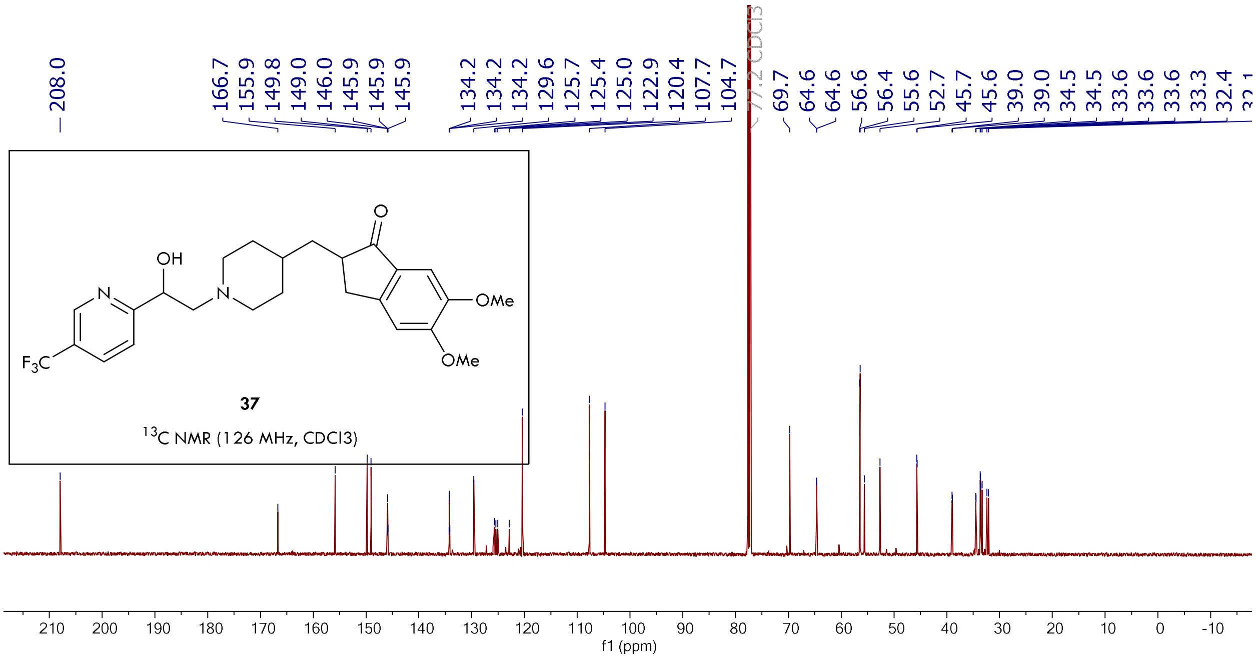

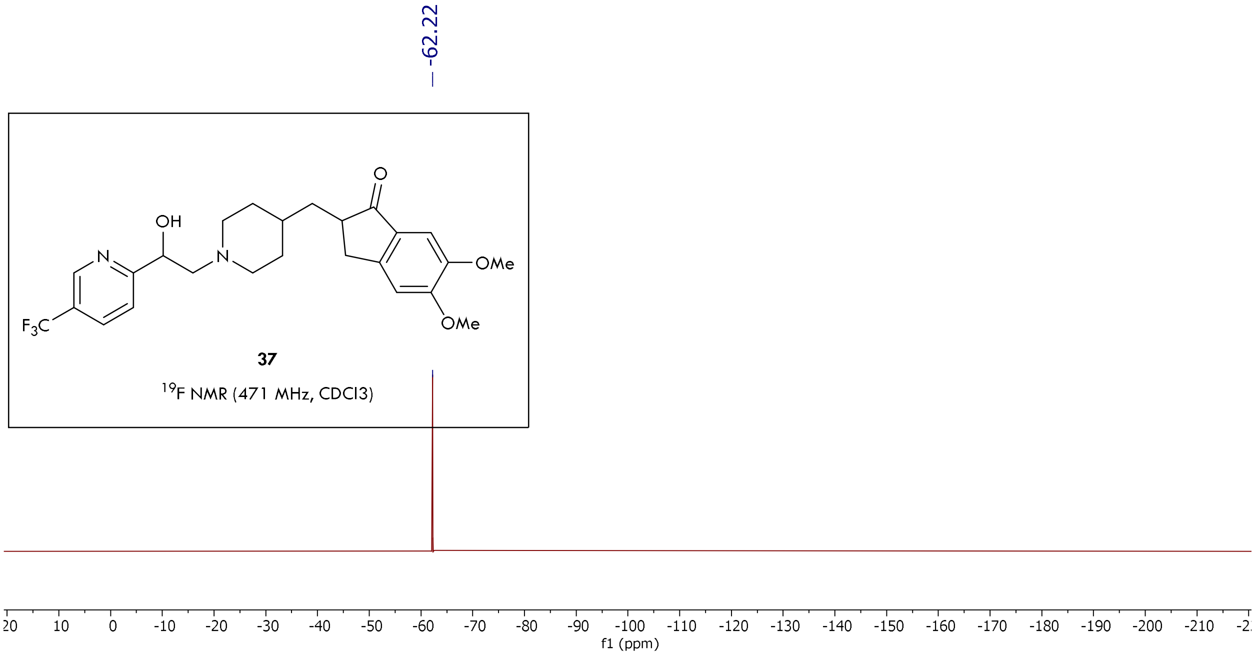
-62.22
OH
N
N
O
OMe
OMe
F3C
37
19F NMR (471 MHz, CDCl3)
20 10 0 -10 -20 -30 -40 -50 -60 -70 -80 -90 -100 -110 -120 -130 -140 -150 -160 -170 -180 -190 -200 -210
f1 (ppm)

5-(2-ethoxy-5-((4-(2-hydroxy-2-(5-(trifluoromethyl)pyridin-2-yl)ethyl)piperazin-1-yl)sulfonyl)phenyl)-1-methyl-3-propyl-1,6-dihydro-7H-pyrazolo[4,3-d]pyrimidin-7-one (38)

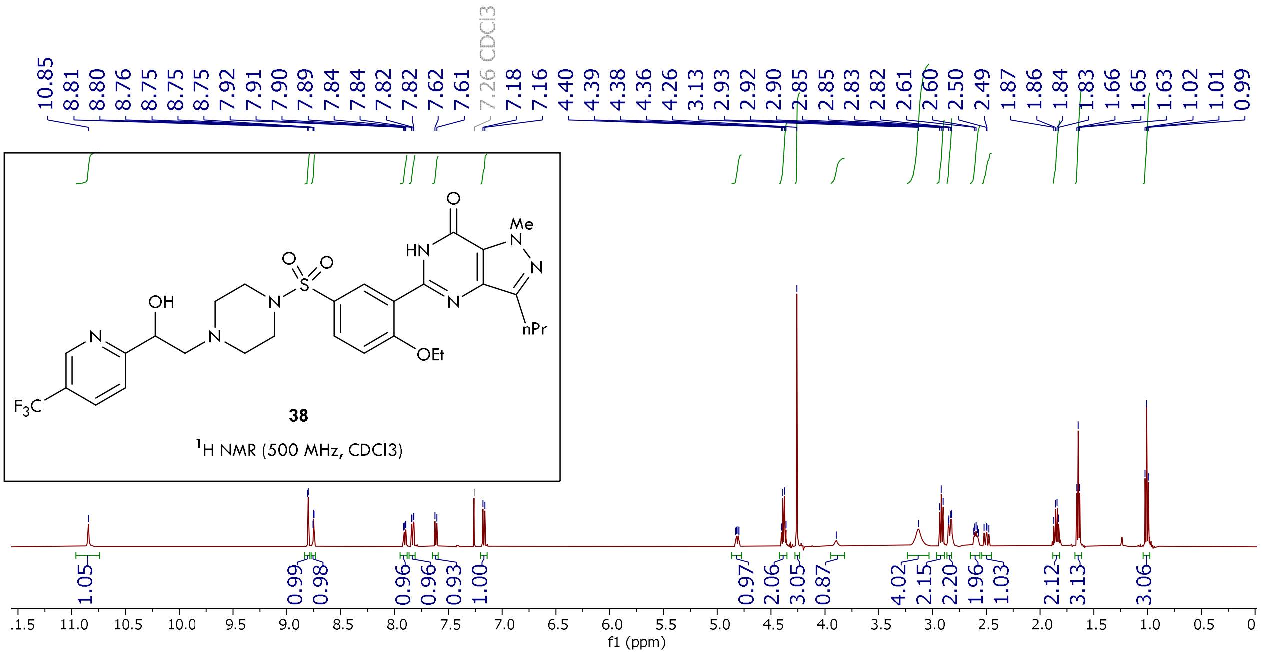

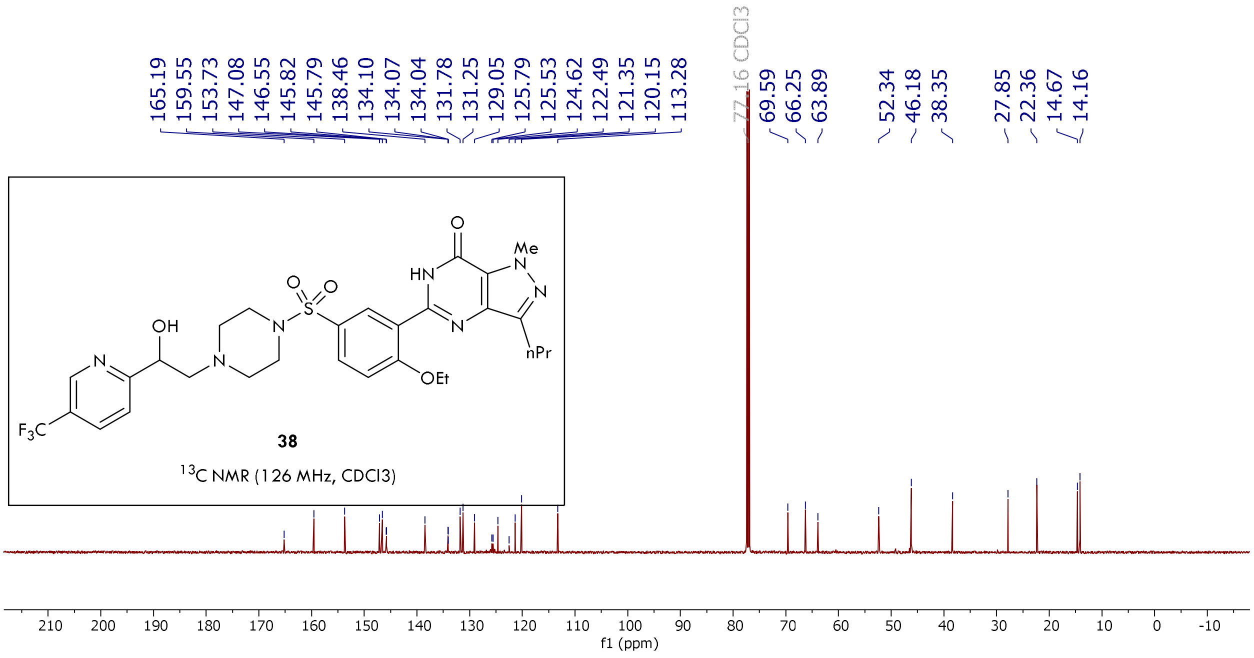

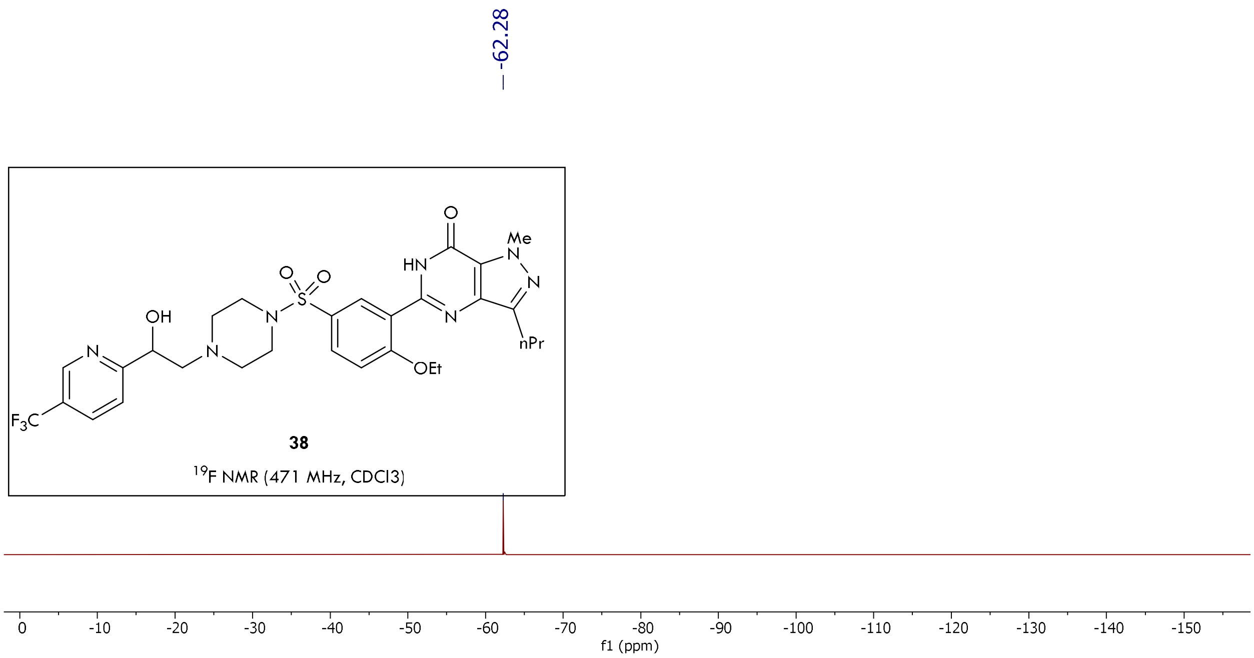
-62.28
38
^{19}F NMR (471 MHz, CDCl3)
0 -10 -20 -30 -40 -50 -60 -70 -80 -90 -100 -110 -120 -130 -140 -150
f1 (ppm)

5-(2-ethoxy-5-((4-(2-hydroxy-2-(5-(trifluoromethyl)pyridin-2-yl)ethyl)piperazin-1-yl)sulfonyl)phenyl)-1-methyl-3-propyl-1,6-dihydro-7H-pyrazolo[4,3-d]pyrimidin-7-one (40)

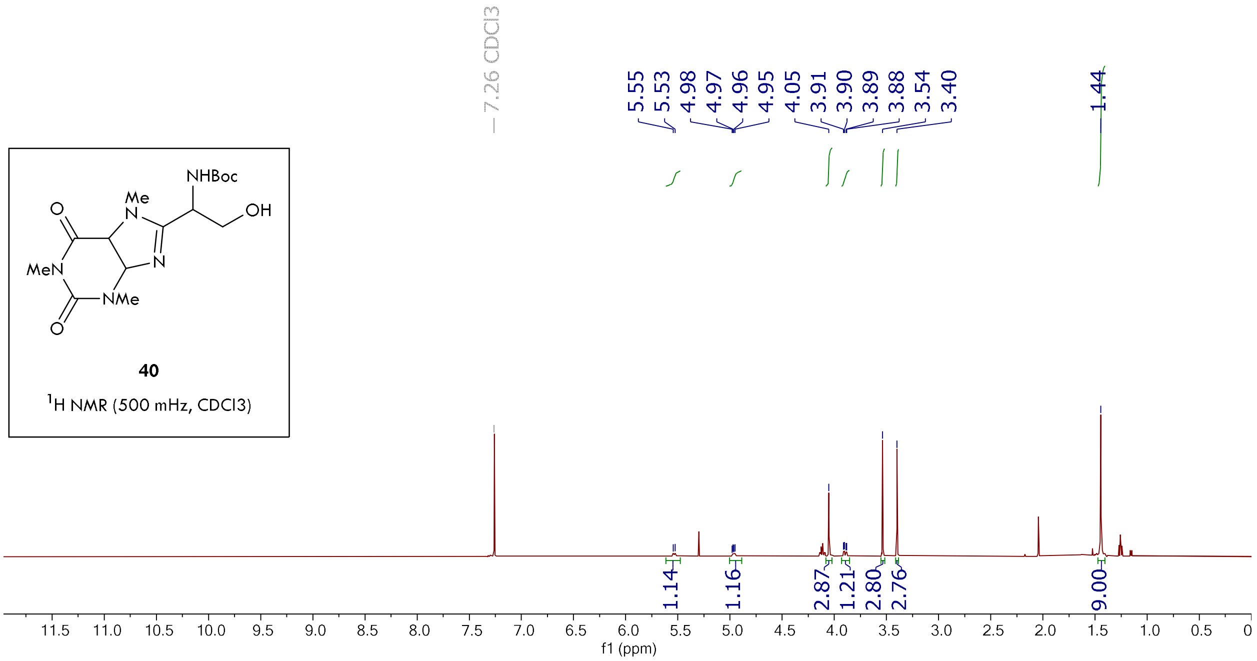

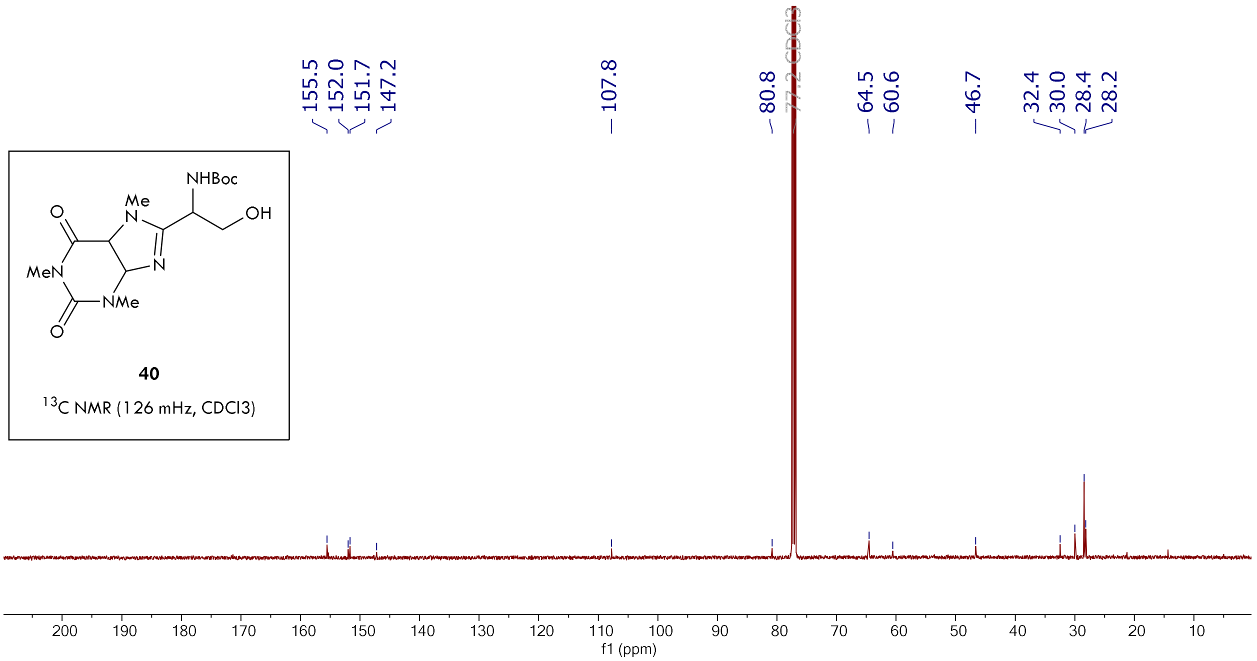

methyl 4-(1-((tert-butoxycarbonyl)amino)-2-hydroxyethyl)benzoate (41)

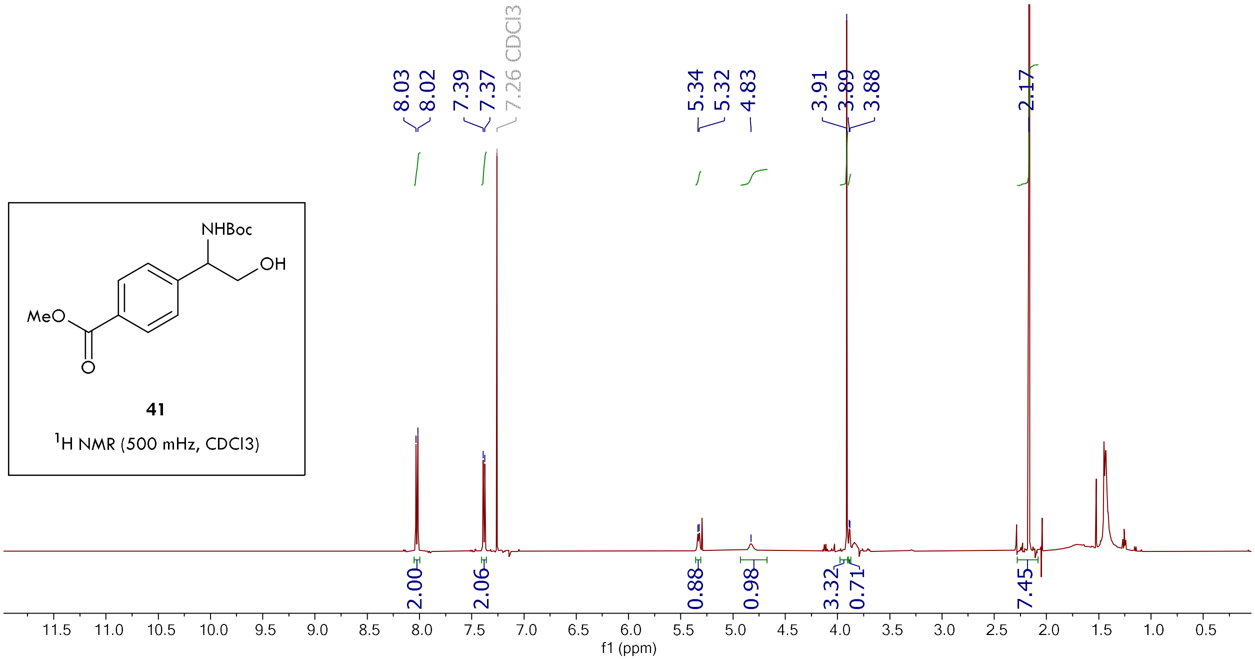

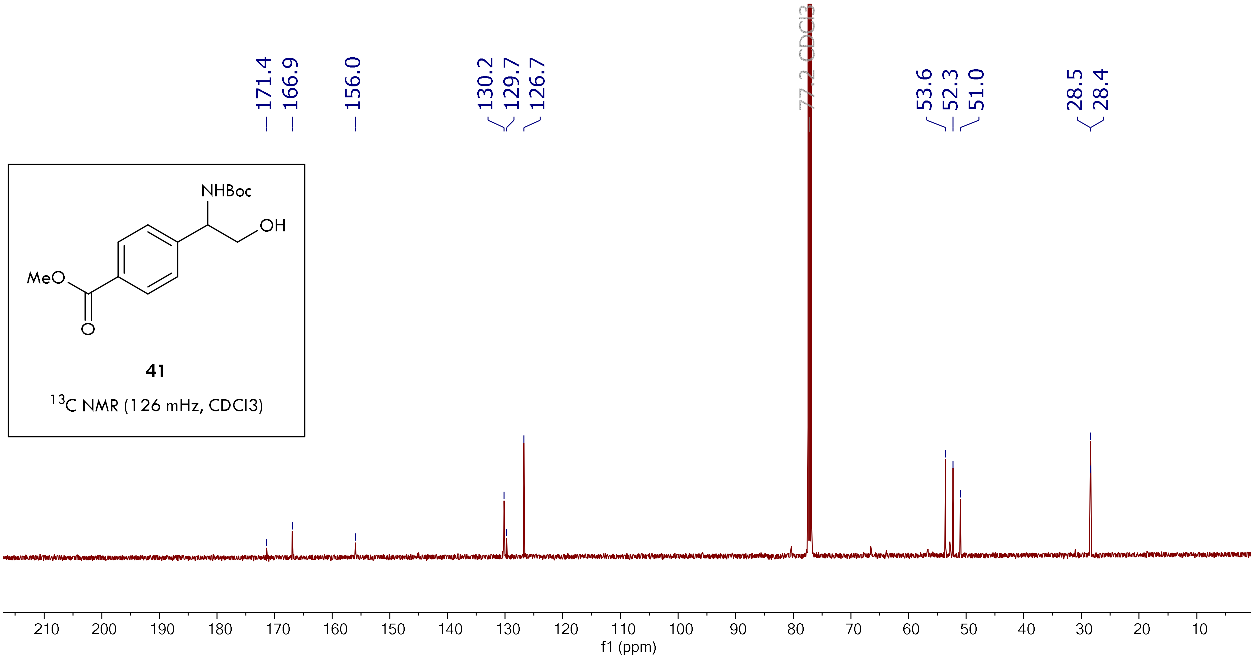

tert-butyl (1-(benzo[b]thiophen-5-yl)-2-hydroxyethyl)carbamate (42)

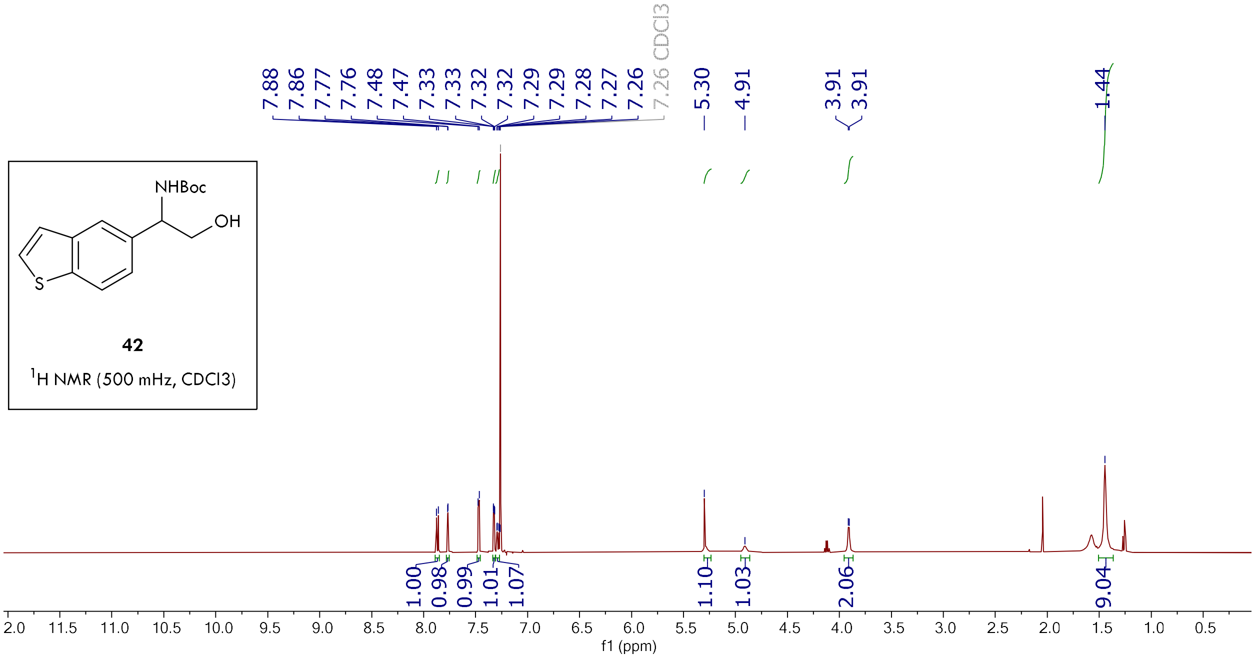

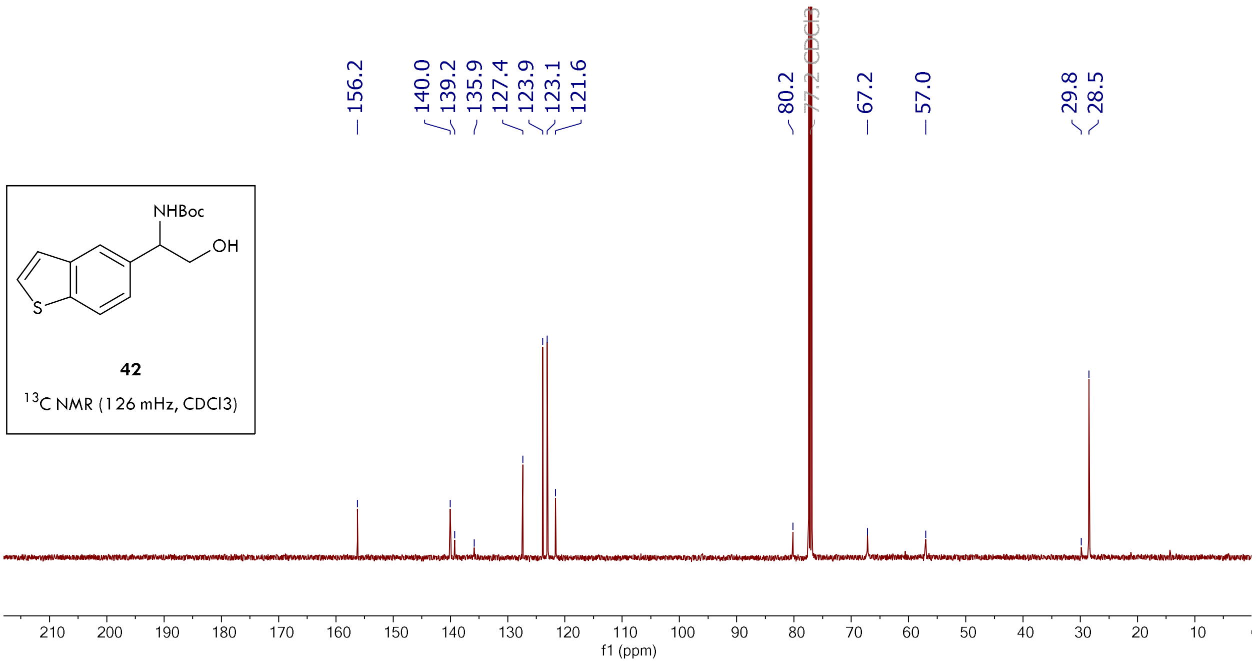

methyl 4-(1-((tert-butoxycarbonyl)amino)-2-morpholinoethyl)benzoate (43)

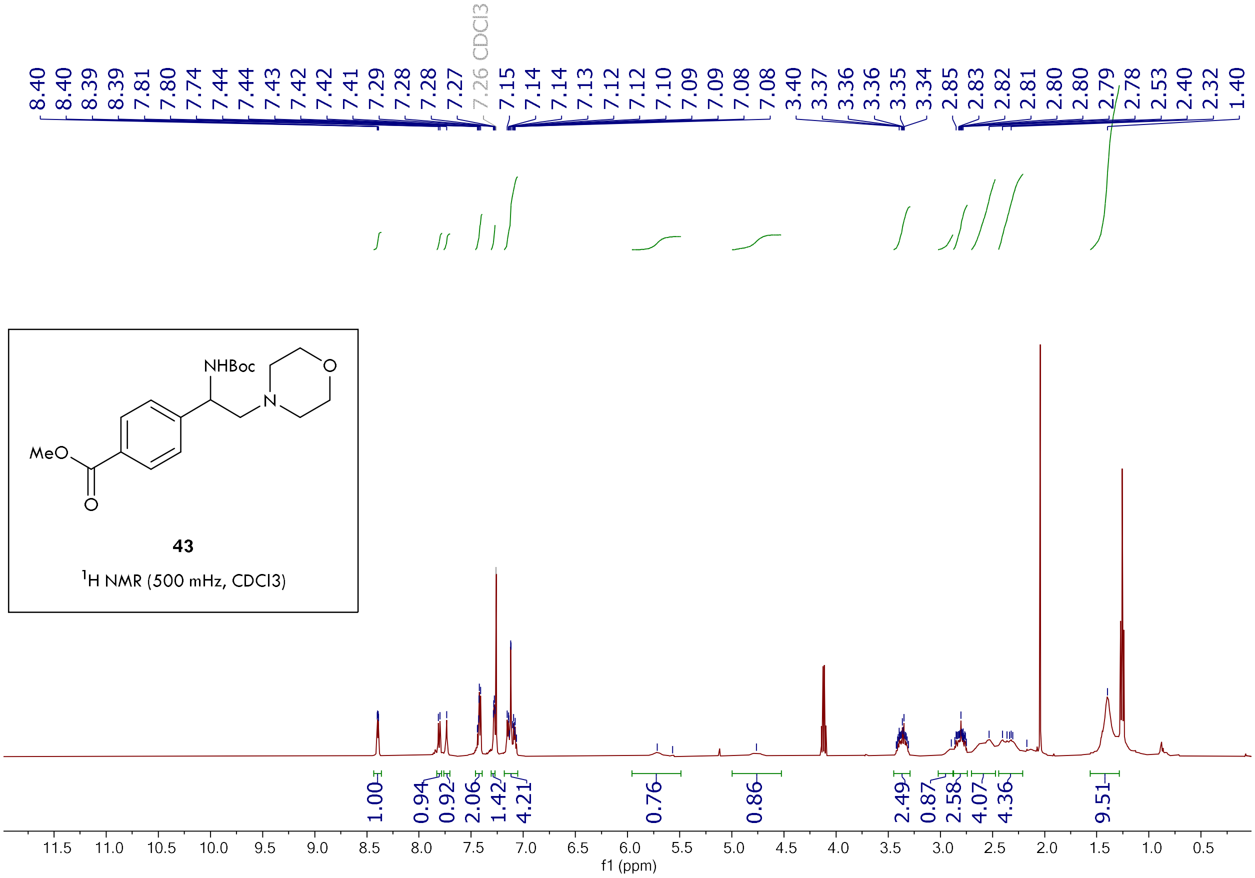

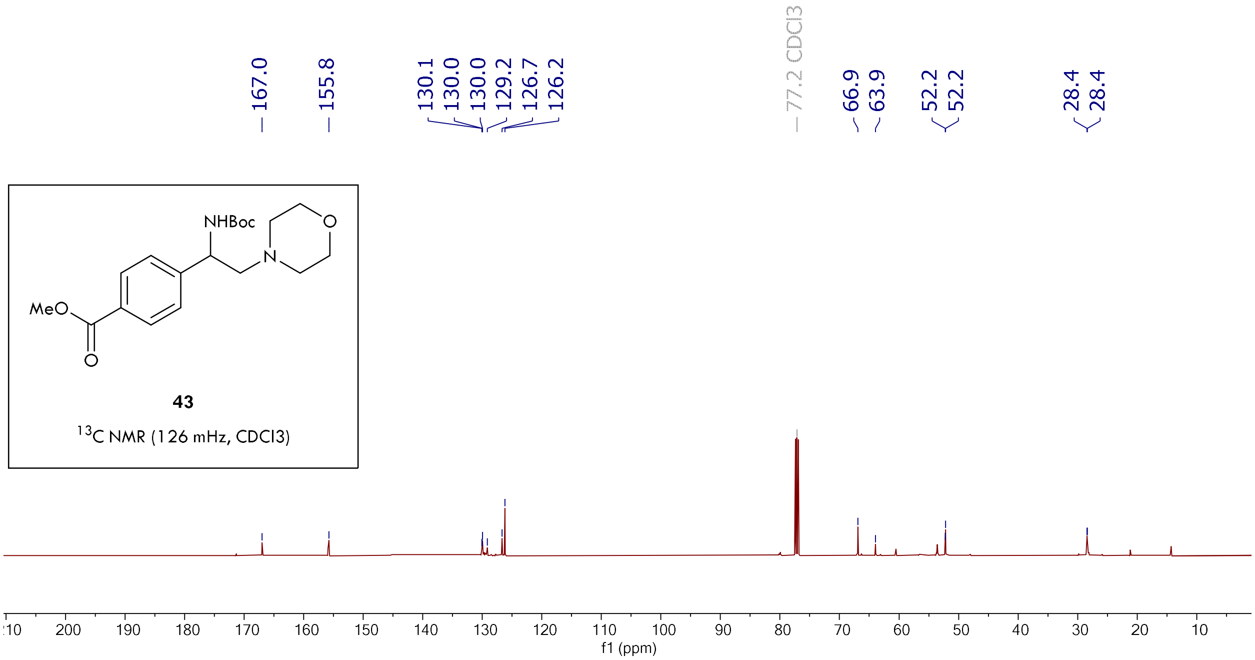

tert-butyl (1-(benzo[b]thiophen-5-yl)-2-(4-(8-chloro-5,6-dihydro-11H-benzo[5,6]cyclohepta[1,2-b]pyridin-11-ylidene)piperidin-1-yl)ethyl)carbamate (44)

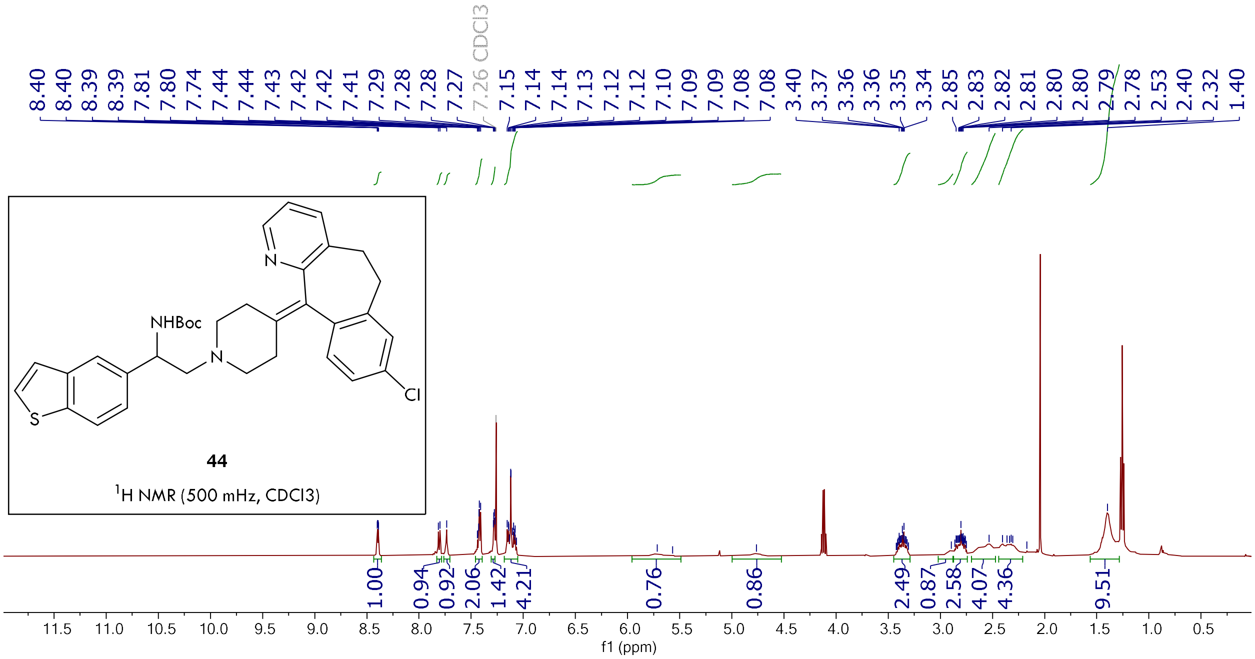

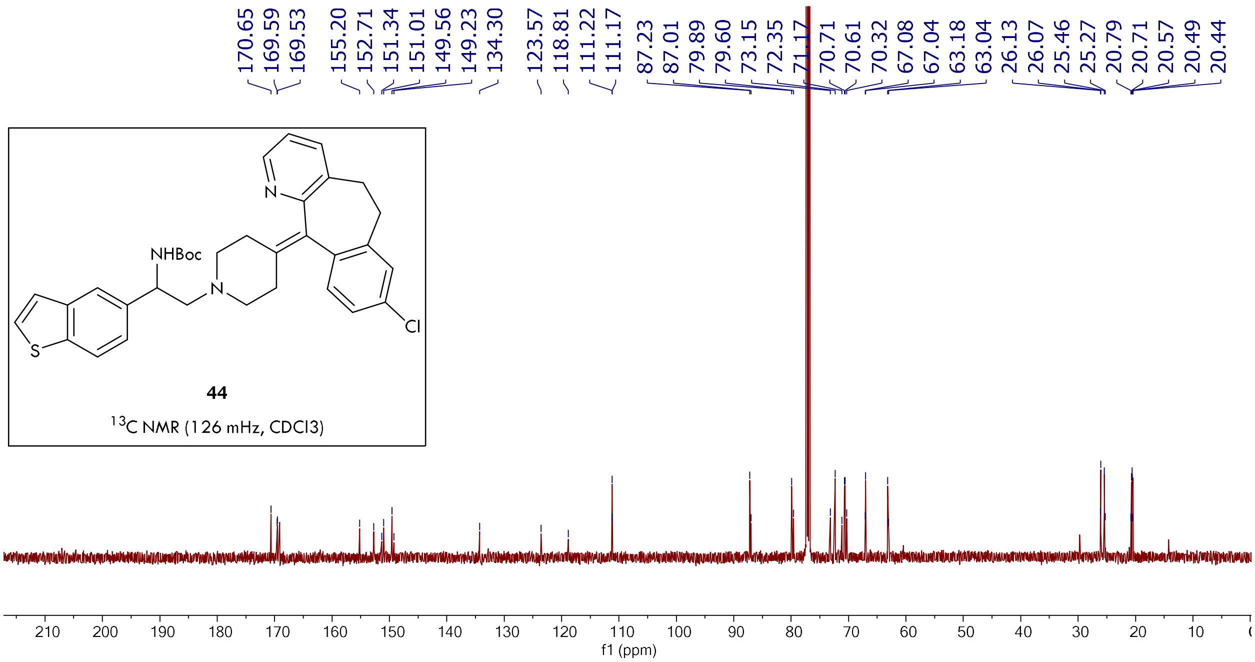

Supplement: SI [file NIHMS2175324-supplement-SI.pdf]
